# Development of atropisomeric 5*N*-sulfonyl-1,5-benzodiazepin-2-ones with high inhibitory activity against neutrophil elastase

**DOI:** 10.1039/d6md00430j

**Published:** 2026-08-07

**Authors:** Hidetsugu Tabata, Yoshiki Miyata, Yoshio Kusakabe, Hideyo Takahashi, Hideaki Natsugari, Tetsuta Oshitari

**Affiliations:** a Faculty of Pharmaceutical Sciences, Teikyo University 2-11-1 Kaga Itabashi-ku Tokyo 173-8605 Japan tabata.hidetsugu.ad@teikyo-u.ac.jp; b Faculty of Pharmaceutical Sciences, Tokyo University of Science 6-3-1, Niijuku Katsushika-ku Tokyo 125-8585 Japan; c Faculty of Pharmacy, Niigata University of Pharmacy and Medical and Life Sciences 265-1 Higashijima, Akiha-ku Niigata 956-8603 Japan

## Abstract

Novel 5*N*-sulfonyl-1,5-benzodiazepin-2-one derivatives (2b, d) with strong inhibition of neutrophil elastase have been developed. The atropisomers of 2d, based on the rotation of the two interlocking sp^2^–sp^2^ (Ar–N) axes [(a*R*, a*R*) and (a*S*, a*S*)], were stably isolated at room temperature by HPLC using a chiral column. A significant difference was observed in the inhibitory activity between the atropisomers [(+)-2d, (−)-2d, and (±)-2d], and the eutomer was revealed to be (+)-2d. The X-ray analysis revealed that the configuration of the eutomer (+)-2d was (a*R*, a*R*). The eutomer exhibited approximately 10-fold higher inhibitory activity than the currently used lead compound sivelestat (ONO-0546) (1), indicating its potential as a treatment for acute lung injury. Docking studies with target proteins supported our hypothesis of high activity.

## Abstract 2

Novel *N*-sulfonyl-1,5-benzodiazepin-2-one derivatives (2d) were designed based on Sivelestat (1). The a*R*, a*R* atropisomer exhibited approximately 10-fold higher inhibitory activity against neutrophil elastase than Sivelestat.

## Graphical abstract

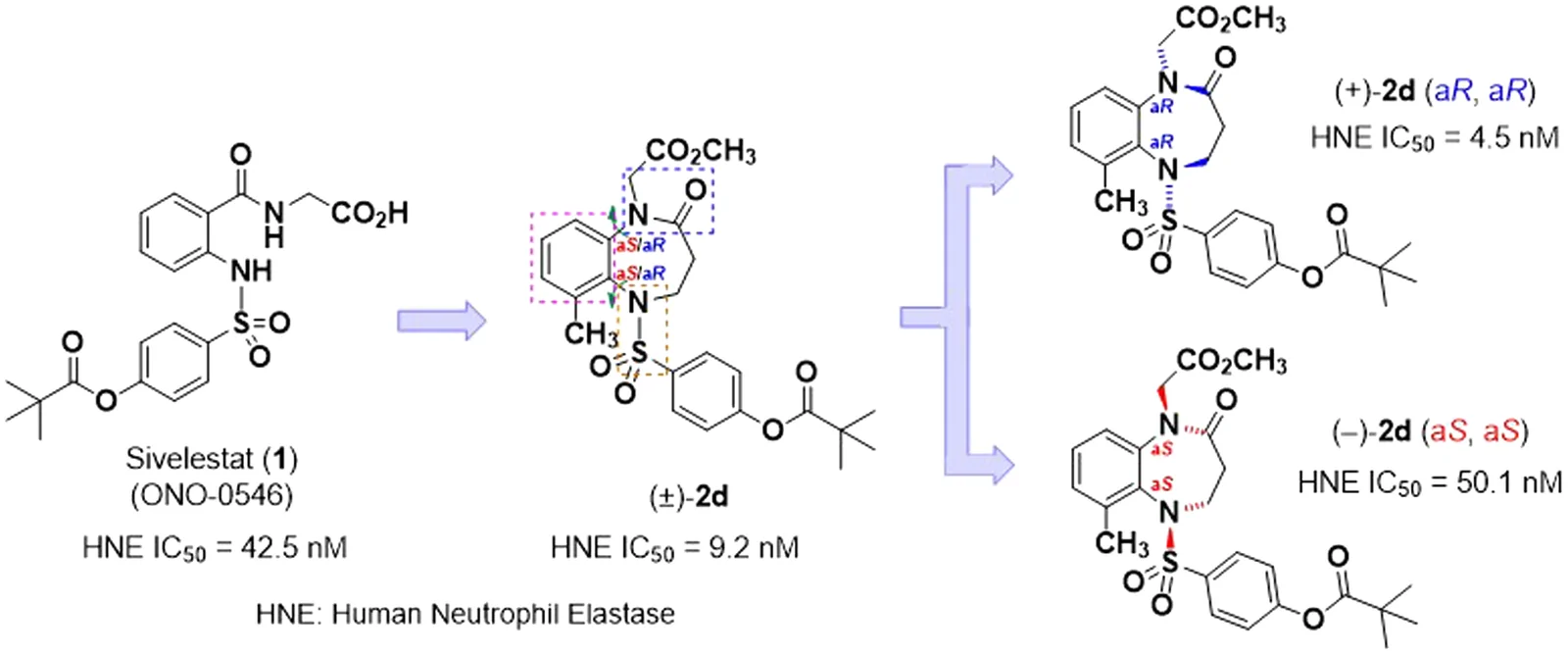

## Introduction

**Fig. 1:**
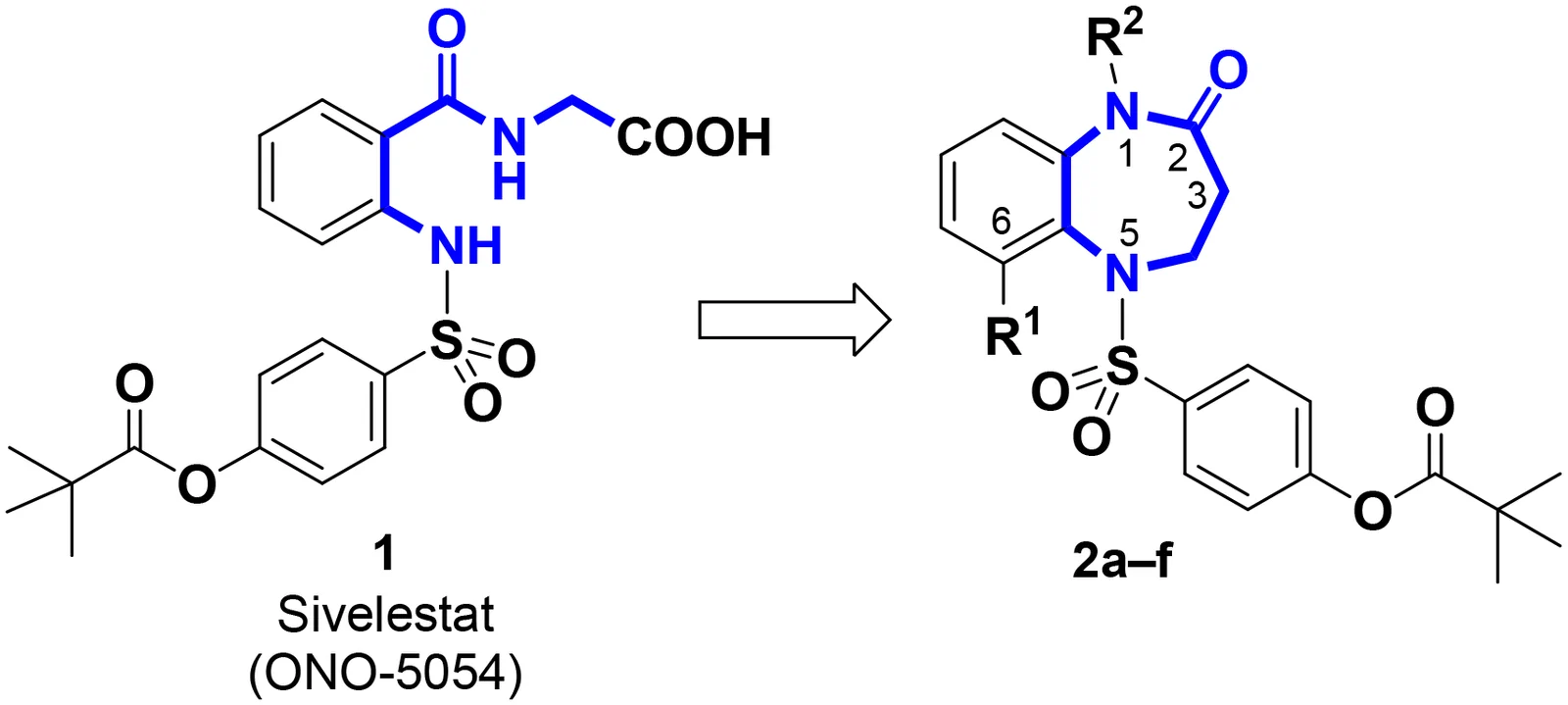
Structures of Sivelestat (1) and derivatives 5*N*-sulfonyl-1,5-benzodiazepin-2-one (2a–f).

**Fig. 2:**
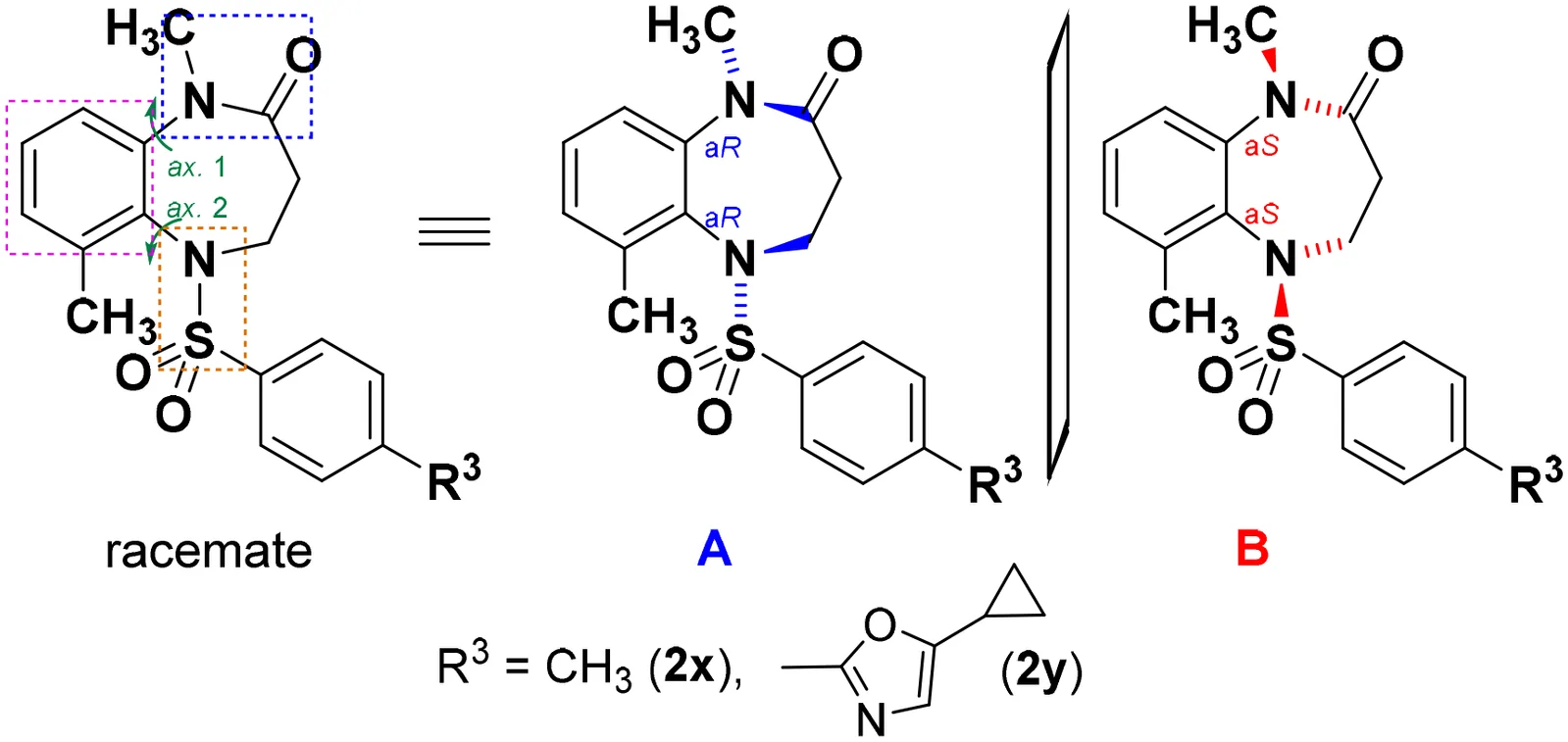
Atropisomers (A and B) of 5*N*-sulfonyl-1,5-benzodiazepin-2-one derivatives (2x, y) based on rotation around the Ar–N axes.

Neutrophil elastase (NE) is related to diseases such as severe acute pancreatitis,^1–3^ disseminated intravascular coagulation (DIC),^4–7^ acute lung injury (ALI),^8–12^ and obesity.^13,14^ Many researchers have extensively studied NE as a therapeutic target.^15–17^ One of these diseases is ALI, which occurs through systemic inflammatory response syndrome (SIRS) and is induced by NE, which increases neutrophil chemotactic factors. ONO-5046 (1) was developed by Ono Pharmaceutical Co., Ltd. in 2002 for the treatment of ALI and released as sivelestat sodium (Elaspol®100) (Fig. 1).^18–21^ It is a widely used drug that selectively inhibits elastase released from neutrophils, thereby suppressing increased protein permeability in pulmonary vascular endothelial cells and alveolar epithelial cells and disrupting the vascular basement membrane. However, since the approval of Sivelestat, the development of novel neutrophil elastase inhibitors for the treatment of ALI has been limited, and there remains an unmet need for effective new therapeutic agents. Therefore, we attempted to develop novel neutrophil elastase inhibitors incorporating axial chirality as a key factor contributing to potent inhibitory activity. We focused on the structure of sivelestat, which has two sp^2^–sp^2^ axes consisting of three planes: a benzene ring, an amide bond, and a sulfonamide bond. Moreover, the fact that cyclization of this structure results in a benzodiazepine scaffold is also noteworthy. Benzodiazepines are widely recognized as important scaffolds for biologically active compounds.^22–24^ In previous studies, we reported the stereochemistry and biological activity based on axial chirality in benzodiazepinone^25–28^ and dibenzazepinone^29,30^ derivatives with acyl or sulfonyl groups at the ring nitrogen. For example, although 5*N*-sulfonyl-1,5-benzodiazepin-2-one 2x, y (Fig. 2) have two sp^2^–sp^2^ axes, only one atropisomer exists because the axes move like gears (Fig. 2A and B).^31,32^ Furthermore, it has been elucidated that target molecules recognize axial chirality, and clear differences in biological activity are observed between atropisomers.^33^ Accordingly, the construction of *N*-sulfonylbenzodiazepinone scaffolds, as shown in.

Fig. 1, affords atropisomers and may lead to the discovery of highly potent compounds. Herein, we report the atropisomeric properties and inhibitory activity of newly designed 5*N*-sulfonyl-1,5-benzodiazepin-2-one derivatives with a pivaloyloxy group as the R^3^ functional group (2a–f). This cyclization-based structural transformation provides the advantage of fixing the rotational conformation of the amide and sulfamoyl bonds in sivelestat.

## Results and discussion

**Scheme 1:**
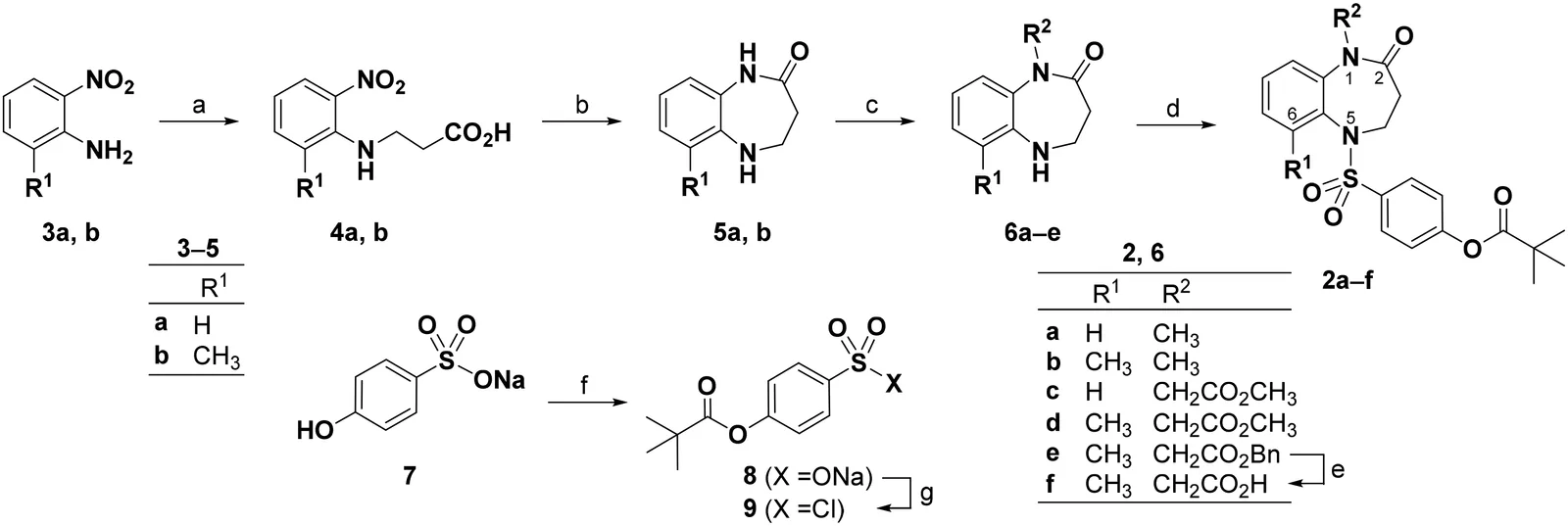
Synthesis of 5*N*-sulfonyl-1,5-benzodiazepin-2-one derivatives (2a–f). (a) Acrylic acid, H_2_SO_4_, 80 °C; (b) Zn, H_3_PO_4_, 1,4-dioxane, 110 °C; (c) R^2^-X, NaH, DMF, RT; (d) 9, pyridine, CH_2_Cl_2_, RT; (e) Pd/C, EtOH, H_2_, RT; (f) pivalic acid, TFA, RT; (g) (COCl)_2_, DMF, CH_2_Cl_2_, RT.

**Scheme 2:**
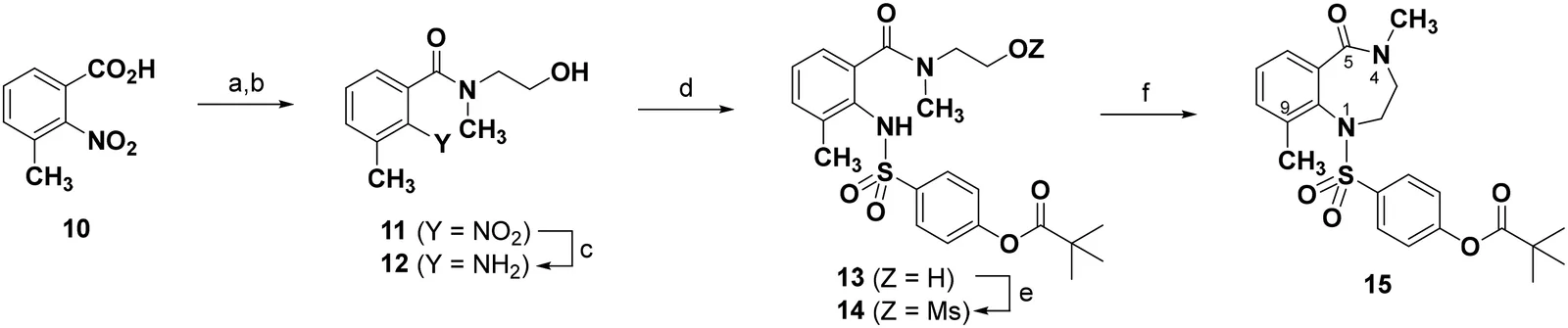
Synthesis of 1*N*-sulfonyl-1,4-benzodiazepin-5-one derivative (15). (a) (COCl)_2_, DMF, CH_2_Cl_2_, RT.; (b) *N*-methylaminoethanol, Et_3_N, THF, RT; (c) Pd/C, EtOH, H_2_, RT; (d) 9, pyridine, CH_2_Cl_2_, RT; (e) MsCl, Et_3_N, CH_2_Cl_2_, RT; (f) NaH, THF, 90 °C.

The synthesis of 1,5-benzodiazepin-2-ones (2a–f) is shown in Scheme 1. In accordance with our previous report,^28^ 1,5-benzodiazepin-2-ones 5a and 5b were synthesized from nitroanilines in two steps. *N*-Alkylation with an alkyl halide (R-X) was performed to give derivatives 6a–e. Sodium *p*-hydroxysulfonate was esterified with pivalic acid and then converted to acid chloride with oxalyl chloride.^34,35^ 1,5-Benzodiazepin-2-ones 6a–e were led to compound 2a–e*via N*-sulfonation with *p*-pivaloyloxybenzenesulfonyl chloride 9. Deprotection of 2e by reduction afforded carboxylic acid 2f. In addition, 1,4-benzodiazepin-5-one 15 (a skeletal analog of 2) was synthesized from 3-methyl-2-nitrobenzoic acid (10) and treated with ethanolamine *via* an acid chloride to form amide 11 (Scheme 2). The reduction of the nitro group of 11, followed by *N*-sulfonylation with sulfonyl chloride 9, resulted in 13. After mesylation of the hydroxyl group of 13, intramolecular amidation was performed in the presence of sodium hydride, yielding 15.

We evaluated the NE inhibitory activity of the synthesized compounds (Table 1). Assessments were performed using an NE Inhibition Assay Kit (abcom®, AB118971). The inhibitory activity of the synthesized sivelestat 1 was generally consistent with that reported in the literature (IC_50_: 42.5 nM).^36^ Compound 2a inhibited elastase with an IC_50_ value of 26.3 nM, which was higher than that of 1.

**Table 1:** Neutrophil elastase inhibitory activities of synthetic compounds (2a–f, 15)

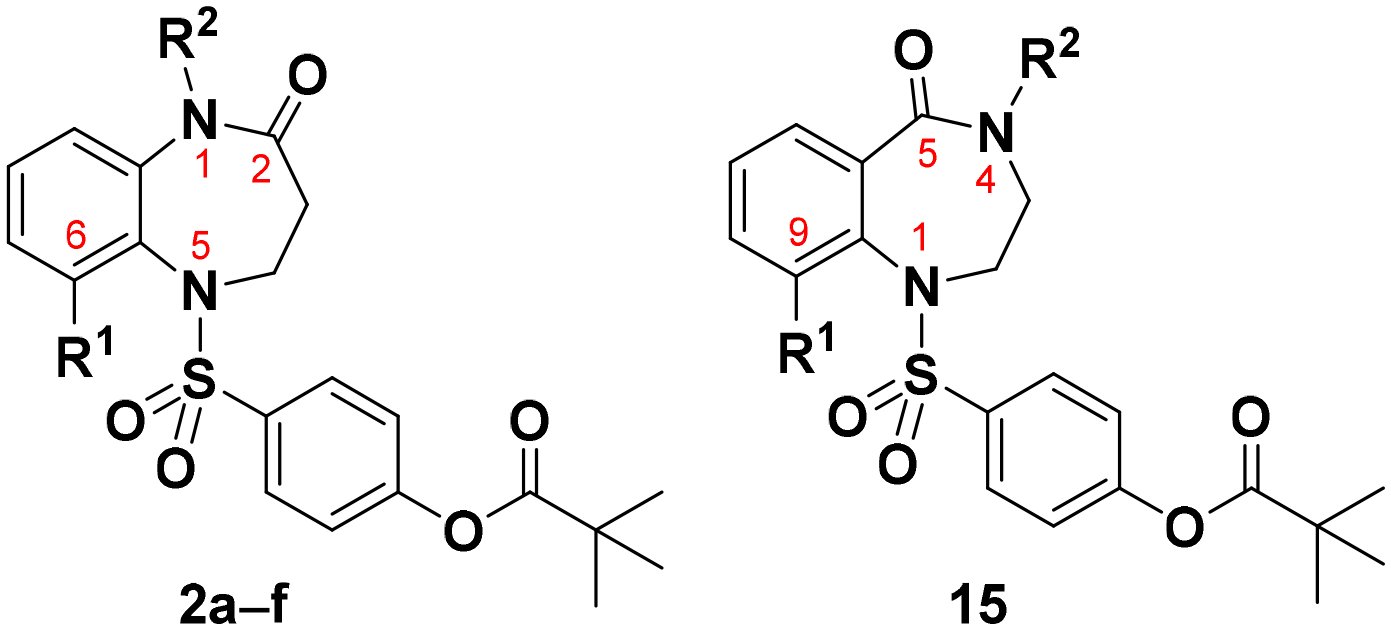			
Compound	R^1^	R^2^	IC_50_ (nM)
2a	H	CH_3_	26.3
2b	CH_3_	CH_3_	17.2
2c	H	CH_2_CO_2_CH_3_	45.2
2d	CH_3_	CH_2_CO_2_CH_3_	9.2
2e	CH_3_	CH_2_CO_2_Bn	356.0
2f	CH_3_	CH_2_CO_2_H	61.3
15	CH_3_	CH_3_	68.0
Sivelestat (1)			42.5

Furthermore, compound 2b, which had a methyl group at the C6-position, exhibited an inhibitory activity twice as potent as that of 1, with an IC_50_ value of 17.2 nM. In contrast, the inhibitory activity of skeletal analog (15), which contains a 1,4-benzodiazepin-5-one ring, was significantly lower than that of 1. The introduction of a carboxymethyl group at the N1-position (2f) was expected to result in high efficacy because of its structural similarity to sivelestat. However, the IC_50_ value of 2f was low (61.3 nM). Interestingly, compound 2d, in which the carboxymethyl group of 2f was replaced with a methyl ester, inhibited the compound with an IC_50_ value of 9.2 nM, showing 4-fold more potency than that of 1. Contrary to our expectations, 2c (IC_50_: 45.2 nM) was less active than 2a (IC_50_: 26.3 nM), indicating that the activity was reduced in the absence of a methyl group at the C6-position even though methyl acetate was introduced at the N1-position. Benzyl ester 2e exhibited low solubility and inhibitory activity, with an IC_50_ of 356 nM.

**Fig. 3:**
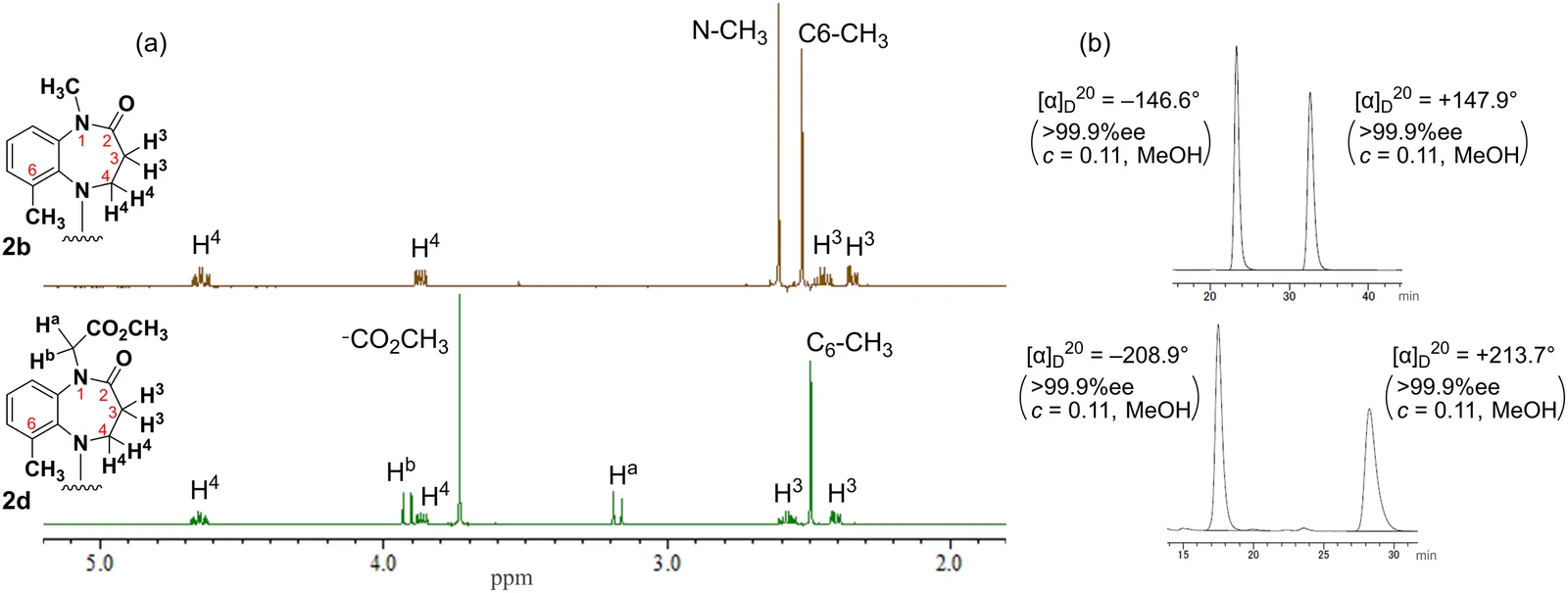
(a) ^1^H-NMR (600 MHz, CDCl_3_) spectra and (b) HPLC chromatogram resulted from the analysis of 2b and 2d using chiral column. 2b (hexane/EtOH = 1/1 as eluent; flow rate 0.3 mL min^−1^); 2d (hexane/EtOH = 4/1 as eluent; flow rate 0.18 mL min^−1^).

**Fig. 4:**
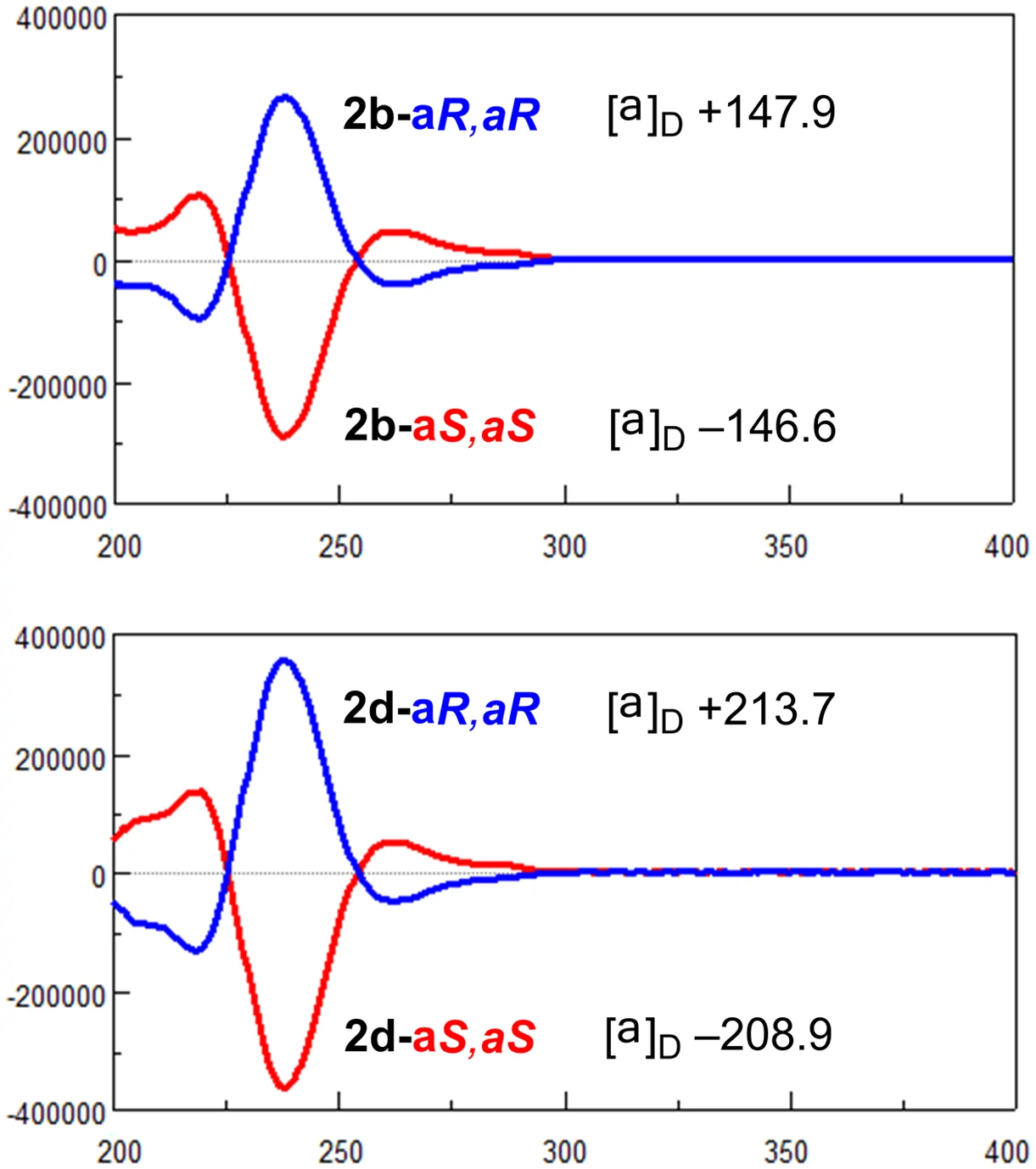
CD spectra and [*a*]_D_ data of 2b and 2d atropisomers.

**Fig. 5:**
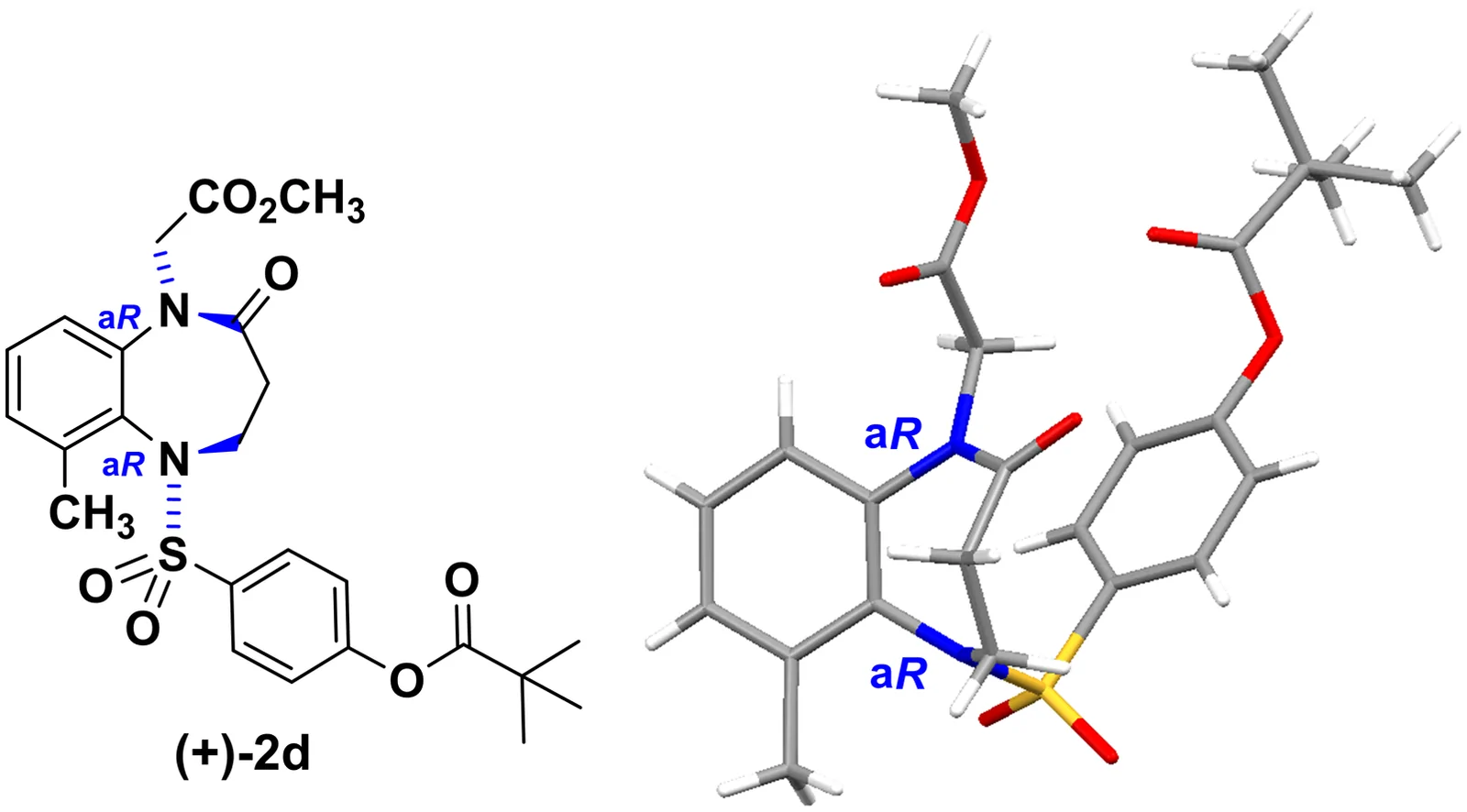
X-ray crystal structure of isolated (+)-atropisomer of 2d.

Some of the evaluated derivatives exhibited higher inhibitory activity than sivelestat (1); among them, derivatives 2b and 2d, which possess a methyl group at the C6-position, may be isolated as atropisomers based on the Ar–N axes. The introduction of a substituent at the 6-position of 5*N*-sulfonyl-1,5-benzodiazepin-2-ones induces steric hindrance, which enables the isolation of stable atropisomers. We previously found that atropisomers based on rotation around the Ar–N axis can be isolated from C6-methyl-5*N*-tosyl-1,5-benzodiazepin-2-one derivatives.^29^ In anticipation of further improvement in activity, we attempted to isolate atropisomers for compounds 2b and 2d. The NMR spectra of 2b and 2d displayed diastereotopic methylene protons, indicating the presence of atropisomers (Fig. 3a). Compounds 2b and 2d were analyzed by HPLC using a chiral column and were separated into two peaks with equal ratios (Fig. 3b). The optical properties (specific rotation, αD) and CD spectra of the isolated atropisomers were measured, and the results are shown in Fig. 4. Fortunately, X-ray crystallography of the (+)-atropisomer of 2d was successfully performed, revealing that the conformations of (+)-2d were a*R* and a*R* (Fig. 5). The circular dichroism spectra of atropisomers 2b and 2d were remarkably similar, suggesting that the conformation of (+)-2b with Ar–N axes was also a*R*, a*R*.

Next, the thermodynamic stabilities of the separated atropisomers 2b and 2d were investigated. The low stability of the asymmetric axis is often an obstacle in the development of axially chiral ligands.

**Fig. 6:**
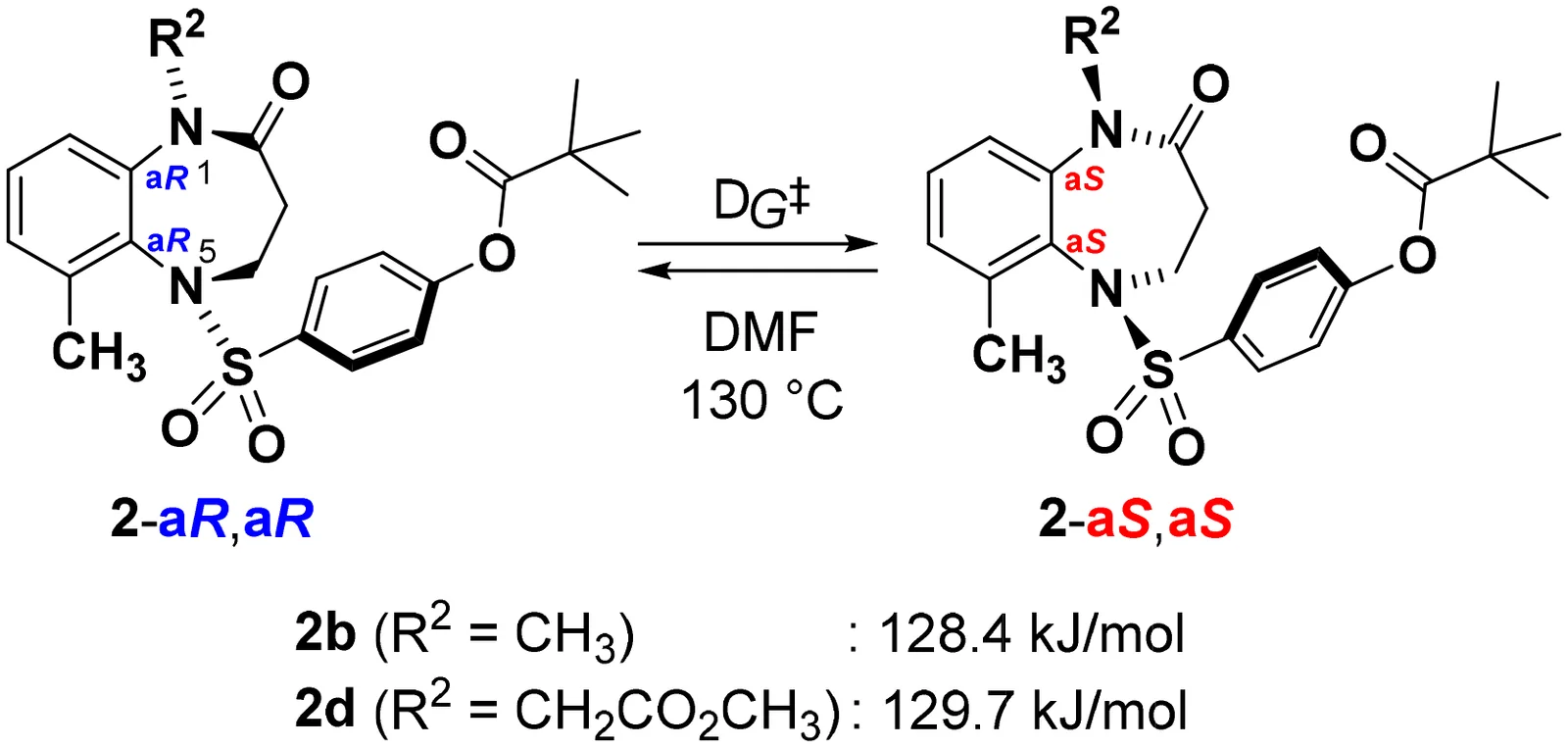
Thermodynamic stability of 2b and 2d atropisomers. A rotational barrier (D*G*^‡^) exists on the Ar–N axes.

However, in our previous report,^31^ we demonstrated that *N*-sulfonyl-1,5-benzodiazepin-2-one derivatives have high thermodynamic stability. Therefore, derivatives 2b and 2d were also expected to have high rotational barriers around the Ar–N axes. One of the isolated pure atropisomers was heated in toluene, and the isomerization rate over time was measured. The rotational barrier (Δ*G*^‡^) around the Ar–N axes was calculated from the rate constant using the Eyring equation (Fig. 6).^37^ The isomerization of the atropisomer of 2b at 37 °C remained below 0.5% even after 6 h, and no isomerization occurred in 2d. The atropisomer of 2b was almost racemized at 130 °C for 2.5 h, and Δ*G*^‡^ was calculated to be 128.4 kJ mol^−1^. On the other hand, the stability of 2d was slightly higher than that of 2b, and racemization took place after approximately 4 h at 130 °C, with a Δ*G*^‡^ of 129.7 kJ mol^−1^ (the graphic charts of isomerization over time are shown in the SI). The slight difference in stability was attributed to the size of the N1 substituent between 2b and 2d. Neither atropisomer 2b nor 2d isomerized at physiological temperatures, demonstrating their sufficient stability for use as axially chiral ligands.

The NE-inhibitory activities of isolated atropisomers 2b and 2d are listed in Table 2. For 2b, 2b-a*R*, a*R* showed an IC_50_ value of 9.7 nM, whereas 2b-a*S*, a*S* had an IC_50_ value of 33.5 nM. Similarly, the enantiomers of 2d also showed clear differences in activity, and the IC_50_ value of 2d-a*R*, a*R* was significantly higher than that of 2d-a*S*, a*S* (4.5 nM *vs.* 50.1 nM). These results indicate that the a*R*, a*R* conformations of the Ar–N axis contribute to the elastase-inhibitory activity. Moreover, 2b-a*R*, a*R* and 2d-a*R*, a*R* both exhibited a 2-fold inhibitory activity than the racemate. Thus, an improvement inactivity was achieved by separating the atropisomers, resulting in an approximate 10-fold increase in inhibitory activity compared to that of sivelestat (1). To evaluate the selectivity of the racemates of 2b and 2d and their corresponding enantiomers, inhibitory activity assay against trypsin were examined. No inhibitory activity against trypsin was observed at 1 μM for any of the compounds, demonstrating the high selectivity of 2b and 2d for neutrophil elastase (see SI for data).

**Table 2:** Neutrophil elastase inhibitory activities of racemate and atropisomers of 2b and 2d

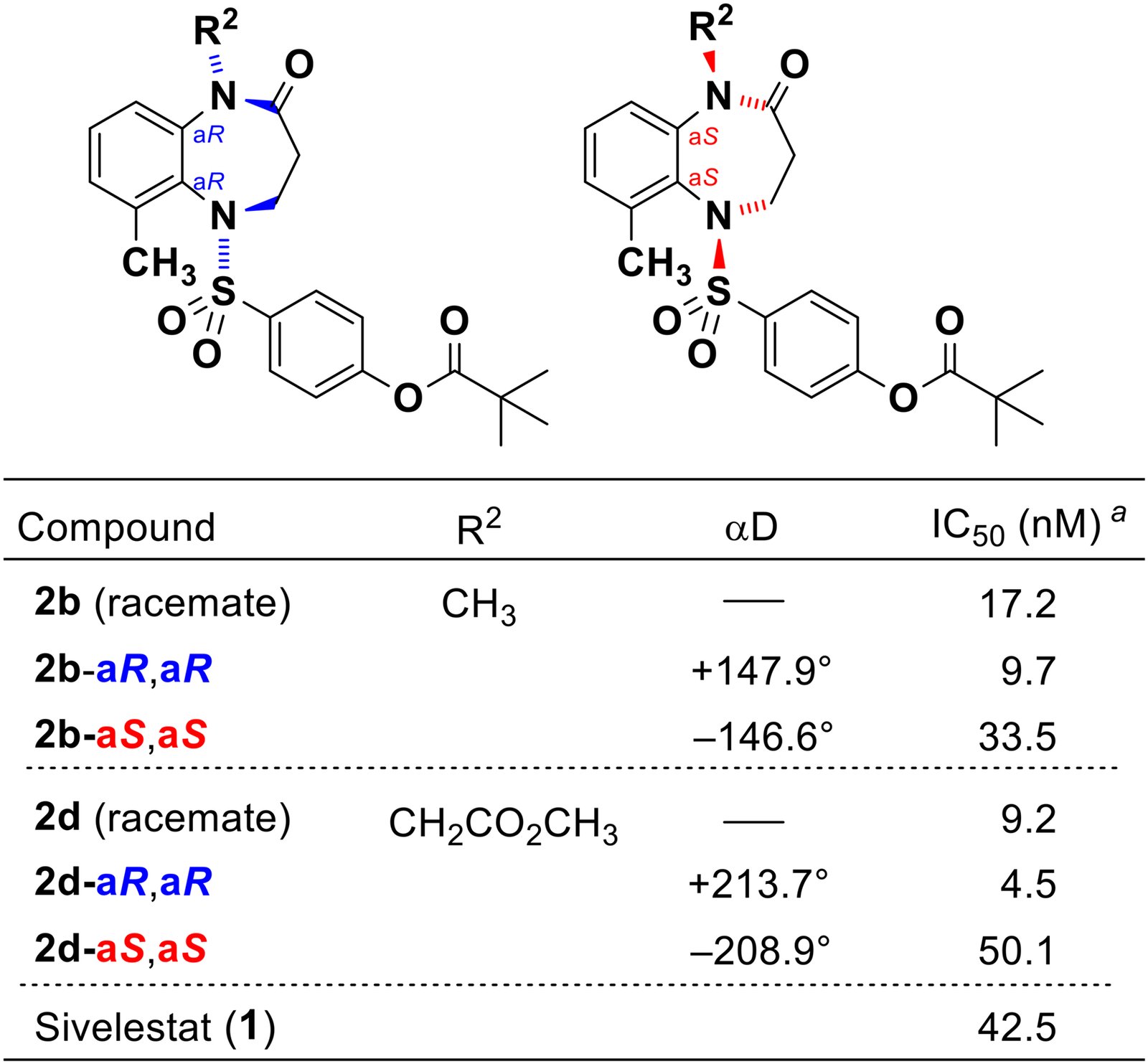

**Fig. 7:**
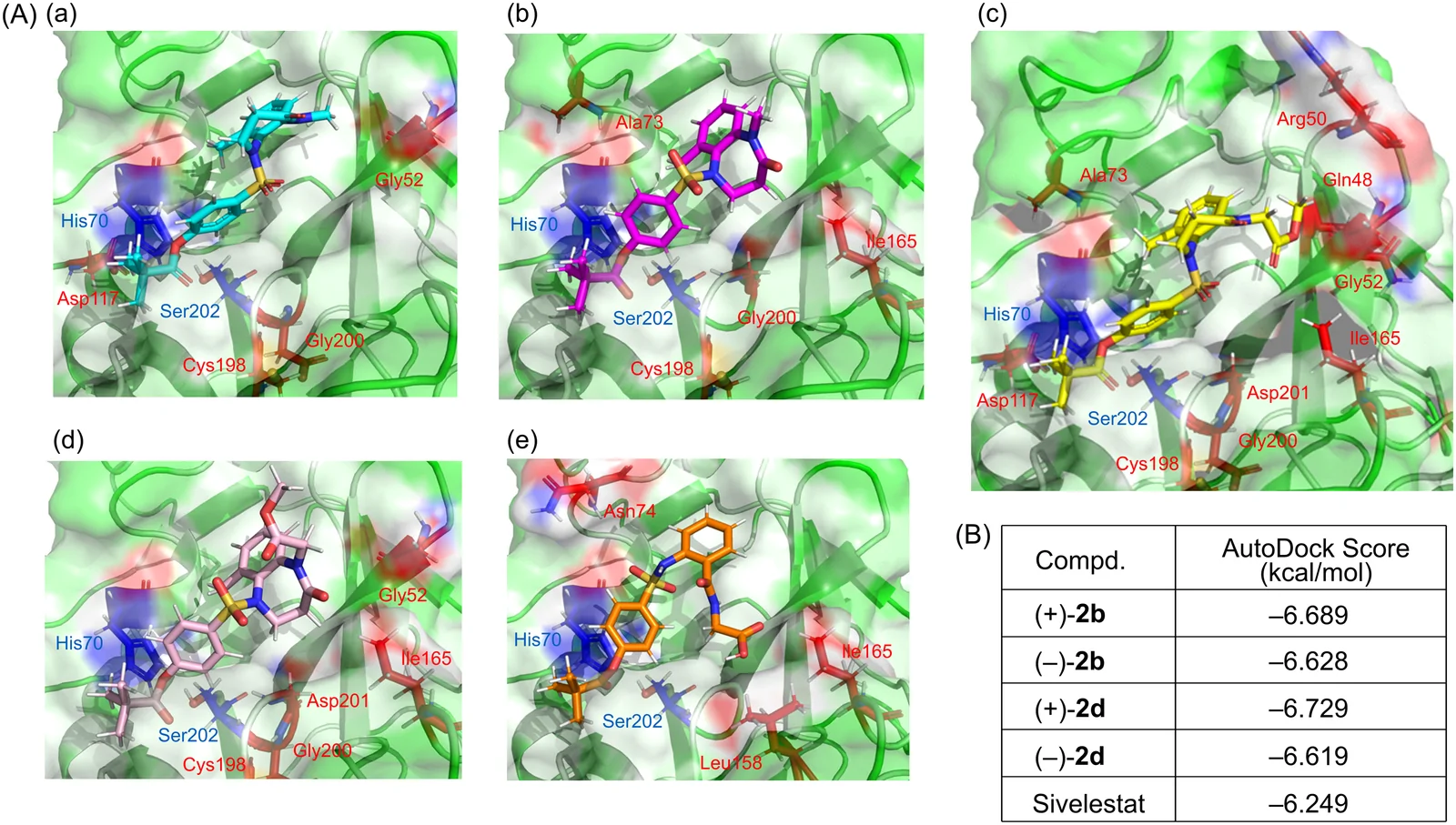
(A) Docking models of (+)-2b (a), (−)-2b (b), (+)-2d (c), (−)-2d (d) and Sivelestat (e) with human Neurophil Elastase (PDB code: 1B0F) and (B) their binding energies. Amino acids that specifically interacted with each compound are highlighted in red.

We performed docking simulations with the target proteins using AutoDock Vina 1.5.7 (ref. 38) to investigate the differences in biological activity between the enantiomers. According to previously reported studies,^36,39^ the active site is defined as a space containing His70 and Ser202 that interact with the pivaloyloxy group. We considered Ser202, His70, Asp117, and Gly200 as four key amino acid residues and investigated their interactions based on a model in which the pivaloyloxy group was fixed through hydrogen bonding with the corresponding amino acid residues, particularly His70 and Ser202. In these models, the a*R*, a*R* forms (eutomers), (+)-2b and (+)-2d, showed interactions with a larger number of amino acid residues compared to the a*S*, a*S* forms (distomers). Notably, the observed interaction with Asp117 may account for their strong activity of (+)-2b and (+)-2d. Furthermore, in 2d, (+)-2d interacts with Arg50 and Gln48, possibly due to the near44.by methyl ester moiety. Conversely, the lack of interactions with these amino acid residues (Arg50 and Gln48) in (−)-2d can be explained by the docking structure in which the methyl ester moiety of (−)-2d was inverted from that of (+)-2d. The low IC_50_ value of Sivelestat was suggested to result from the lack of interactions with both Asp117 and Gly200. The affinity of the compounds for neutrophil elastase was compared based on the calculated binding energies. The docking scores correlated well with the inhibitory activity, and the binding energies of the a*R* and a*R* forms were lower than those of the a*S* and a*S* forms (Fig. 7B). In particular, (+)-2d, which has strong inhibitory activity, showed a strong affinity at a low binding energy (−6.729 kcal mol^−1^). Additionally, sivelestat did not interact sufficiently with amino acid residues and exhibited a high binding energy (−6.249 kcal mol^−1^). As shown in the docking simulation, the conformation based on the rotation of the Ar–N axes contributed to elastase binding. In other words, the a*R* and a*R* forms exhibited higher inhibitory activity than the a*S*, a*S*, and sivelestat forms because they had a more suitable conformation for the elastase active site.

## Conclusions

In conclusion, our approach to constructing a diazepinone scaffold from sivelestat successfully led to the development of novel 5*N*-sulfonyl-1,5-benzodiazepin-2-one derivatives with high inhibitory activity. In addition, atropisomers with rotations around the Ar–N axes were stably isolated, and clear differences in their activities were observed. The conformation of the Ar–N axes of the eutomer was a*R*, a*R*, which exhibited up to approximately 10-fold more potent activity than the currently used sivelestat. Docking studies suggested that a conformation based on axial chirality contributes to this interaction. These findings indicate the conformation based on the axial chirality of the benzodiazepinone scaffold makes a significant contribution to neutrophil elastase inhibitory activity and suggest that these compounds can serve as novel therapeutic agents for ALI. Moreover, as neutrophil elastase is involved not only in ALI but also in other diseases, the developed series of compounds is expected to have potential applicability to additional disease indications. Further structural optimization and cell-based assays are being conducted.

## Experimental section

### General experimental

Materials were obtained from commercial suppliers. NMR spectra were recorded on a JEOL spectrometer at 400 MHz or 600 MHz for ^1^H NMR, and 100 MHz or 150 MHz for ^13^C NMR. Chemical shifts are given in parts per million (ppm) downfield from tetramethylsilane as an internal standard, and coupling constants (*J*) are reported in Hertz (Hz). Splitting patterns are abbreviated as follows: singlet (s), doublet (d), triplet (t), quartet (q), multiplet (m), and broad (br). IR spectra were recorded on a FT-IR spectrometer equipped with ATR (Diamond). The high-resolution mass spectra (HRMS) were obtained with an ionization mode of ESI. Melting points were recorded on a melting point apparatus and are uncorrected. Optical rotations were determined with a digital polarimeter. Analytical thin-layer chromatography was performed on precoated, glass-backed silica gel plates. Column chromatography was performed using silica gel (45–60 μm). Extracted solutions were dried over anhydrous MgSO_4_ or Na_2_SO_4_. Solvents were evaporated under reduced pressure.

### Synthesis and characterization of compounds

#### 1-Methyl-5-(*p*-pivaloyloxybenzenesulfonyl)-1,3,4,5-tetrahydro-2*H*-1,5-benzodiazepin-2-one (2a)

To a stirred solution of 1-methyl-1,3,4,5-tetrahydro-2*H*-1,5-benzodiazepin-2-one 6a (67 mg, 0.38 mmol) in CH_2_Cl_2_ (4.2 mL) at 0 °C under argon were added pyridine (77 mL, 0.96 mmol) and *p*-pivaloyloxybenzenesulfonyl chloride 9 (126 mg, 0.46 mmol). After being stirred at 25 °C for 15 h, the mixture was treated with H_2_O and extracted with CH_2_Cl_2_. The extract was dried, and concentrated. The concentrate was purified by column chromatography (silica gel, ethyl acetate/hexane = 1/4) to afford 2a (157 mg, 99%) as white solid: mp 137–139 °C: ^1^H NMR (600 MHz, CDCl_3_) *δ* 7.63 (dd, *J* = 1.3, 7.5 Hz, 1H, ArH), 7.58 (d, *J* = 8.9 Hz, 2H, ArH), 7.39 (ddd, *J* = 1.3, 7.5, 7.5 Hz, 1H, ArH), 7.29, (ddd, *J* = 1.3, 7.5, 8.2 Hz, 1H, ArH), 7.11 (d, *J* = 8.9 Hz, 2H, ArH), 7.07 (dd, *J* = 1.3, 8.2 Hz, 1H, ArH), 4.71 (br, 1H, CH_2_), 3.94 (br, 1H, CH_2_), 2.43 (s, 3H, NCH_3_), 2.52 (br, 2H, CH_2_), 1.33 (s, 9H, C(CH_3_)_3_); ^13^C{^1^H} NMR (150 MHz, CDCl_3_) *δ* 176.4, 154.4, 142.8, 137.1, 133.0, 130.0, 129.7, 128.4, 126.6, 122.8, 122.3, 52.0, 39.1, 34.3, 33.9, 27.0; IR (ATR) 2937, 2031, 1663, 1347, 1084 cm^−1^; HRMS (ESI) *m*/*z*: [M + H]^+^ calcd for: C_21_H_25_N_2_O_5_S 417.1479; found, 417.1480.

#### 1,6-Dimethyl-5-(*p*-pivaloyloxybenzenesulfonyl)-1,3,4,5-tetrahydro-2*H*-1,5-benzodiazepin-2-one (2b)

Compound 2b was prepared from 1,6-dimethyl-1,3,4,5-tetrahydro-2*H*-1,5-benzodiazepin-2-one 6b (82 mg 0.43 mmol) according to a similar procedure as described for the preparation of 6a from 2a. White solid (45 mg, 24%): mp 72–74 °C: ^1^H NMR (600 MHz, CDCl_3_) *δ* 7.67 (d, *J* = 8.7 Hz, 2H, ArH), 7.29 (dd, *J* = 7.7, 7.9 Hz, 1H, ArH), 7.21, (dd, *J* = 0.8, 7.7 Hz, 1H, ArH), 7.17 (d, *J* = 8.7 Hz, 2H, ArH), 6.92 (dd, *J* = 0.8, 7.9 Hz, 1H, ArH), 4.65 (ddd, *J* = 5.2, 13.4, 13.7 Hz, 1H, CH_2_), 3.87 (ddd, *J* = 0.8, 7.0, 13.4 Hz, 1H, CH_2_), 2.60 (s, 3H, NCH_3_), 2.52 (s, 3H, CH_3_), 2.45 (ddd, *J* = 7.0, 13.7, 13.8 Hz, 1H, CH_2_), 2.34 (ddd, *J* = 0.8, 5.2, 13.8 Hz, 1H, CH_2_), 1.35 (s, 9H, C(CH_3_)_3_); ^13^C{^1^H} NMR (150 MHz, CDCl_3_) *δ* 176.4, 169.9, 154.4, 143.7, 142.4, 137.6, 129.3, 129.2, 128.9, 128.6, 122.4, 120.2, 51.1, 39.1, 34.2, 34.1, 27.0, 19.2; IR (ATR) 2973, 1973, 1666, 1344, 1101 cm^−1^; HRMS (ESI) *m*/*z*: [M + H]^+^ calcd for: C_22_H_27_N_2_O_5_S 431.1635; found, 431.1636.

#### Methyl 5-(*p*-pivaloyloxybenzenesulfonyl)-2,3,4,5-tetrahydro-2-oxo-1*H*-1,5-benzodiazepine-1-acetate (2c)

Compound 2c was prepared from methyl-2,3,4,5-tetrahydro-2-oxo-1*H*-1,5-benzodiazepine-1-acetate 6c (29 mg 0.12 mmol) according to a similar procedure as described for the preparation of 6a from 2a. White solid (42 mg, 72%): mp 145–146 °C: ^1^H NMR (600 MHz, CDCl_3_) *δ* 7.61 (dd, *J* = 1.3, 7.5 Hz, 1H, ArH), 7.58 (d, *J* = 8.9 Hz, 2H, ArH), 7.37 (ddd, *J* = 1.3, 7.5, 8.2 Hz, 1H, ArH), 7.31 (ddd, *J* = 1.3, 7.5, 8.2 Hz, 1H, ArH), 7.14, (d, *J* = 8.9 Hz, 2H, ArH), 7.09 (dd, *J* = 1.3, 8.2 Hz, 1H, ArH), 4.71 (br, 1H, CH_2_), 4.03 (br-d, *J* = 15.2 Hz, 1H, NCH_2_), 3.92 (br, 1H, CH_2_), 3.74 (s, 3H, CH_3_), 3.10 (br-d, *J* = 15.2 Hz, 1H, NCH_2_), 2.64 (br, 1H, CH_2_), 2.46 (br, 1H, CH_2_), 1.35 (s, 9H, C(CH_3_)_3_);, C(CH_3_)_3_^13^C{^1^H} NMR (150 MHz, CDCl_3_) *δ* 176.3, 170.4, 169.6, 154.5, 141.9, 137.1, 132.7, 130.0, 129.9, 128.3, 127.2, 122.7, 122.4, 52.4, 51.7, 49.6, 39.1, 33.6, 27.0; IR (ATR) 2973, 1976, 1746, 1214, 1109 cm^−1^; HRMS (ESI) *m*/*z*: [M + H]^+^ calcd for: C_23_H_27_N_2_O_7_S 475.1533; found, 475.1535.

#### Methyl 5-(*p*-pivaloyloxybenzenesulfonyl)-6-methyl-2,3,4,5-tetrahydro-2-oxo-1*H*-1,5-benzodiazepine-1-acetate (2d)

Compound 2d was prepared from 6d (53 mg 0.21 mmol) according to a similar procedure as described for the preparation of 6a from 2a. Colorless crystals (25 mg, 24%): mp 188–190 °C: ^1^H NMR (600 MHz, CDCl_3_) *δ* 7.66 (d, *J* = 8.7 Hz, 2H, ArH), 7.27 (dd, *J* = 7.7, 7.8 Hz, 1H, ArH), 7.23 (dd, *J* = 0.9, 7.7 Hz, 1H, ArH), 7.20 (d, *J* = 8.7 Hz, 2H, ArH), 6.92, (dd, *J* = 0.9, 7.8 Hz, 1H, ArH), 4.66 (ddd, *J* = 5.2, 13.4, 13.7 Hz, 1H, CH_2_), 3.92 (d, *J* = 17.6 Hz, 1H, NCH_2_), 3.87 (ddd, *J* = 0.7, 7.2, 13.4 Hz, 1H, CH_2_), 3.73 (s, 3H, OCH_3_), 3.17 (d, *J* = 17.6 Hz, 1H, NCH_2_), 2.57 (ddd, *J* = 7.2, 13.5, 13.7 Hz, 1H, CH_2_), 2.49 (s, 3H, CH_3_), 2.40 (ddd, *J* = 0.7, 5.2, 13.5 Hz, 1H, CH_2_), 1.37 (s, 9H, C(CH_3_)_3_); ^13^C{^1^H} NMR (150 MHz, CDCl_3_) *δ* 170.2, 169.7, 154.5, 143.0, 142.2, 137.6, 129.6, 129.5, 128.6, 122.5, 120.1, 52.4 50.8, 49.6, 33.9, 27.0, 19.1; IR (ATR) 2959, 2030, 1661, 1347, 1092 cm^−1^; HRMS (ESI) *m*/*z*: [M + H]^+^ calcd for: C_24_H_29_N_2_O_7_S 489.1690; found, 489.1692.

#### Benzyl 5-(*p*-pivaloyloxybenzenesulfonyl)-6-methyl-2,3,4,5-tetrahydro-2-oxo-1*H*-1,5-benzodiazepine-1-acetate (2e)

Compound 2e was prepared from 6e (232 mg 0.71 mmol) according to a similar procedure as described for the preparation of 6a from 2a. White solid (99 mg, 25%): mp 74–76 °C: ^1^H NMR (600 MHz, CDCl_3_) *δ* 7.64 (d, *J* = 8.7 Hz, 2H, ArH), 7.30–7.36 (m, 5H, ArH), 7.24 (dd, *J* = 7.5, 7.7 Hz, 1H, ArH), 7.2 (dd, *J* = 1.9, 7.7 Hz, 1H, ArH), 7.18 (d, *J* = 8.7 Hz, 2H, ArH), 6.87, (dd, *J* = 1.9, 7.5 Hz, 1H, ArH), 5.16 (s, 2H, CH_2_Ar), 4.68 (ddd, *J* = 5.2, 13.6, 13.7 Hz, 1H, CH_2_), 3.97 (d, *J* = 17.7 Hz, 1H, NCH_2_), 3.88 (ddd, *J* = 0.8, 7.3, 13.6 Hz, 1H, CH_2_), 3.18 (d, *J* = 17.7 Hz, 1H, NCH_2_), 2.57 (ddd, *J* = 7.3, 13.5, 13.7 Hz, 1H, CH_2_), 2.50 (s, 3H, CH_3_), 2.41 (ddd, *J* = 0.8, 5.2, 13.5 Hz, 1H, CH_2_), 1.32 (s, 9H, C(CH_3_)_3_); ^13^C{^1^H} NMR (150 MHz, CDCl_3_) *δ* 176.2, 170.2, 169.1, 154.5, 142.9, 142.3, 137.6, 135.2, 129.6, 129.4, 129.0, 128.5, 128.5, 128.2, 128.0, 122.5, 120.1, 67.1, 51.0, 49.7, 39.1, 33.9, 26.9, 19.2; IR (ATR) 2979, 1975, 1672, 1346, 1090 cm^−1^; HRMS (ESI) *m*/*z*: [M + H]^+^ calcd for: C_30_H_32_N_2_O_7_S 565.2003; found, 565.2004.

#### 5-(*p*-Pivaloyloxybenzenesulfonyl)-6-methyl-2,3,4,5-tetrahydro-2-oxo-1*H*-1,5-benzodiazepine-1-acetic acid (2f)

To a solution of 2e (24 mg, 0.042 mmol) in EtOH (1.5 mL) was added 10% palladium on carbon (2.3 mg). After the mixture was stirred at 25 °C for 9 h under a hydrogen atmosphere, the mixture was filtered. The filtrate was concentrated under vacuum, and the residue was purified by column chromatography (silica gel, methanol/ethyl acetate = 1/2) to afford 2f (19 mg, 95%) as white solid: mp 252–255 °C: ^1^H NMR (600 MHz, CD_3_OD) *δ* 7.72 (d, *J* = 8.2 Hz, 2H, ArH), 7.30 (dd, *J* = 6.8, 7.5 Hz, 1H, ArH), 7.28 (d, *J* = 8.2 Hz, 2H, ArH), 7.24 (d, *J* = 6.8 Hz, 1H, ArH), 7.17, (d, *J* = 7.5 Hz, 1H, ArH), 4.53 (ddd, *J* = 5.1, 13.1, 13.7 Hz, 1H, CH_2_), 3.96 (d, *J* = 17.2 Hz, 1H, NCH_2_), 3.85 (dd, *J* = 7.4, 13.1 Hz, 1H, CH_2_), 3.24 (d, *J* = 17.2 Hz, 1H, NCH_2_), 2.58 (ddd, *J* = 7.4, 13.4, 13.7 Hz, 1H, CH_2_), 2.41 (s, 3H, CH_3_), 2.28 (dd, *J* = 5.1, 13.1 Hz, 1H, CH_2_), 1.35 (s, 9H, C(CH_3_)_3_); ^13^C{^1^H} NMR (150 MHz, CD_3_OD) *δ* 177.6, 172.8, 156.0, 145.1, 143.0, 139.2, 130.9, 130.5, 130.1, 130.0, 123.8, 121.9, 53.5, 51.7, 40.2, 35.1, 27.3, 19.2; IR (ATR) 2923, 1974, 1583, 1339, 1098 cm^−1^; HRMS (ESI) *m*/*z*: [M + H]^+^ calcd for: C_23_H_27_N_2_O_7_S 475.1533; found, 475.1535.

#### 4,9-Dimethyl-1-(*p*-pivaloyloxybenzenesulfonyl)-1,2,3,4-tetrahydro-5*H*-1,4-benzodiazepin-5-one (15)

To a solution of 14 (61 mg, 0.12 mmol) in THF (2.3 mL) was sodium hydride (46 mg, 1.2 mmol) at 0 °C. After the mixture was stirred at 90 °C for 2 h under an argon atmosphere, H_2_O was added, and the mixture was extracted with CH_2_Cl_2_. The extract was washed with brine, dried, and concentrated. The residue was purified by column chromatography (silica gel, ethyl acetate/hexane = 1/3) to afford 15 (19 mg, 95%) as white solid: mp 192–193 °C: ^1^H NMR (600 MHz, CDCl_3_) *δ* 7.73 (d, *J* = 8.7 Hz, 2H, ArH), 7.49 (dd, *J* = 1.3, 7.5 Hz, 1H, ArH), 7.46, (dd, *J* = 1.3, 7.6 Hz, 1H, ArH), 7.36 (dd, *J* = 7.5, 7.6 Hz, 1H, ArH), 7.20 (d, *J* = 8.7 Hz, 2H, ArH), 4.12 (ddd, *J* = 5.0, 13.4, 13.6 Hz, 1H, CH_2_), 3.58 (dd, *J* = 4.1, 13.4 Hz, 1H, CH_2_), 3.30 (ddd, *J* = 4.1, 13.6, 15.3 Hz, 1H, CH_2_), 3.00 (dd, *J* = 5.0, 15.3 Hz, 1H, CH_2_), 2.54 (s, 3H, NCH_3_), 2.47 (s, 3H), 1.36 (s, 9H, C(CH_3_)_3_); ^13^C{^1^H} NMR (150 MHz, CDCl_3_) *δ* 176.4, 168.5, 154.6, 141.0, 136.3, 135.6, 134.0, 132.9, 129.3, 129.1, 127.7, 122.6, 49.9, 47.6, 39.1, 33.7, 27.0, 19.0; IR (ATR) 2971, 1976,1651, 1337, 1100 cm^−1^; HRMS (ESI) *m*/*z*: [M + H]^+^ calcd for: C_22_H_26_N_2_O_5_S 431.1635; found, 431.1637.

### X-ray crystallographic data collection

All measurements were made on a Rigaku Raxis Rapid imaging plate area detector with graphite monochromated Cu-Kα radiation. The data were collected at a temperature of −100 °C. The structure was solved by direct method SIR92 and expanded using Fourier techniques. The non-hydrogen atoms were refined anisotropically. All calculations were performed using the Crystal Structure (Crystal Structure 4.3.3) crystallographic software package. Deposition numbers 2506269 [(+)-2d] contain the supplementary crystallographic data for this paper. This data is provided free of charge by the joint Cambridge Crystallographic Data Centre and Fachinformationszentrum Karlsruhe Access Structures service.

Crystal data for (+)-2d: C_24_H_28_N_2_O_7_S_1_; mp 197–198 °C, Mr = 488.55, CuKα (*λ* = 1.54187 Å), monoclinic, *P*2_1_, colorless prism 0.200 × 0.150 × 0.030 mm, crystal dimensions *a* = 8.2604(4) Å, *b* = 9.4759(5) Å, *c* = 15.5250(8) Å, *α* = 90°, *β* = 91.749(7)°, *γ* = 90°, *T* = 173 K, *Z* = 2, *V* = 1214.65(10) Å^3^, *D*_calc_ = 1.336 g cm^−3^, *μ*CuKα = 15.853 cm^−1^, *F*_000_ = 516.00, GOF = 1.025, *R*_int_ = 0.0646, *R*_1_ = 0.0526, w*R*_2_ = 0.0665, Flack parameter = 0.038(19).

### Neutrophil elastase inhibition assay

The ability to inhibit neutrophil elastase (NE) activity was evaluated by fluorimetry using a commercially available kit (ab118971, Abcam, Cambridge, UK). The evaluation was carried out in accordance with the manufacturer's instructions and the procedure. Briefly, test compounds at concentrations of 3.9, 15.6, 62.5, 250 and 1000 nM were placed in a 96-well plate. Enzyme control (assay buffer) was added to other wells instead of test compounds. NE and elastase substrate were then prepared according to the manufacturer's instructions. The NE solution was diluted as appropriate and added to the individual wells. The contents of the wells were thoroughly mixed and then incubated at 37 °C for 5 min. In the meantime, a reaction mixture was prepared by mixing the assay buffer with the NE substrate. After incubation, the reaction mixture was added to the wells and mixed thoroughly. Fluorescence measurements were started immediately with a microplate reader (SpectraMax i3X, Molecular Devices, San Jose, CA, USA) at excitation wavelength = 400 nm and emission wavelength = 505 nm. The measurements for RFU of fluorescence were performed in kinetic mode for 30 min at 37 °C. The ability of the analyzed samples to inhibit NE activity was calculated using equation shown below. The IC_50_ values shown in Tables 1 and 2 were estimated from% NE inhibition at various concentrations (3.9, 15.6, 62.5, 250 and 1000 nM) of the test compounds.%NE inhibition = (1 − (RFU of Test compound)/(RFU of Enzyme Control)) × 100%

### Docking simulation

Two independent structural models of human neutrophil elastase were generated using the AlphaFold Server (powered by AlphaFold3) and used as receptor structures for molecular docking. Molecular docking simulations of compounds (+)/(−)-2b, (+)/(−)-2d and Sivelestat (1) were performed using AutoDock Vina (version 1.5.7). The docking grid was defined to encompass the entire protein structure for each model. For Model 1, the grid center coordinates were set to *X* = 5.509, *Y* = 0.121, and *Z* = −1.988. For Model 2, the grid center coordinates were set to *X* = 5.382, *Y* = −2.695, and *Z* = 4.395. The grid box dimensions were set to 50 × 50 × 50 Å for both models. All other docking parameters were kept at their default values. Docking poses were initially ranked according to their predicted binding affinities. For each compound, the final docking pose was selected based on both the docking score and the presence of interactions with Ser202 and His70, residues previously reported to play important roles in inhibitor binding to human neutrophil elastase. Among the poses satisfying these criteria, the pose with the highest docking score was selected for subsequent interaction analysis. This selection criterion was applied consistently to the simulated compounds.

## Author contributions

H. Tabata conceived the study, synthesized the compounds, and analyzed the data. Y. Miyata performed biological evaluations. Y. Kusakabe conducted the computational study. H. Tabata carried out the single-crystal X-ray analysis. H. Takahashi and T. Oshitari provided valuable advice in advancing the research. H. Tabata and H. Natsugari supervised the project and wrote the manuscript. All authors contributed to reviewing and editing the manuscript.

## Conflicts of interest

The authors declare no competing financial interests.

## Supplementary Material

MD-017-D6MD00430J-s001

## Data Availability

The data supporting this article have been included as part of the supplementary information (SI), which includes synthesis and characterization of intermediate compounds, separation of enantiomers, stereochemical stability, graphs of assay data and NMR spectra. Supplementary information is available. See DOI: https://doi.org/10.1039/d6md00430j. CCDC 2506269 contains the supplementary crystallographic data for this paper.^40^
