# Supplementary material for: Development of atropisomeric 5N-sulfonyl-1,5-benzodiazepin-2-ones with high inhibitory activity against neutrophil elastase
Source: RSC Med Chem. 2026 Aug 7;17(9):4338–46. doi: 10.1039/d6md00430j (PMC13459609; doi:10.1039/d6md00430j)
Supplement: Supplementary file 1 [file MD-017-D6MD00430J-s001.pdf]

Supplementary Information (SI) for RSC Medicinal Chemistry.
This journal is © The Royal Society of Chemistry

Supporting Information

Development of Atropisomeric 5*N*-Sulfonyl-1,5-Benzodiazepin-2-ones with High Inhibitory Activity against Neutrophil Elastase

Hidetsugu Tabata,[†,*] Yoshiki Miyata,[†] Yoshio Kusakabe,[†] Hideyo Takahashi,[§] Hideaki Natsugari,[‡,*] and Tetsuta Oshitari[†]

[†]Faculty of Pharmaceutical Sciences, Teikyo University, 2-11-1 Kaga, Itabashi-ku, Tokyo 173-8605, Japan. [§]Faculty of Pharmaceutical Sciences, Tokyo University of Science, 6-3-1, Niijuku, Katsushika-ku, Tokyo, 125-8585, Japan. [‡]Faculty of Pharmacy, Niigata University of Pharmacy and Medical and Life Sciences, 265-1 Higashijima, Akiha-ku, Niigata 956-8603, Japan.

Corresponding Author: Hidetsugu Tabata (tabata.hidetsugu.ad@teikyo-u.ac.jp)
Hideaki Natsugari (natsu@affinity-science.com)

Contents

1. Experimental procedure and characterization of intermediate compounds S2
2. Separation of enantiomers using chiral column of **2b** and **2d** S5
3. Stereochemical stability of the enantiomers of **2b** and **2d** (conversion profile and determination of $\Delta G^{\ddagger}$ value) S6
4. Graphs of neutrophil elastase inhibition assay S7
5. Trypsin inhibitory assay of racemic **2b** and **2d** and their enantiomers S8
6. Decomposition of the calculated docking energies and docking models S9
7. NMR spectra S11
8. HRMS data S32

1. Experimental procedure and characterization of intermediate compounds

Methyl 2,3,4,5-tetrahydro-2-oxo-1*H*-1,5-benzodiazepine-1-acetate (6c)

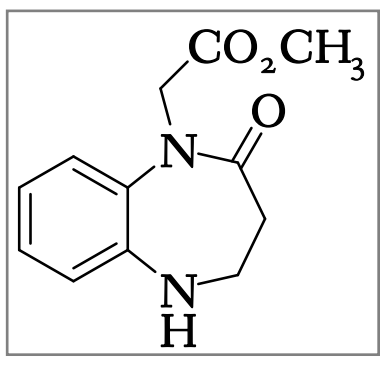

To a stirred solution of **5a** (121 mg, 0.75 mmol) in DMF (7.5 mL) was added sodium hydride (60% in oil) (45 mg, 1.12 mmol) at 0 °C under argon. The mixture was stirred for 30 min at 25 °C, cooled to 0 °C, and treated with methyl bromoacetate (0.13 mL, 1.34 mmol). After being stirred for 2 h at 25 °C, the mixture was treated with H_2O and extracted with ethyl acetate. The extract was washed with brine, dried, and evaporated. The residue was purified by column chromatography (silica gel, ethyl acetate/n-hexane = 1/2) to afford **6c** as an Amorphous solids (100 mg, 57%): ^{1}H NMR (600 MHz, $CDCl_3$), δ 7.10–7.12 (m, 2H, ArH), 7.03 (dt, *J* = 1.3, 7.5 Hz, 1H, ArH), 6.97 (dd, *J* = 1.3, 7.5 Hz, 1H, ArH), 4.62 (s, 2H, NCH_2), 3.78 (s, 3H, OCH_3), 3.77–3.80 (m, 2H, CH_2), 2.61 (t, *J* = 6.6 Hz, 2H, CH_2); ^{13}C{^{1}H} NMR (150 MHz, $CDCl_3$) δ 172.1, 170.0, 135.4, 127.0, 123.4, 122.9, 122.7, 122.3, 52.4, 50.7, 50.2, 33.2; IR (ATR) 2934, 1649 cm^{-1}; HRMS (ESI) *m/z*: [M+H]$^+$ calcd for: $C_{12}H_{15}N_2O_3$ 235.1077; found 235.1078.

Methyl 6-methyl-2,3,4,5-tetrahydro-2-oxo-1*H*-1,5-benzodiazepine-1-acetate (6d)

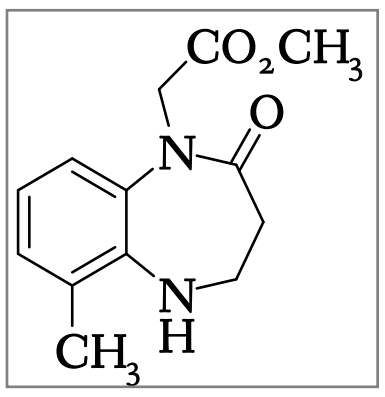

Compound **6d** was prepared from **5b** (53 mg 0.30 mmol) according to a similar procedure as described for the preparation of **6c** from **5a**. Amorphous solids (64 mg, 86%): mp 118–120 °C: ^{1}H NMR (600 MHz, $CDCl_3$), δ 7.03 (dd, *J* = 1.2, 7.4 Hz, 1H, ArH), 6.98 (dd, *J* = 1.2, 7.9 Hz, 1H, ArH), 6.92 (dd, *J* = 7.4, 7.9 Hz, 1H, ArH), 4.44 (s, 2H, NCH_2), 3.77 (s, 3H, OCH_3), 3.76 (t, *J* = 6.7 Hz, 2H, CH_2), 2.56 (t, *J* = 6.7 Hz, 2H, CH_2), 2.30 (s, 3H, CH_3); ^{13}C{^{1}H} NMR (150 MHz, $CDCl_3$), δ 172.5, 169.9, 138.7, 136.6, 130.2, 128.4, 122.5, 120.8, 52.3, 50.7, 49.6, 33.7, 17.7; IR (ATR) 3376, 1743, 1662, 1216 cm^{-1}; HRMS (ESI) *m/z*: [M+H]$^+$ calcd for: $C_{13}H_{17}N_2O_3$ 249.1234; found 249.1235.

Benzyl 6-methyl-2,3,4,5-tetrahydro-2-oxo-1*H*-1,5-benzodiazepine-1-acetate (6e)

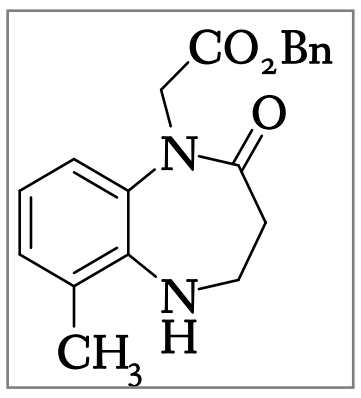

Compound **6e** was prepared from **5b** (226 mg 1.28 mmol) and benzyl bromoacetate (529 mg, 2.31 mmol) according to a similar procedure as described for the preparation of **6c** from **5a**. Colorless oil (253 mg, 61%): ^{1}H NMR (600 MHz, $CDCl_3$), δ 7.30–7.38 (m, 5H, ArH), 7.03 (dd, *J* = 0.9, 7.6 Hz, 1H, ArH), 6.96 (dd, *J* = 0.9, 7.7 Hz, 1H, ArH), 6.90 (dd, *J* = 7.6, 7.7 Hz, 1H, ArH), 5.20 (s, 2H, NCH_2), 4.50 (s, 2H, CH_2Ar), 3.76 (t, *J* = 6.7 Hz, 2H, CH_2), 2.56 (t, *J* = 6.7 Hz, 2H, CH_2), 2.29 (s, 3H, CH_3); ^{13}C{^{1}H} NMR (150 MHz, $CDCl_3$) δ172.5, 169.3, 138.8, 136.5, 135.4, 130.2, 128.5, 128.3, 128.2, 122.5, 120.7, 67.0, 50.8, 49.6, 33.7, 17.7; IR (ATR) 3384, 1741, 1660, 1191 cm^{-1}; HRMS (ESI) *m/z*: [M+H]$^+$ calcd for: $C_{19}H_{21}N_2O_2$ 325.1547; found 325.1548.

N-(2-Hydroxyethyl)-*N*,3-dimethyl-2-nitrobenzamide (11)

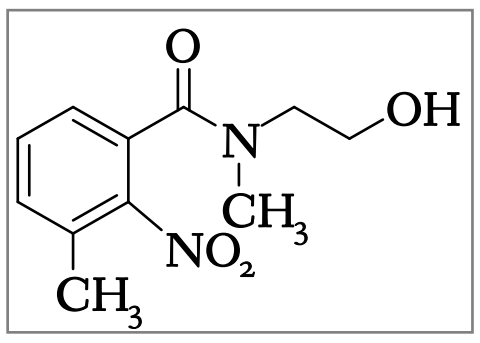

To a solution of 3-methyl-2-nitrobenzoic acid (**10**, commercial reagents) (1.45 g, 8.0 mmol) in THF (32 mL) was added dropwise oxalyl chloride (1.0 mL, 12.0 mmol), followed by a catalytic amount of DMF (one drop). After the mixture was stirred at 25 °C for 1 h, the solvent was then removed under reduced pressure to afford the corresponding crude acid chloride, which was used directly for the next step without any purification. To a solution of acid chloride in THF (32 mL) were added *N*-methylaminoethanol (1.6 mL, 20.0 mmol) and triethylamine (2.8 mL, 20.0 mmol). After stirring at 25 °C for 20 h, the solvent was removed under reduced pressure. The residue was diluted with ethyl acetate, then washed successively with H_2O, 1N aq. HCl, saturated aq. $NaHCO_3$, H_2O and brine. The organic layer was dried over $MgSO_4$, concentrated, and the residue was purified by column chromatography (silica gel, methanol/CH_2Cl_2 = 1/40) to afford **11** of E/Z mixture (1.68 g, 88%) as pale-yellow oil: ^1H NMR (600 MHz, $CDCl_3$) (*trans*-form) δ 7.47 (t, *J* = 7.5 Hz, 1H, ArH), 7.36 (t, *J* = 7.5 Hz, 1H, ArH), 7.24 (t, *J* = 7.5 Hz, 1H, ArH), 3.91 (t, *J* = 5.5 Hz, 2H, CH_2), 3.69 (t, *J* = 5.5 Hz, 2H, CH_2), 3.00 (s, 3H, NCH_3), 2.48 (s, 3H, CH_3), (*cis* -form) δ 7.43 (t, *J* = 7.5 Hz, 0.34H, ArH), 7.34 (t, *J* = 7.5 Hz, 0.34H, ArH), 7.27 (t, *J* = 7.5 Hz, 0.34H, ArH), 3.75 (t, *J* = 5.5 Hz, 0.76H, CH_2), 3.44 (t, *J* = 5.5 Hz, 0.76H, CH_2), 3.12 (s, 1.1H, NCH_3), 2.44 (s, 1.1H, CH_3); $^{13}C\{^1H\}$ NMR (150 MHz, $CDCl_3$) (*trans*-form), δ 168.7, 147.6, 132.7, 132.6, 132.5, 131.5, 125.1, 60.7, 50.7, 38.4, 18.9; (*cis*-form) (only the distinguishable peaks are described), δ 167.9, 148.1, 132.4, 132.1, 131.8, 131.1, 125.7, 59.5, 53.0, 32.8, 18.7; IR (ATR) 3399, 1621, 1525 cm^{-1}; HRMS (ESI) *m/z*: $[M+H]^+$ calcd for : $C_{11}H_{15}N_2O_4$ 239.1026; found 239.1027.

2-Amino-*N*-(2-hydroxyethyl)-*N*,3-dimethylbenzamide (12)

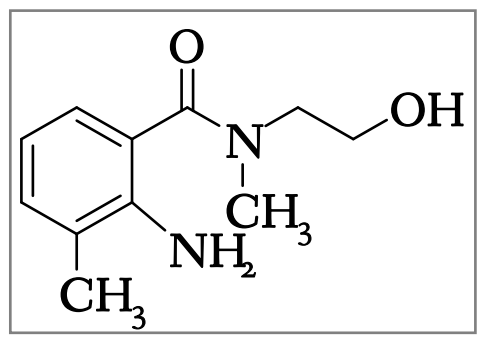

To a solution of **11** (551 mg, 2.31 mmol) in EtOH (23 mL) was added 10% palladium on carbon (35 mg). After the mixture was stirred at 25°C for 7 h under a hydrogen atmosphere, the mixture was filtered. The filtrate was concentrated under vacuum, and the residue was purified by column chromatography. (silica gel, methanol/CH_2Cl_2 = 1/20) to afford **12** (442 g, 92%) as pale-yellow oil. ^1H NMR (600 MHz, THF-d_8), δ 6.94 (d, *J* = 7.5 Hz, 1H, ArH), 6.93 (d, *J* = 8.2 Hz, 1H, ArH), 6.49 (dd, *J* = 7.5, 8.2 Hz, 1H, ArH), 4.57 (exch br s, 2H, NH_2), 3.65 (br, 2H, CH_2), 3.46 (br, 2H, CH_2), 2.99 (s, 3H, NCH_3), 2.11 (s, 3H, CH_3); $^{13}C\{^1H\}$ NMR (150 MHz, THF-d_8) δ 172.0, 145.1, 131.4, 126.5, 123.4, 121.4, 116.4, 67.8, 60.5, 25.7, 17.7; IR (ATR) 3375, 1605 cm^{-1}; HRMS (ESI) *m/z*: $[M+H]^+$ calcd for : $C_{11}H_{17}N_2O_2$ 209.1285; found 209.1289.

4-(*N*-(2-(*N'*-(2-Hydroxyethyl)-*N'*-methylcarbamoyl)-6-methylphenyl)sulfamoyl)phenyl pivalate (13)

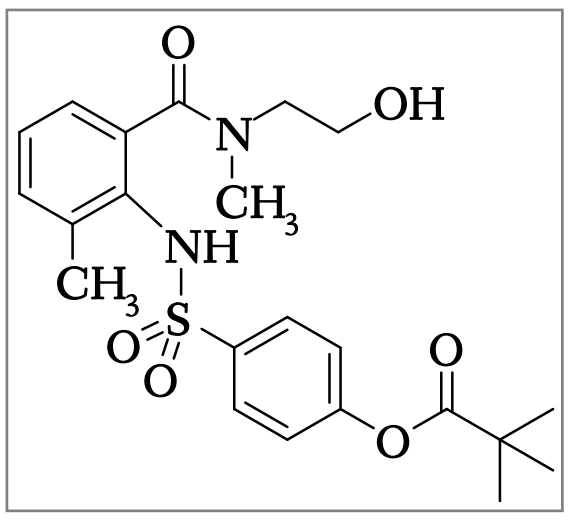

To a stirred solution of **12** (149 mg, 0.71 mmol) in CH_2Cl_2 (4.8 mL) under argon were added pyridine (0.26 mL, 3.2 mmol) and *p*-pivaloyloxybenzenesulfonyl chloride (**9**) (217 mg, 0.79 mmol). After the mixture was stirred at 25 °C for 20 h, 1N aq. HCl was added, and the mixture was extracted with CH_2Cl_2. The extract was washed with brine and dried over $MgSO_4$. The solvent was evaporated, and the residue was purified by column chromatography (silica gel, methanol/CH_2Cl_2 = 1/40) to afford **13** (215 mg, 67%) as white solid. mp 125–127 °C: ^{1}H NMR (600 MHz, $CDCl_3$), δ 7.89 (exch br s, 1H, NH), 7.83 (d, *J* = 8.9 Hz, 2H, ArH), 7.29 (d, *J* = 6.8 Hz, 1H, ArH), 7.18 (dd, *J* = 6.8, 7.5 Hz, 1H, ArH), 7.16 (d, *J* = 8.9 Hz, 2H, ArH), 7.08 (d, *J* = 7.5 Hz, 1H, ArH), 3.83 (br, 2H, CH_2), 3.52 (t, *J* = 5.5 Hz, 2H, CH_2), 2.96 (exch br s, 1H, OH), 2.88 (s, 3H, NCH_3), 2.33 (s, 3H, CH_3), 1.36 (s, 9H, $C(CH_3)_3$); $^{13}C\{^1H\}$ NMR (150 MHz, $CDCl_3$) δ 176.6, 170.8, 154.6, 139.8, 137.1, 133.4, 133.1, 132.4, 129.1, 126.7, 125.3, 122.2, 60.6, 51.0, 39.4, 39.2, 27.0, 19.1; IR (ATR) 2974, 1608, 1108 cm^{-1}; HRMS (ESI) *m/z*: $[M+H]^+$ calcd for : $C_{22}H_{29}N_2O_6S_1$ 449.1741; found 449.1743.

4-(*N*-(2-(*N'*-(2-((Methylsulfonyl)oxy)ethyl)-*N'*-methylcarbamoyl)-6-mthylphenyl)sulfamoyl) phenyl pivalate (14)

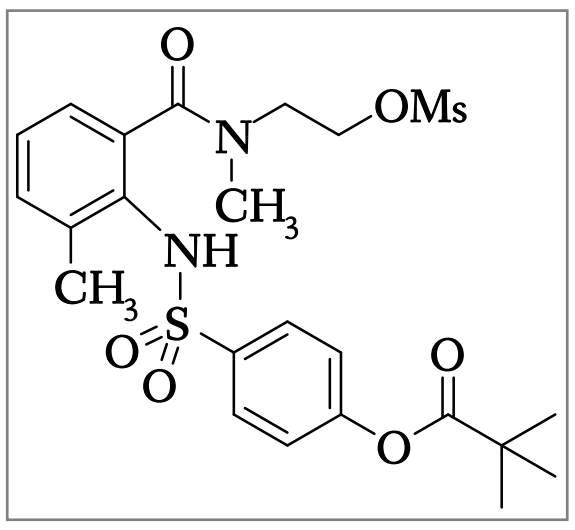

To a stirred solution of **13** (106 mg, 0.23 mmol) in CH_2Cl_2 (2.4 mL) under argon were added triethylamine (98 mL, 0.71 mmol) and methanesulfonyl chloride (37 mL, 0.47 mmol) at 0 °C. After the mixture was stirred at 0 °C for 4 h, diluted with CH_2Cl_2, and the mixture was washed with saturated aq. $NaHCO_3$ and brine. The organic layer was dried over $MgSO_4$. and the solvent was evaporated. The residue was purified by column chromatography (silica gel, ethyl acetate/n-hexane = 1/3) to afford **14** (64 mg, 51%) as white solid. mp 79–82 °C: ^{1}H NMR (600 MHz, $CDCl_3$), δ 7.84 (d, *J* = 8.9 Hz, 2H, ArH), 7.32 (dd, *J* = 7.5, 7.5 Hz, 1H, ArH), 7.24 (dd, *J* = 1.3, 7.5 Hz, 1H, ArH), 7.20 (d, *J* = 8.9 Hz, 2H, ArH), 7.19-7.21 (m, 1H, ArH), 3.90-3.96 (m, 2H, CH_2), 3.85 (s, 3H, NCH_3), 3.74-3.83 (m, 2H, CH_2), 3.15 (s, 3H, SCH_3), 1.63 (s, 3H, CH_3), 1.36 (s, 9H, $C(CH_3)_3$); $^{13}C\{^1H\}$ NMR (150 MHz, $CDCl_3$) δ 176.1, 169.3, 155.7, 142.5, 137.9, 135.0, 132.6, 130.8, 130.7, 129.2, 126.1, 122.3, 49.8, 43.5, 40.6, 39.4, 39.2, 26.9, 18.1; IR (ATR) 2923, 1633, 1371, 1096 cm^{-1}; HRMS (APCI) *m/z*: $[M+H]^+$ calcd for : $C_{23}H_{31}N_2O_8S_2$ 527.1516; found 527.1522.

2. Separation of enantiomers using chiral column of 2b and 2d

Atropisomers of **2b**: CHIRAL ART Amylose-SA (0.46 cmϕx 25 cm); eluent, hexane:EtOH (1:1); flow rate, 0.3 mL/min; temperature, 25 °C; detection, 254 nm. Former peak: retention time = 23.3 min; [α]$_{D}^{20}$ −146.6 (>99.9%ee, *c* 0.11, MeOH). Latter peak: retention time = 32.6 min; [α]$_{D}^{20}$ +147.9 (>99.9%ee, *c* 0.11, MeOH).

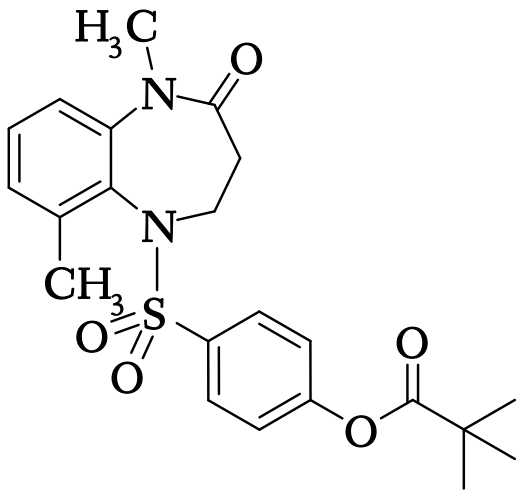

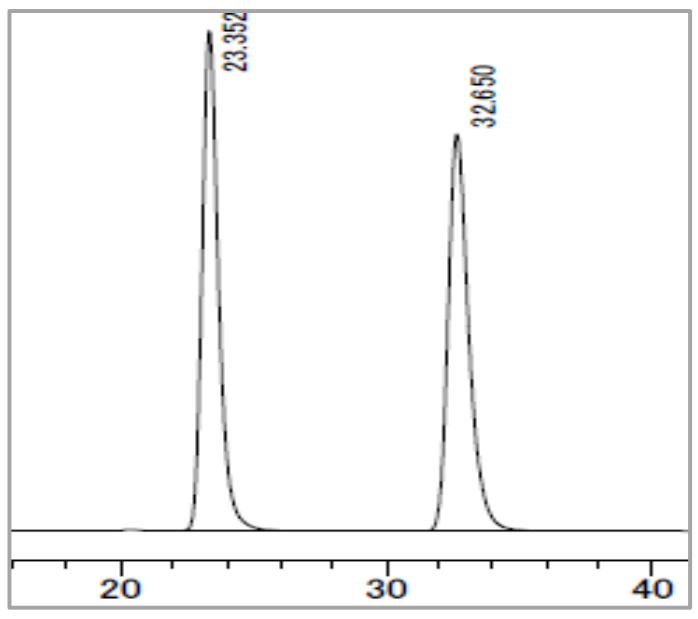

Atropisomers of **2d**: CHIRAL ART Amylose-SA (0.46 cmϕx 25 cm); eluent, hexane:EtOH (4:1); flow rate, 0.18 mL/min; temperature, 25 °C; detection, 254 nm. Former peak: retention time = 17.4 min; [α]$_{D}^{20}$ −208.9 (>99.9%ee, *c* 0.11, MeOH). Latter peak: retention time = 28.2 min; [α]$_{D}^{20}$ +213.7 (>99.9%ee, *c* 0.11, MeOH).

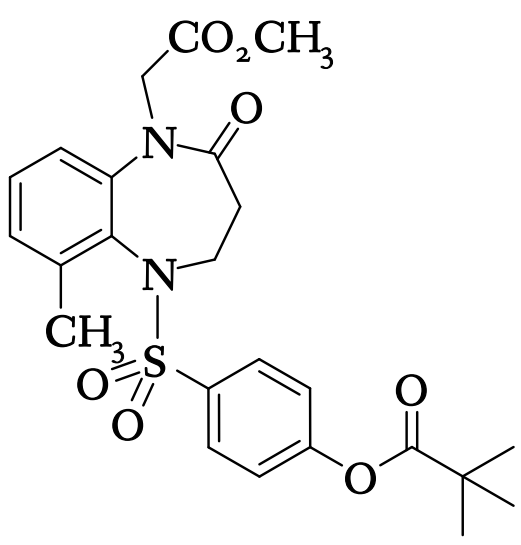

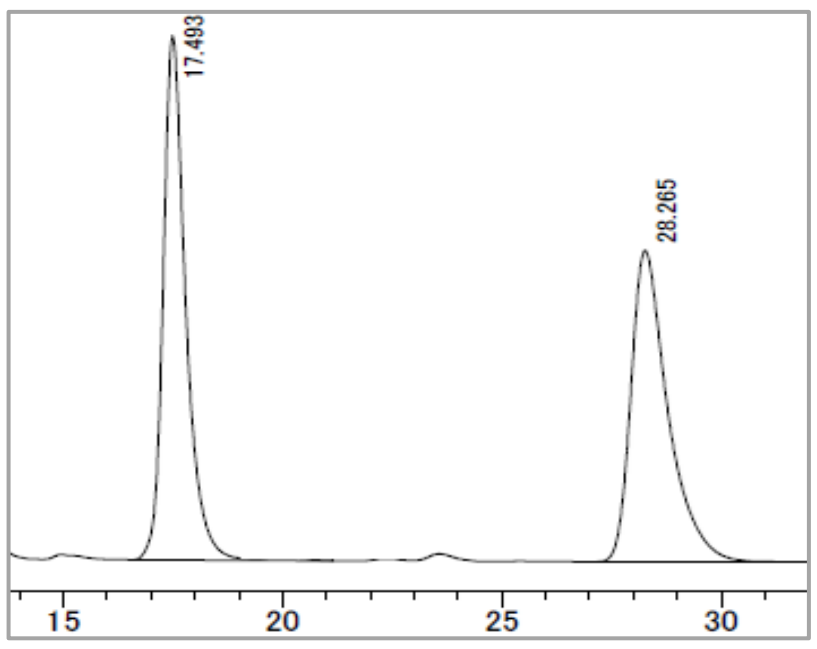

3. Stereochemical stability of the enantiomers of 2b and 2d (conversion profile and determination of $\Delta G^{\ddagger}$ value)

To examine thermal stability of enantiomers, the time-dependent conversion rate (%) was estimated from chiral HPLC analysis of a solution of the enantiomer in toluene or DMF after allowing to stand at designated temperatures. The $\Delta G^{\ddagger}$ value was determined according to a calculation method reported in a literature (ref. 20).

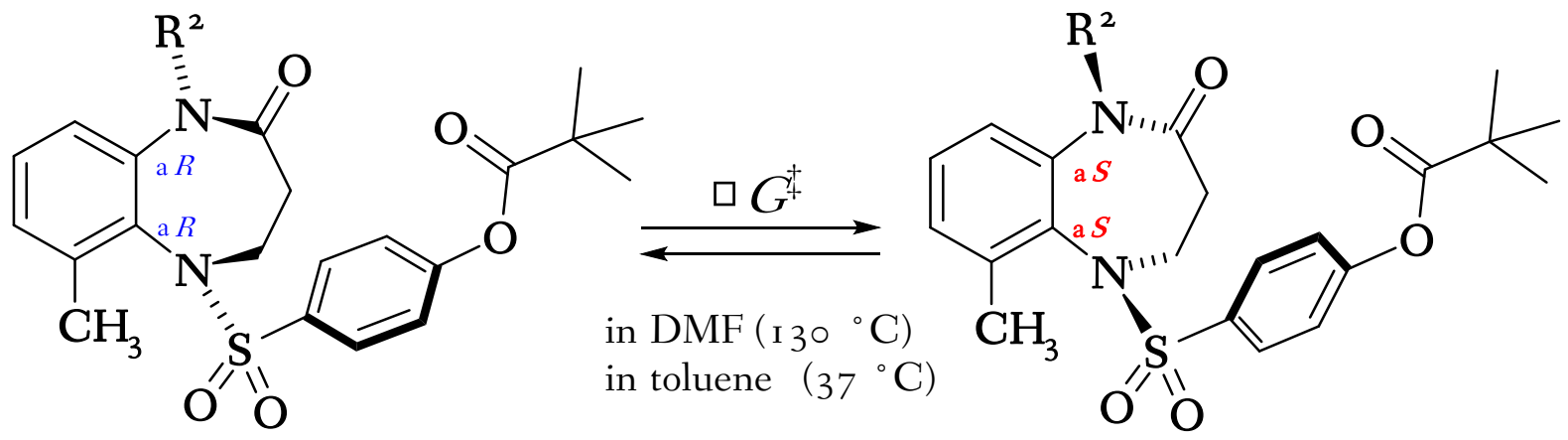

Stereochemical stability of the enantiomers of **2b** ($R^2 = CH_3$)

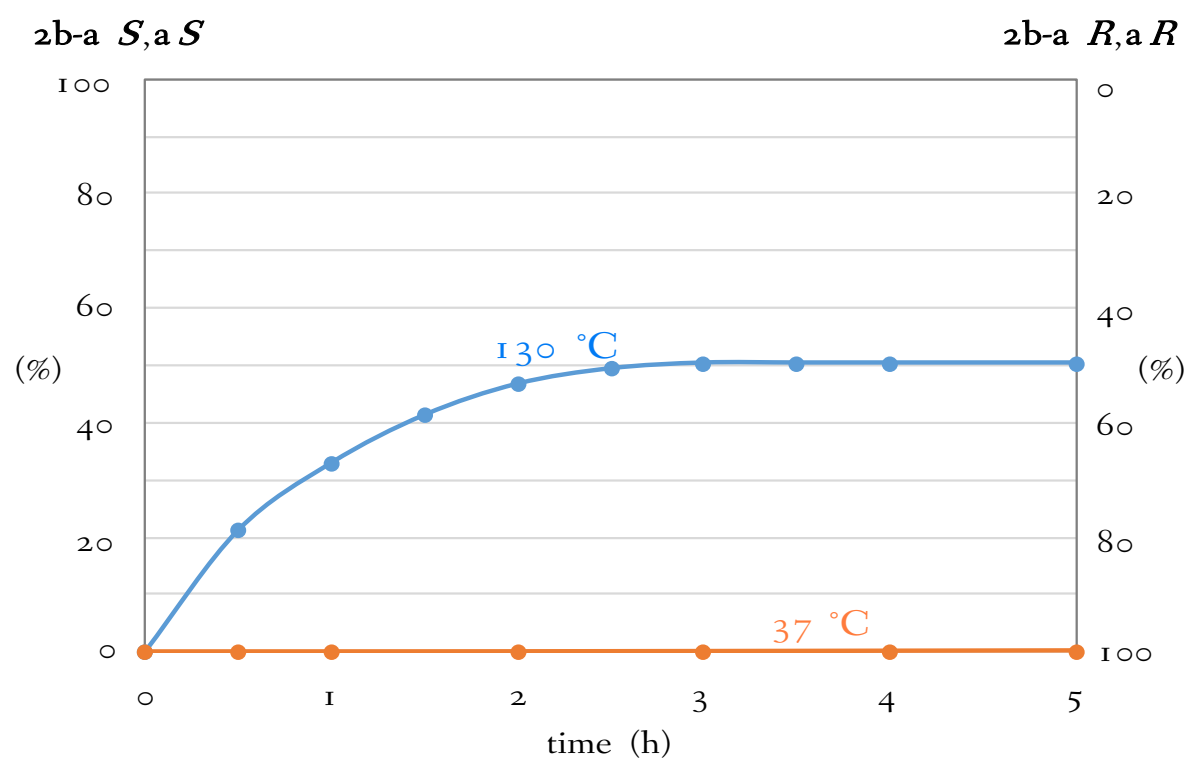

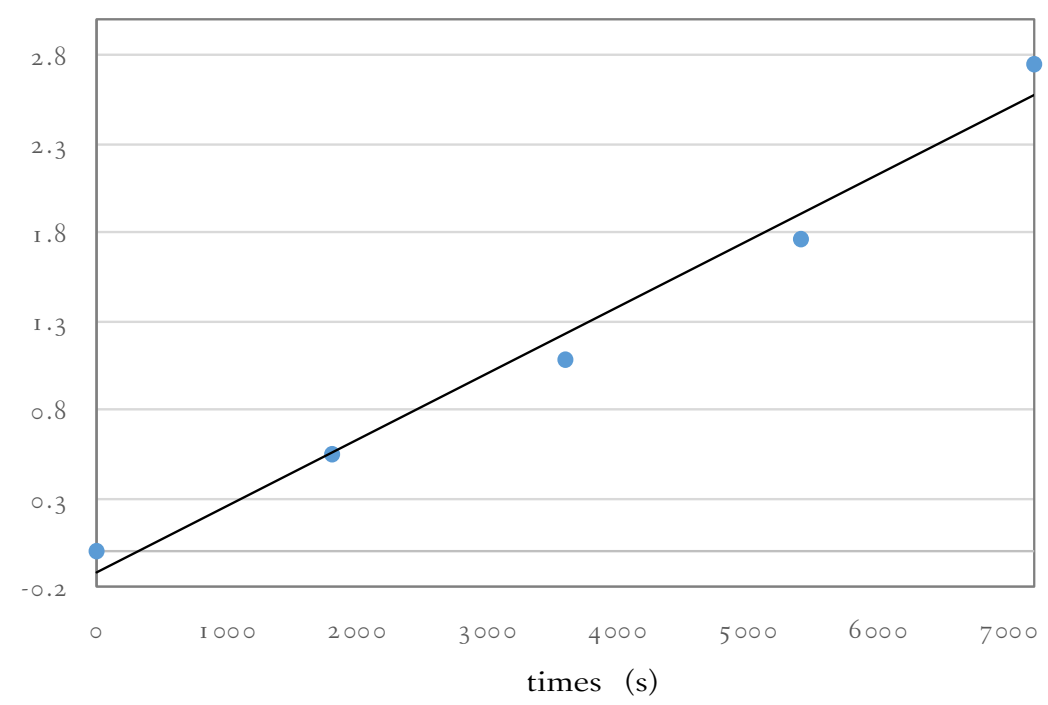

T =403
k = 1/2 slope = 1.864 x 10^{-4}
K = kh/kT = 2.220 x 10^{-17}
$DG^{\ddagger}$ = -RTlnK = 128.4 kJ/mol

Stereochemical stability of the enantiomers of **2d** ($R^2 = CH_2CO_2CH_3$)

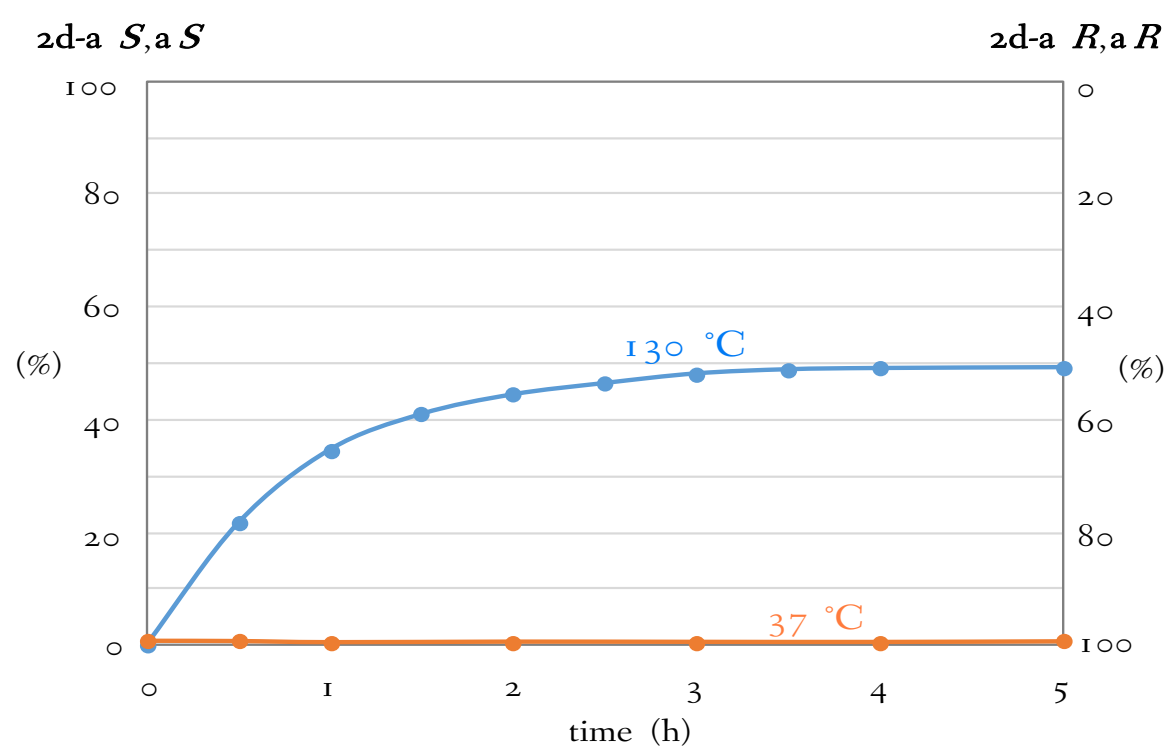

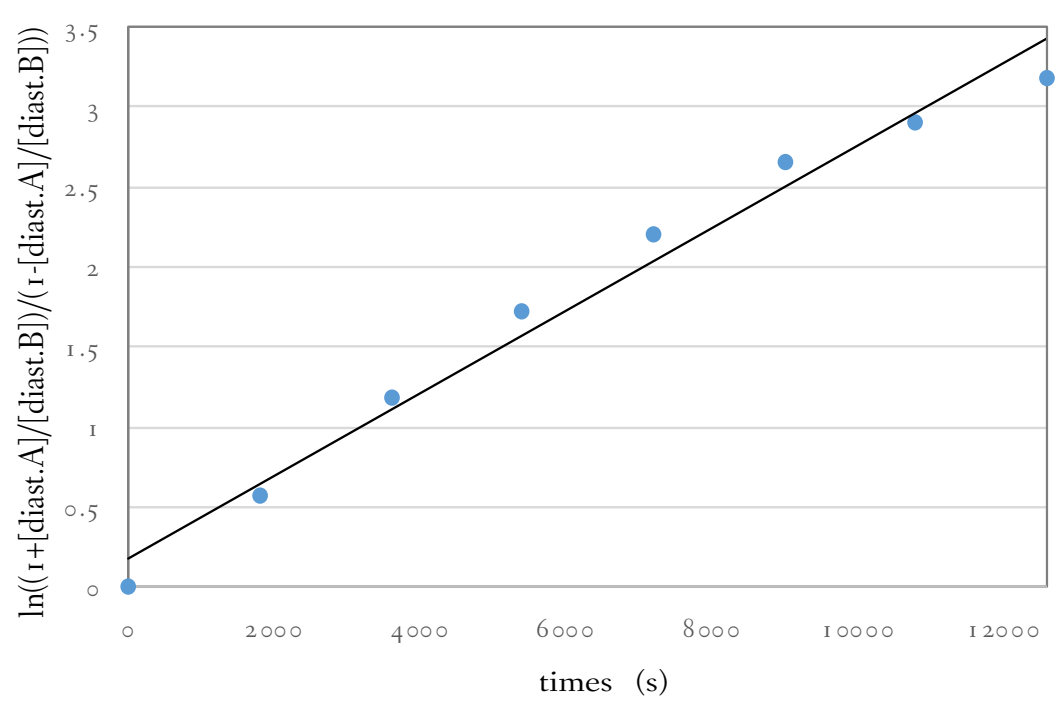

T =403
k = 1/2 slope = 1.286 x 10^{-4}
K = kh/kT = 1.532 x 10^{-17}
$DG^{\ddagger}$ = -RTlnK = 129.7 kJ/mol

4. Graphs of Neutrophil Elastase Inhibition Assay

The ability to inhibit neutrophil elastase (NE) activity was evaluated by fluorimetry using a commercially available kit (ab118971, Abcam, Cambridge, UK). The evaluation was carried out in accordance with the manufacturer's instructions and the procedure. Briefly, test compounds at concentrations of 3.9, 15.6, 62.5, 250 and 1000 nM were placed in a 96-well plate. Enzyme control (assay buffer) was added to other wells instead of test compounds. NE and elastase substrate were then prepared according to the manufacturer's instructions. The NE solution was diluted as appropriate and added to the individual wells. The contents of the wells were thoroughly mixed and then incubated at 37℃ for 5 min. In the meantime, a reaction mixture was prepared by mixing the assay buffer with the NE substrate. After incubation, the reaction mixture was added to the wells and mixed thoroughly. Fluorescence measurements were started immediately with a microplate reader (SpectraMax i3X, Molecular Devices, San Jose, CA, USA) at excitation wavelength = 400 nm and emission wavelength =505 nm. The measurements for RFU of fluorescence were performed in kinetic mode for 30 min at 37℃. The ability of the analyzed samples to inhibit NE activity was calculated using Equation shown below. The IC_{50} values shown in Table 1 and 2 were estimated from % NE inhibition at various concentrations (3.9, 15.6, 62.5, 250 and 1000 nM) of the test compounds.

% NE inhibition = (1 – (RFU of Test compound) /(RFU of Enzyme Control)) x 100%

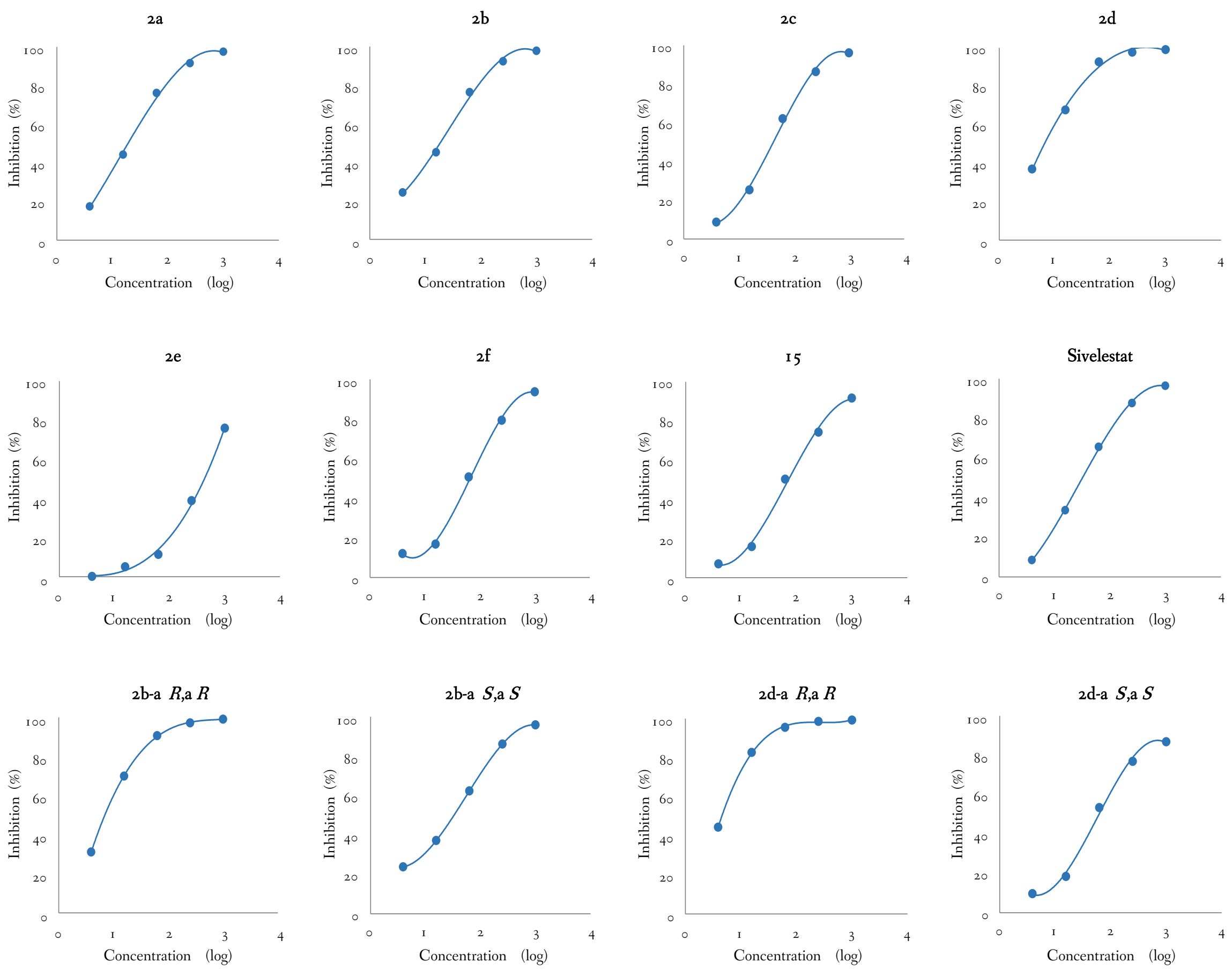

5. Trypsin inhibitory assay of racemic 2b and 2d and their enantiomers

To evaluate the selective NE inhibition of test compounds, trypsin activity was also evaluated by fluorimetry using a commercially available kit (DTRI-100, BioAssay system, Hayward, UK). The trypsin solution was diluted in 1mM HCl, and then prepared as appropriate using assay buffer. The evaluation was carried out in accordance with the manufacturer's instructions and the procedure. Briefly, trypsin solution (1 unit/well) were added in a 96-well plate. Test compounds at concentrations of 3.9, 15.6, 62.5, 250 and 1000 nM were then placed in a 96-well plate. Enzyme control (Control) or assay buffer (Blank) was added to other wells instead of test compounds (Compound). The contents of the wells were mixed and then incubated at room temperature for 15 min. In the meantime, a reaction mixture was prepared by mixing the assay buffer with the trypsin substrate. After incubation, the reaction mixture was added to the wells and mixed and then incubated at room temperature for 30 min. Fluorescence (F) measurements were started immediately with a microplate reader (SpectraMax i3X, Molecular Devices, San Jose, CA) at excitation wavelength = 485 nm and emission wavelength =530 nm. The trypsin enzyme activity in each well was calculated using Equation shown below.

Enzyme activity = $(F_{Compound} - F_{Blank}) / (F_{Control} - F_{Blank}) \times 100\%$

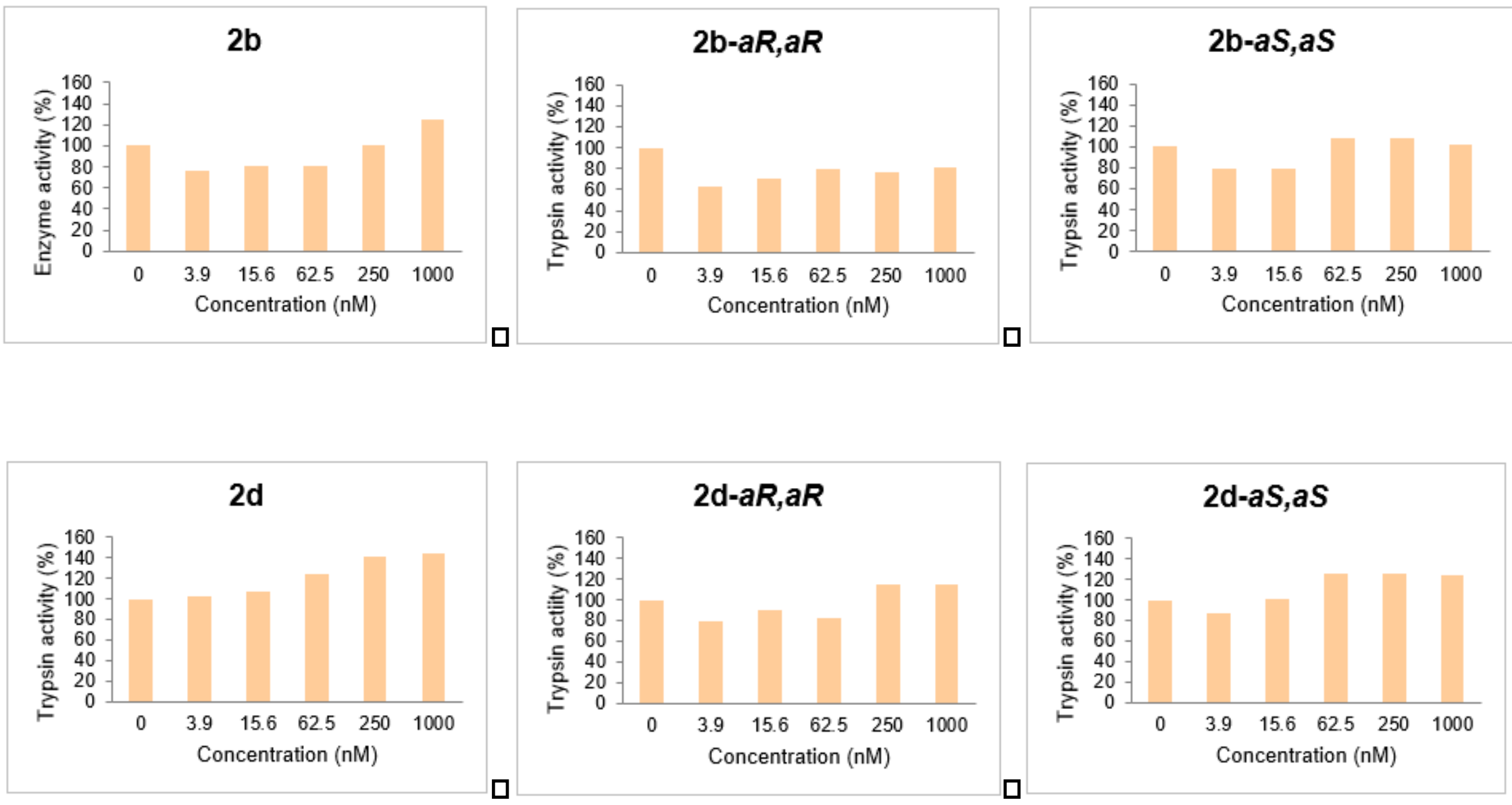

6. Docking simulation

Two independent structural models of human neutrophil elastase were generated using the AlphaFold Server (powered by AlphaFold3) and used as receptor structures for molecular docking. Molecular docking simulations of compounds (+) / (–)-**2b**, (+) / (–)-**2d** and Sivelestat (**1**) were performed using AutoDock Vina (version 1.5.7). The docking grid was defined to encompass the entire protein structure for each model. For Model 1, the grid center coordinates were set to X = 5.509, Y = 0.121, and Z = –1.988. For Model 2, the grid center coordinates were set to X = 5.382, Y = –2.695, and Z = 4.395. The grid box dimensions were set to 50 × 50 × 50 Å for both models. All other docking parameters were kept at their default values. Docking poses were initially ranked according to their predicted binding affinities. For each compound, the final docking pose was selected based on both the docking score and the presence of interactions with Ser202 and His70, residues previously reported to play important roles in inhibitor binding to human neutrophil elastase. Among the poses satisfying these criteria, the pose with the highest docking score was selected for subsequent interaction analysis. This selection criterion was applied consistently to the simulated compounds.

1) Decomposition of docking energies calculated by AutoDock Vina

**(+)-2b**	Kcal/mol	**(–)-2b**	Kcal/mol
Autodock Score:	–6.549	Autodock Score:	–6.325
Intermolecular Energy:	–8.382	Intermolecular Energy:	–9.184
Intramolecular Energy:	–1.129	Intramolecular Energy:	–0.139
Unbound Energy:	–1.047	Unbound Energy:	–1.149

**(+)-2d**	Kcal/mol	**(–)-2d**	Kcal/mol
Autodock Score:	–6.857	Autodock Score:	–6.260
Intermolecular Energy:	–9.522	Intermolecular Energy:	–9.301
Intramolecular Energy:	–1.804	Intramolecular Energy:	–0.720
Unbound Energy:	–1.262	Unbound Energy:	–0.833

**Sivelestat**	Kcal/mol
Autodock Score:	–6.260
Intermolecular Energy:	–9.768
Intramolecular Energy:	–1.585
Unbound Energy:	–1.617

2) Ligand-interacting amino acid residues in the docking models

(+)- **2b**

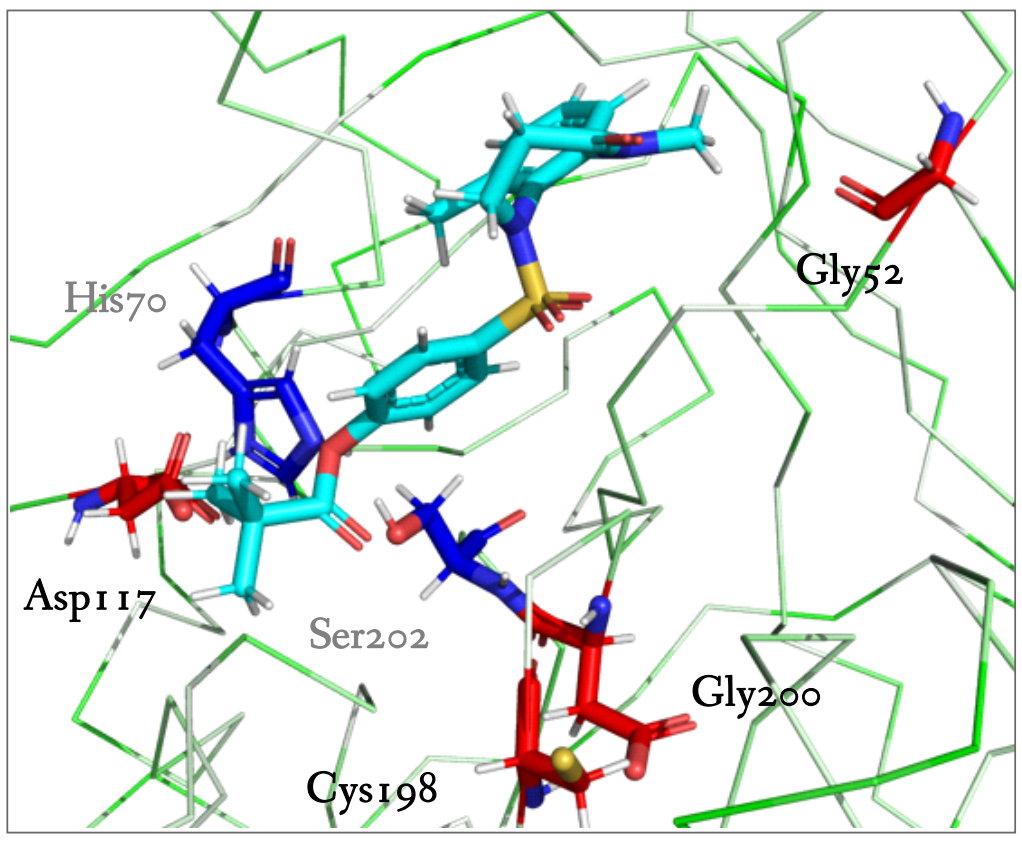

(–)- **2b**

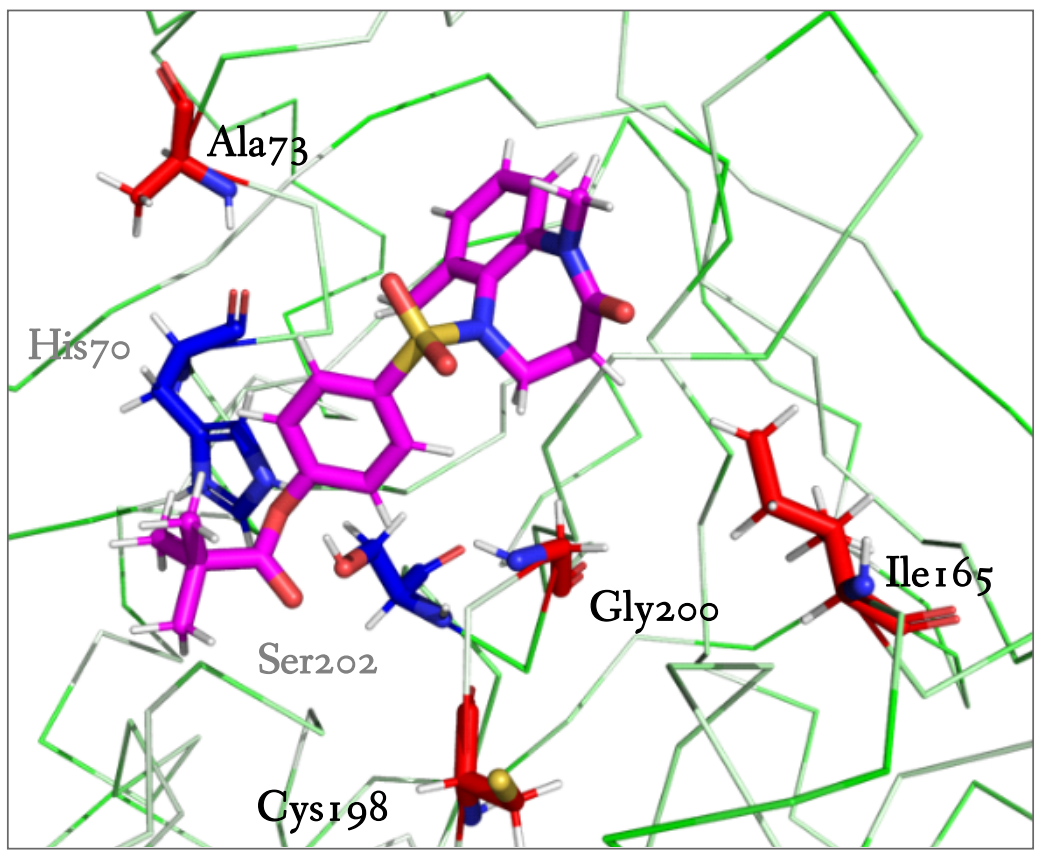

(+)- **2d**

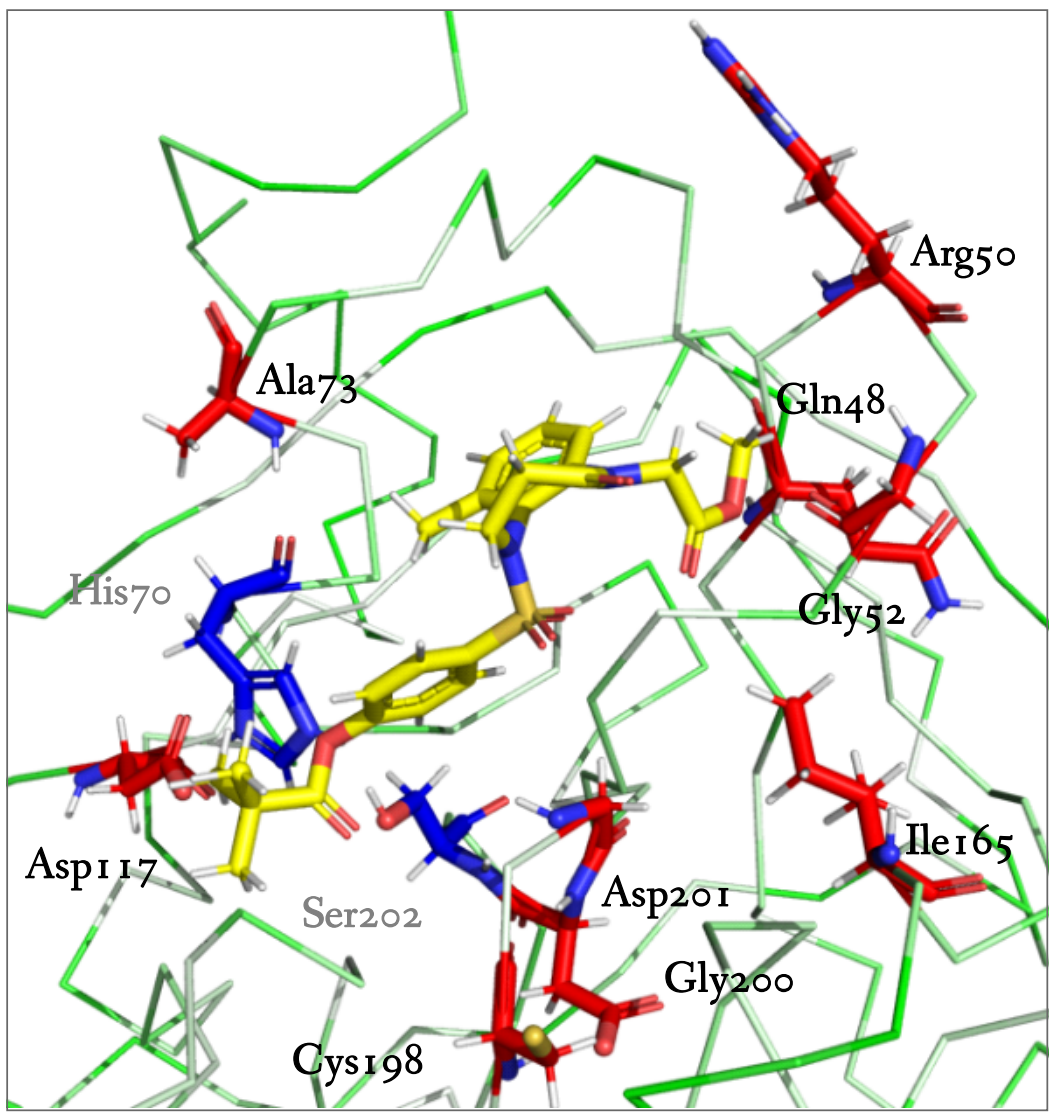

(–)- **2d**

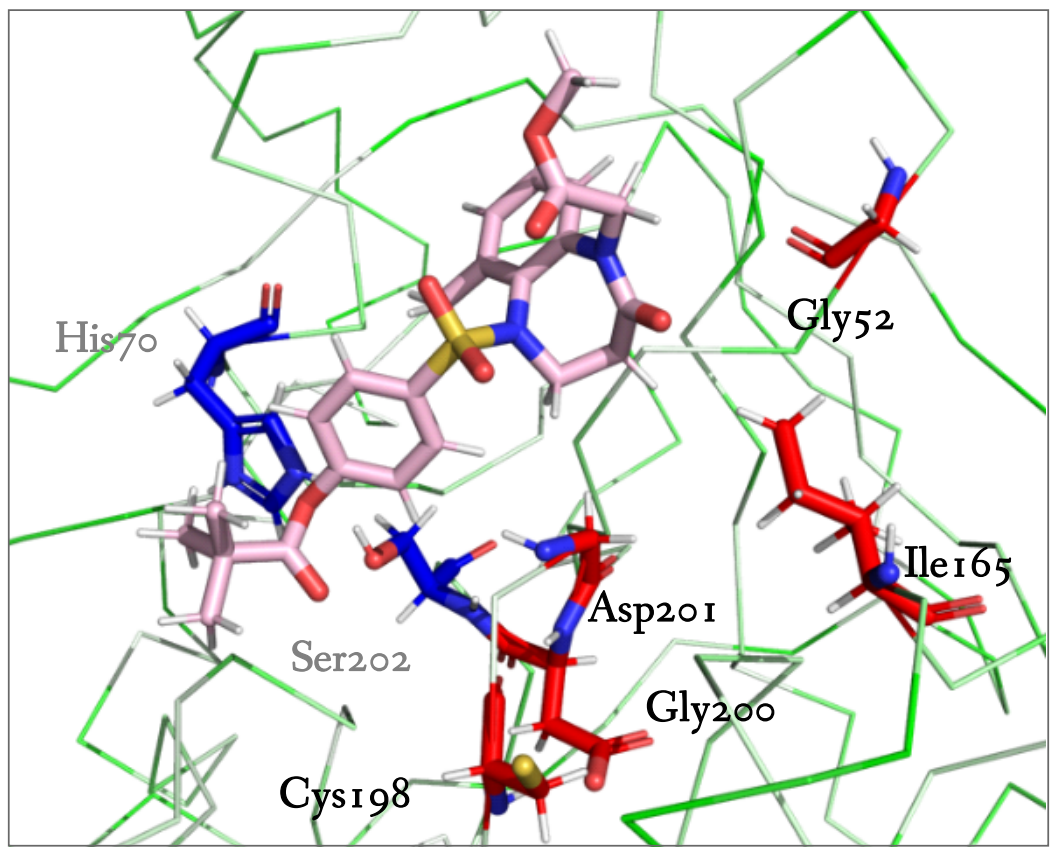

Sivelestat

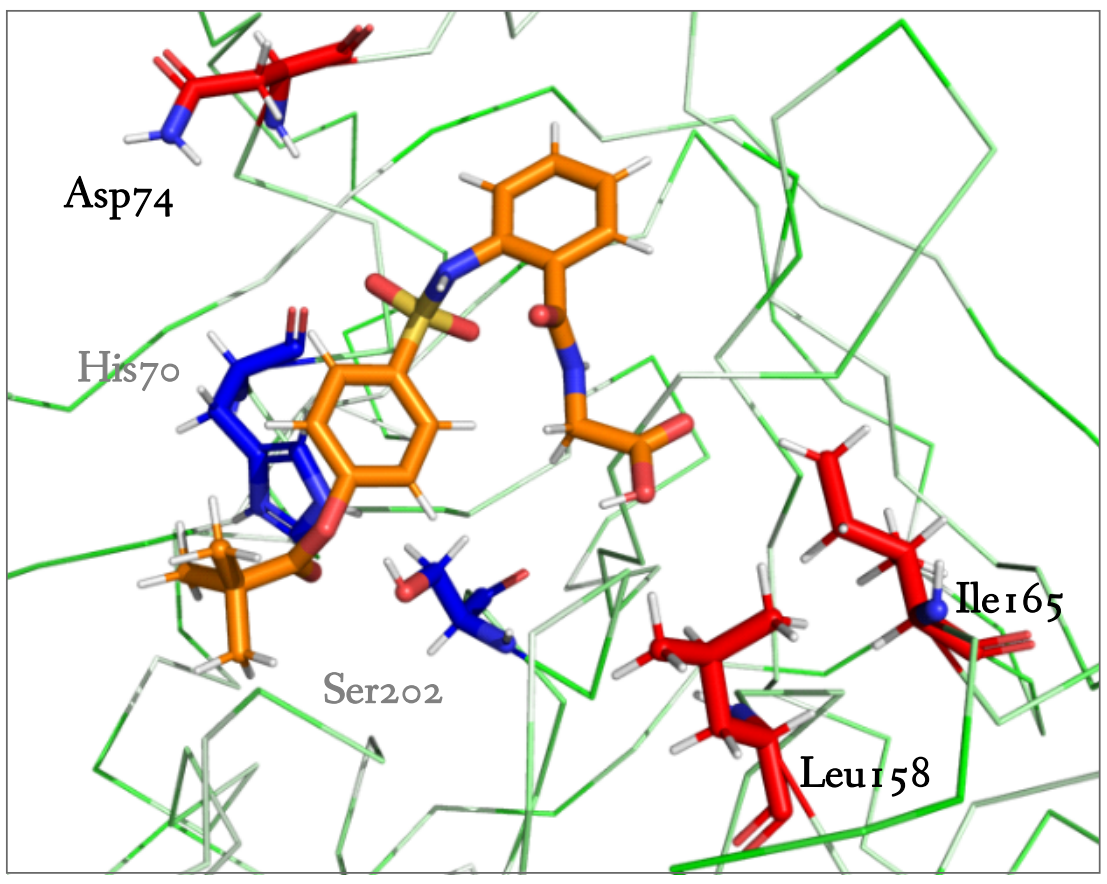

7. NMR spectra

^{1}H-NMR ($CDCl_3$)

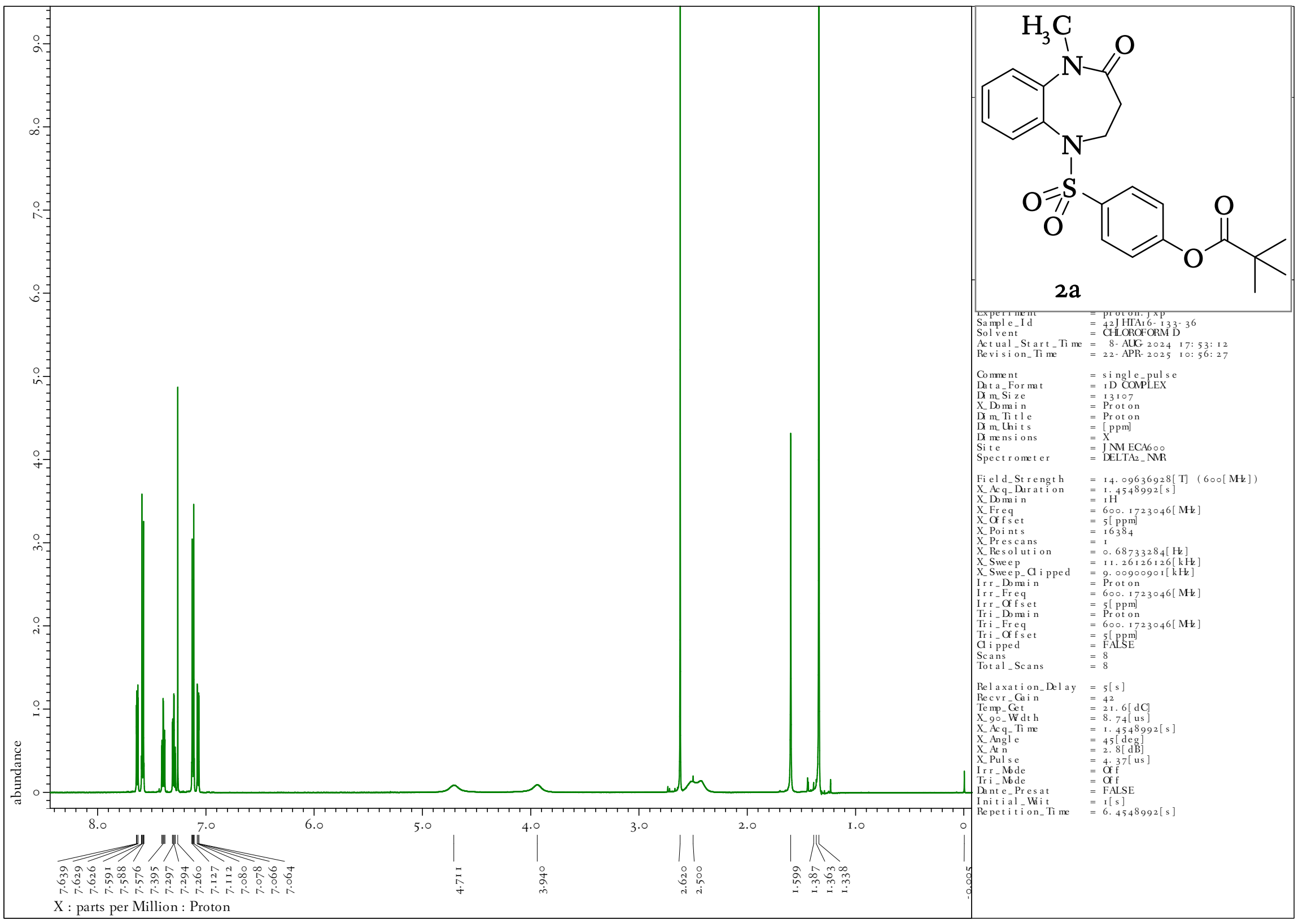

^{13}C-NMR ($CDCl_3$)

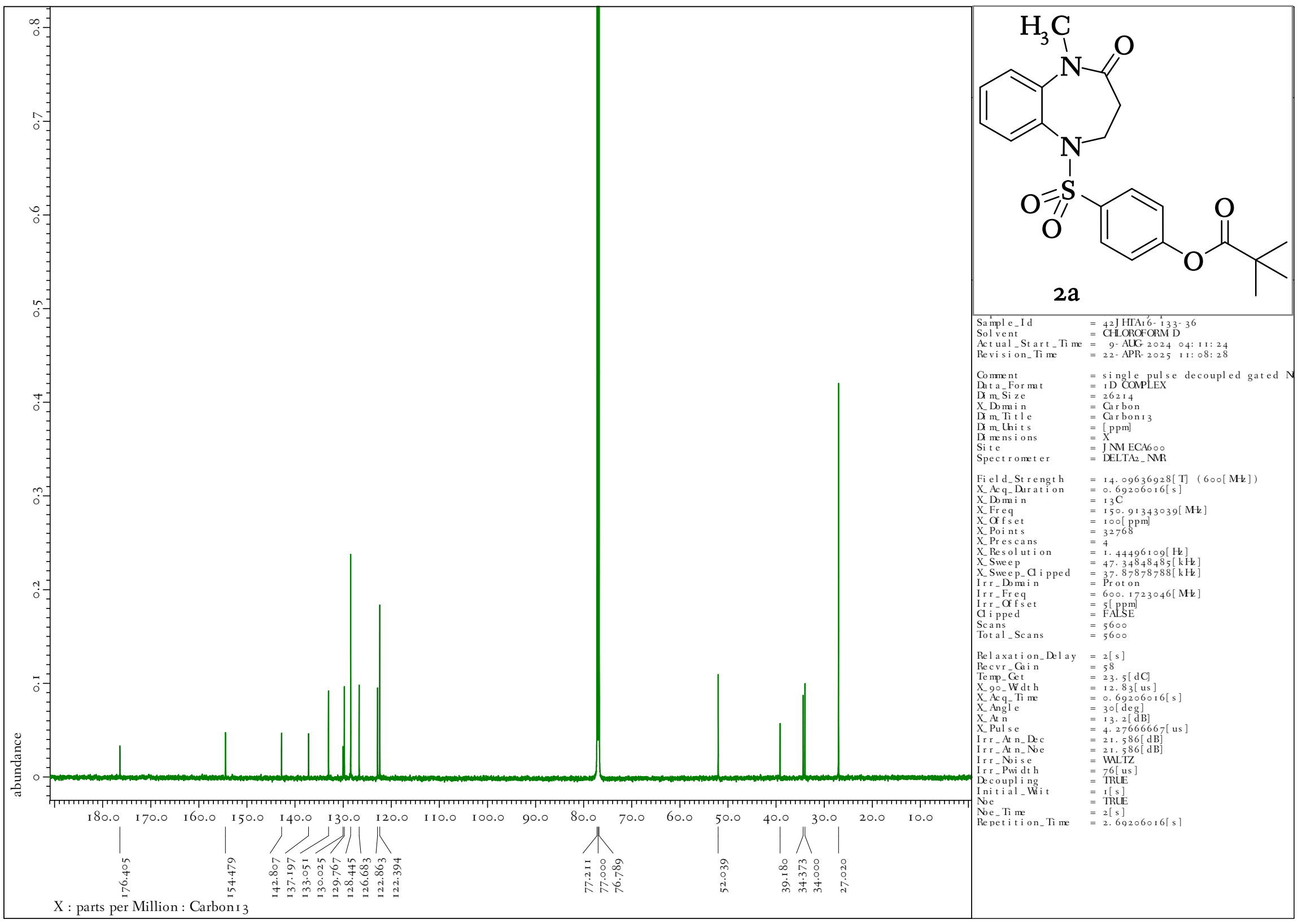

H-H COSY ($CDCl_3$)

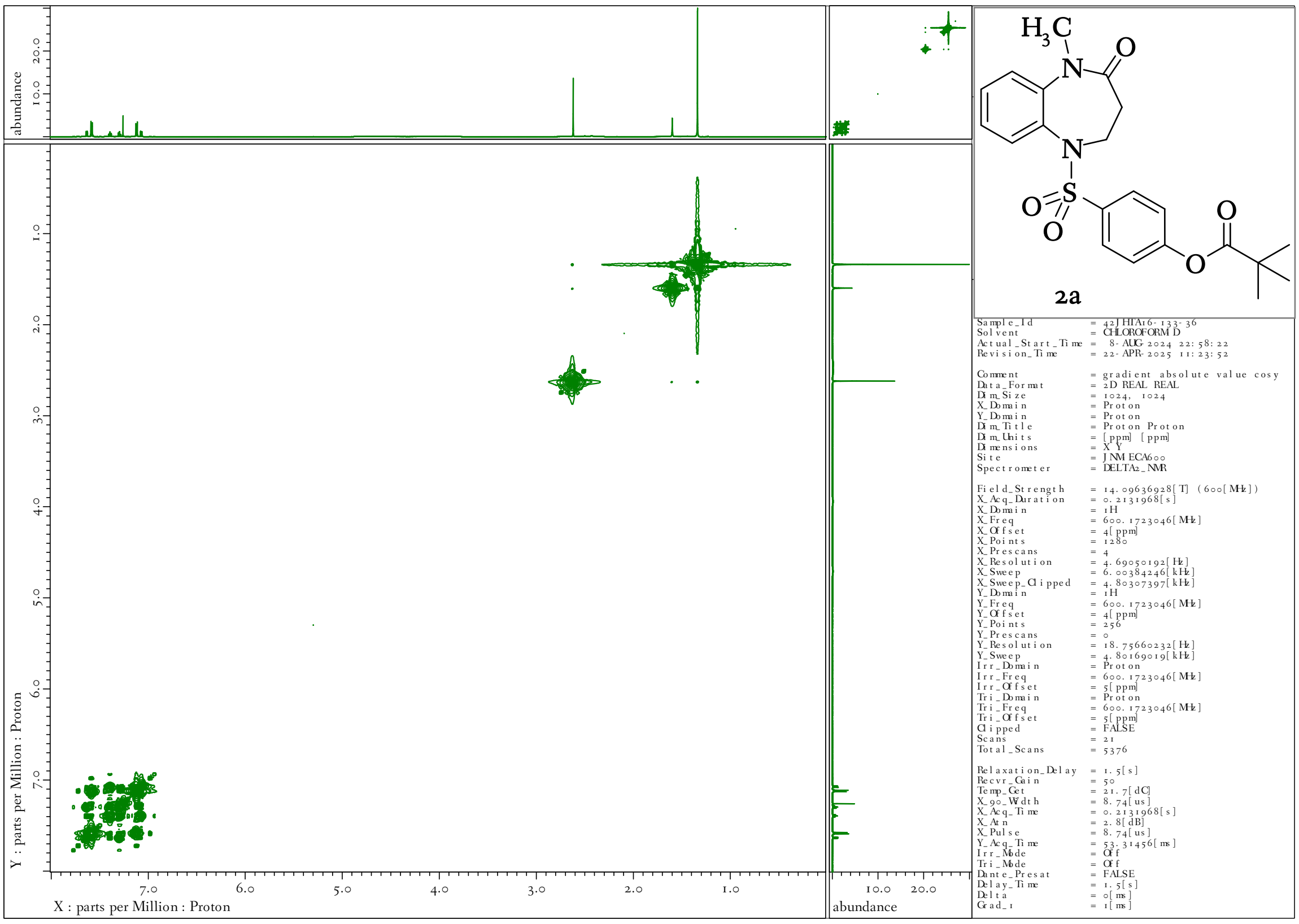

NOESY ($CDCl_3$)

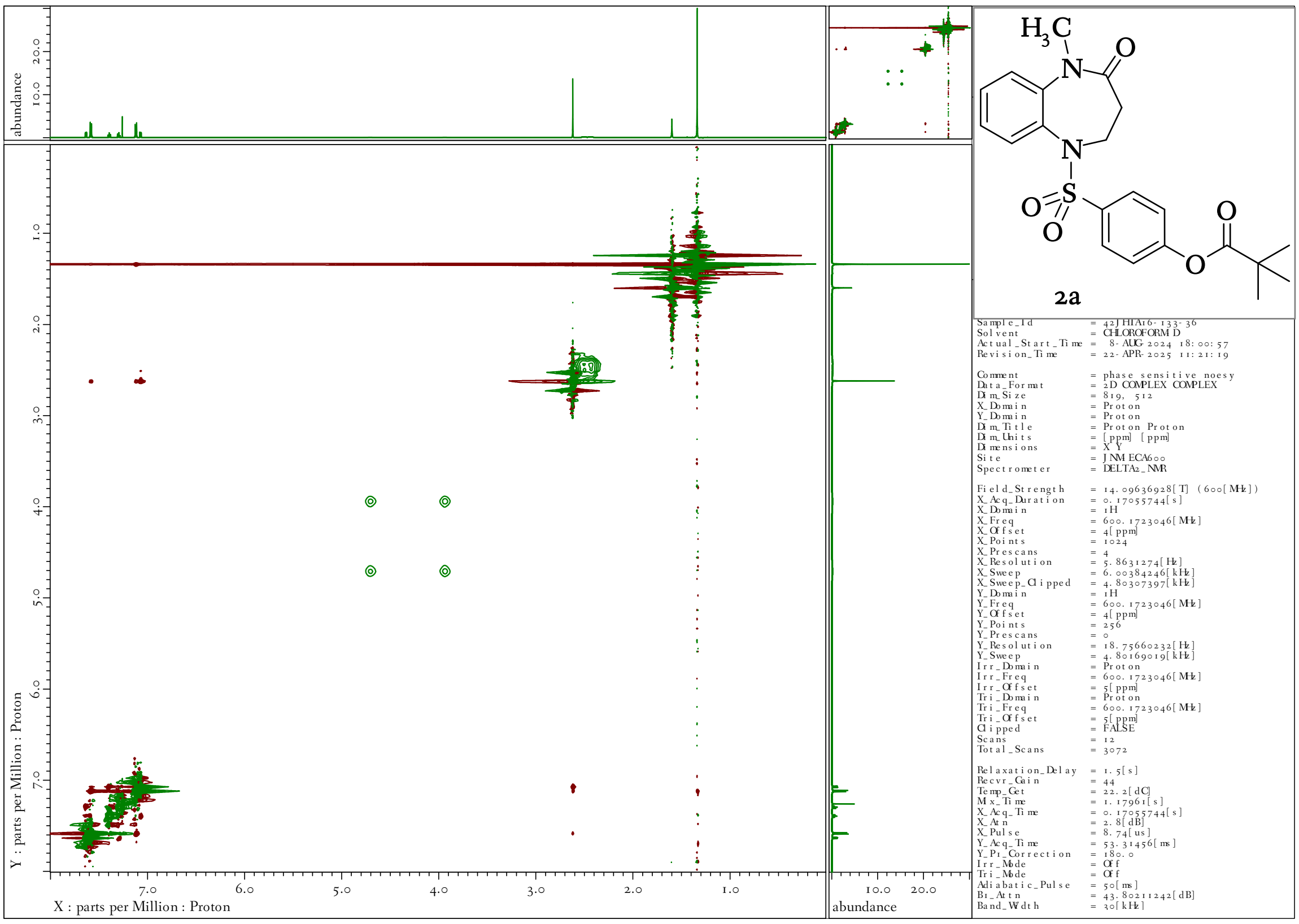

^{1}H-NMR ($CDCl_3$)

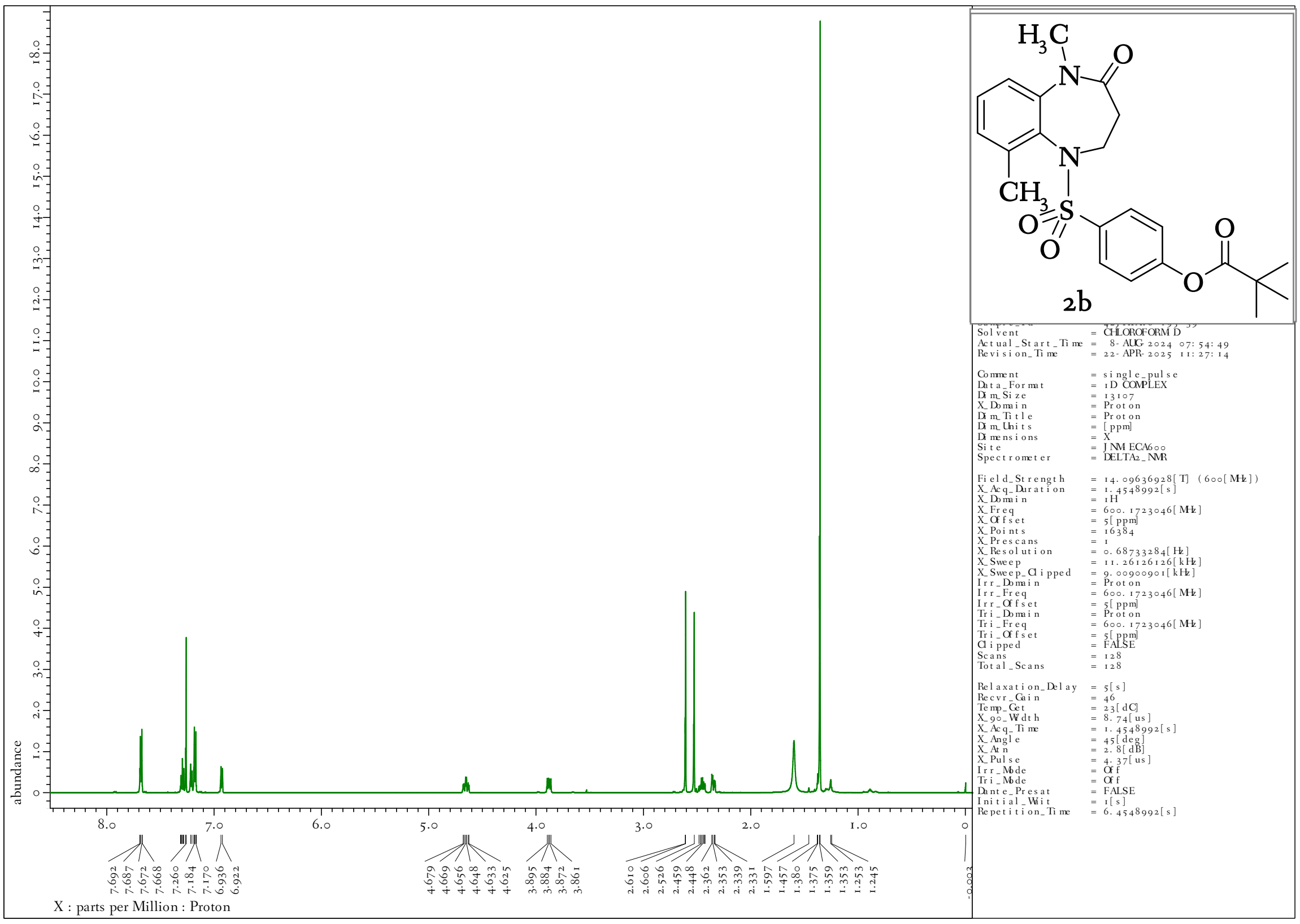

2b

^{13}C-NMR ($CDCl_3$)

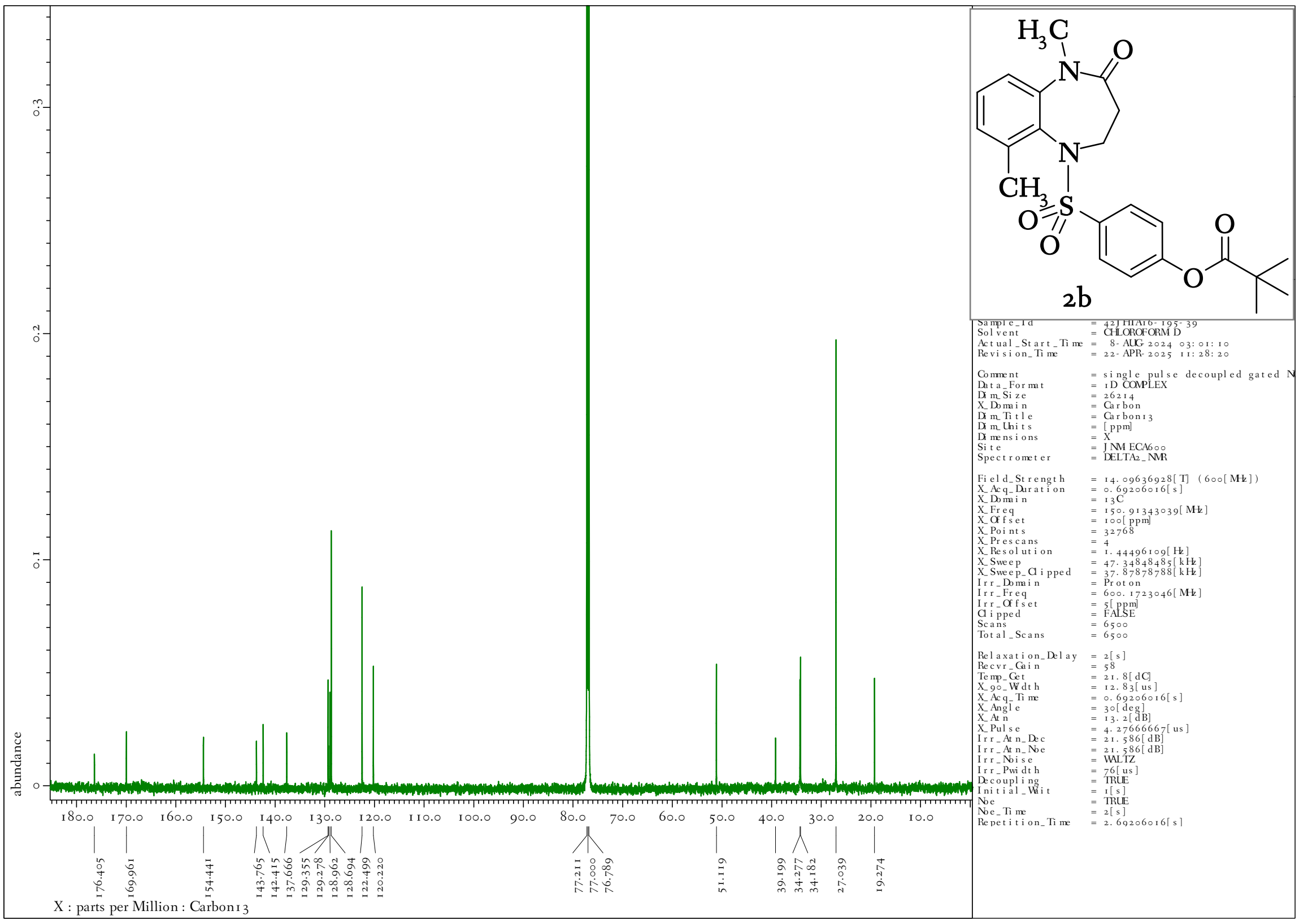

H-H COSY ($CDCl_3$)

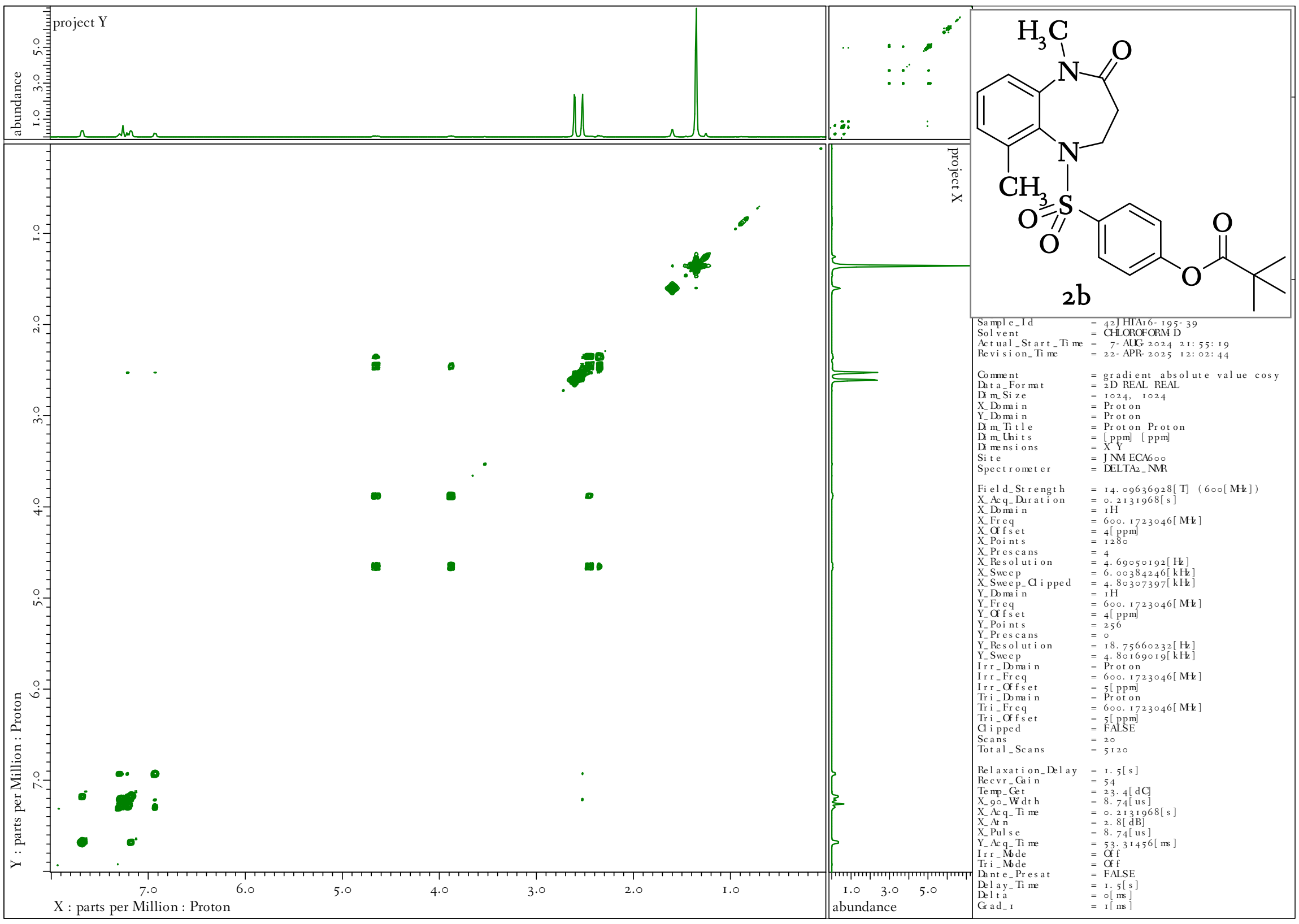

NOESY ($CDCl_3$)

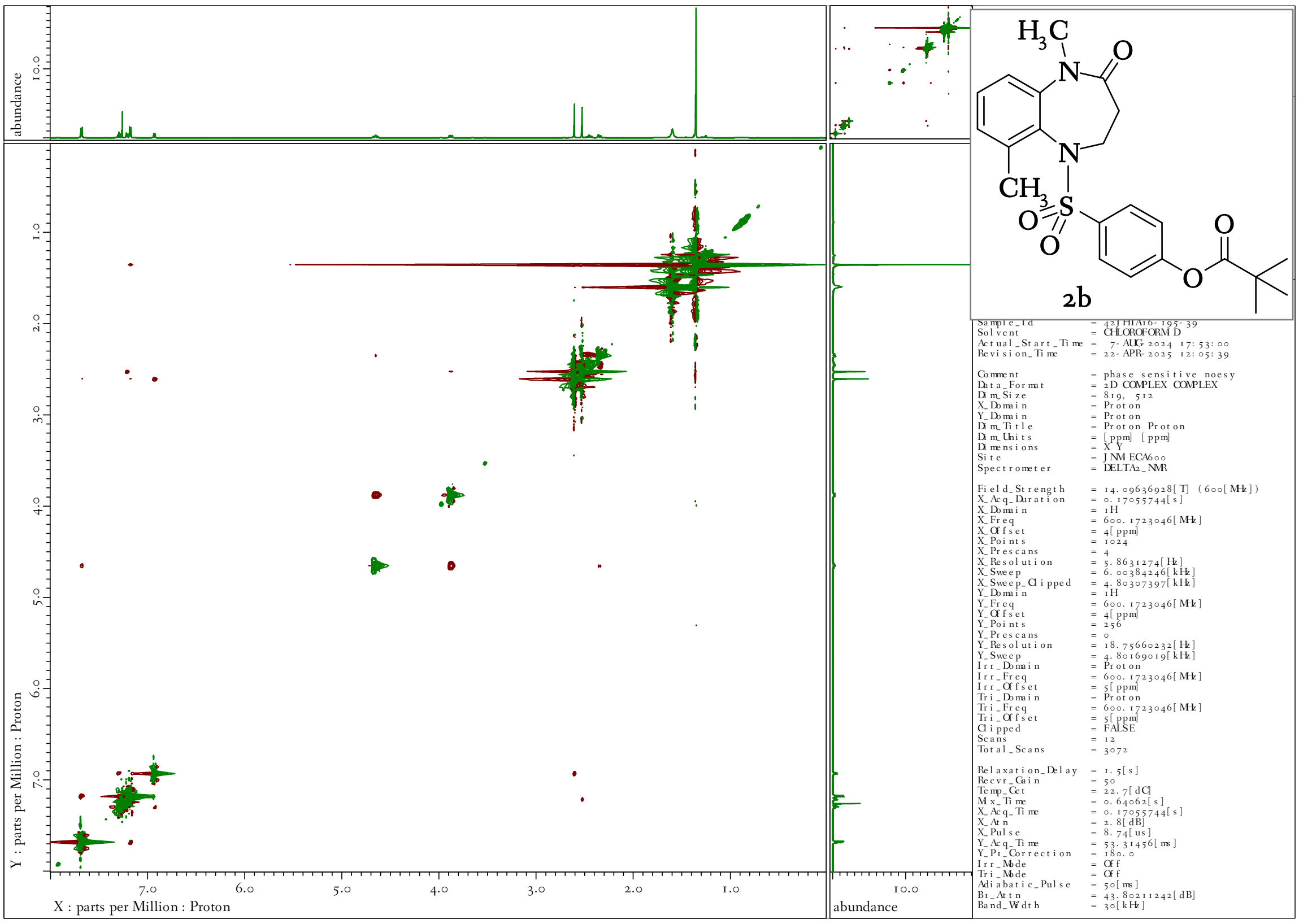

abundance
10.0
1.0
2.0
3.0
4.0
5.0
6.0
7.0
Y : parts per Million : Proton
X : parts per Million : Proton
H_3C
N
O
N
CH_3
O
S
O
O
O
2b
Sample_Id = 42JHIA16-195-39
Solvent = CHLOROFORM-D
Actual_Start_Time = 7-AUG-2024 17:53:00
Revision_Time = 22-APR-2025 12:05:39
Comment = phase sensitive noesy
Data_Format = 2D COMPLEX COMPLEX
Dim_Size = 819, 512
X_Domain = Proton
Y_Domain = Proton
Dim_Title = Proton Proton
Dim_Units = [ppm] [ppm]
Dimensions = X Y
Site = JNM-ECA600
Spectrometer = DELTA2_NMR
Field_Strength = 14.09636928[T] (600[MHz])
X_Acq_Duration = 0.17055744[s]
X_Domain = 1H
X_Freq = 600.1723046[MHz]
X_Offset = 4[ppm]
X_Points = 1024
X_Prescans = 4
X_Resolution = 5.8631274[Hz]
X_Sweep = 6.00384246[kHz]
X_Sweep_Clipped = 4.80307397[kHz]
Y_Domain = 1H
Y_Freq = 600.1723046[MHz]
Y_Offset = 4[ppm]
Y_Points = 256
Y_Prescans = 0
Y_Resolution = 18.75660232[Hz]
Y_Sweep = 4.80169019[kHz]
Irr_Domain = Proton
Irr_Freq = 600.1723046[MHz]
Irr_Offset = 5[ppm]
Tri_Domain = Proton
Tri_Freq = 600.1723046[MHz]
Tri_Offset = 5[ppm]
Clipped = FALSE
Scans = 12
Total_Scans = 3072
Relaxation_Delay = 1.5[s]
Recvr_Gain = 50
Temp_Get = 22.7[dC]
Mix_Time = 0.64062[s]
X_Acq_Time = 0.17055744[s]
X_Atn = 2.8[dB]
X_Pulse = 8.74[us]
Y_Acq_Time = 53.31456[ms]
Y_P1_Correction = 180.0
Irr_Mode = Off
Tri_Mode = Off
Adiabatic_Pulse = 50[ms]
B1_Attn = 43.80211242[dB]
Band_Width = 30[kHz]

¹H-NMR ($CDCl_3$)

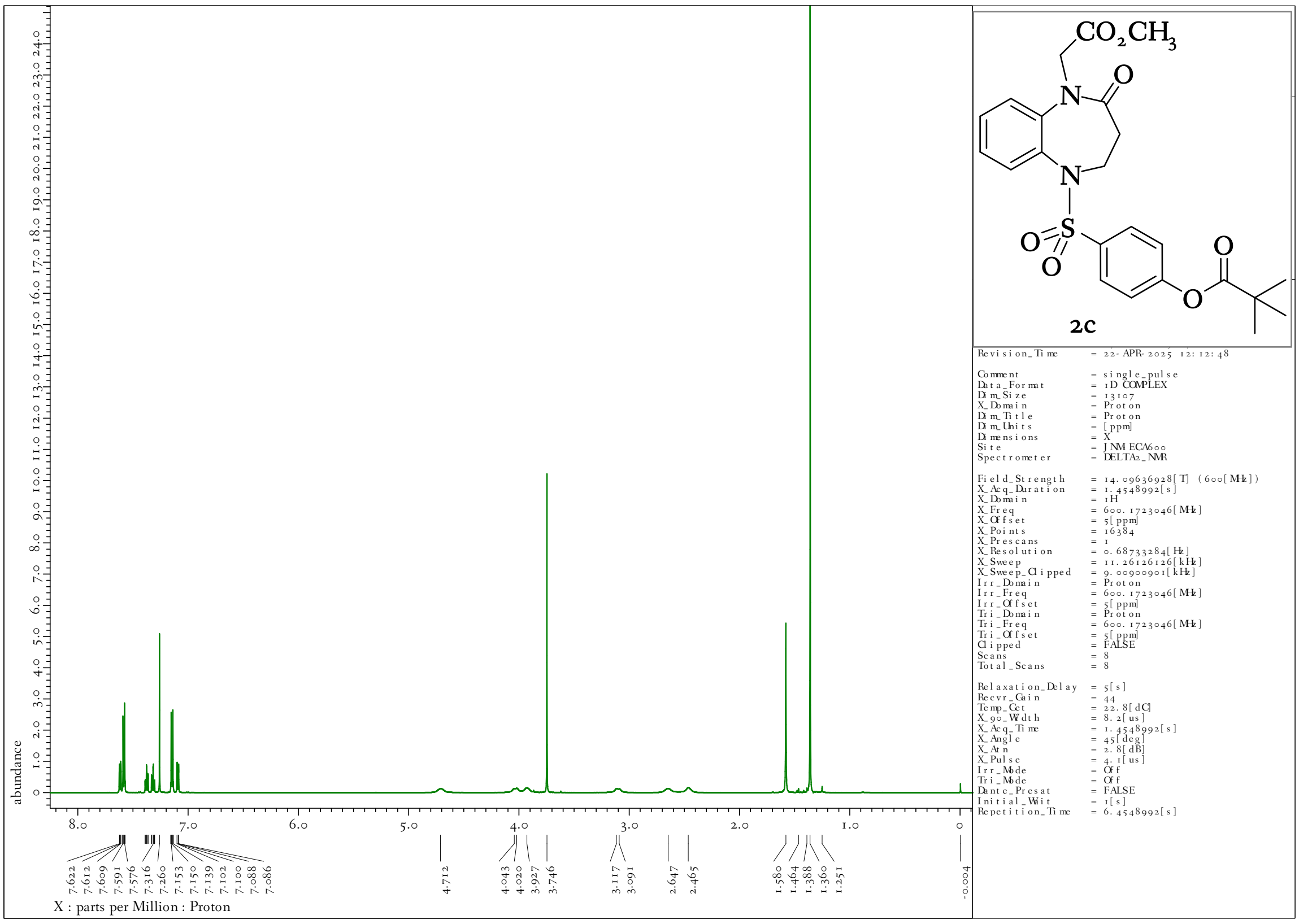

¹³C-NMR ($CDCl_3$)

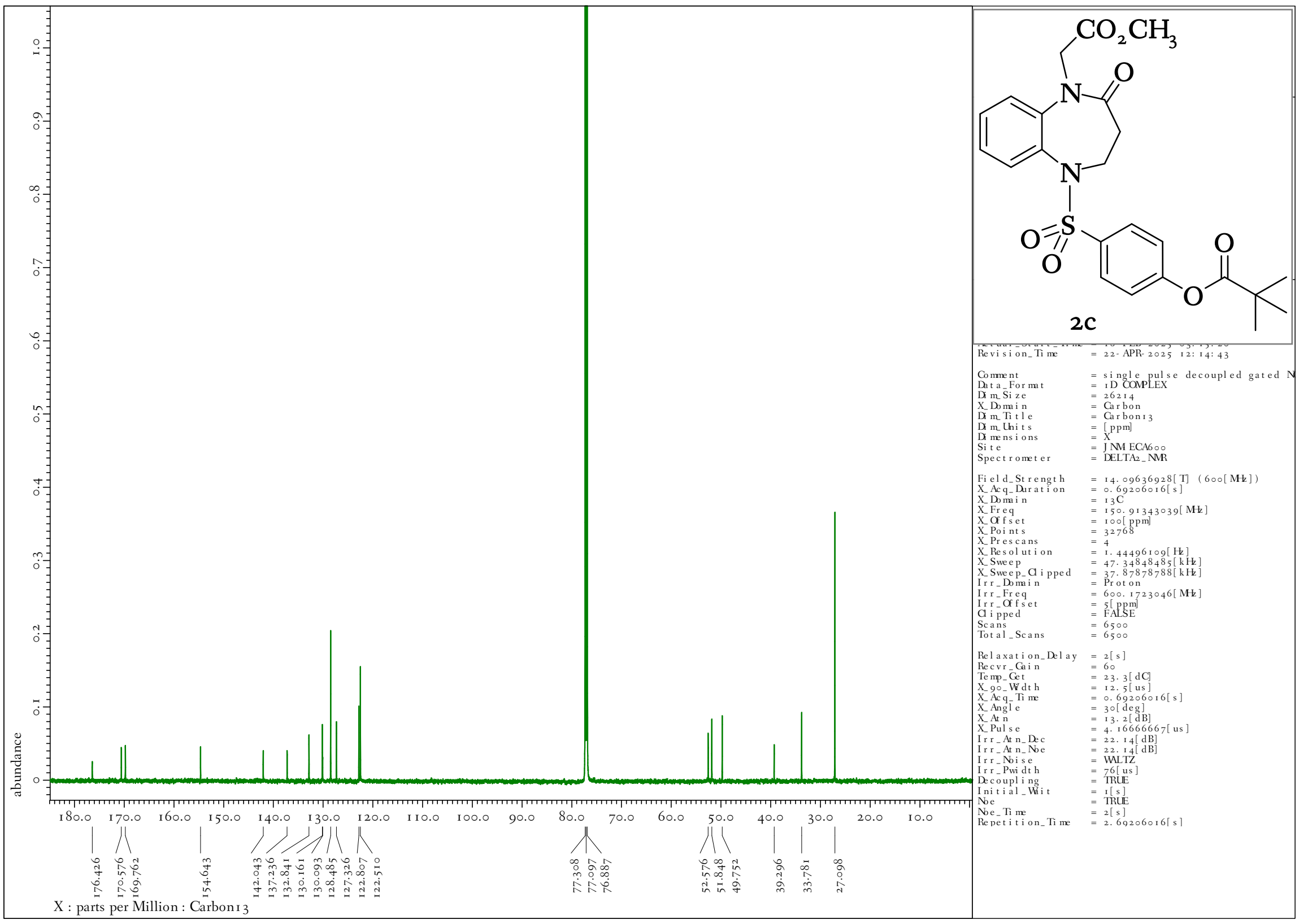

H-H COSY ($CDCl_3$)

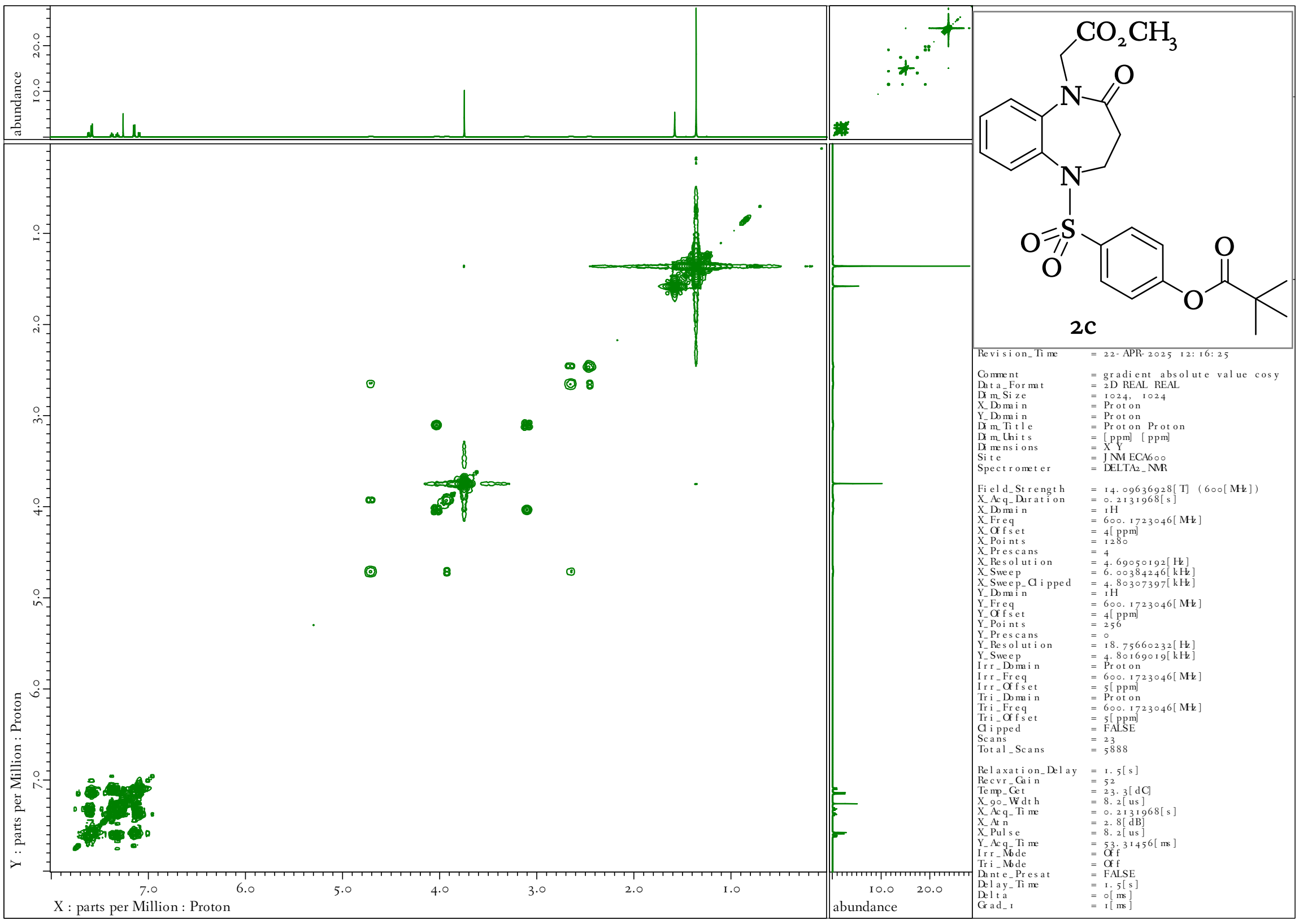

NOESY ($CDCl_3$)

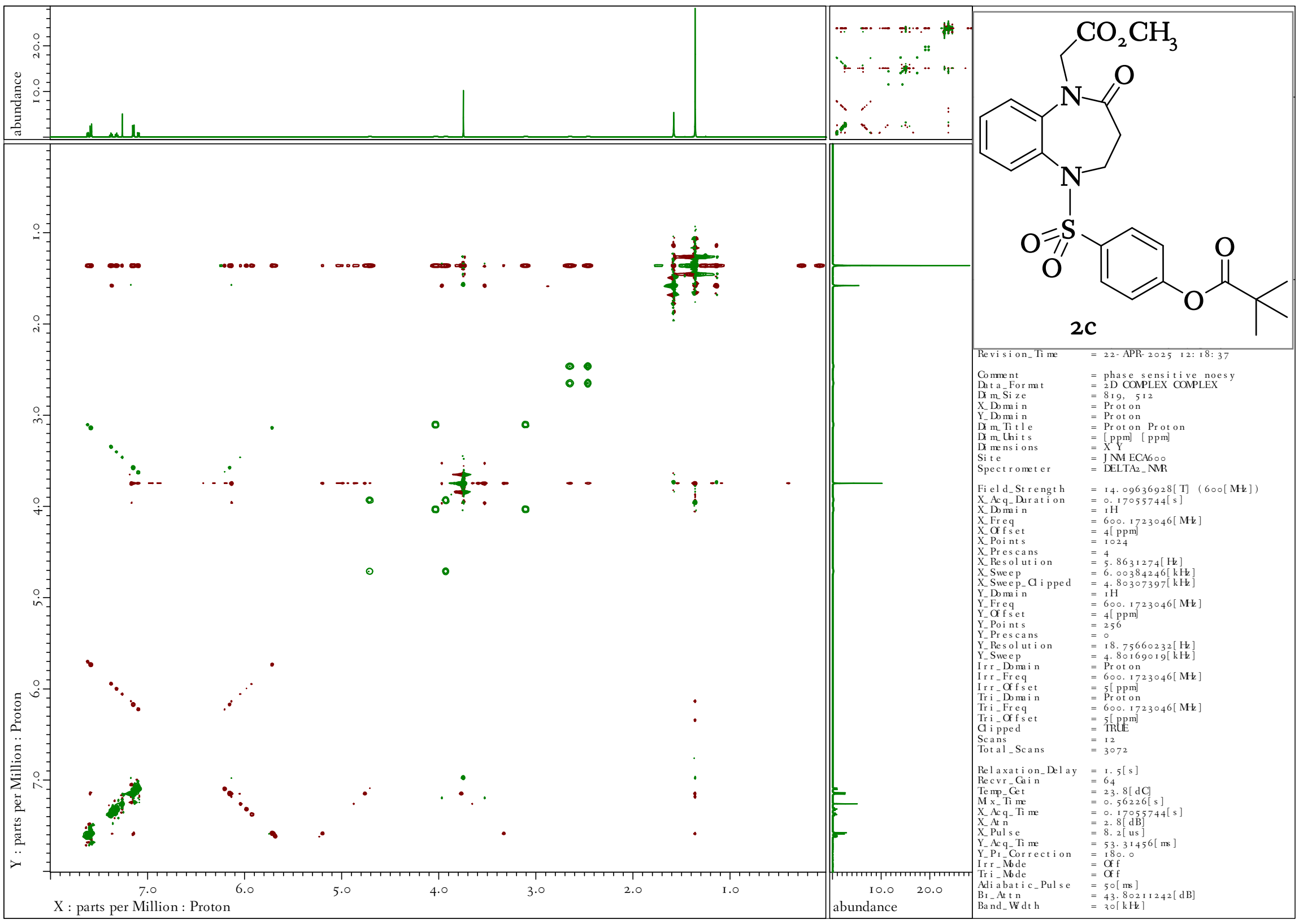

^{1}H-NMR ($CDCl_3$)

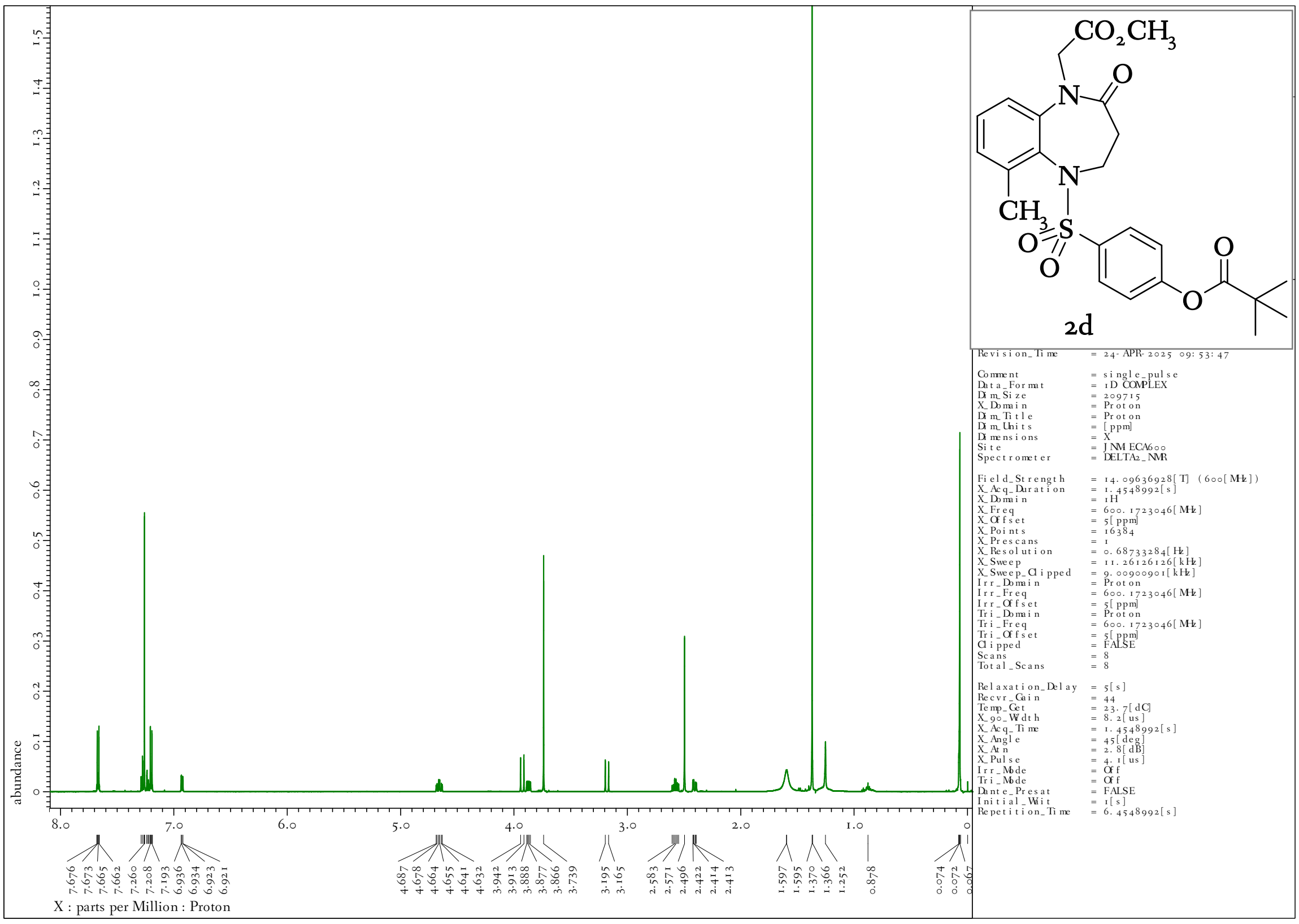

^{13}C-NMR ($CDCl_3$)

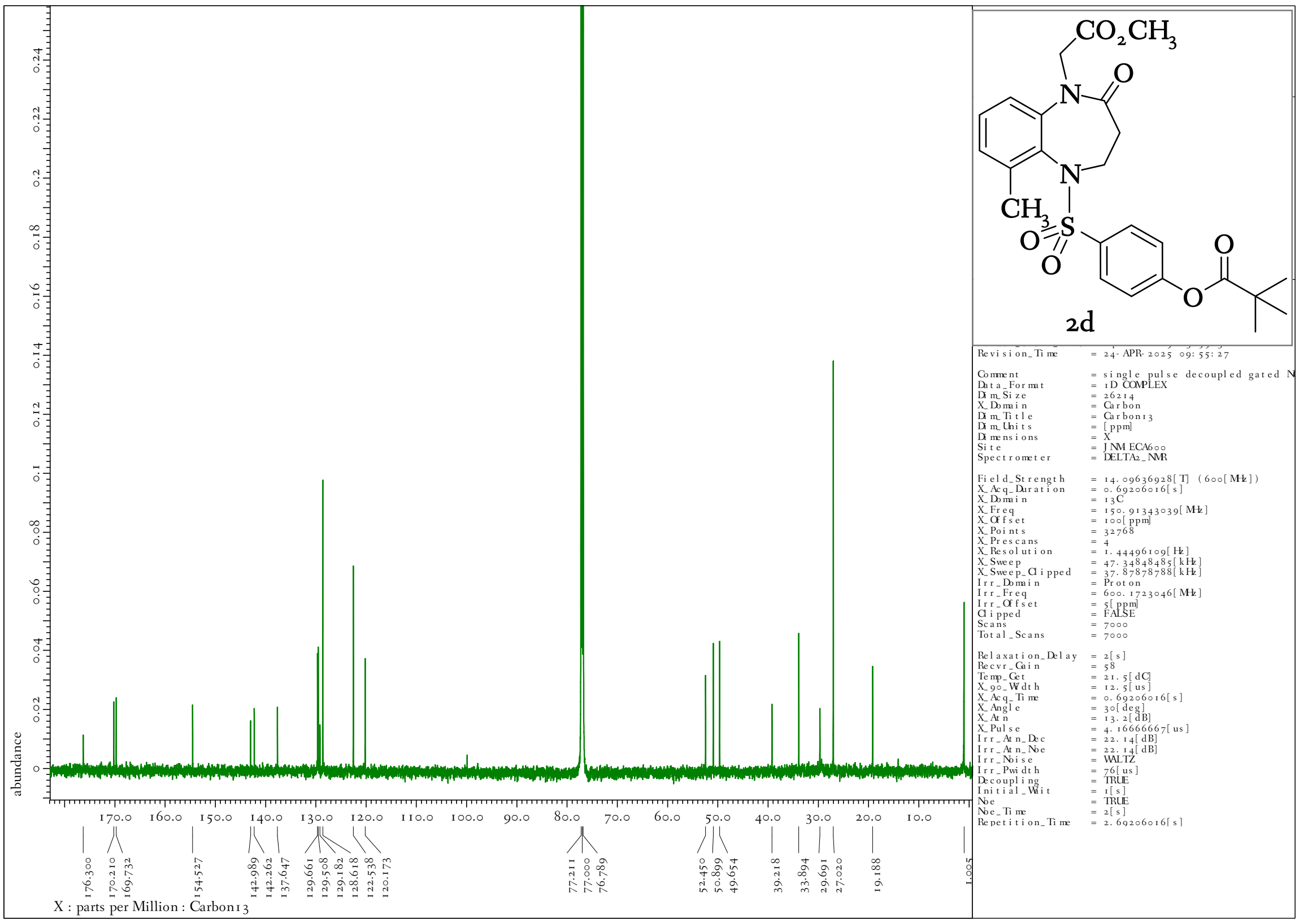

CO_2CH_3
N
O
N
CH_3
S
O
O
O
O
2d
Revision_Time = 24-APR-2025 09:55:27
Comment = single pulse decoupled gated N
Data_Format = 1D COMPLEX
Dim_Size = 26214
X_Domain = Carbon
Dim_Title = Carbon13
Dim_Units = [ppm]
Dimensions = X
Site = JNM ECA600
Spectrometer = DELTA2_NMR
Field_Strength = 14.09636928[T] (600[MHz])
X_Acq_Duration = 0.69206016[s]
X_Domain = 13C
X_Freq = 150.91343039[MHz]
X_Offset = 100[ppm]
X_Points = 32768
X_Prescans = 4
X_Resolution = 1.44496109[Hz]
X_Sweep = 47.34848485[kHz]
X_Sweep_Clipped = 37.87878788[kHz]
Irr_Domain = Proton
Irr_Freq = 600.1723046[MHz]
Irr_Offset = 5[ppm]
Clipped = FALSE
Scans = 7000
Total_Scans = 7000
Relaxation_Delay = 2[s]
Recvr_Gain = 58
Temp_Get = 21.5[dC]
X_90_Width = 12.5[us]
X_Acq_Time = 0.69206016[s]
X_Angle = 30[deg]
X_Atn = 13.2[dB]
X_Pulse = 4.16666667[us]
Irr_Atn_Dec = 22.14[dB]
Irr_Atn_Noe = 22.14[dB]
Irr_Noise = WALTZ
Irr_Pwidth = 76[us]
Decoupling = TRUE
Initial_Wait = 1[s]
Noe = TRUE
Noe_Time = 2[s]
Repetition_Time = 2.69206016[s]
abundance
0
0.02
0.04
0.06
0.08
0.1
0.12
0.14
0.16
0.18
0.2
0.22
0.24
170.0
160.0
150.0
140.0
130.0
120.0
110.0
100.0
90.0
80.0
70.0
60.0
50.0
40.0
30.0
20.0
10.0
176.300
170.210
169.732
154.527
142.989
142.262
137.647
129.661
129.508
129.182
128.618
122.538
120.173
77.211
77.000
76.789
52.450
50.899
49.654
39.218
33.894
29.691
27.020
19.188
1.005
X : parts per Million : Carbon13

H-H COSY ($CDCl_3$)

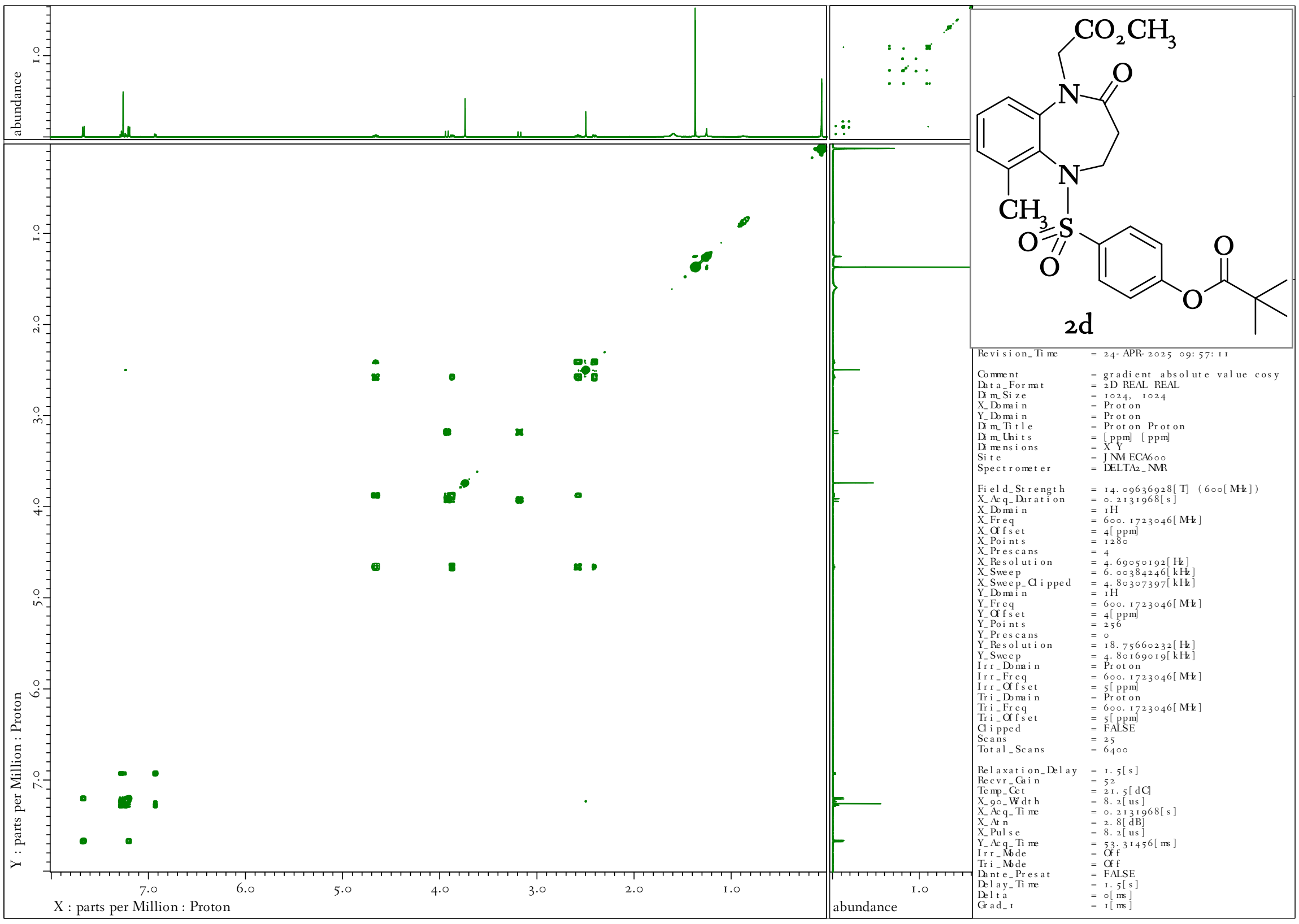

NOESY ($CDCl_3$)

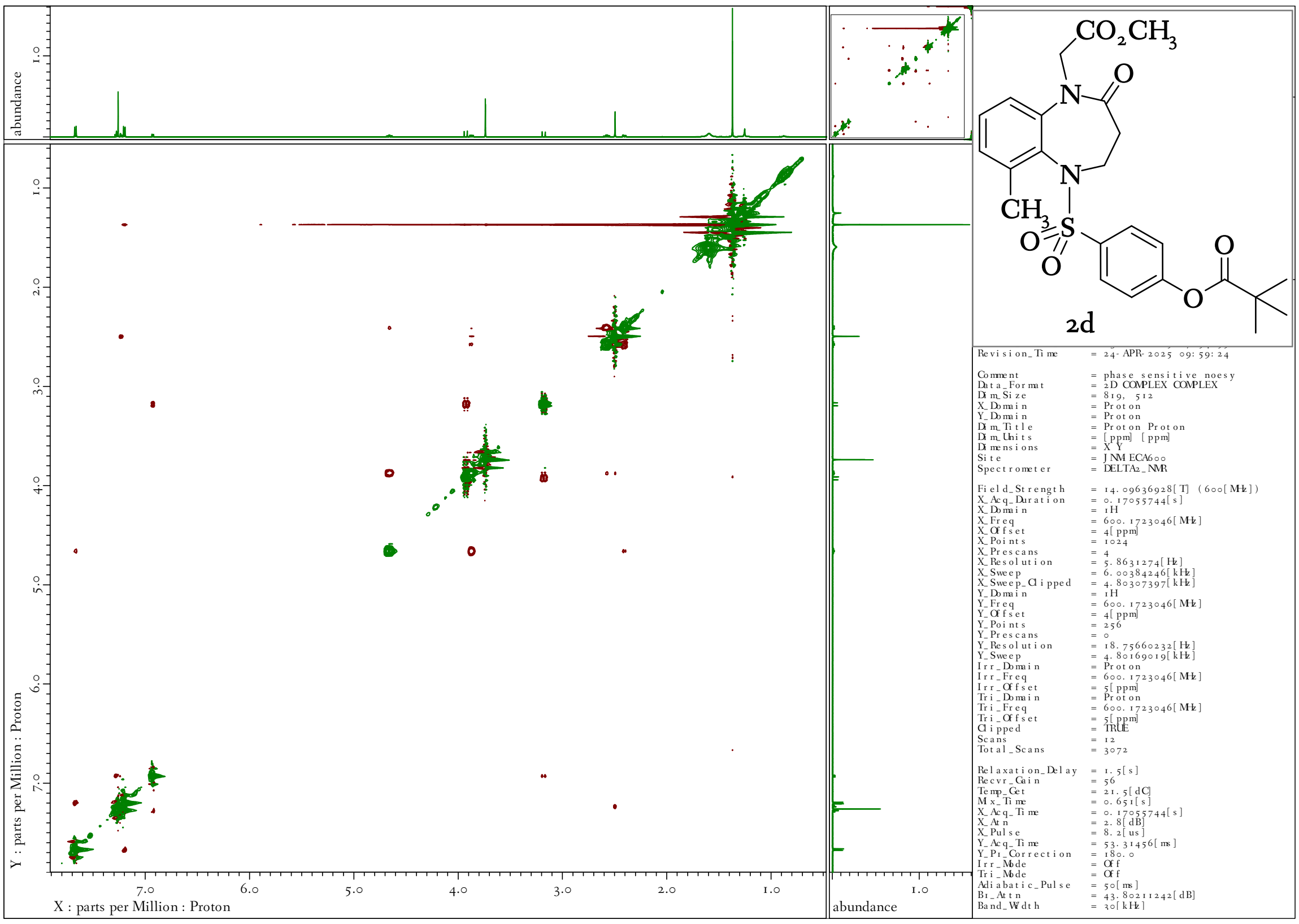

^{1}H-NMR ($CDCl_3$)

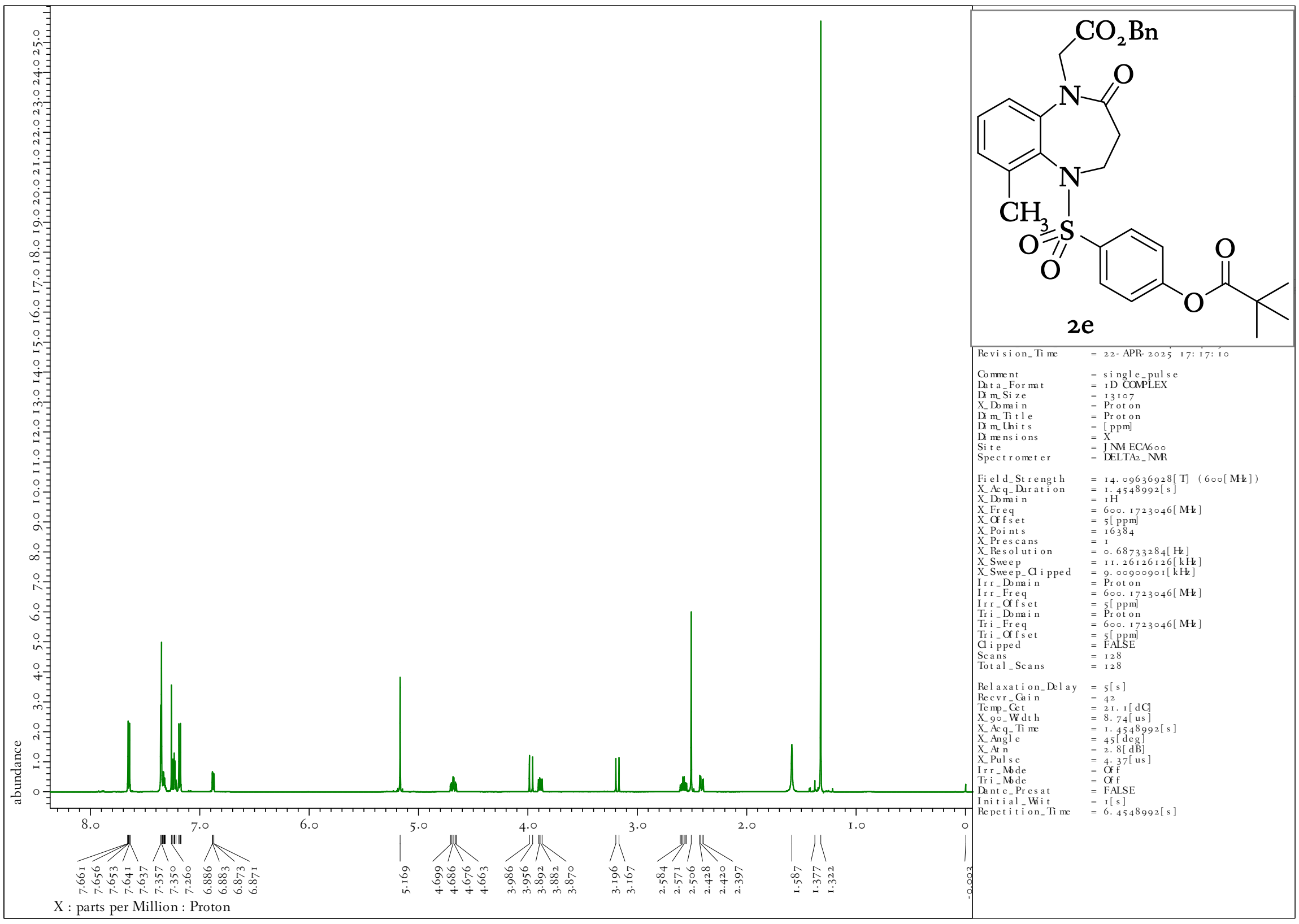

^{13}C-NMR ($CDCl_3$)

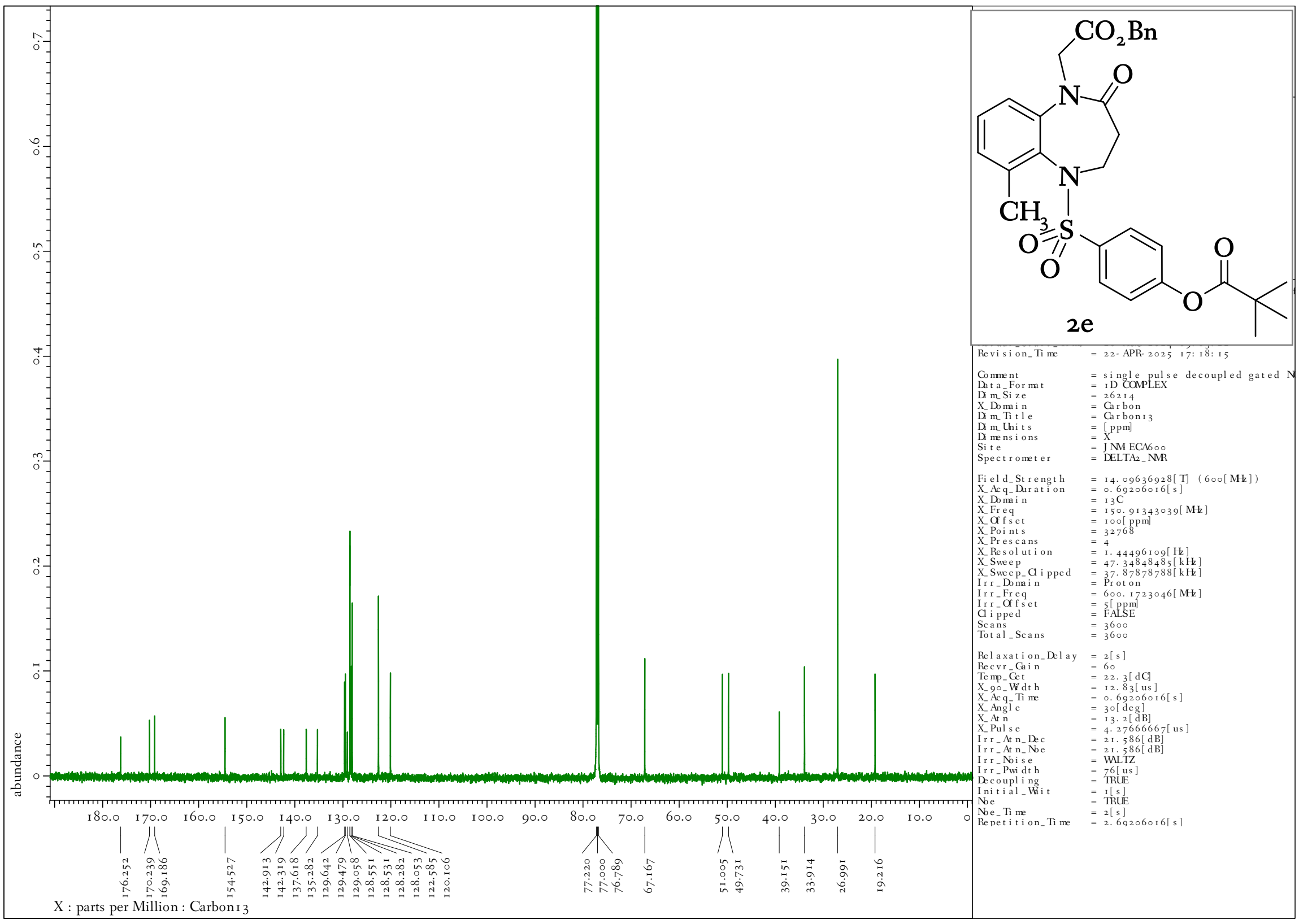

H-H COSY ($CDCl_3$)

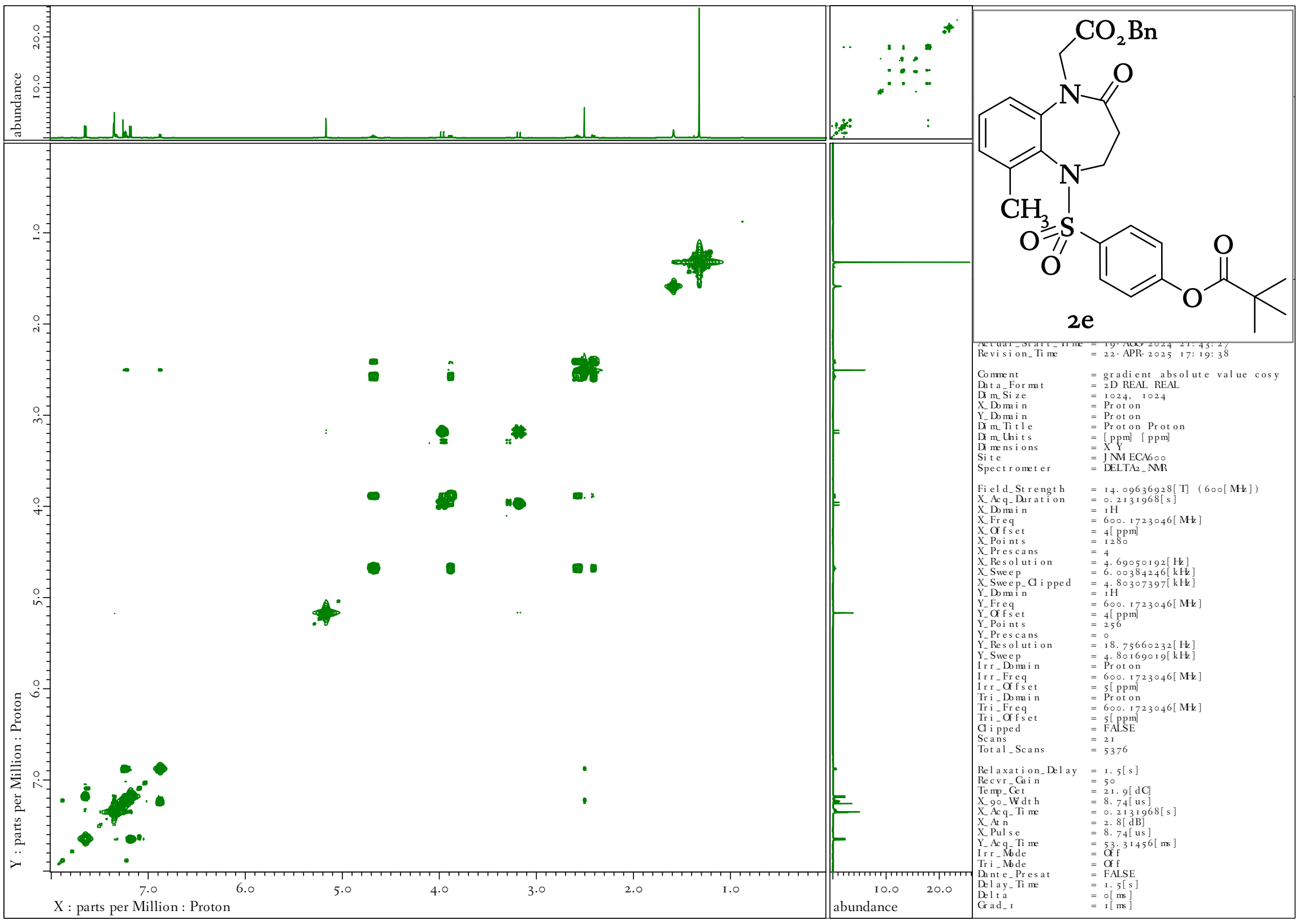

NOESY ($CDCl_3$)

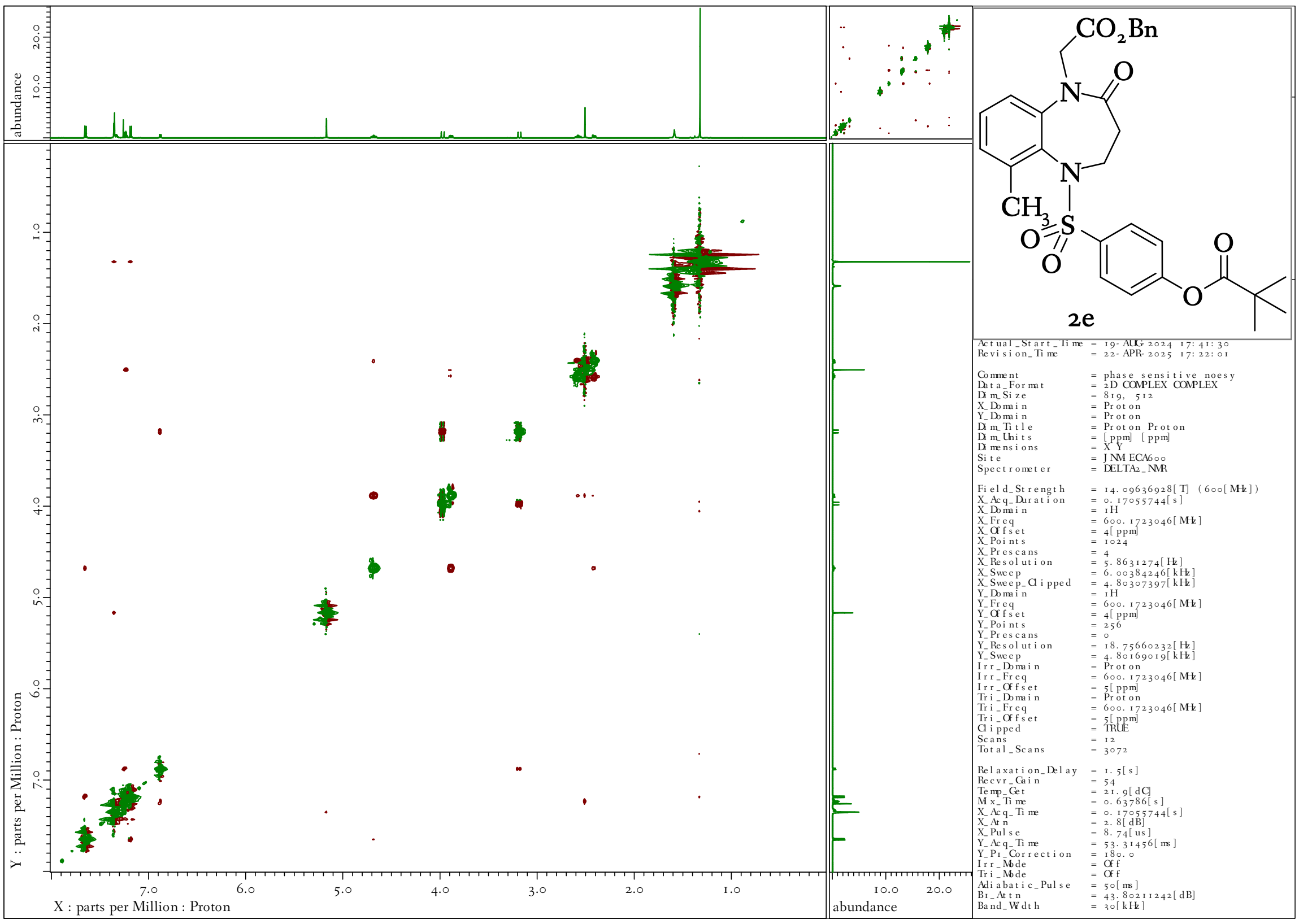

^{1}H-NMR (CD_3OD)

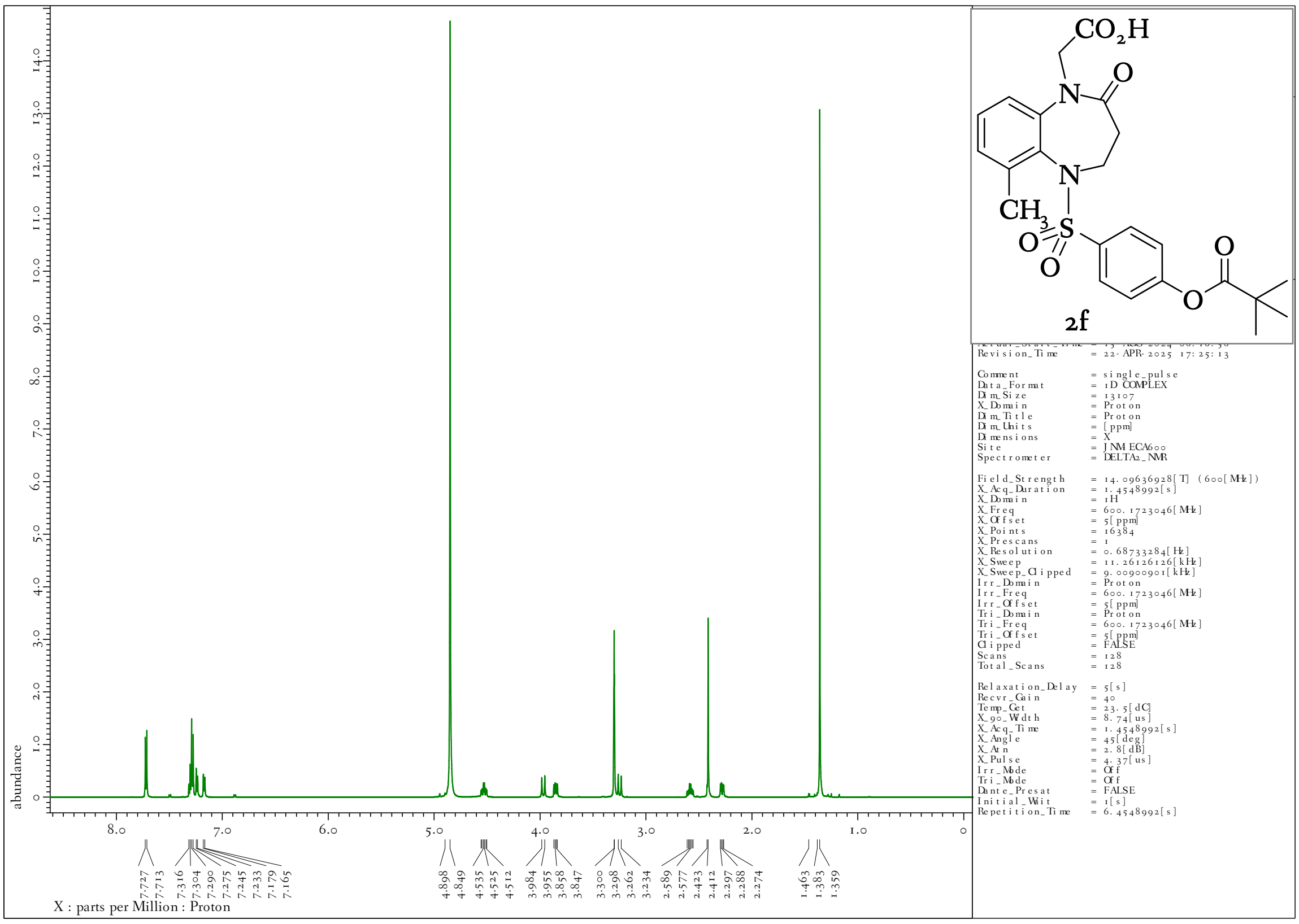

^{13}C-NMR (CD_3OD)

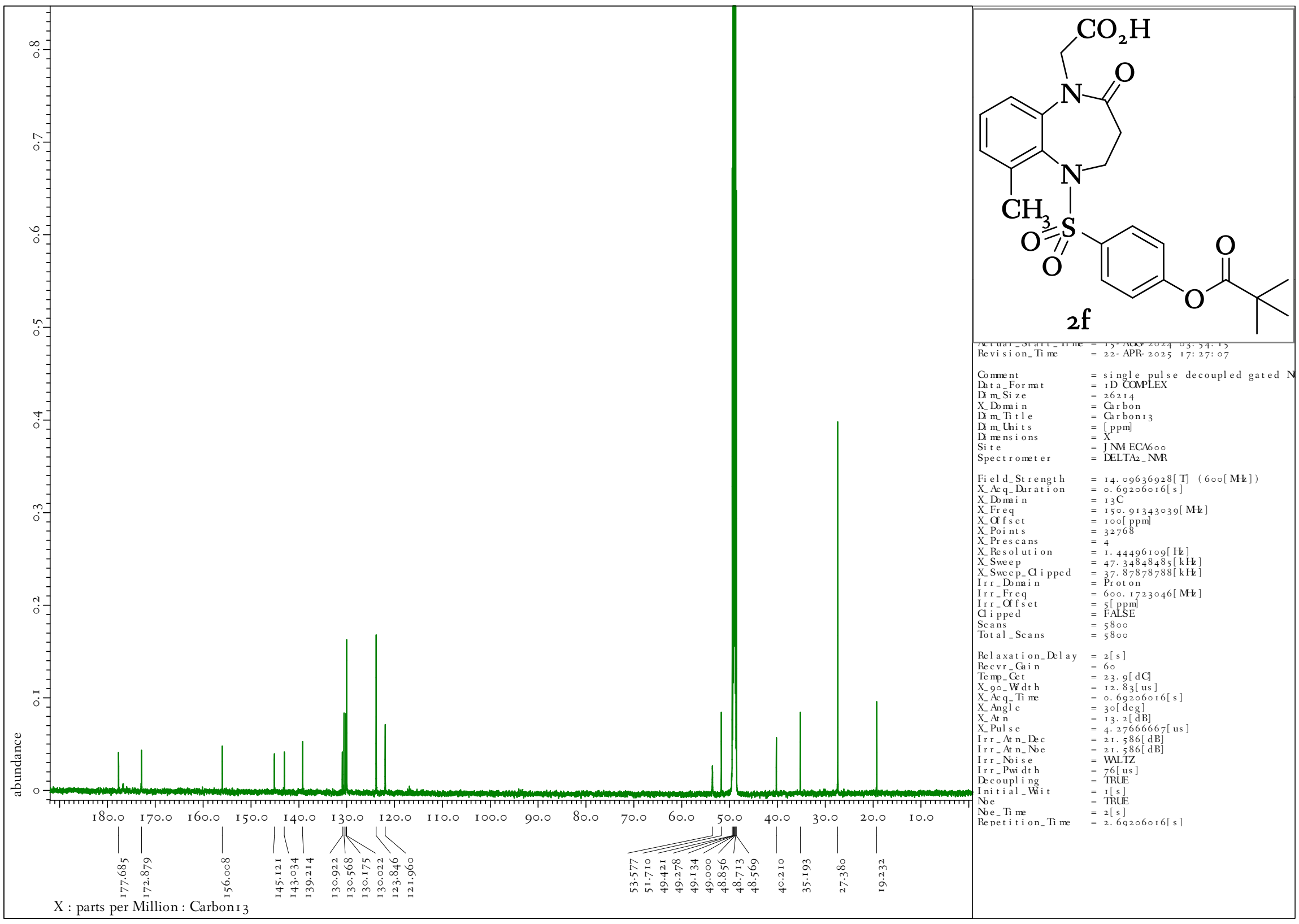

H-H COSY (CD_3OD)

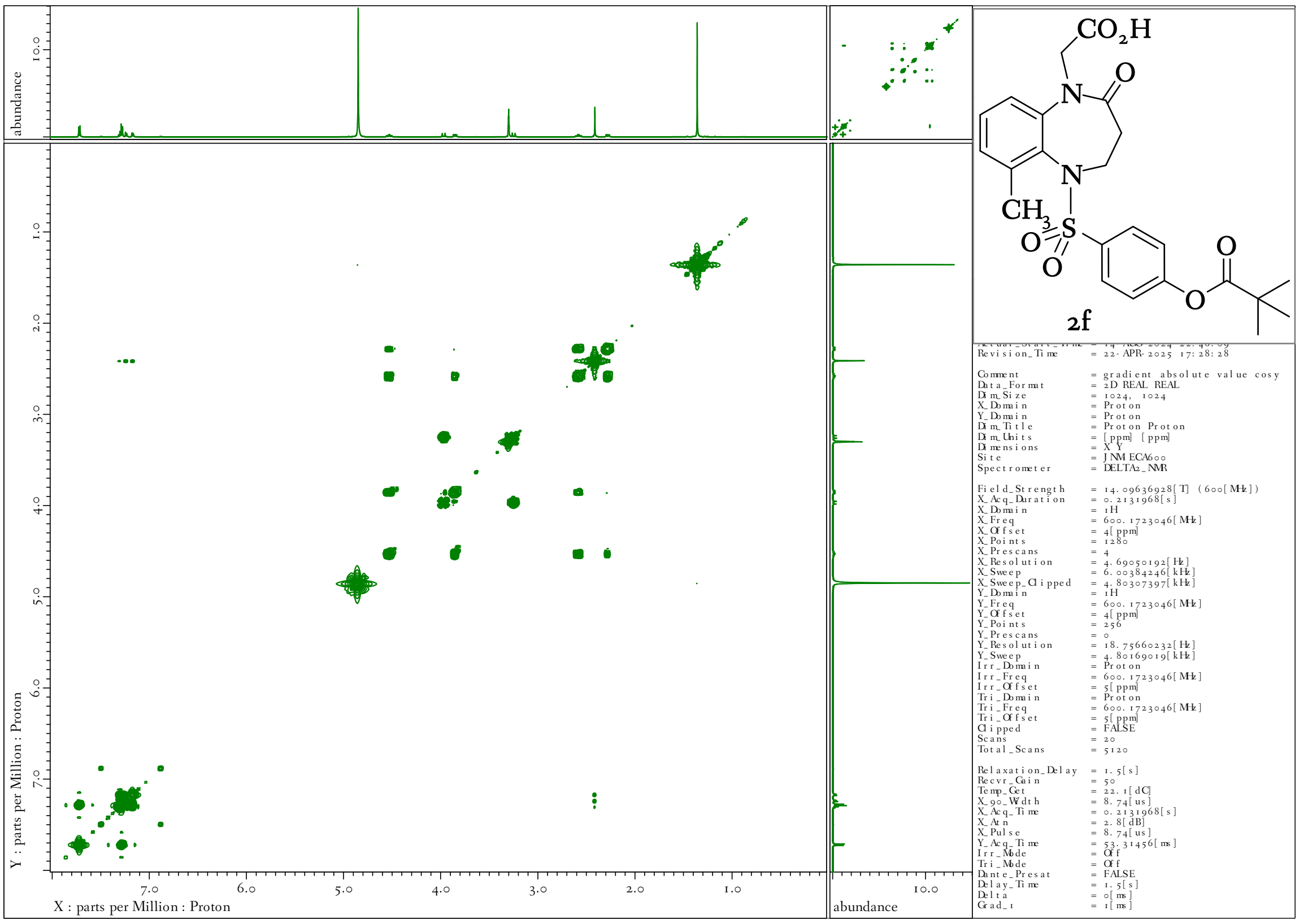

NOESY (CD_3OD)

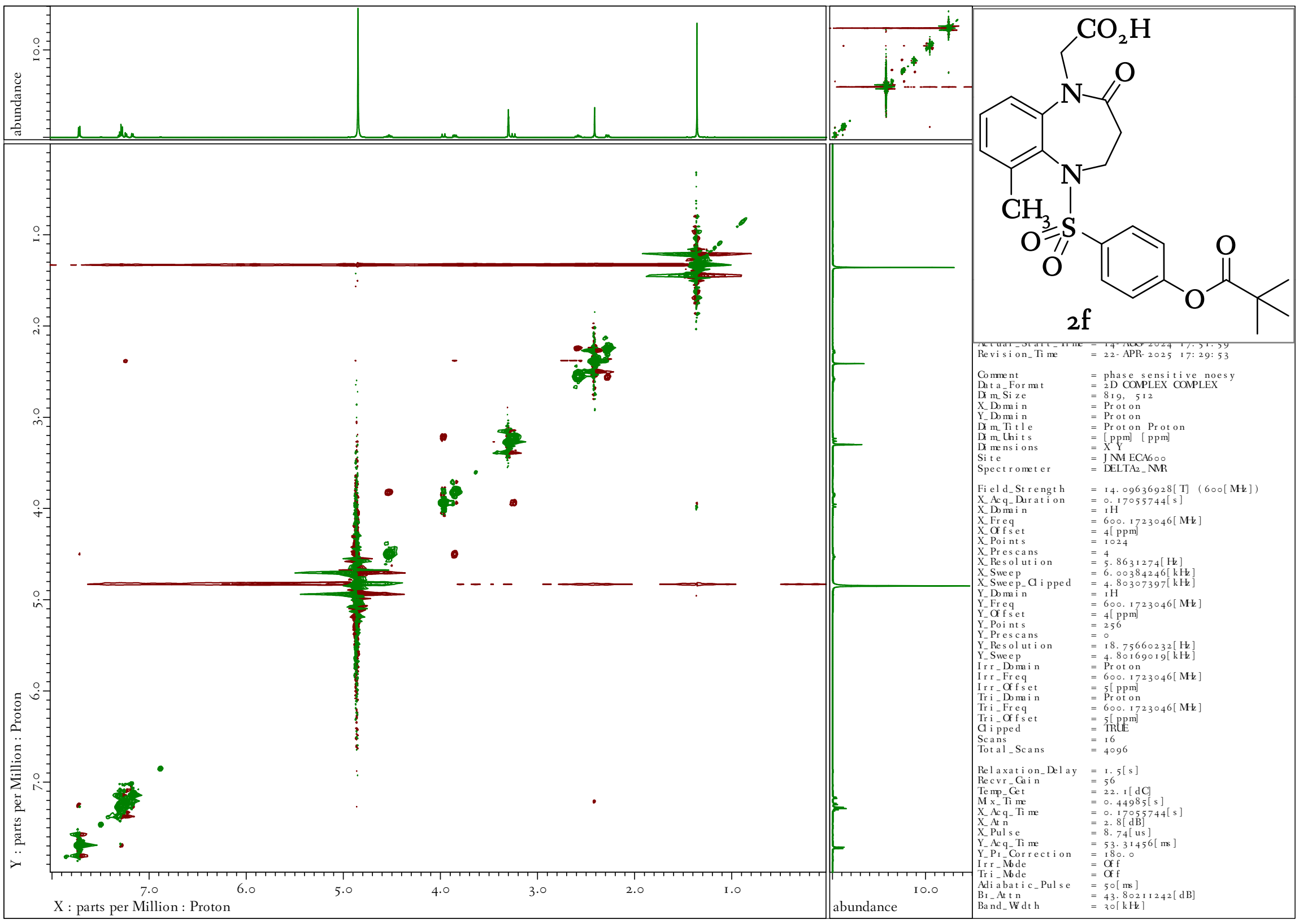

^{1}H-NMR ($CDCl_3$)

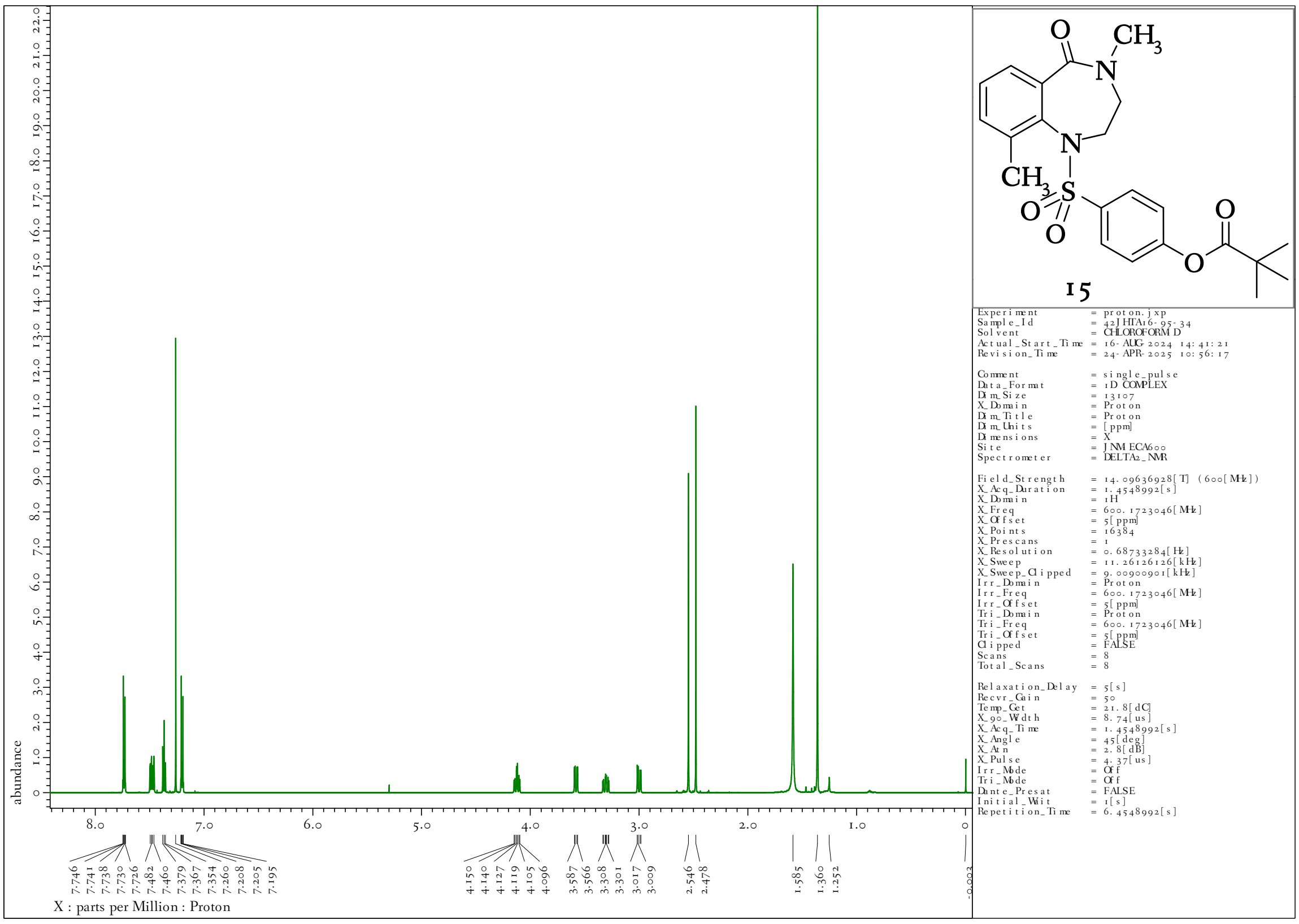

^{13}C-NMR ($CDCl_3$)

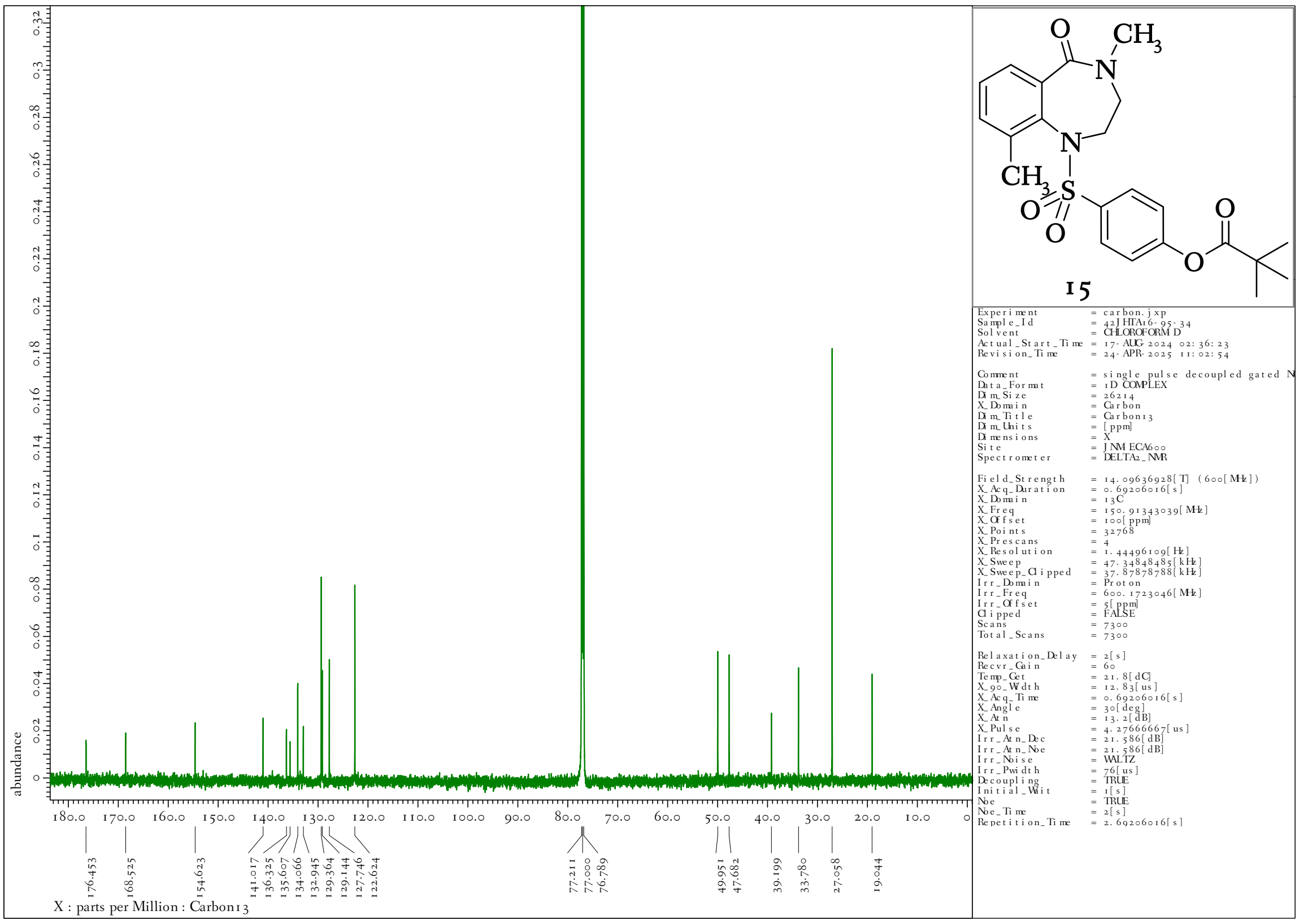
15
Experiment = carbon.jxp
Sample_Id = 42JHTA16-95-34
Solvent = CHLOROFORM-D
Actual_Start_Time = 17-AUG-2024 02:36:23
Revision_Time = 24-APR-2025 11:02:54
Comment = single pulse decoupled gated N
Data_Format = 1D COMPLEX
Dim_Size = 26214
X_Domain = Carbon
Dim_Title = Carbon13
Dim_Units = [ppm]
Dimensions = X
Site = JNM-ECA600
Spectrometer = DELTA2_NMR
Field_Strength = 14.09636928[T] (600[MHz])
X_Acq_Duration = 0.69206016[s]
X_Domain = 13C
X_Freq = 150.91343039[MHz]
X_Offset = 100[ppm]
X_Points = 32768
X_Prescans = 4
X_Resolution = 1.44496109[Hz]
X_Sweep = 47.34848485[kHz]
X_Sweep_Clipped = 37.87878788[kHz]
Irr_Domain = Proton
Irr_Freq = 600.1723046[MHz]
Irr_Offset = 5[ppm]
Clipped = FALSE
Scans = 7300
Total_Scans = 7300
Relaxation_Delay = 2[s]
Recvr_Gain = 60
Temp_Get = 21.8[dC]
X_90_Width = 12.83[us]
X_Acq_Time = 0.69206016[s]
X_Angle = 30[deg]
X_Atn = 13.2[dB]
X_Pulse = 4.27666667[us]
Irr_Atn_Dec = 21.586[dB]
Irr_Atn_Noe = 21.586[dB]
Irr_Noise = WALTZ
Irr_Pwidth = 76[us]
Decoupling = TRUE
Initial_Wait = 1[s]
Noe = TRUE
Noe_Time = 2[s]
Repetition_Time = 2.69206016[s]
abundance
176.453
168.525
154.623
141.017
136.325
135.607
134.066
132.945
129.364
129.144
127.746
122.624
77.211
77.000
76.789
49.951
47.682
39.199
33.780
27.058
19.044
X : parts per Million : Carbon13

H-H COSY ($CDCl_3$)

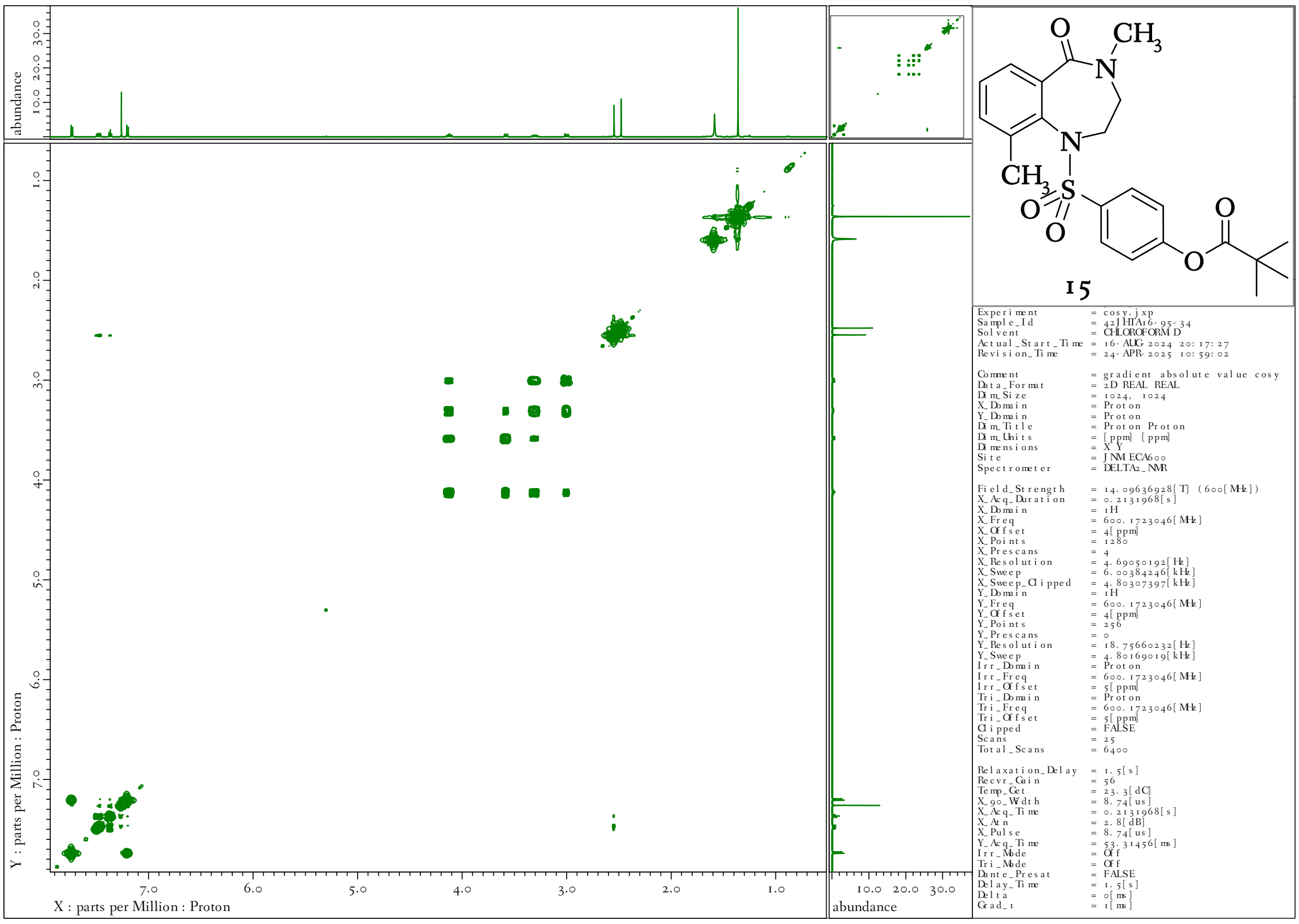

NOESY ($CDCl_3$)

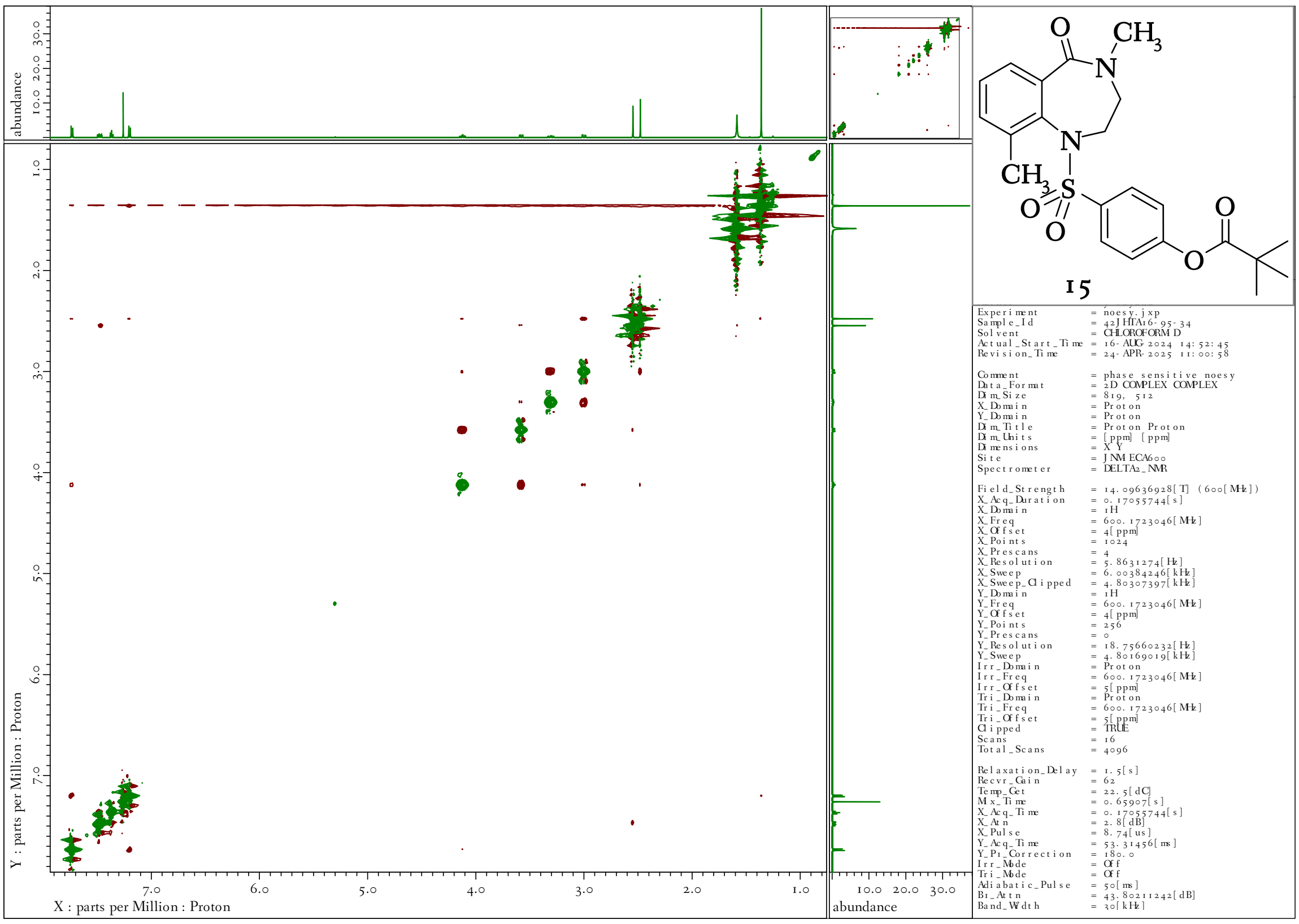

^{1}H-NMR ($CDCl_3$)

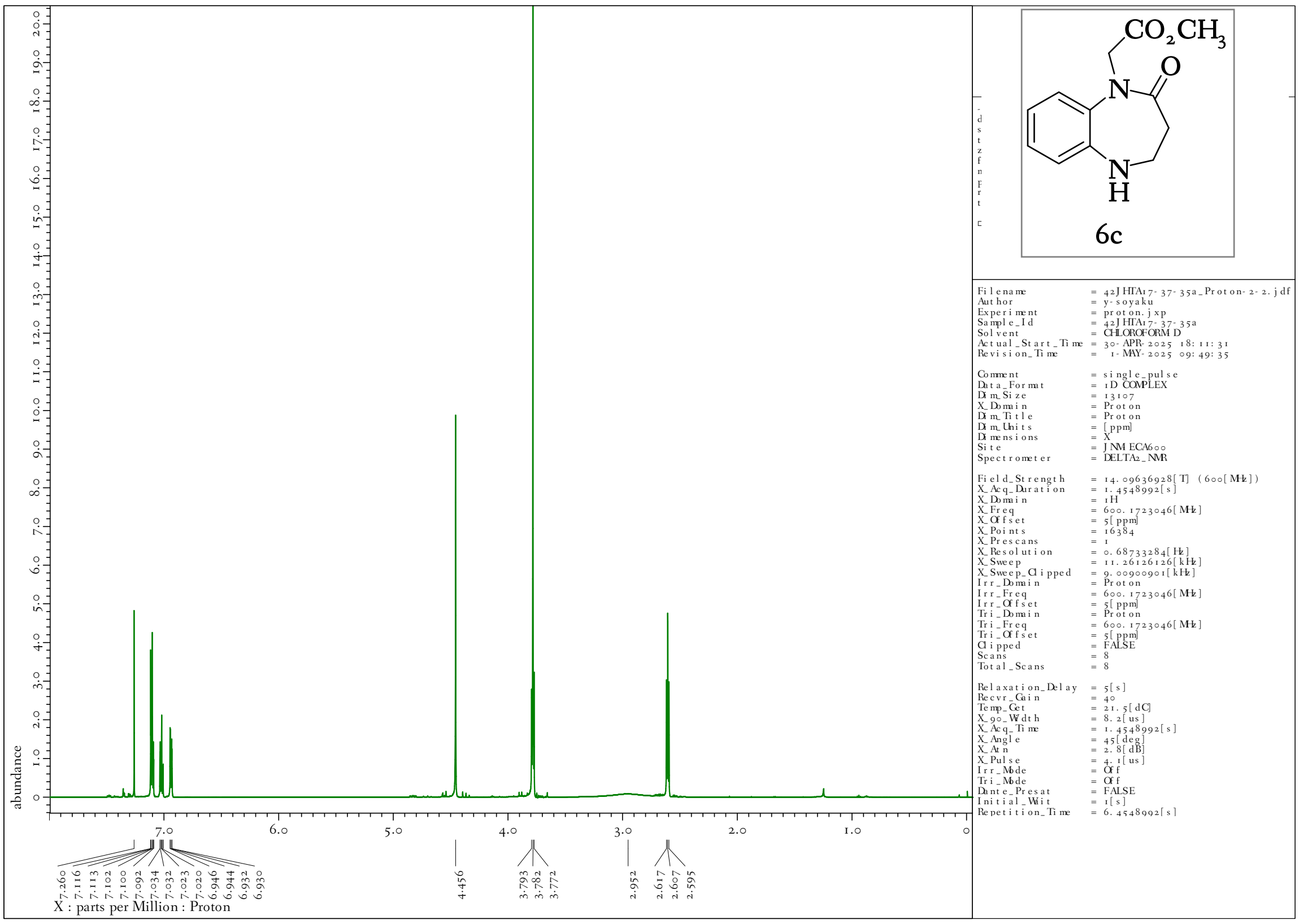

^{13}C-NMR ($CDCl_3$)

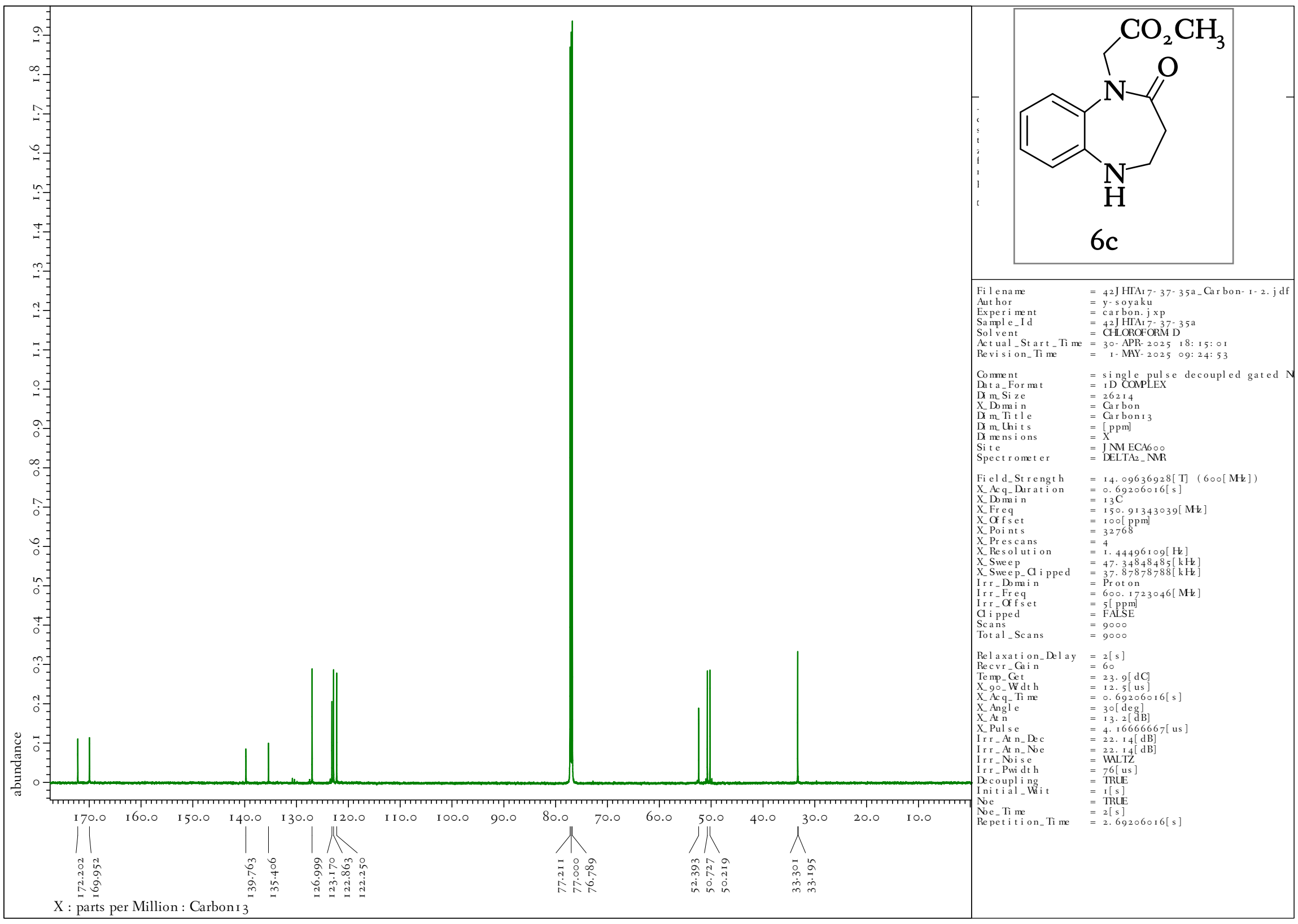

^{1}H-NMR ($CDCl_3$)

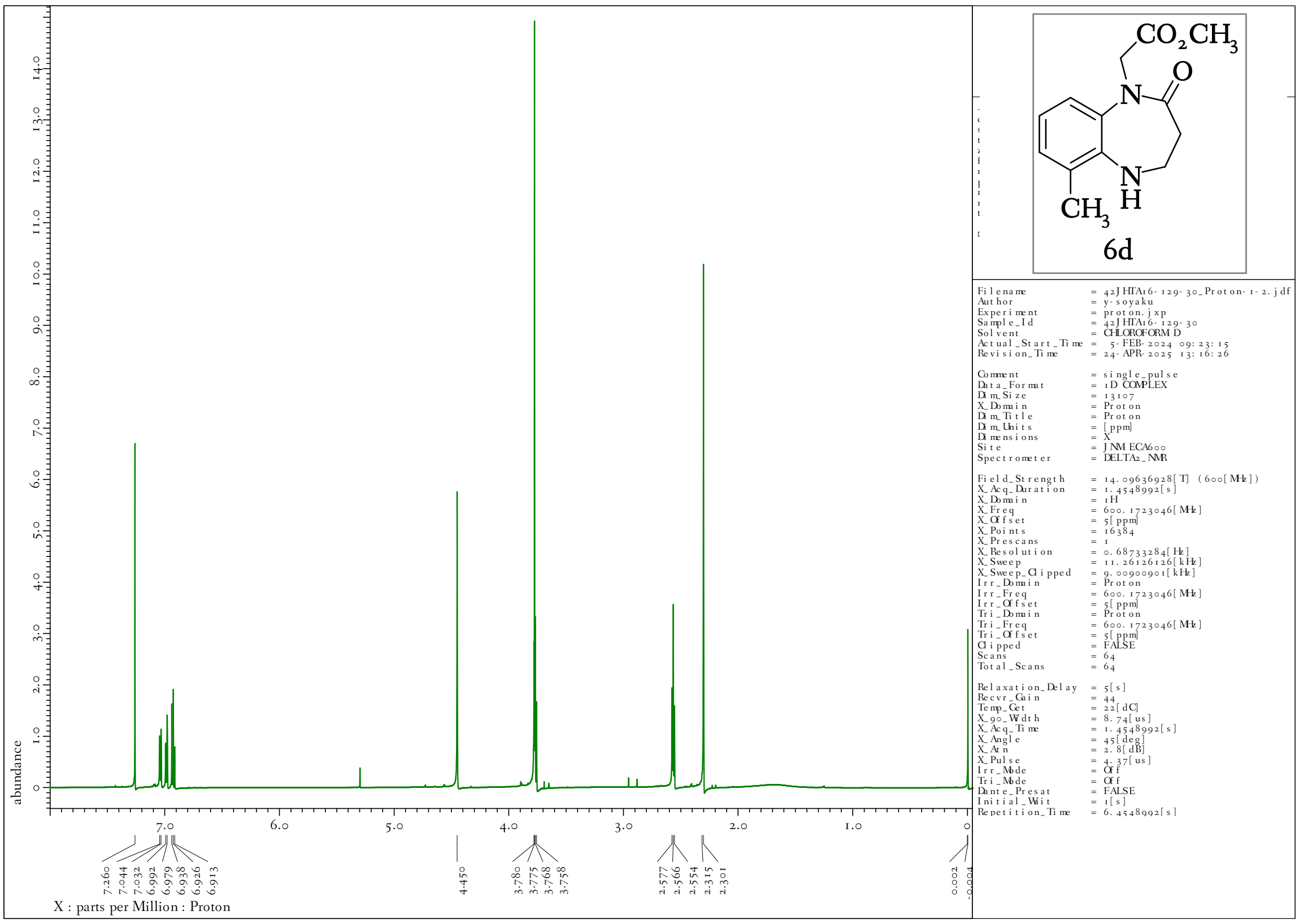

^{13}C-NMR ($CDCl_3$)

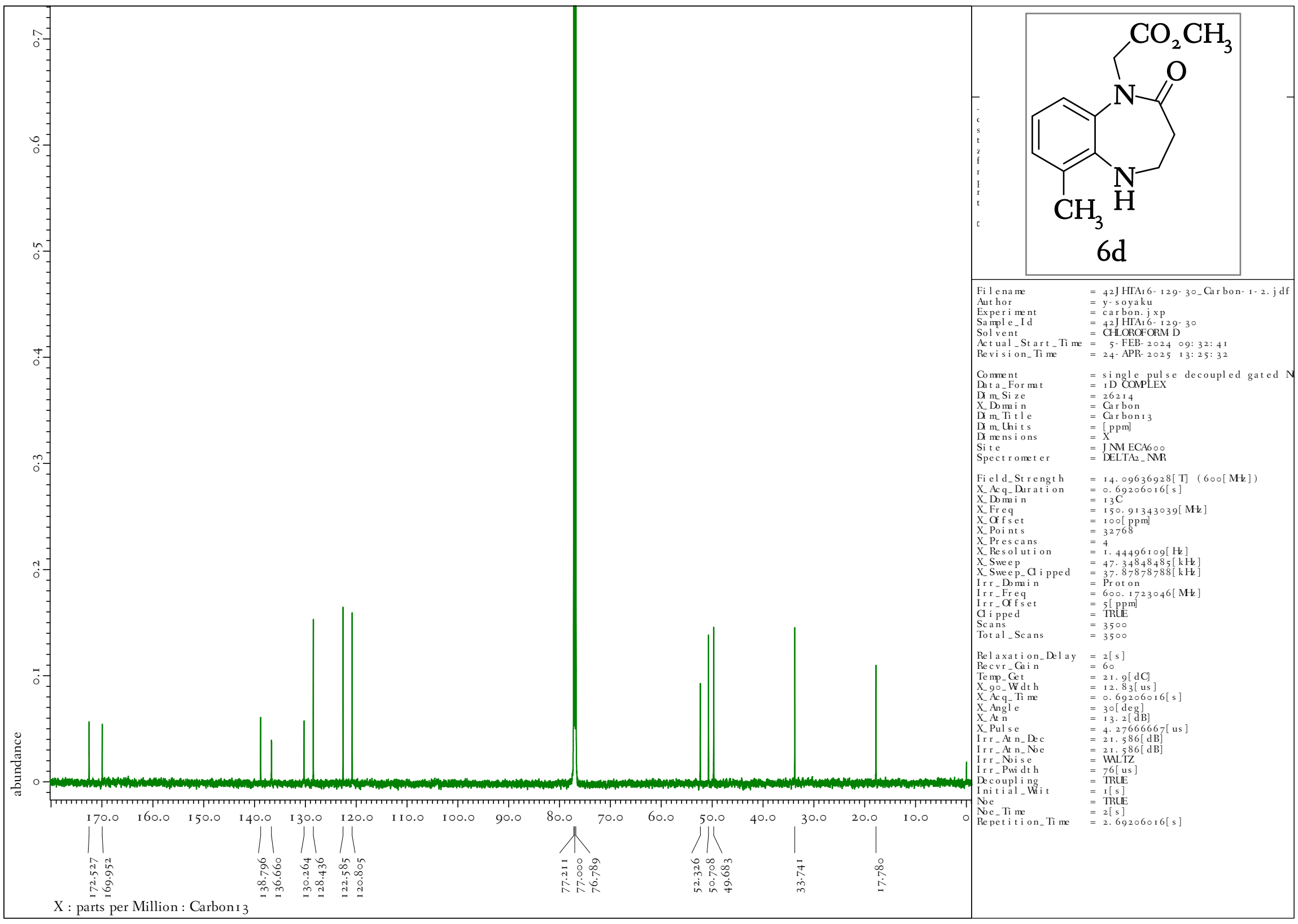

^{1}H-NMR ($CDCl_3$)

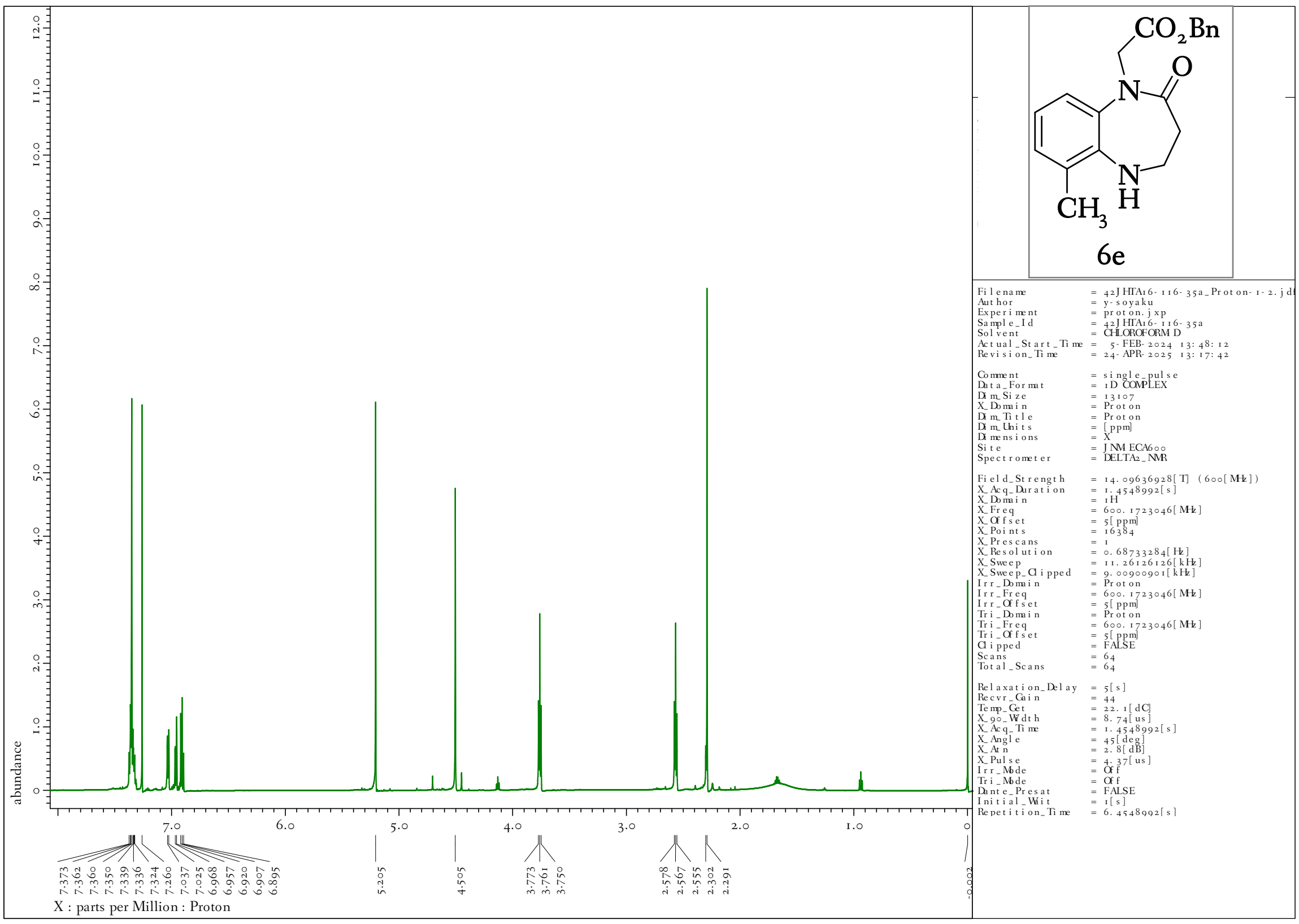

^{13}C-NMR ($CDCl_3$)

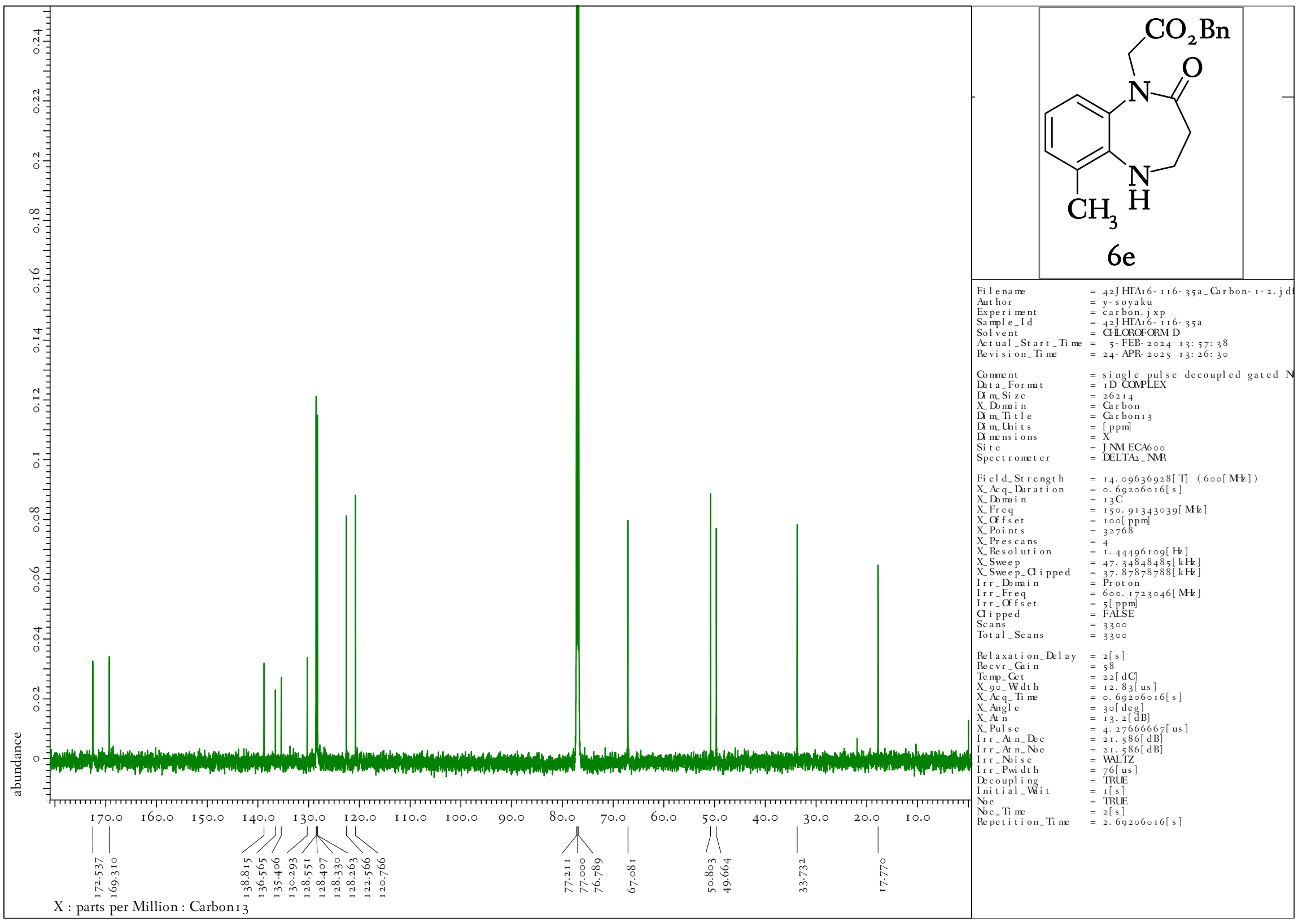

^{1}H-NMR ($CDCl_3$)

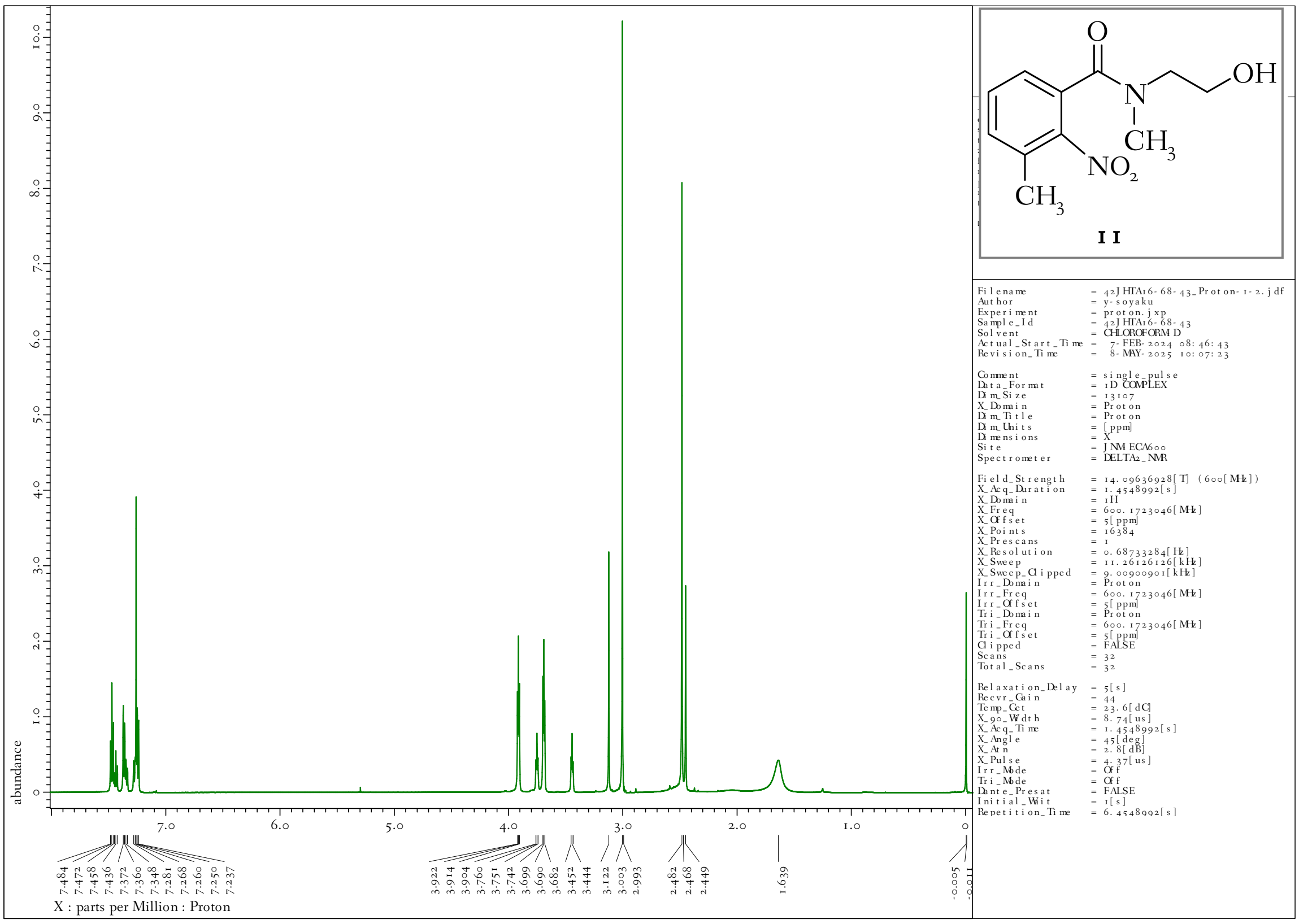

^{13}C-NMR ($CDCl_3$)

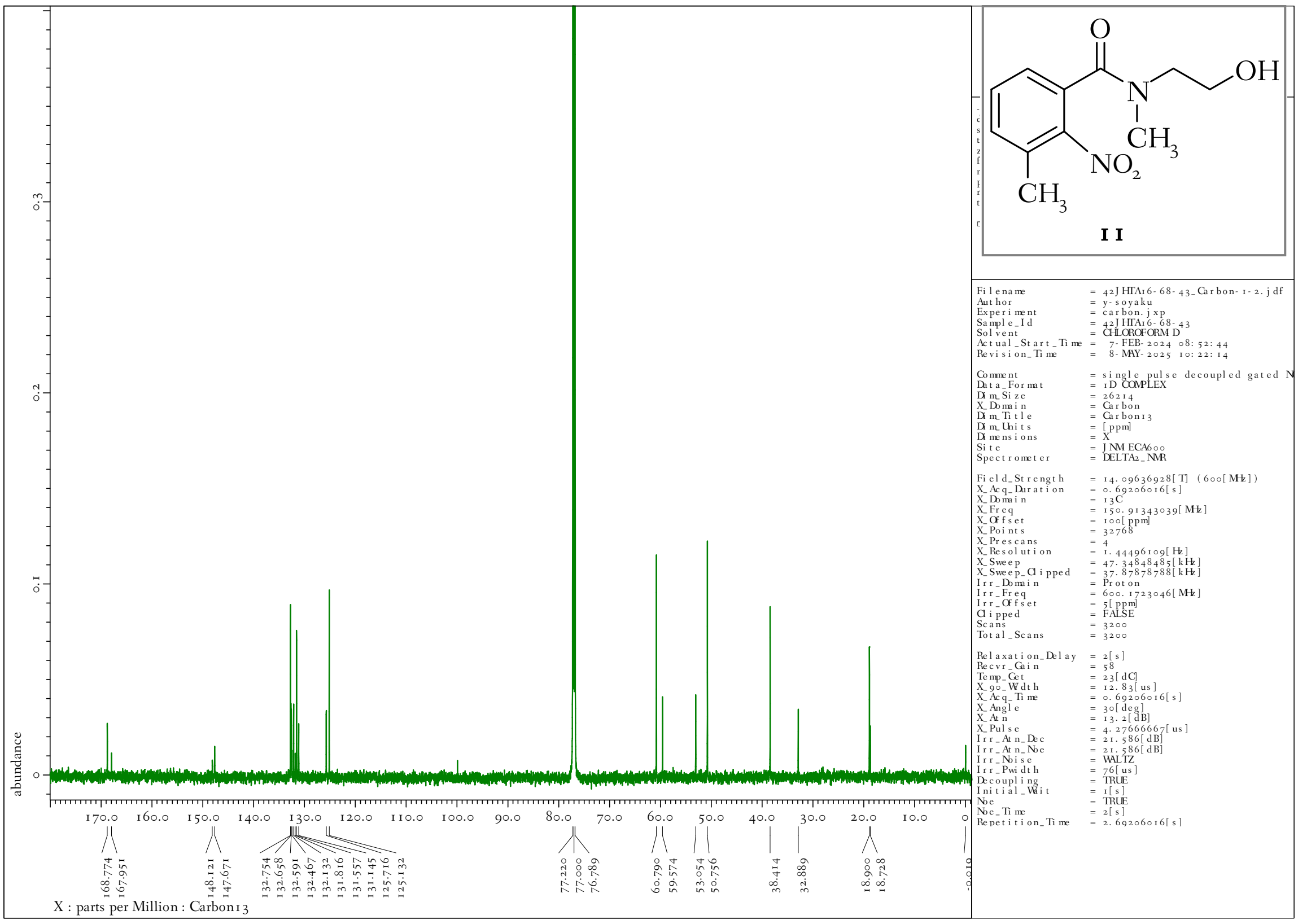

^{1}H-NMR (THF-d_8)

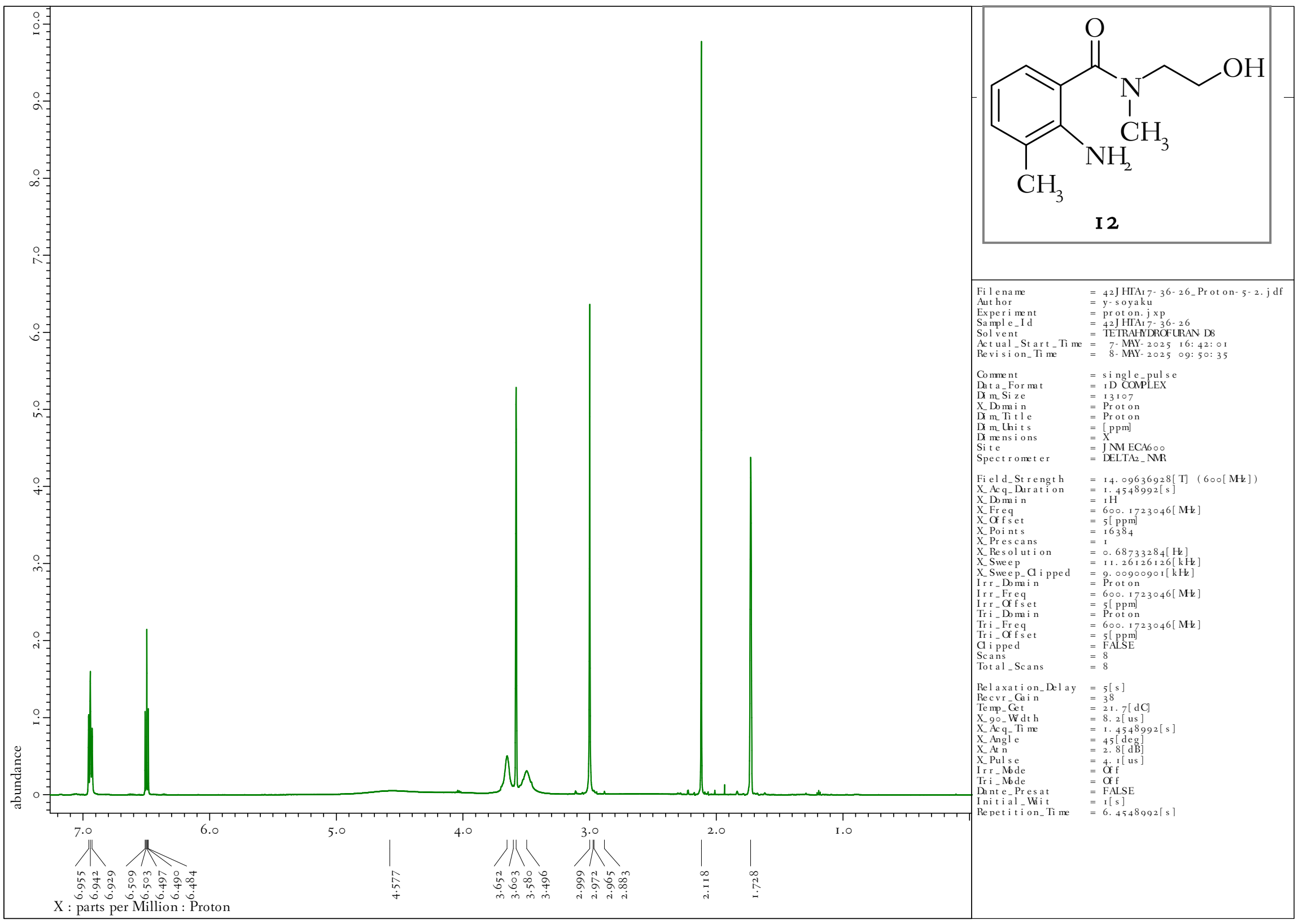

^{13}C-NMR (THF-d_8)

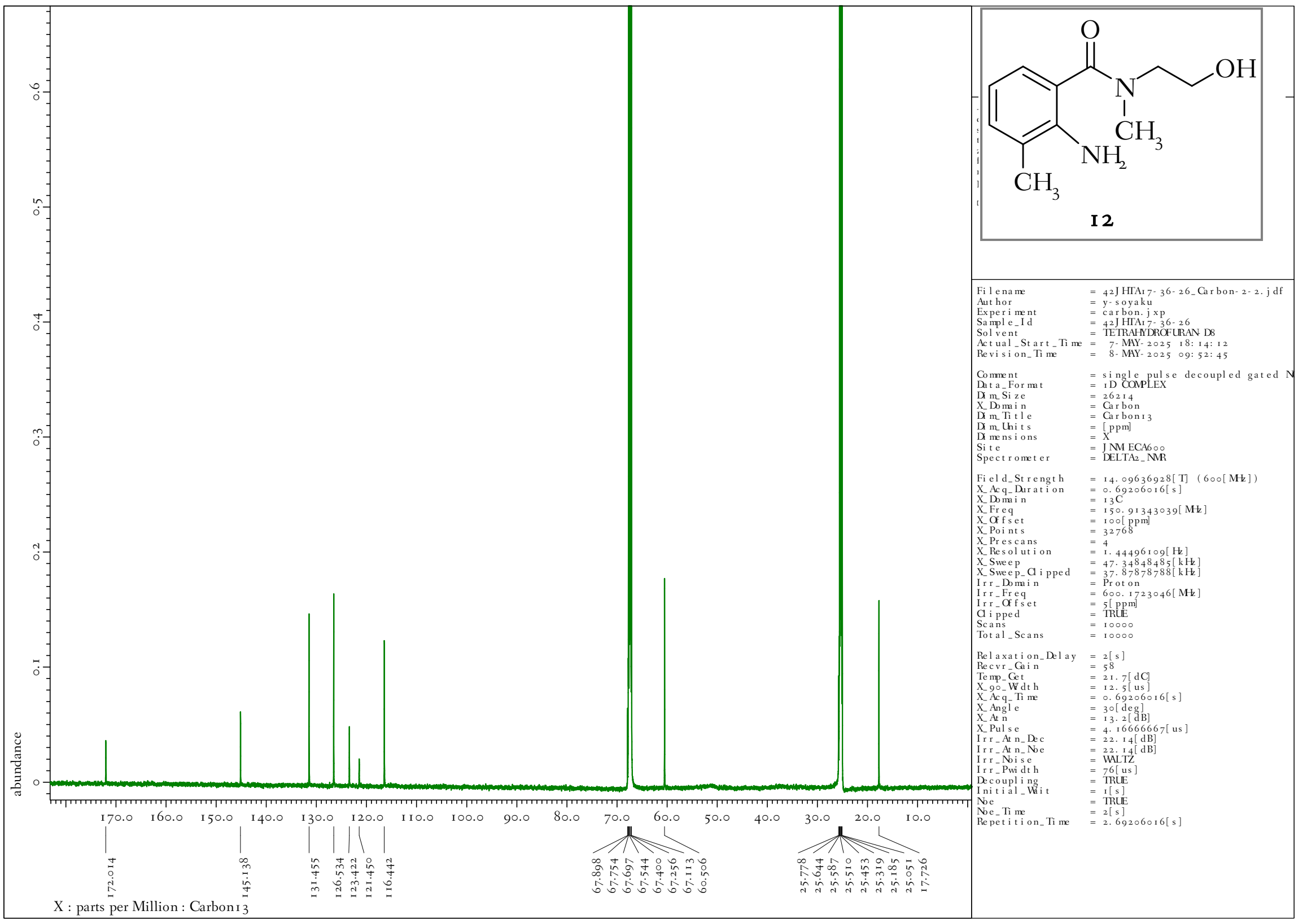

^{1}H-NMR ($CDCl_3$)

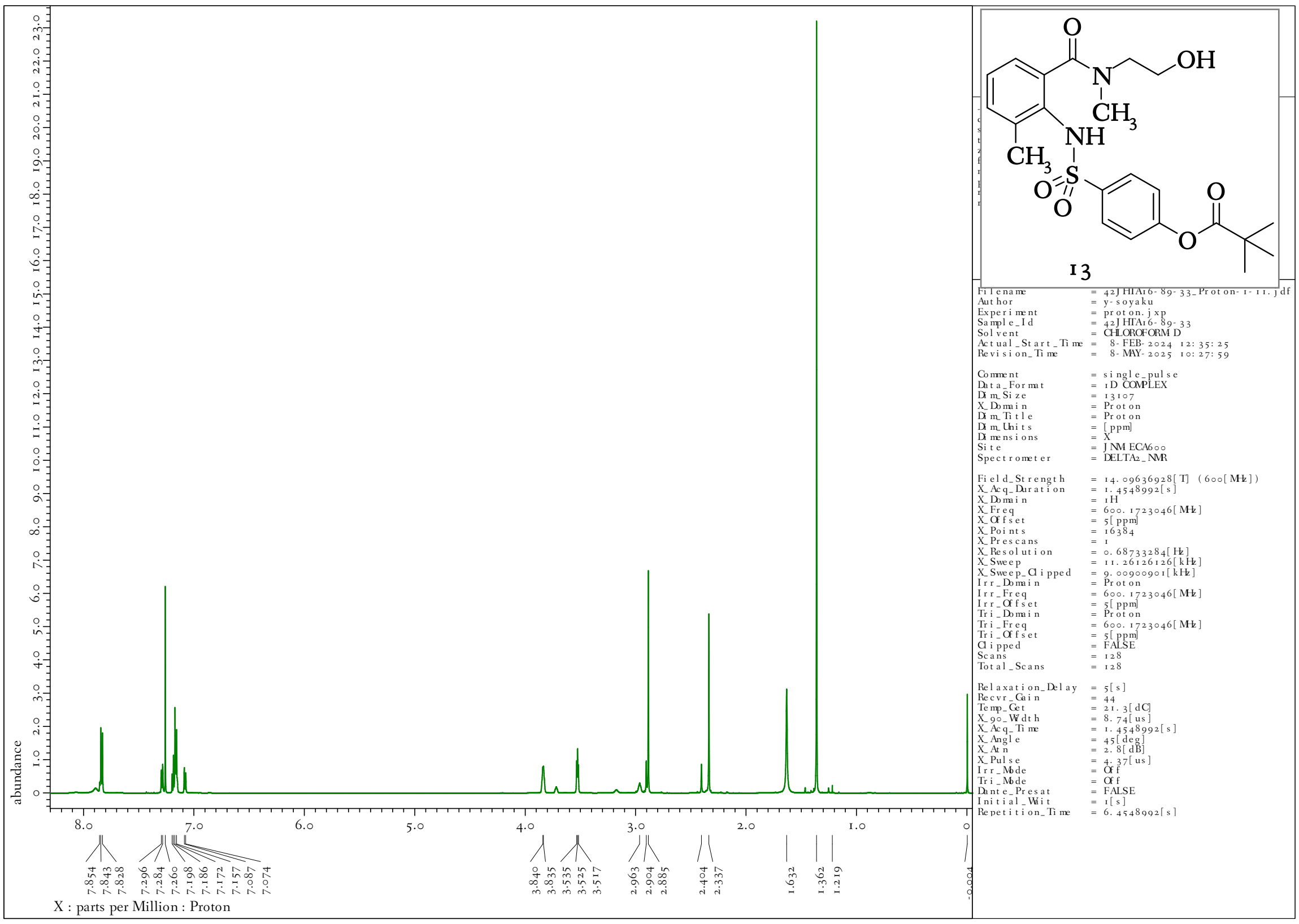

^{13}C-NMR ($CDCl_3$)

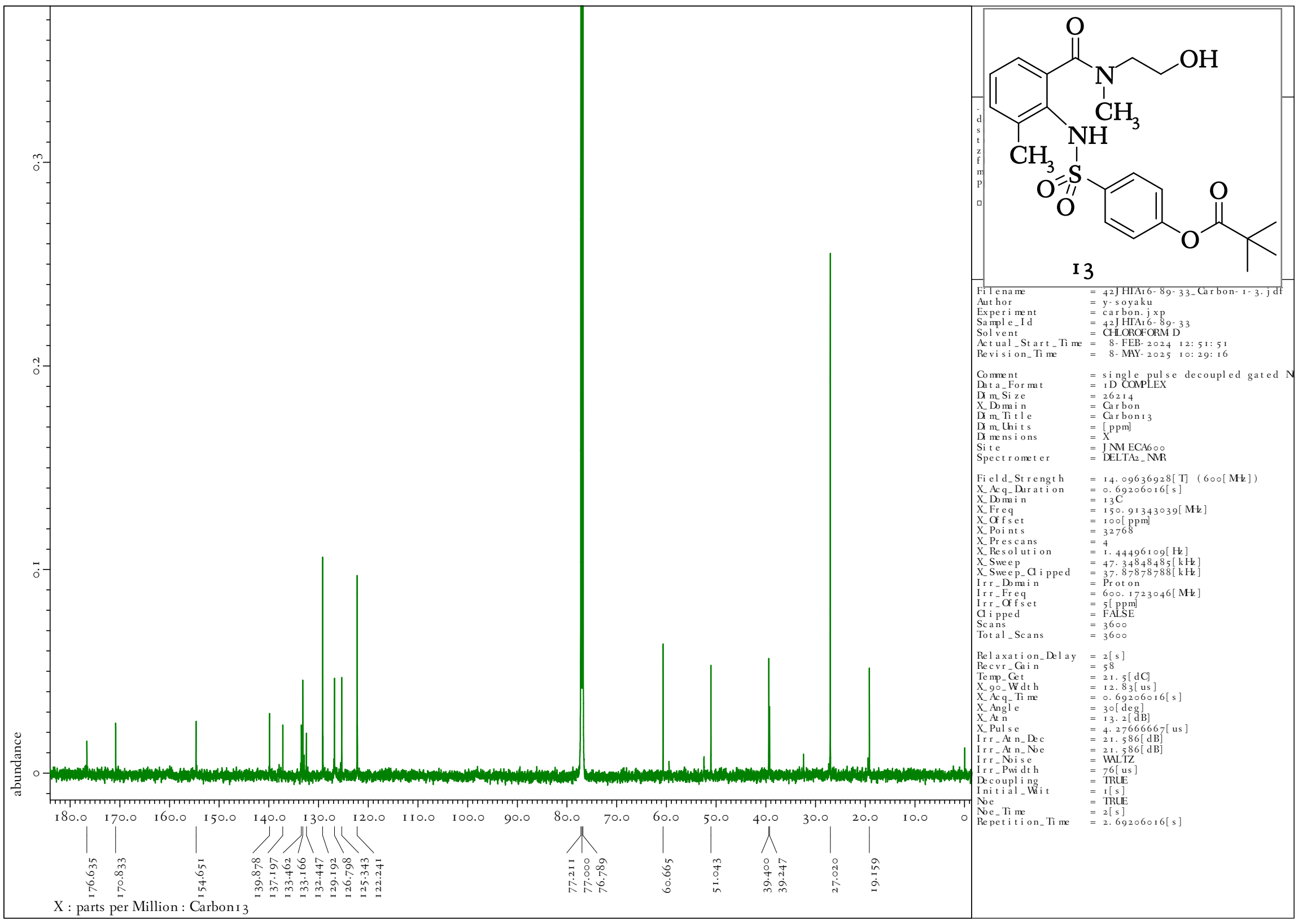

^{1}H-NMR ($CDCl_3$)

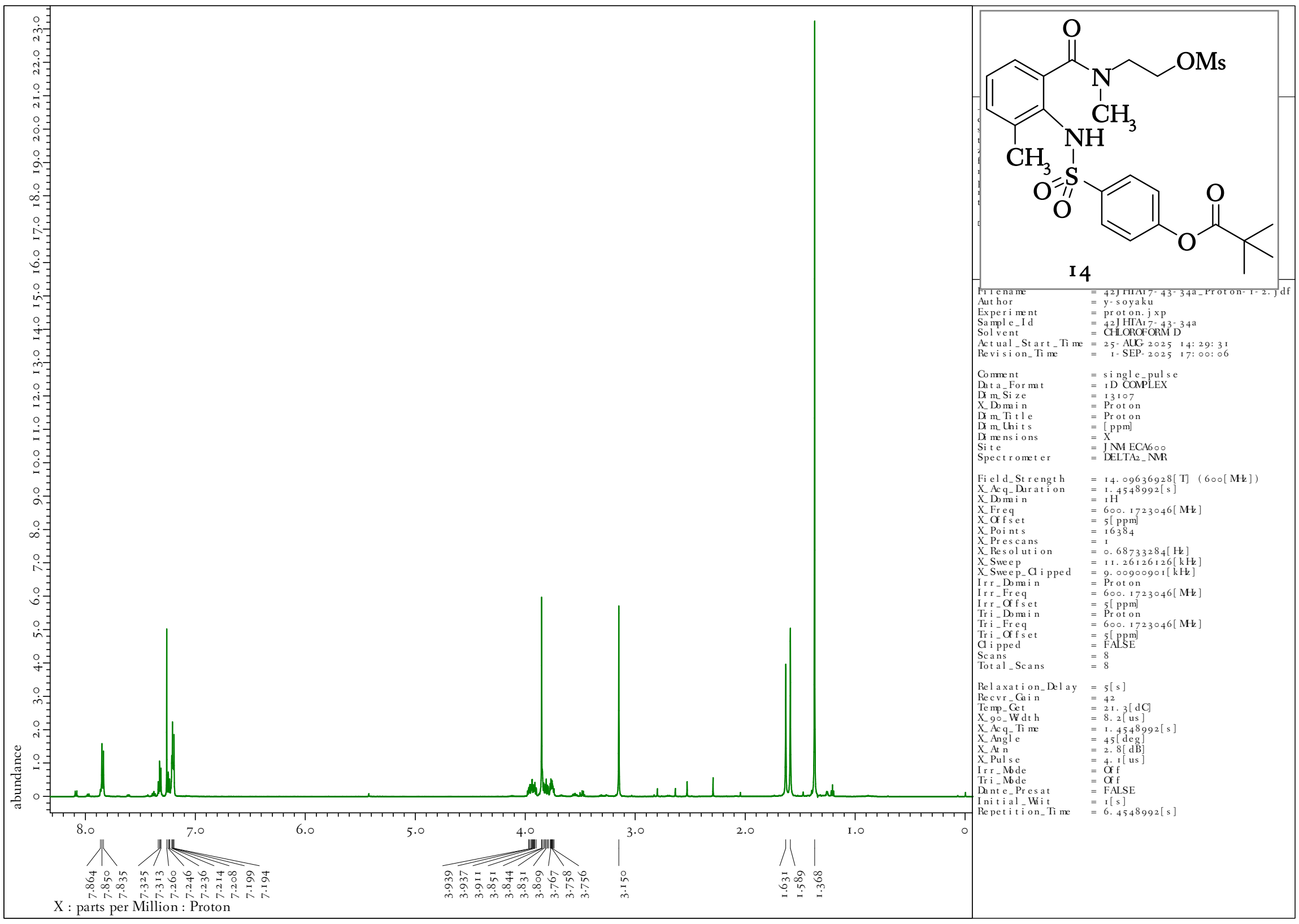

^{13}C-NMR ($CDCl_3$)

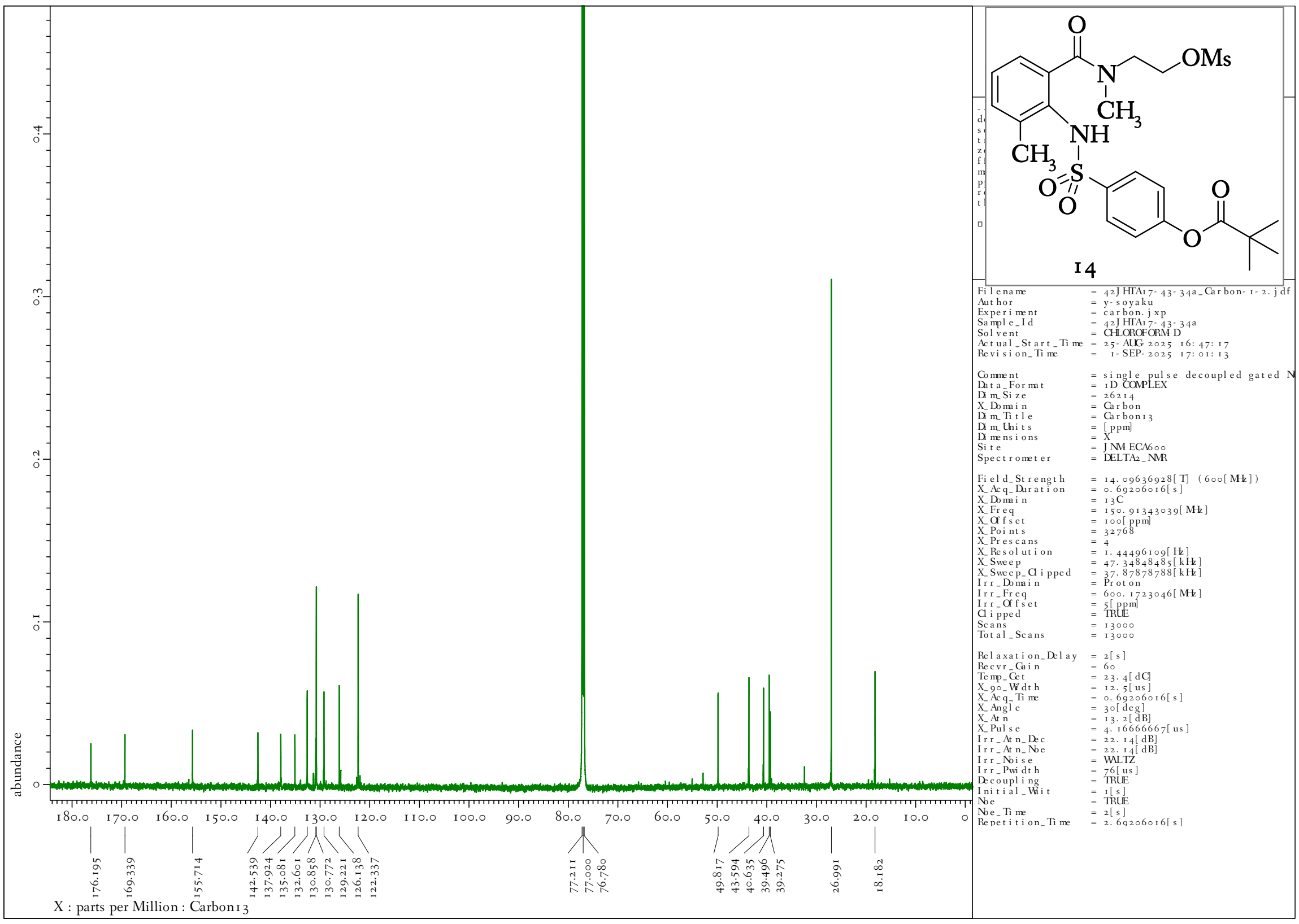

8. HRMS data

1-Methyl-5-(*p*-pivaloyloxybenzenesulfonyl)-1,3,4,5-tetrahydro-2*H*-1,5-benzodiazepin-2-one (2a).

Data File: C:¥LabSolutions¥Data¥TeikyoData¥創薬¥42J¥HTA16-133-36.lcd

Elmt	Val.	Min	Max	Elmt	Val.	Min	Max	Elmt	Val.	Min	Max	Elmt	Val.	Min	Max	Use Adduct
H	1	10	40	N	3	2	3	P	3	0	0	Br	1	0	0	H
2H	1	0	0	O	2	2	6	S	2	1	2	Pd	2	0	0	Na
B	3	0	0	F	1	0	0	Cl	1	0	0	I	3	0	0	K
C	4	19	26	Si	4	0	0	Cu	2	0	0	Pt	2	0	0	

Error Margin (ppm):	1000	DBE Range:	not fixed	Electron Ions:	both
HC Ratio:	unlimited	Apply N Rule:	no	Use MSn Info:	no
Max Isotopes:	all	Isotope RI (%):	1.00	Isotope Res:	10000
MSn Iso RI (%):	75.00	MSn Logic Mode:	AND	Max Results:	40

ｲﾍﾞﾝﾄ#: 1 MS(E+)　保持時間: 1.547 -> 1.547 - 1.747 -> 1.770　ｽｷｬﾝ番号: 83 -> 83 - 113 -> 117

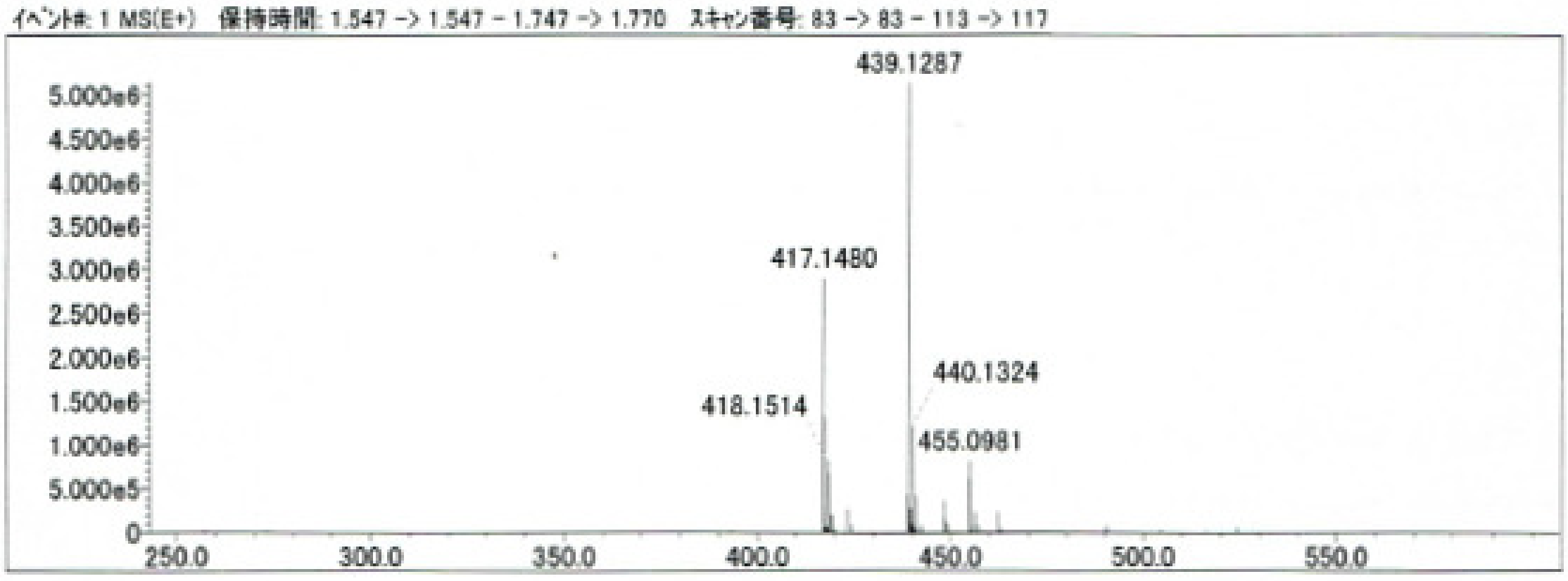

Measured region for 417.1480 m/z

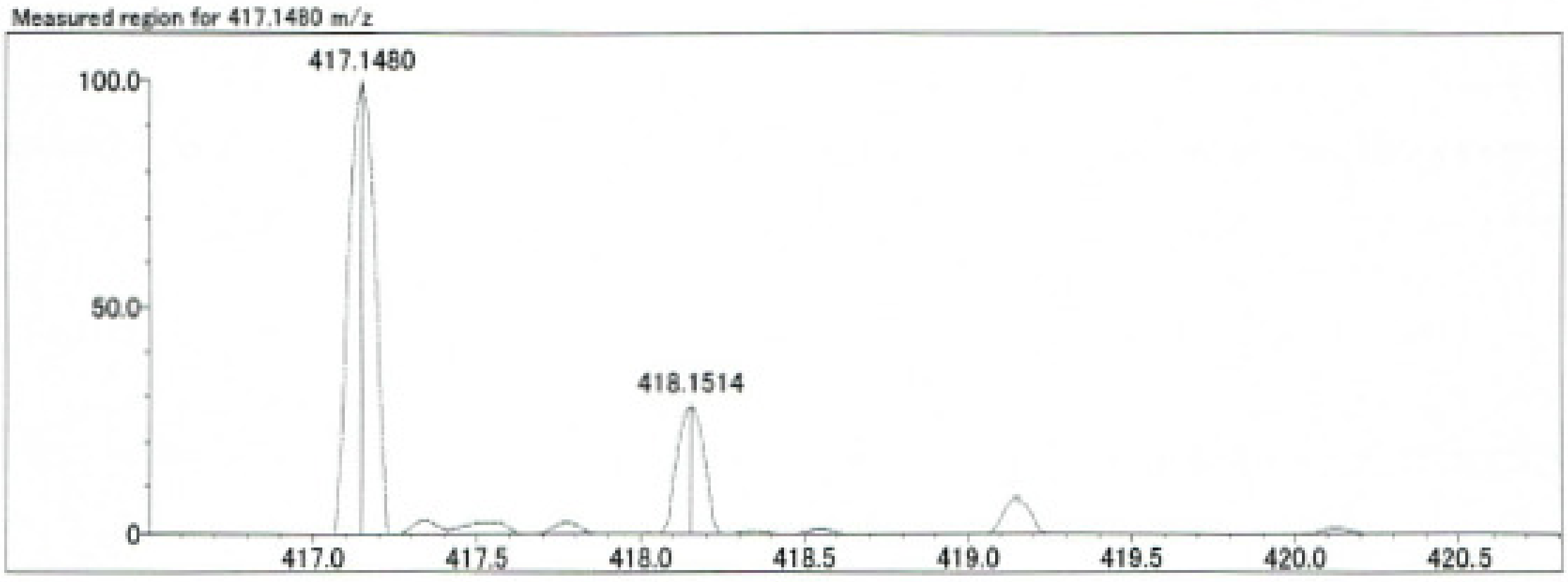

C21 H24 N2 O5 S [M+H]+ : Predicted region for 417.1479 m/z

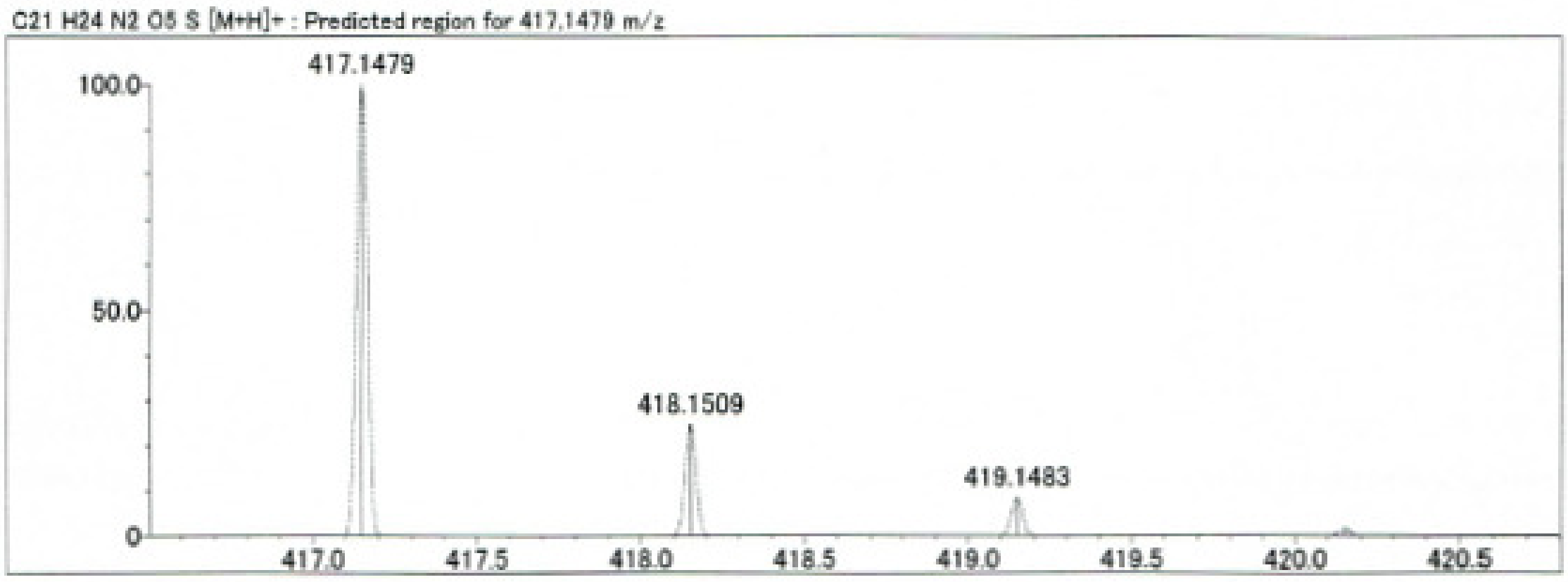

Rank	Score	Formula (M)	Ion	Meas. m/z	Pred. m/z	Df. (mDa)	Df. (ppm)	Iso	DBE
2	72.34	C21 H24 N2 O5 S	[M+H]+	417.1480	417.1479	0.1	0.24	72.34	11.0

1,6-Dimethyl-5-(*p*-pivaloyloxybenzenesulfonyl)-1,3,4,5-tetrahydro-2*H*-1,5-benzodiazepin-2-one (2b).

Data File: C:¥LabSolutions¥Data¥TeikyoData¥創薬¥42J¥HTA16-95-34.lcd

Elmt	Val.	Min	Max	Elmt	Val.	Min	Max	Elmt	Val.	Min	Max	Elmt	Val.	Min	Max	Use Adduct
H	1	10	40	N	3	2	3	P	3	0	0	Br	1	0	0	H
2H	1	0	0	O	2	2	6	S	2	1	2	Pd	2	0	0	Na
B	3	0	0	F	1	0	0	Cl	1	0	0	I	3	0	0	K
C	4	19	26	Si	4	0	0	Cu	2	0	0	Pt	2	0	0	

Error Margin (ppm): 1000
HC Ratio: unlimited
Max Isotopes: all
MSn Iso RI (%): 75.00

DBE Range: not fixed
Apply N Rule: no
Isotope RI (%): 1.00
MSn Logic Mode: AND

Electron Ions: both
Use MSn Info: no
Isotope Res: 10000
Max Results: 40

イベント#: 1 MS(E+)　保持時間: 1.307 -> 1.320　スキャン番号: 47 -> 49

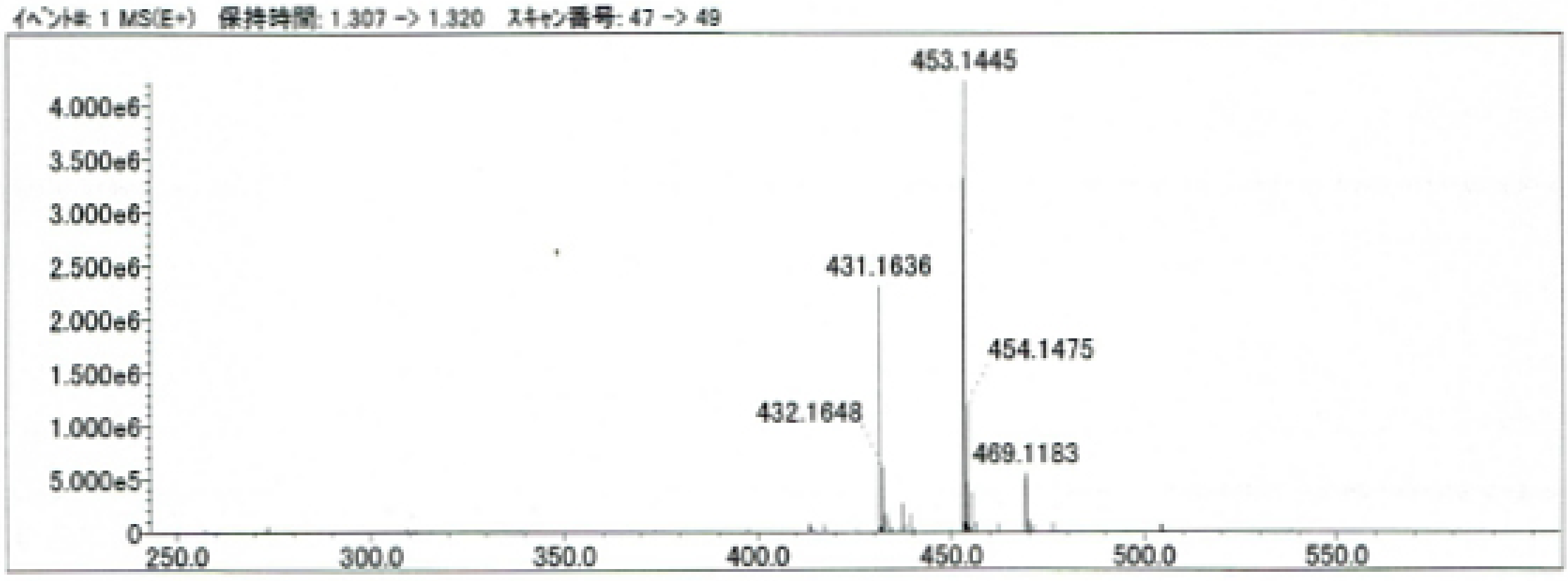

Measured region for 431.1636 m/z

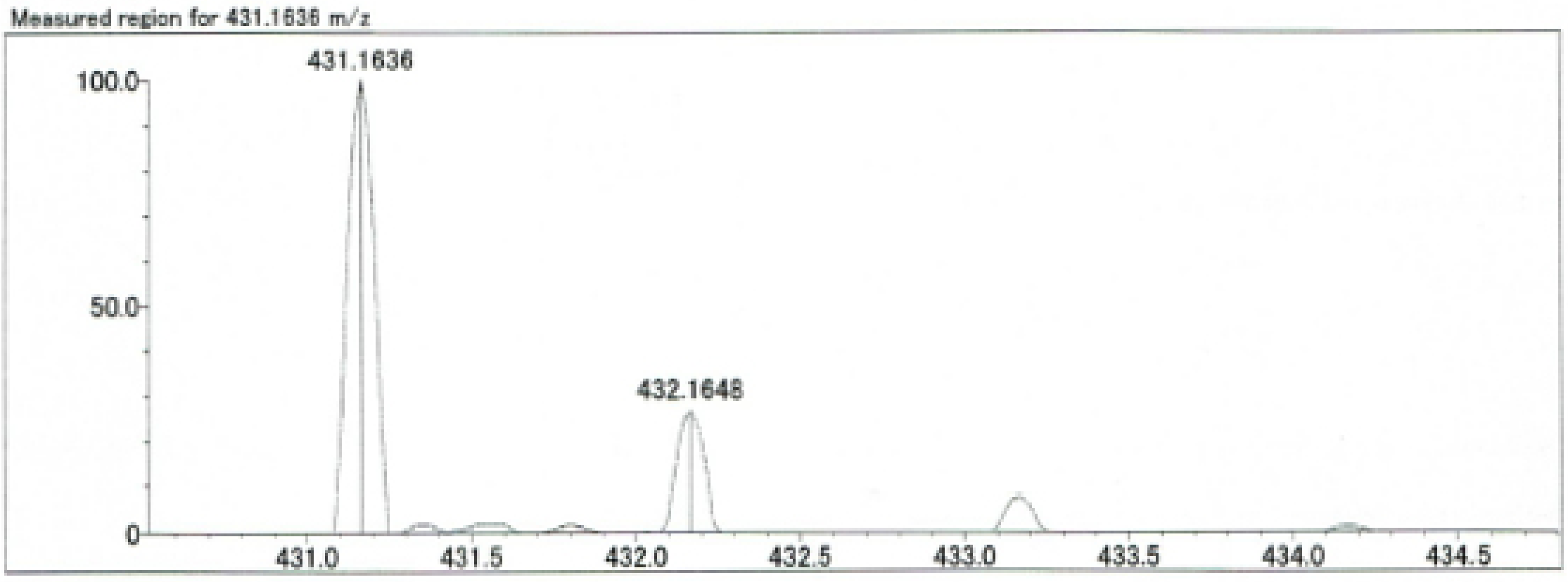

C22 H26 N2 O5 S [M+H]+ : Predicted region for 431.1635 m/z

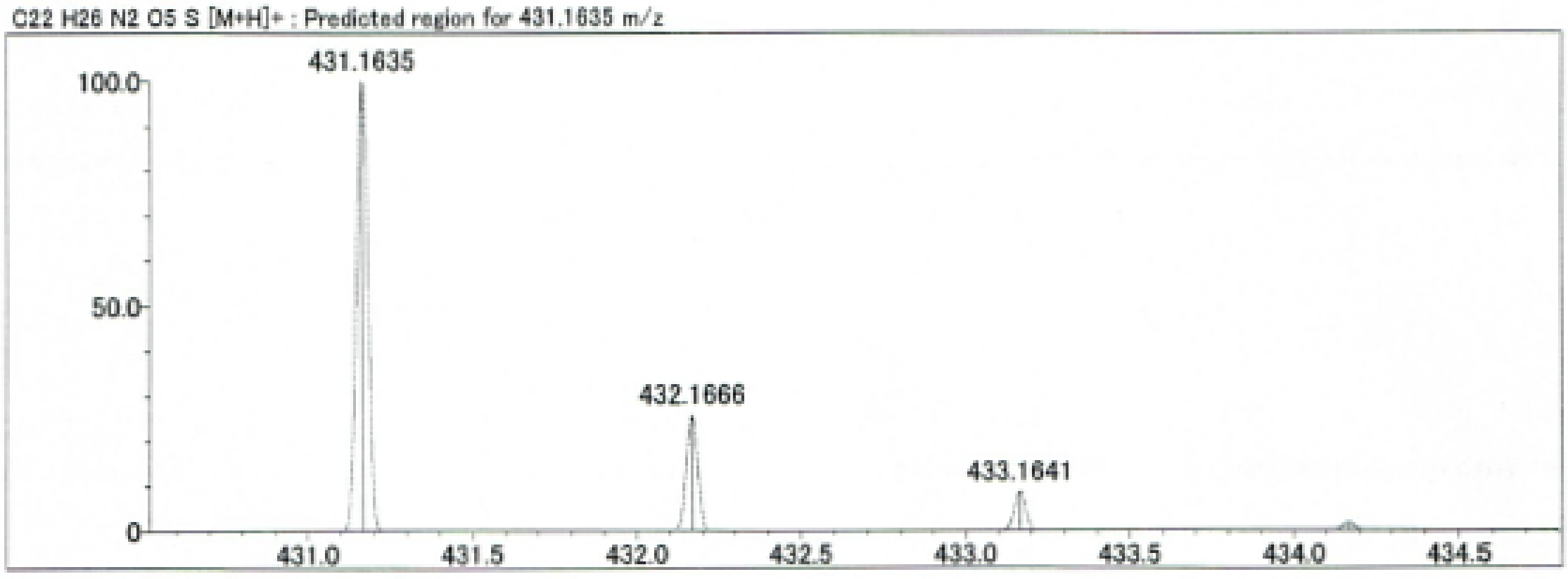

Rank	Score	Formula (M)	Ion	Meas. m/z	Pred. m/z	Df. (mDa)	Df. (ppm)	Iso	DBE
2	86.07	C22 H26 N2 O5 S	[M+H]+	431.1636	431.1635	0.1	0.23	86.07	11.0

Methyl 5-(*p*-pivaloyloxybenzenesulfonyl)-2,3,4,5-tetrahydro-2-oxo-1*H*-1,5-benzodiazepine-1-acetate (2c).

Data File: C:¥LabSolutions¥Data¥TeikyoData¥創薬¥42J¥HTA16-147-35.lcd

Elmt	Val.	Min	Max	Elmt	Val.	Min	Max	Elmt	Val.	Min	Max	Elmt	Val.	Min	Max	Use Adduct
H	1	0	30	N	3	0	3	P	3	0	0	Br	1	0	0	H
2H	1	0	0	O	2	0	8	S	2	0	1	Pd	2	0	0	Na
B	3	0	0	F	1	0	0	Cl	1	0	0	I	3	0	0	K
C	4	0	25	Si	4	0	0	Cu	2	0	0	Pt	2	0	0	

Error Margin (ppm): 50
HC Ratio: unlimited
Max Isotopes: all
MSn Iso RI (%): 75.00

DBE Range: not fixed
Apply N Rule: no
Isotope RI (%): 1.00
MSn Logic Mode: AND

Electron Ions: both
Use MSn Info: no
Isotope Res: 10000
Max Results: 40

ｲﾍﾞﾝﾄ#: 1 MS(E+) 保持時間: 1.453 -> 1.453 - 1.560 -> 1.562 ｽｷｬﾝ番号: 69 -> 69 - 85 -> 85

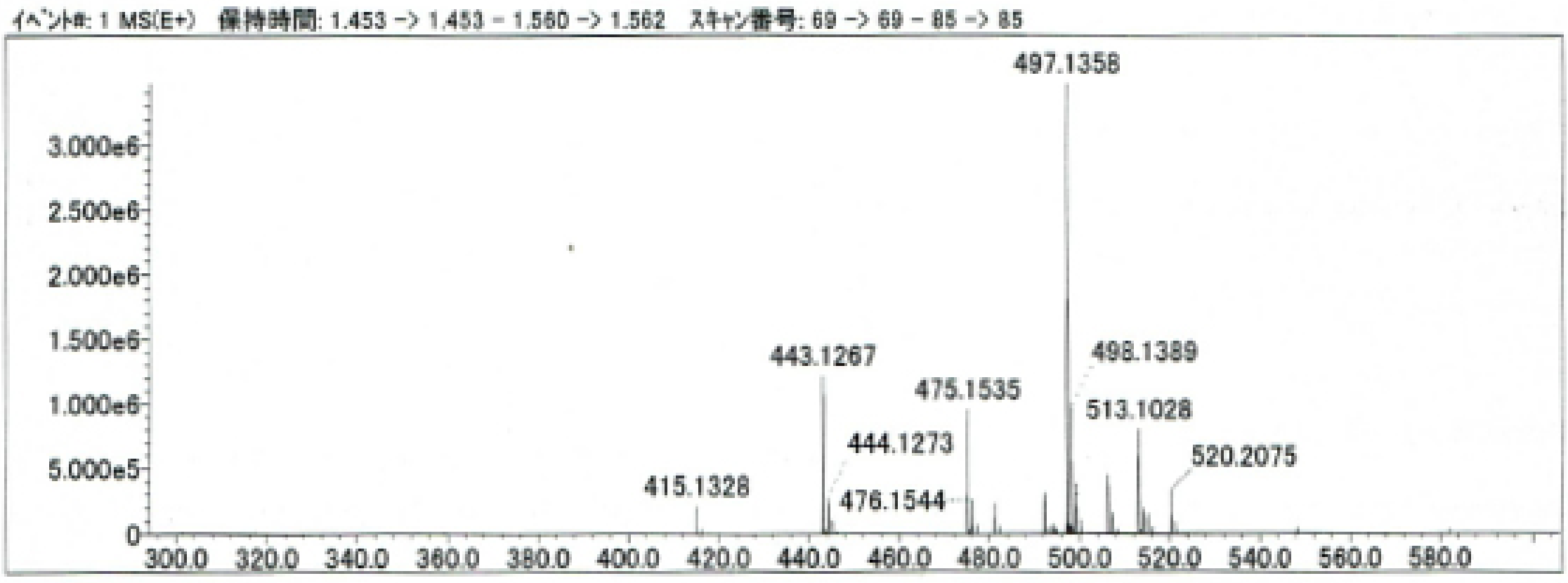

Measured region for 475.1535 m/z

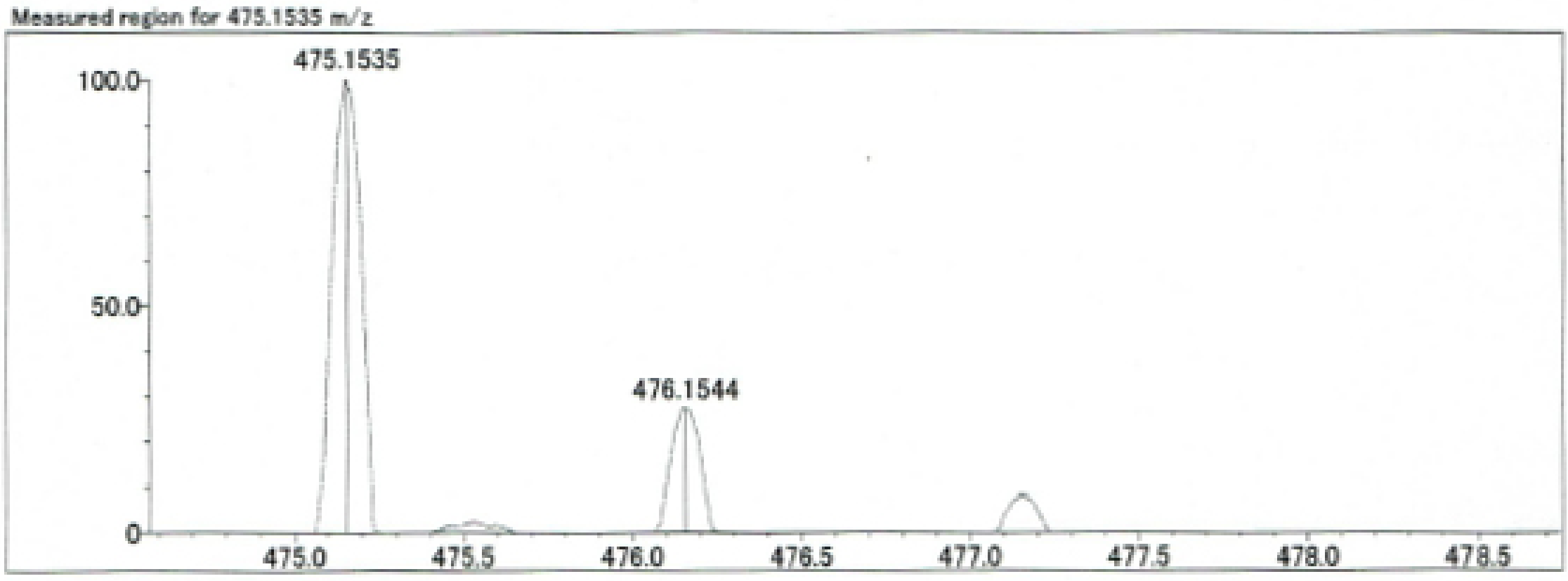

C23 H26 N2 O7 S [M+H]+ : Predicted region for 475.1533 m/z

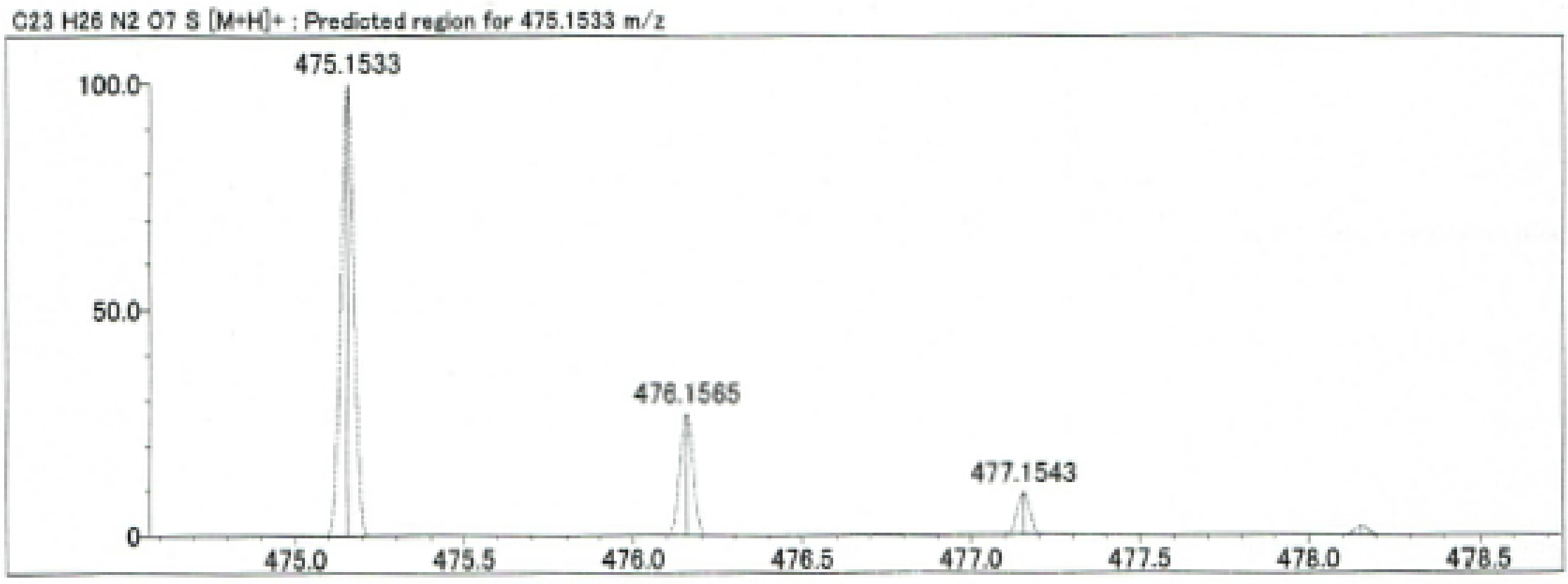

Rank	Score	Formula (M)	Ion	Meas. m/z	Pred. m/z	Df. (mDa)	Df. (ppm)	Iso	DBE
2	100.00	C23 H26 N2 O7 S	[M+H]+	475.1535	475.1533	0.2	0.42	100.00	12.0

Methyl 5-(*p*-pivaloyloxybenzenesulfonyl)-6-methyl-2,3,4,5-tetrahydro-2-oxo-1*H*-1,5-benzodiazepine-1-acetate (2d).

Data File: C:¥LabSolutions¥Data¥TeikyoData¥創薬¥42J¥HTA16-132-34.lcd

Elmt	Val.	Min	Max	Elmt	Val.	Min	Max	Elmt	Val.	Min	Max	Elmt	Val.	Min	Max	Use Adduct
H	1	10	40	N	3	2	3	P	3	0	0	Br	1	0	0	H
2H	1	0	0	O	2	2	7	S	2	1	2	Pd	2	0	0	Na
B	3	0	0	F	1	0	0	Cl	1	0	0	I	3	0	0	K
C	4	19	26	Si	4	0	0	Cu	2	0	0	Pt	2	0	0	

Error Margin (ppm):	1000	DBE Range:	not fixed	Electron Ions:	both
HC Ratio:	unlimited	Apply N Rule:	no	Use MSn Info:	no
Max Isotopes:	all	Isotope RI (%):	1.00	Isotope Res:	10000
MSn Iso RI (%):	75.00	MSn Logic Mode:	AND	Max Results:	40

イベント#: 1 MS(E+) 保持時間: 1.320 -> 1.320 - 1.560 -> 1.569 スキャン番号: 49 -> 49 - 85 -> 87

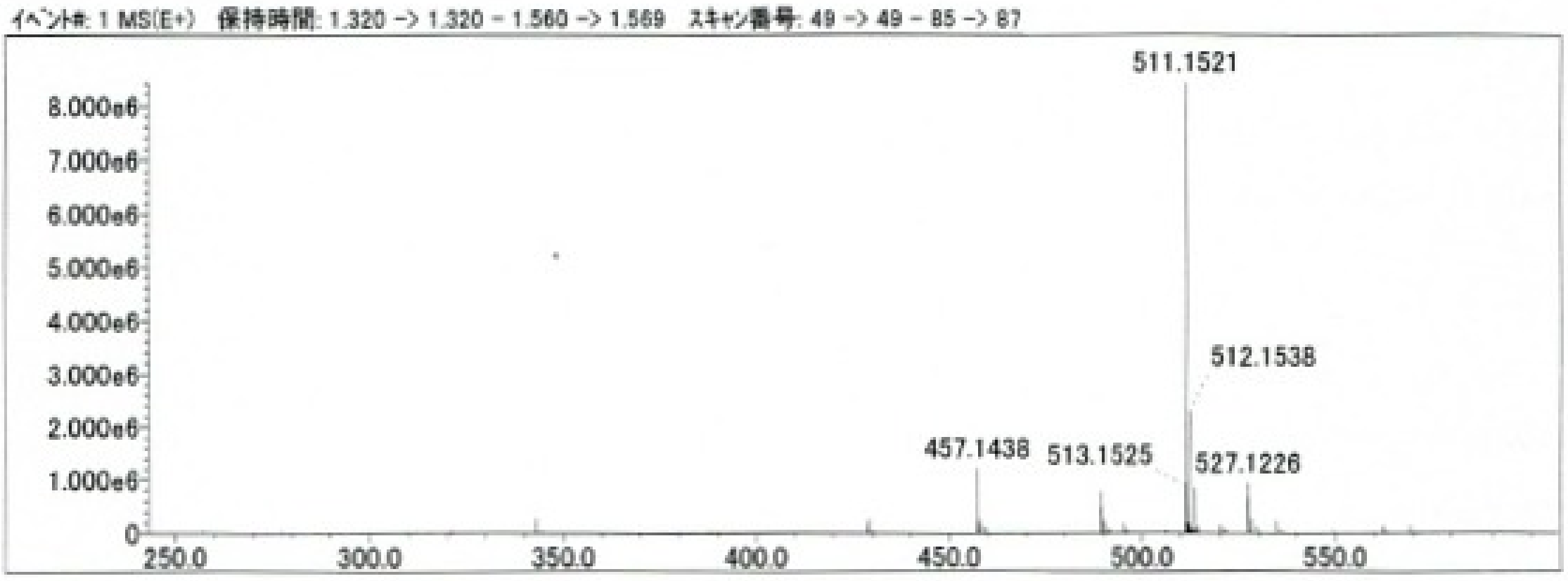

Measured region for 489.1692 m/z

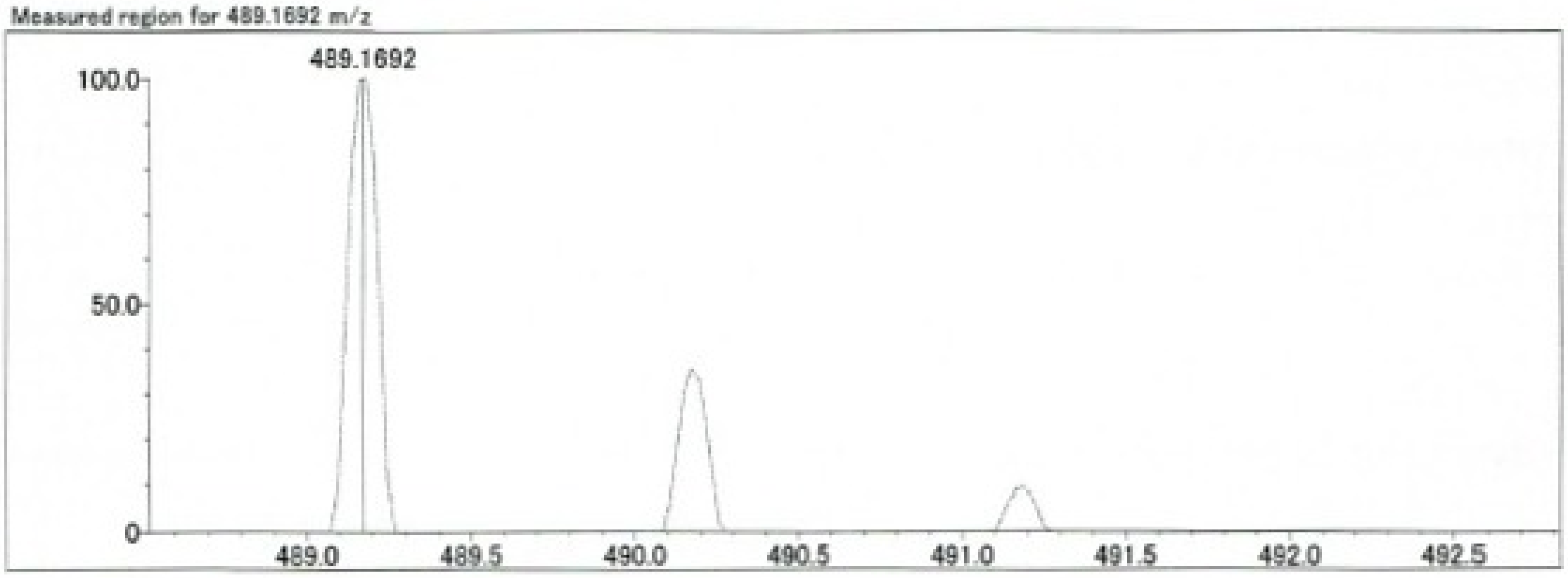

C24 H28 N2 O7 S [M+H]+ : Predicted region for 489.1690 m/z

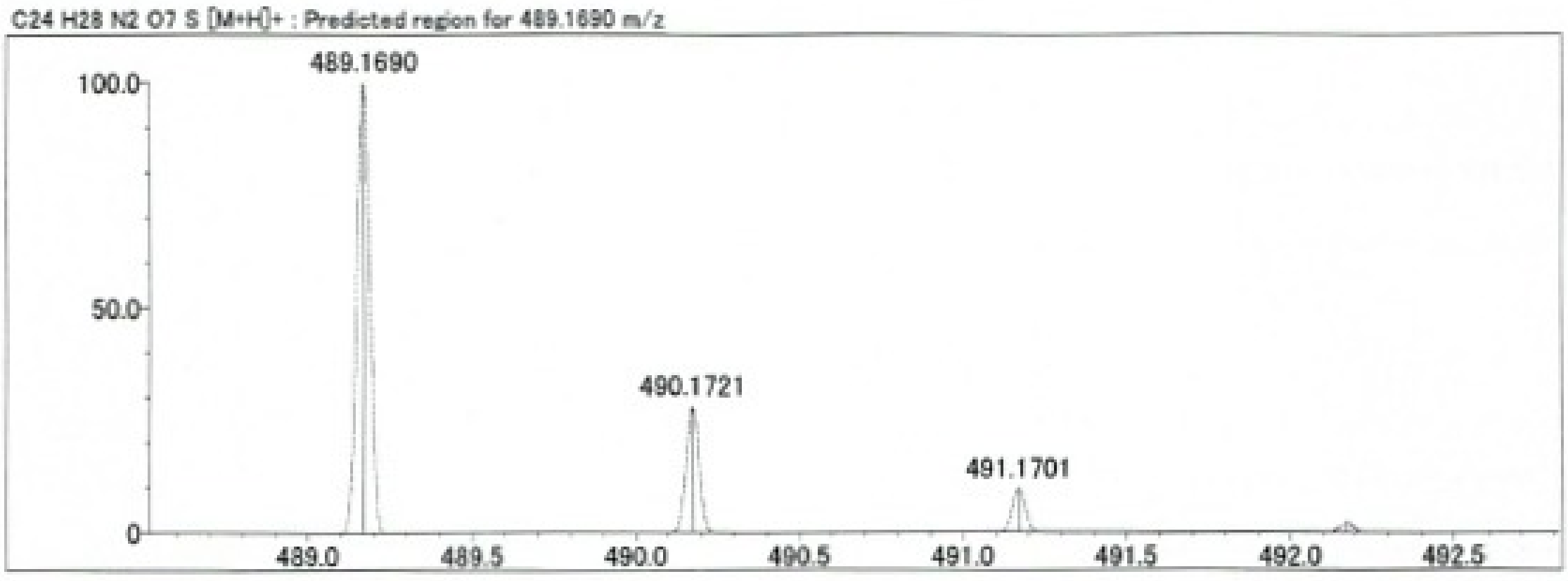

Rank	Score	Formula (M)	Ion	Meas. m/z	Pred. m/z	Df. (mDa)	Df. (ppm)	Iso	DBE
2	71.65	C24 H28 N2 O7 S	[M+H]+	489.1692	489.1690	0.2	0.41	71.65	12.0

Benzyl 5-(*p*-pivaloyloxybenzenesulfonyl)-6-methyl-2,3,4,5-tetrahydro-2-oxo-1*H*-1,5-benzodiazepine-1-acetate (2e).

Data File: C:¥LabSolutions¥Data¥TeikyoData¥創薬¥42J¥HTA16-134-37a.lcd

Elmt	Val.	Min	Max	Elmt	Val.	Min	Max	Elmt	Val.	Min	Max	Elmt	Val.	Min	Max	Use Adduct
H	1	17	40	N	3	2	3	P	3	0	0	Br	1	0	1	H
2H	1	0	0	O	2	2	7	S	2	0	1	Pd	2	0	0	Na
B	3	0	0	F	1	0	0	Cl	1	0	1	I	3	0	0	K
C	4	18	32	Si	4	0	0	Cu	2	0	0	Pt	2	0	0	

Error Margin (ppm): 50
HC Ratio: unlimited
Max Isotopes: all
MSn Iso RI (%): 75.00

DBE Range: not fixed
Apply N Rule: no
Isotope RI (%): 1.00
MSn Logic Mode: AND

Electron Ions: both
Use MSn Info: no
Isotope Res: 10000
Max Results: 40

ｲﾍﾞﾝﾄ#: 1 MS(E+) 保持時間: 1.133 -> 1.133 ｽｷｬﾝ番号: 21 -> 21

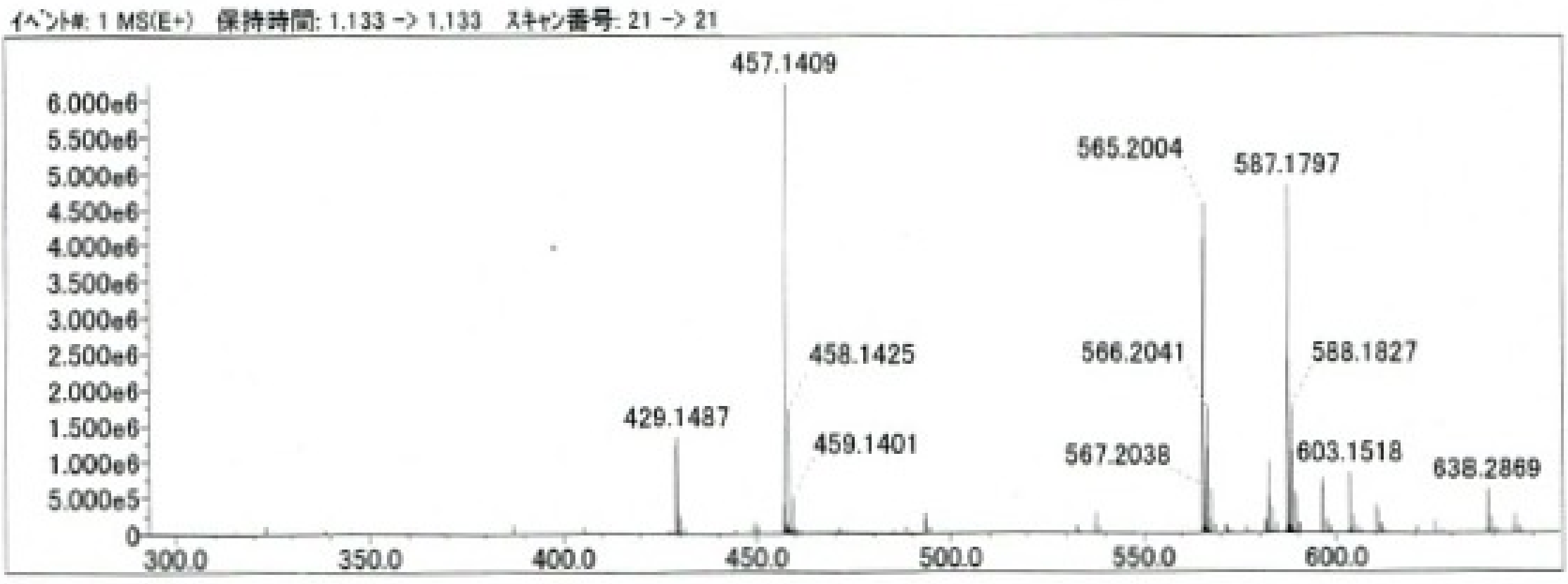

Measured region for 565.2004 m/z

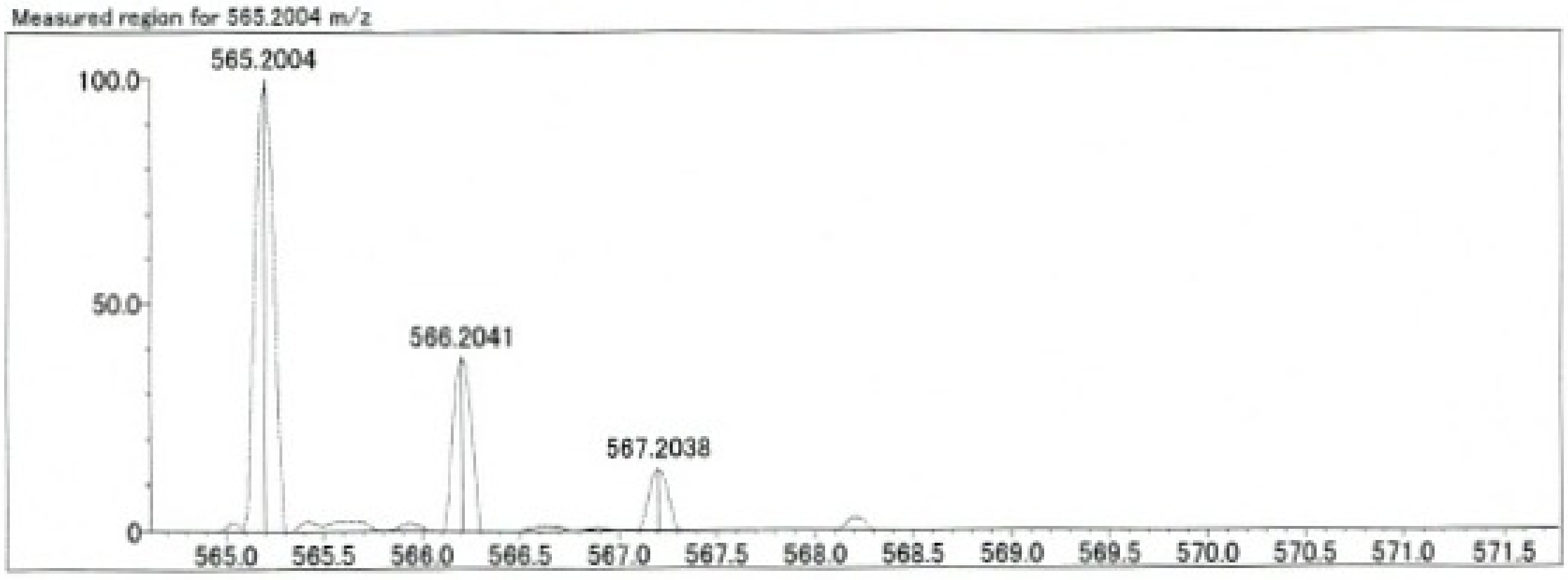

C30 H32 N2 O7 S [M+H]+ : Predicted region for 565.2003 m/z

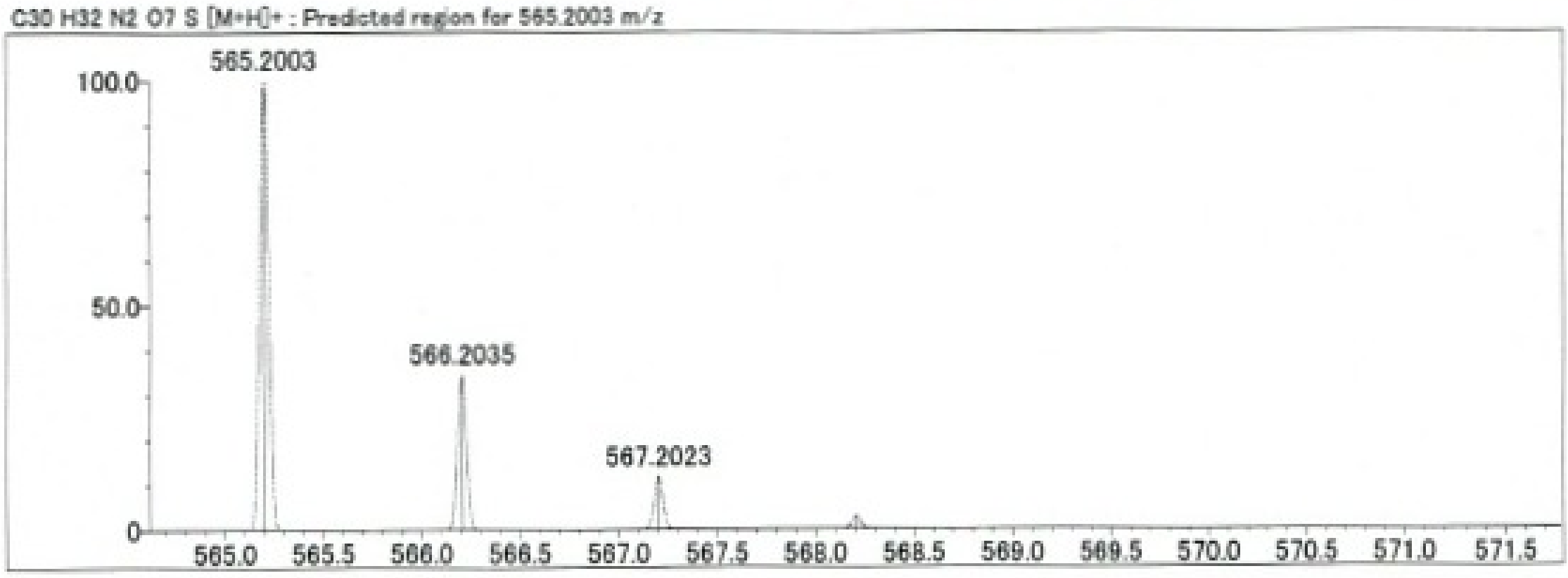

Rank	Score	Formula (M)	Ion	Meas. m/z	Pred. m/z	Df. (mDa)	Df. (ppm)	Iso	DBE
1	100.00	C30 H32 N2 O7 S	[M+H]+	565.2004	565.2003	0.1	0.18	100.00	16.0

5-(*p*-Pivaloyloxybenzenesulfonyl)-6-methyl-2,3,4,5-tetrahydro-2-oxo-1*H*-1,5-benzodiazepine-1-acetic acid (2f).

Data File: C:¥LabSolutions¥Data¥TeikyoData¥創薬¥42J¥HTA16-139-29.lcd

Elmt	Val.	Min	Max	Elmt	Val.	Min	Max	Elmt	Val.	Min	Max	Elmt	Val.	Min	Max	Use Adduct
H	1	0	40	N	3	0	3	P	3	0	0	Br	1	0	0	H
2H	1	0	0	O	2	0	8	S	2	0	2	Pd	2	0	0	Na
B	3	0	0	F	1	0	0	Cl	1	0	0	I	3	0	0	K
C	4	0	30	Si	4	0	0	Cu	2	0	0	Pt	2	0	0	

Error Margin (ppm): 1000
HC Ratio: unlimited
Max Isotopes: all
MSn Iso RI (%): 75.00

DBE Range: not fixed
Apply N Rule: no
Isotope RI (%): 1.00
MSn Logic Mode: AND

Electron Ions: both
Use MSn Info: no
Isotope Res: 10000
Max Results: 40

イベント#: 1 MS(E+)　保持時間: 1.413 -> 1.413 - 1.467 -> 1.476　スキャン番号: 63 -> 63 - 71 -> 73

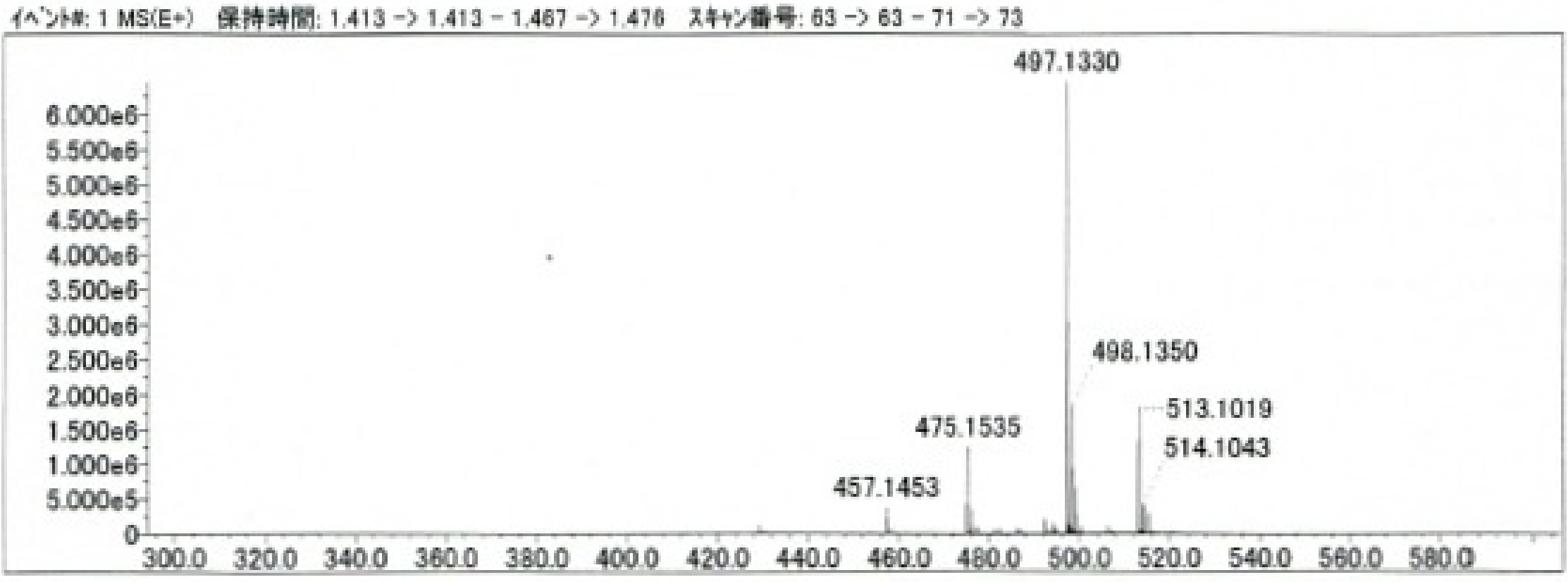

Measured region for 475.1535 m/z

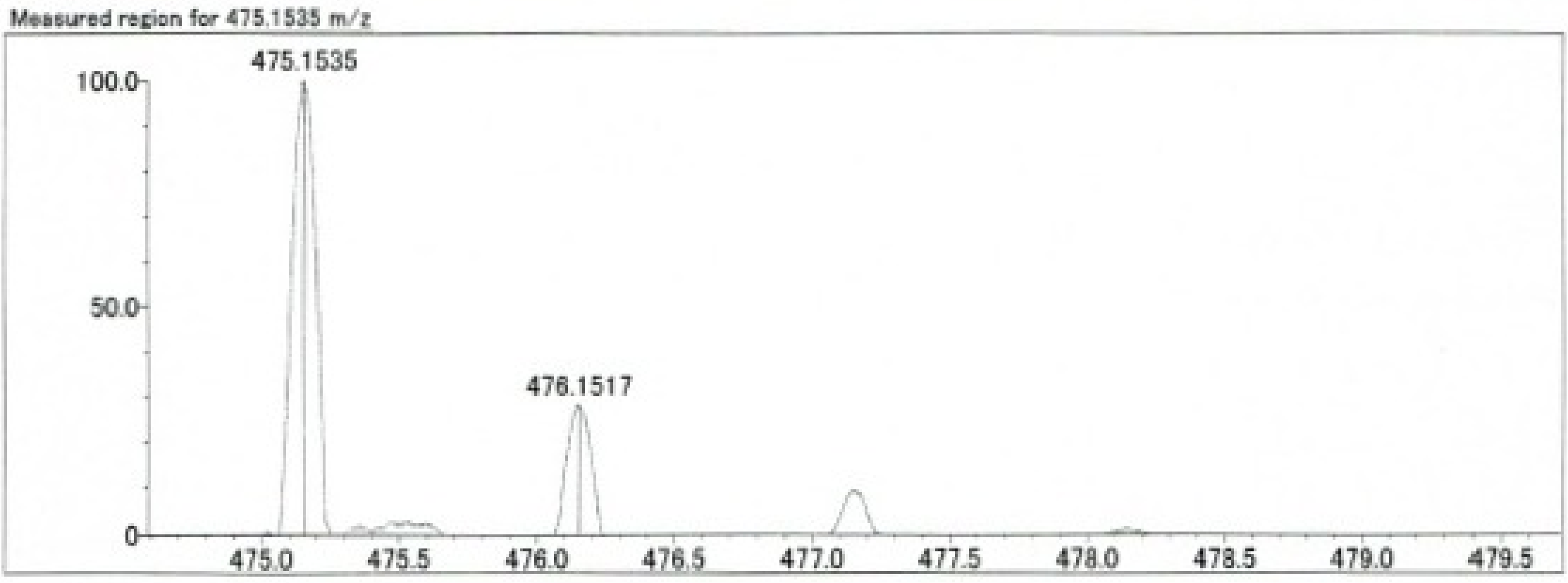

C23 H26 N2 O7 S [M+H]+ : Predicted region for 475.1533 m/z

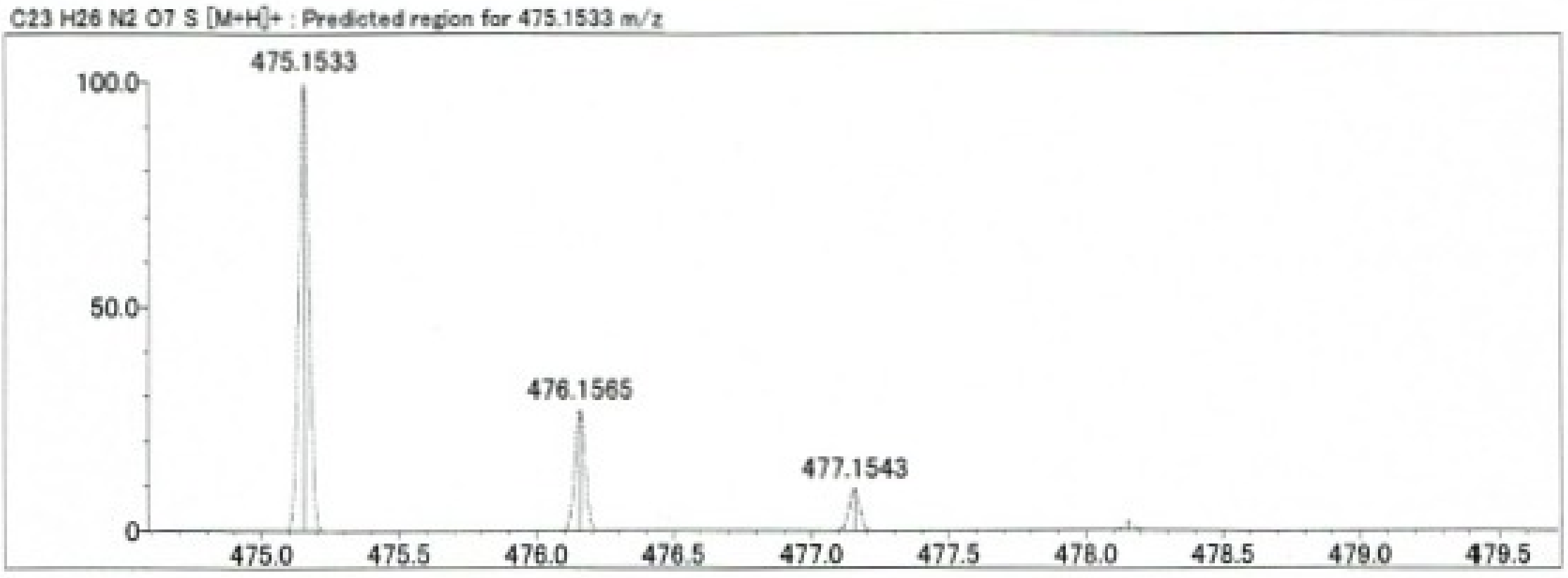

Rank	Score	Formula (M)	Ion	Meas. m/z	Pred. m/z	Df. (mDa)	Df. (ppm)	Iso	DBE
2	84.77	C23 H26 N2 O7 S	[M+H]+	475.1535	475.1533	0.2	0.42	84.77	12.0

4,9-Dimethyl-1-(*p*-pivaloyloxybenzenesulfonyl)-1,2,3,4-tetrahydro-5*H*-1,4-benzodiazepin-5-one (15).

Data File: C:¥LabSolutions¥Data¥TeikyoData¥創薬¥42J¥HTA16-95-34.lcd

Elmt	Val.	Min	Max	Elmt	Val.	Min	Max	Elmt	Val.	Min	Max	Elmt	Val.	Min	Max	Use Adduct
H	1	22	30	N	3	2	2	P	3	0	0	Pd	2	0	0	H
2H	1	0	0	O	2	2	7	S	2	1	1	I	3	0	0	Na
B	3	0	0	F	1	0	0	Cl	1	0	0					K
C	4	21	30	Si	4	0	0	Br	1	0	0					NH4

Error Margin (ppm): 20
HC Ratio: unlimited
Max Isotopes: all
MSn Iso RI (%): 75.00

DBE Range: not fixed
Apply N Rule: yes
Isotope RI (%): 1.00
MSn Logic Mode: AND

Electron Ions: both
Use MSn Info: no
Isotope Res: 10000
Max Results: 20

イベント#: 1 MS(E+) 保持時間: 1.360 -> 1.360 スキャン番号: 55 -> 55

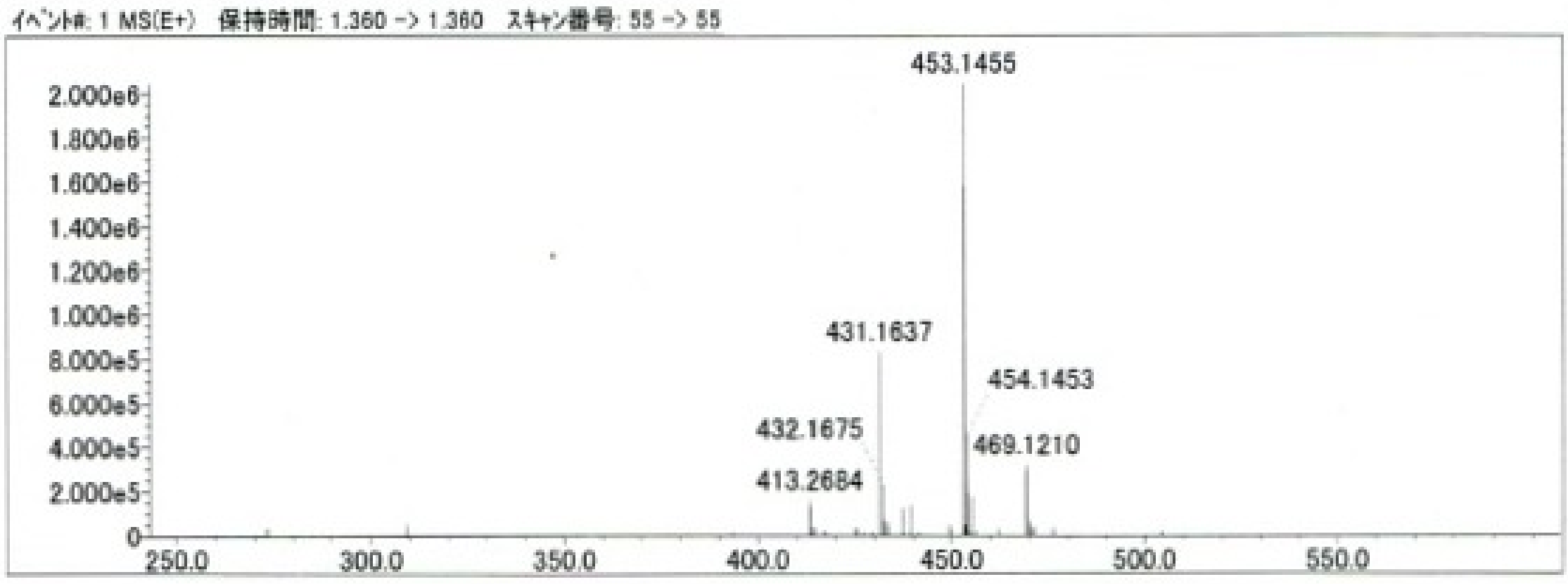

Measured region for 431.1637 m/z

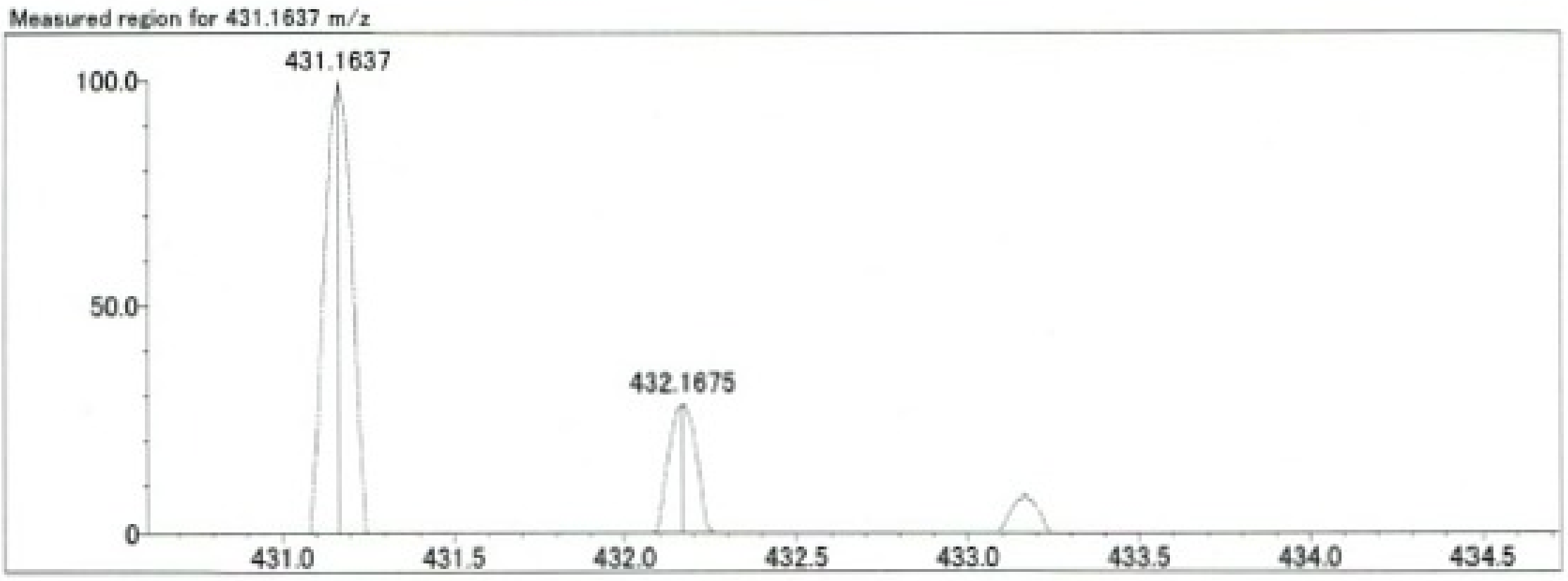

C22 H26 N2 O5 S [M+H]+ : Predicted region for 431.1635 m/z

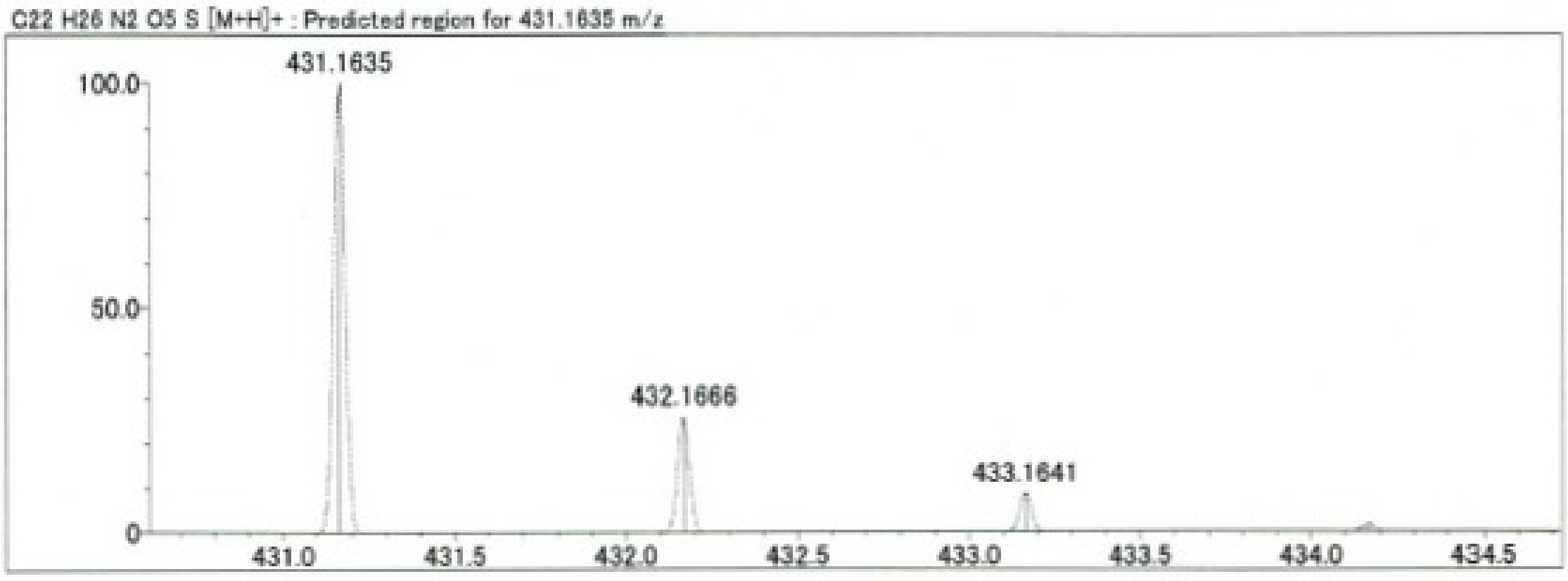

Rank	Score	Formula (M)	Ion	Meas. m/z	Pred. m/z	Df. (mDa)	Df. (ppm)	Iso	DBE
1	100.00	C22 H26 N2 O5 S	[M+H]+	431.1637	431.1635	0.2	0.46	100.00	11.0

Methyl 2,3,4,5-tetrahydro-2-oxo-1*H*-1,5-benzodiazepine-1-acetate (6c)

Data File: C:¥LabSolutions¥Data¥TeikyoData¥創薬¥42J¥HTA17-26-24.lcd

Elmt	Val.	Min	Max	Elmt	Val.	Min	Max	Elmt	Val.	Min	Max	Elmt	Val.	Min	Max	Use Adduct
H	1	0	50	N	3	0	4	P	3	0	0	Br	1	0	0	H
2H	1	0	0	O	2	0	5	S	2	0	0	Pd	2	0	0	Na
B	3	0	0	F	1	0	0	Cl	1	0	0	I	3	0	0	K
C	4	0	30	Si	4	0	0	Cu	2	0	0	Pt	2	0	0	

Error Margin (ppm): 50
HC Ratio: unlimited
Max Isotopes: all
MSn Iso RI (%): 75.00

DBE Range: not fixed
Apply N Rule: no
Isotope RI (%): 1.00
MSn Logic Mode: AND

Electron Ions: both
Use MSn Info: no
Isotope Res: 10000
Max Results: 40

イベント#: 1 MS(E+) 保持時間: 1.213 -> 1.240 - 1.467 -> 1.464 スキャン番号: 33 -> 37 - 71 -> 71

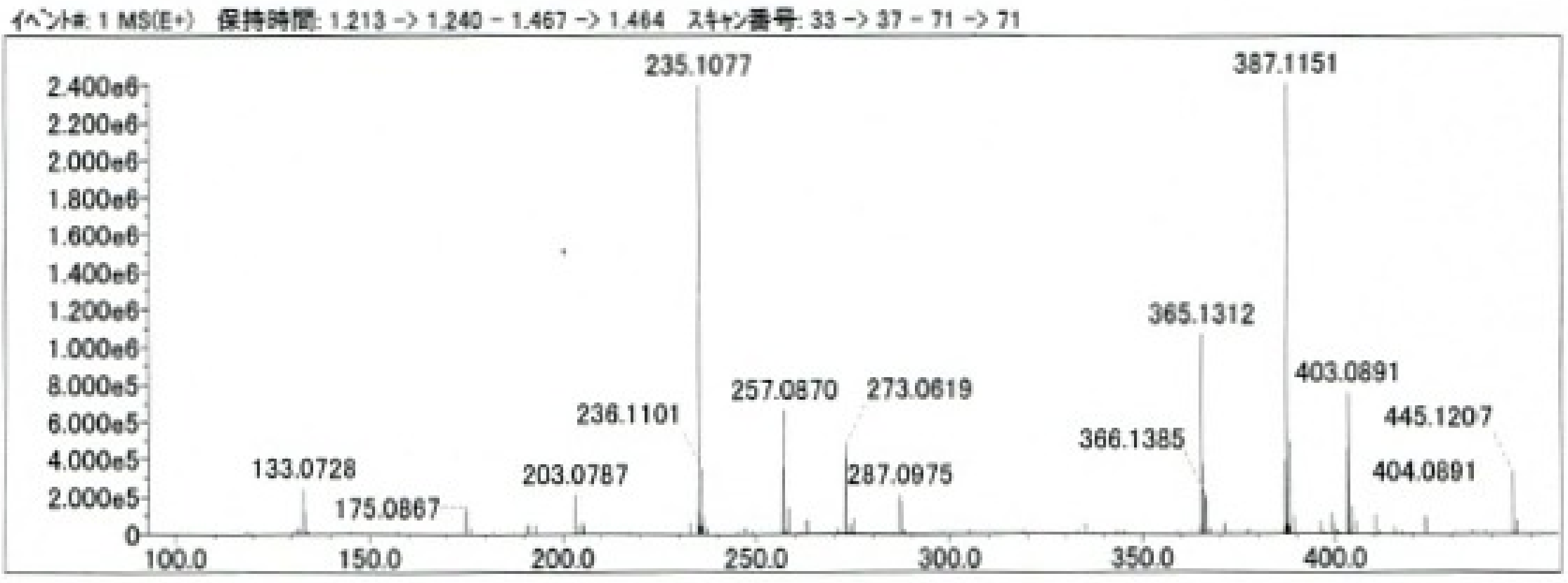

Measured region for 235.1077 m/z

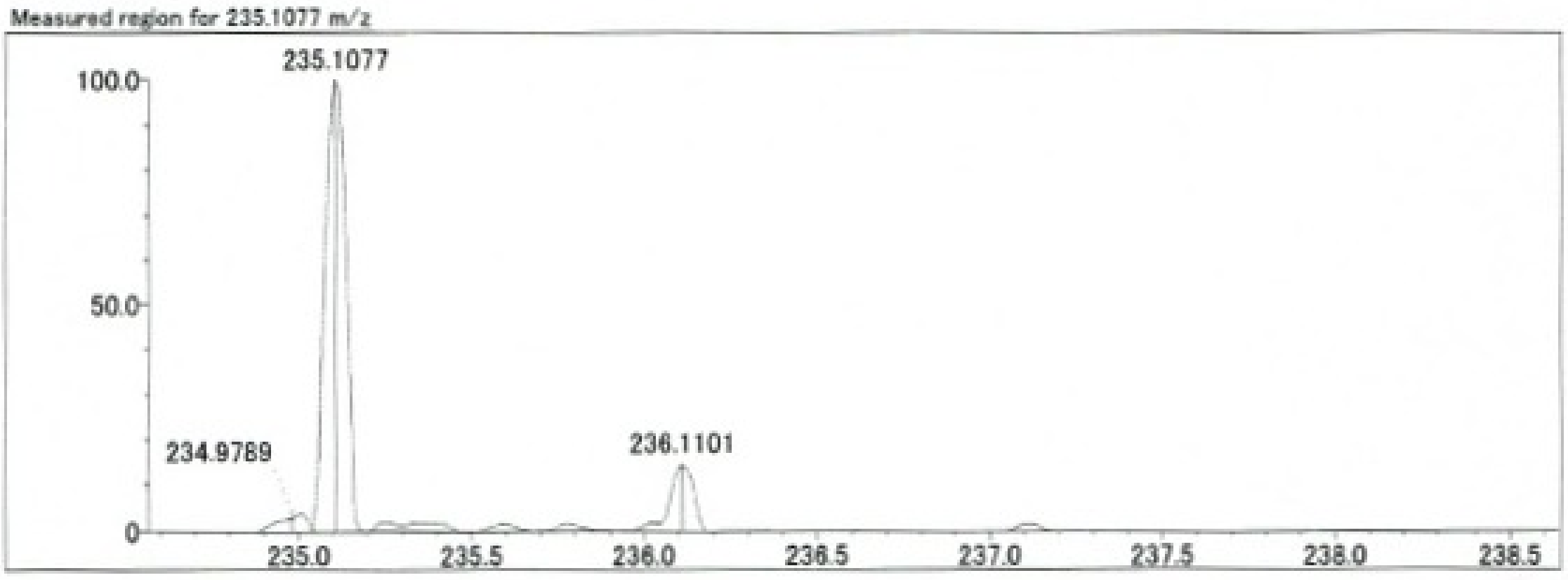

C12 H14 N2 O3 [M+H]+ : Predicted region for 235.1077 m/z

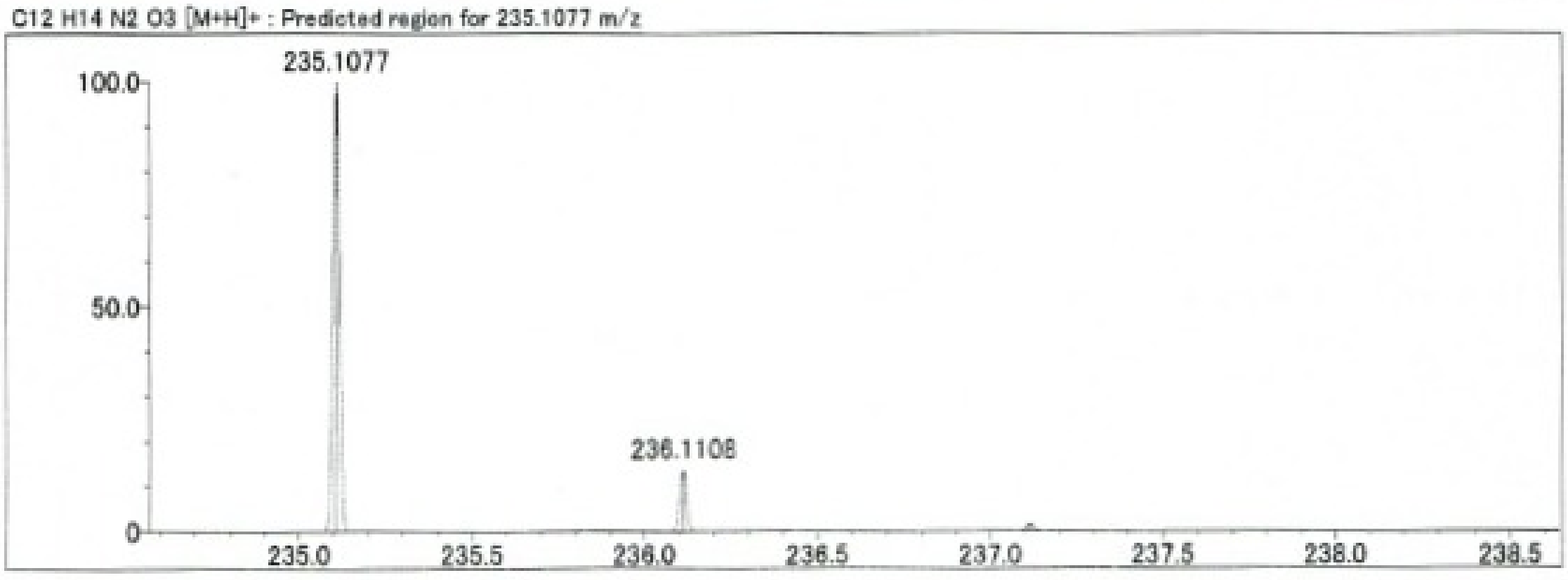

Rank	Score	Formula (M)	Ion	Meas. m/z	Pred. m/z	Df. (mDa)	Df. (ppm)	Iso	DBE
1	74.87	C12 H14 N2 O3	[M+H]+	235.1077	235.1077	-0.0	0.00	74.87	7.0

Methyl 6-methyl-2,3,4,5-tetrahydro-2-oxo-1*H*-1,5-benzodiazepine-1-acetate (6d)

Data File: C:¥LabSolutions¥Data¥TeikyoData¥創薬¥42J¥HTA16-129-35.lcd

Elmt	Val.	Min	Max	Elmt	Val.	Min	Max	Elmt	Val.	Min	Max	Elmt	Val.	Min	Max	Use Adduct
H	1	0	50	N	3	0	10	P	3	0	0	Br	1	0	1	H
2H	1	0	0	O	2	0	15	S	2	0	0	Pd	2	0	0	Na
B	3	0	0	F	1	0	0	Cl	1	0	0	I	3	0	0	
C	4	0	50	Si	4	0	0	Cu	2	0	0	Pt	2	0	0	

Error Margin (ppm): 50
HC Ratio: unlimited
Max Isotopes: all
MSn Iso RI (%): 75.00

DBE Range: not fixed
Apply N Rule: no
Isotope RI (%): 1.00
MSn Logic Mode: AND

Electron Ions: both
Use MSn Info: no
Isotope Res: 10000
Max Results: 40

ｲﾍﾞﾝﾄ#: 1 MS(E+) 保持時間: 1.227 -> 1.227 - 1.347 -> 1.340 ｽｷｬﾝ番号: 35 -> 35 - 53 -> 53

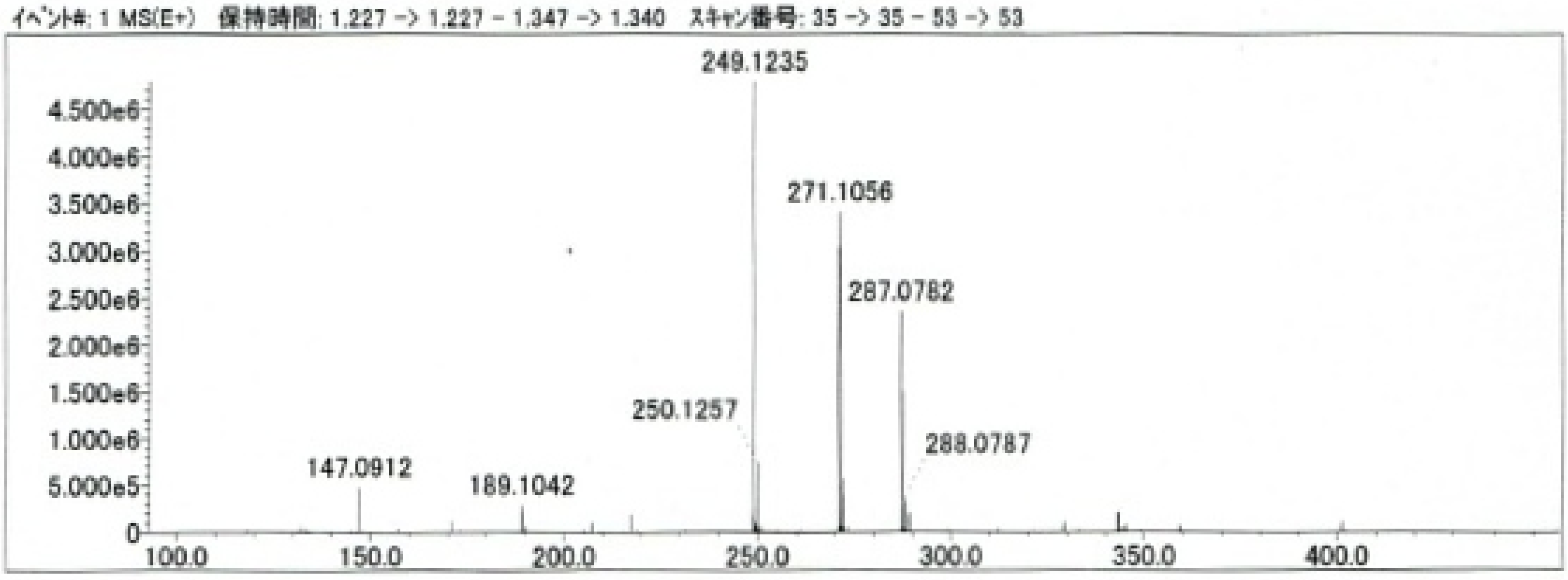

Measured region for 249.1235 m/z

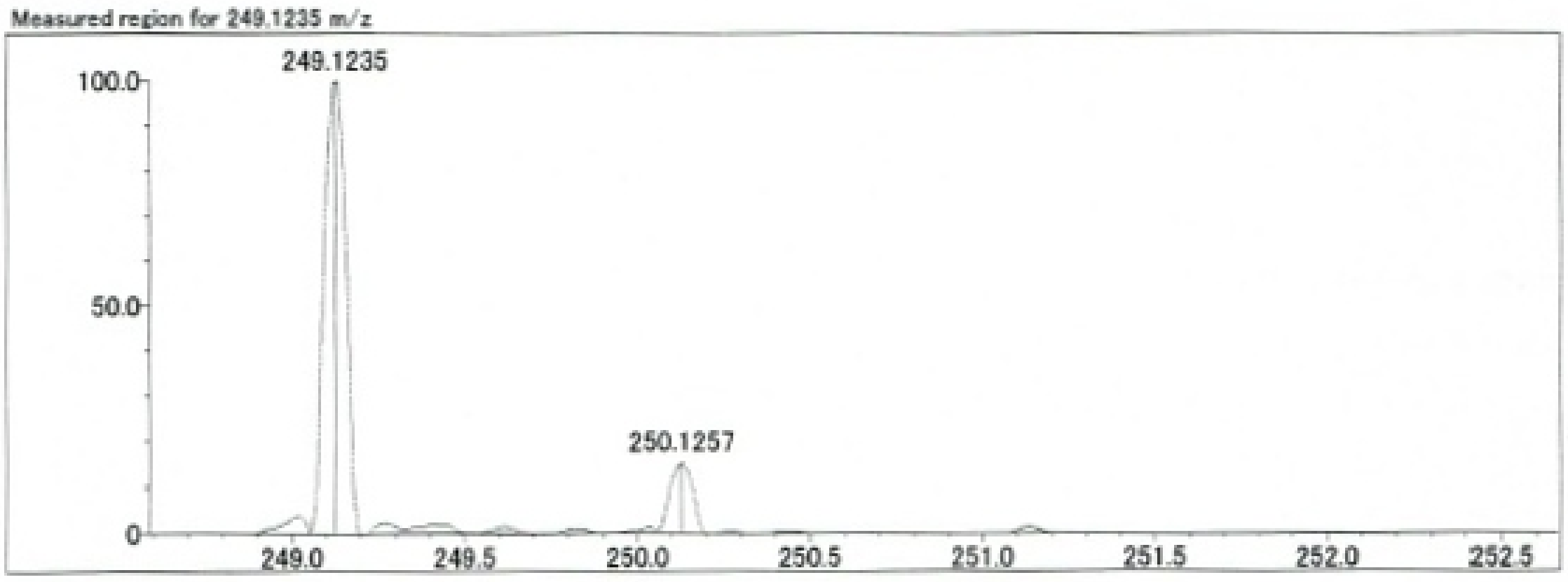

C13 H16 N2 O3 [M+H]+ : Predicted region for 249.1234 m/z

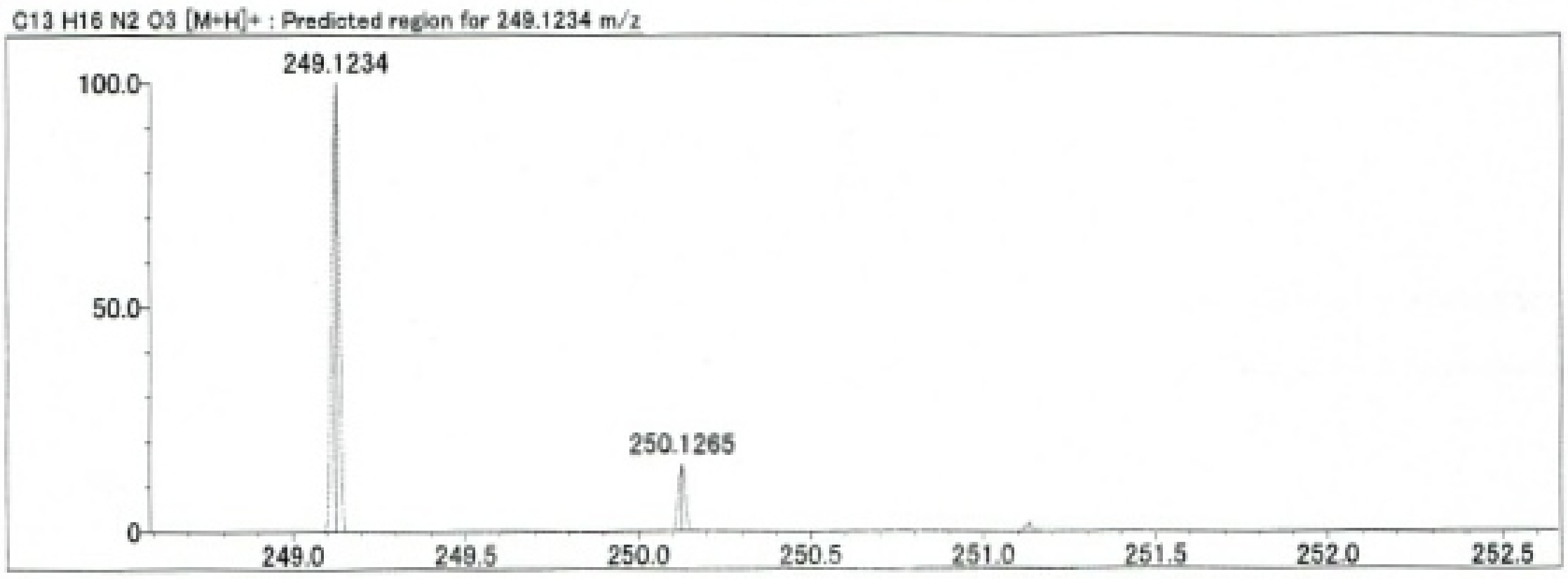

Rank	Score	Formula (M)	Ion	Meas. m/z	Pred. m/z	Df. (mDa)	Df. (ppm)	Iso	DBE
1	76.40	C13 H16 N2 O3	[M+H]+	249.1235	249.1234	0.1	0.40	76.40	7.0

Benzyl 6-methyl-2,3,4,5-tetrahydro-2-oxo-1*H*-1,5-benzodiazepine-1-acetate (6e)

Data File: C:¥LabSolutions¥Data¥TeikyoData¥創薬¥42J¥HTA16-131-37.lcd

Elmt	Val.	Min	Max	Elmt	Val.	Min	Max	Elmt	Val.	Min	Max	Elmt	Val.	Min	Max	Use Adduct
H	1	0	50	N	3	0	10	P	3	0	0	Br	1	0	1	H
2H	1	0	0	O	2	0	15	S	2	0	0	Pd	2	0	0	Na
B	3	0	0	F	1	0	0	Cl	1	0	0	I	3	0	0	
C	4	0	50	Si	4	0	0	Cu	2	0	0	Pt	2	0	0	

Error Margin (ppm): 50
HC Ratio: unlimited
Max Isotopes: all
MSn Iso RI (%): 75.00

DBE Range: not fixed
Apply N Rule: no
Isotope RI (%): 1.00
MSn Logic Mode: AND

Electron Ions: both
Use MSn Info: no
Isotope Res: 10000
Max Results: 40

ｲﾍﾞﾝﾄ#: 1 MS(E+)　保持時間: 1.160 -> 1.173 - 1.387 -> 1.385　ｽｷｬﾝ番号: 25 -> 27 - 59 -> 59

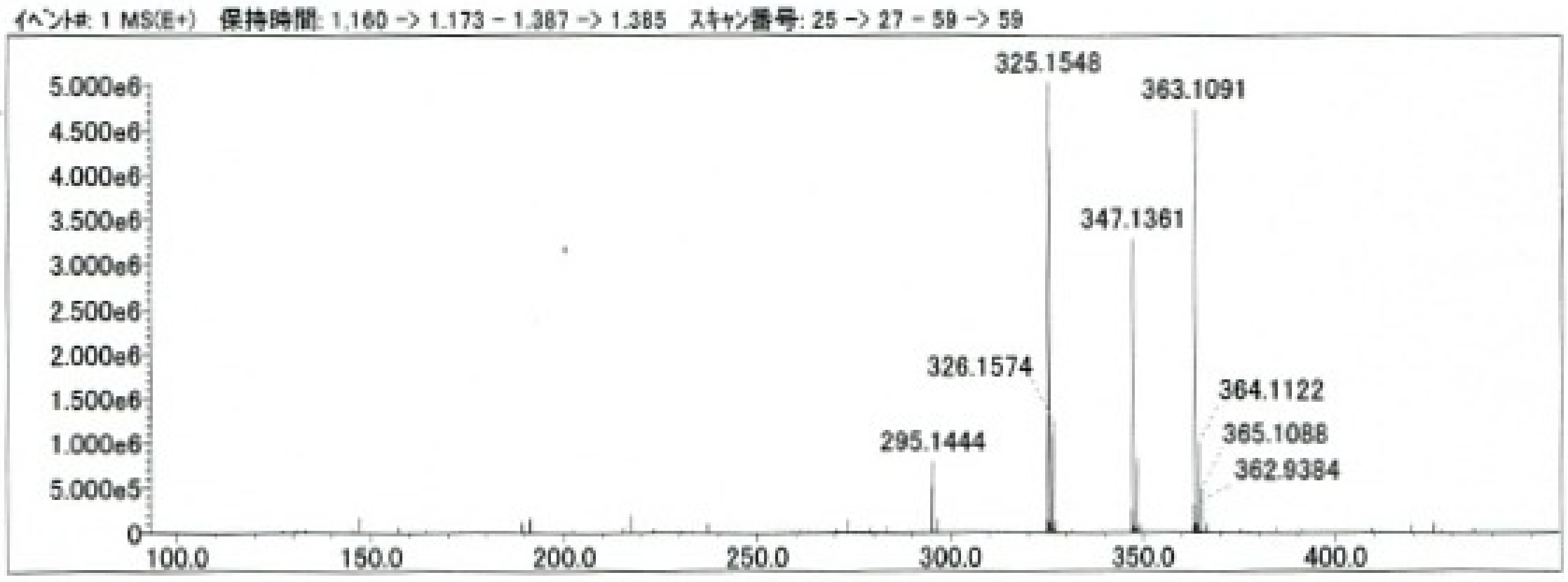

Measured region for 325.1548 m/z

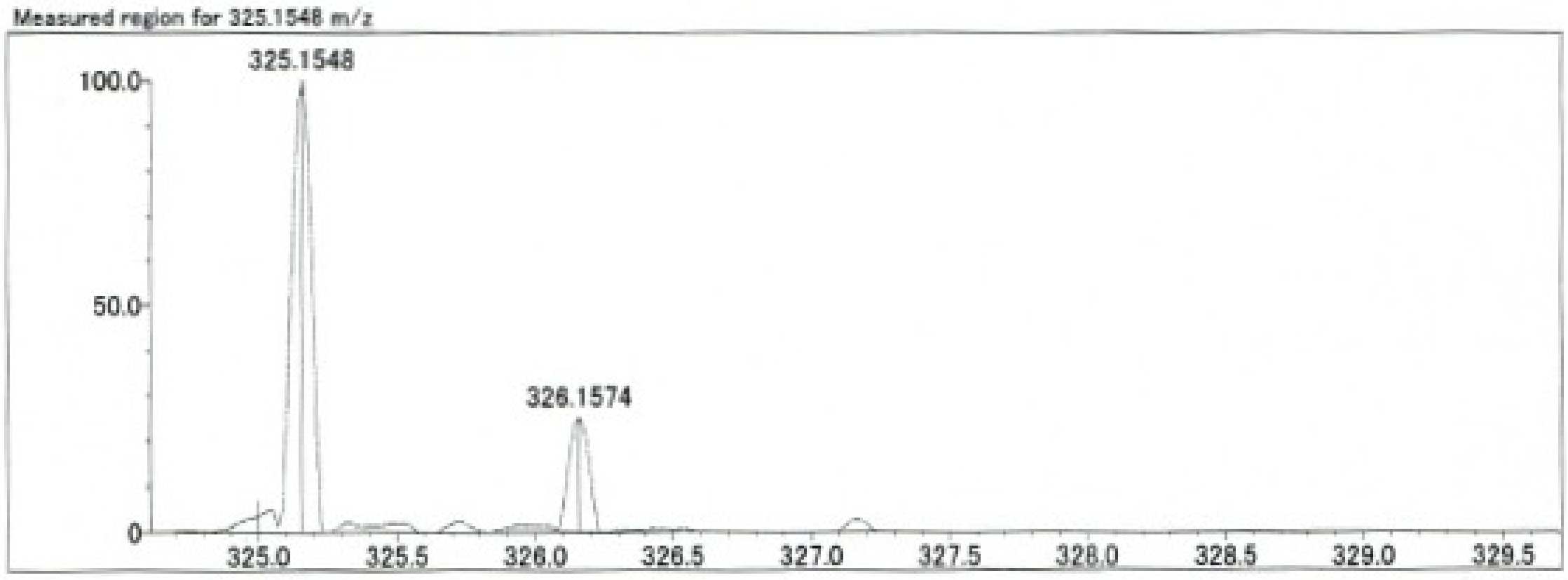

C19 H20 N2 O3 [M+H]+ : Predicted region for 325.1547 m/z

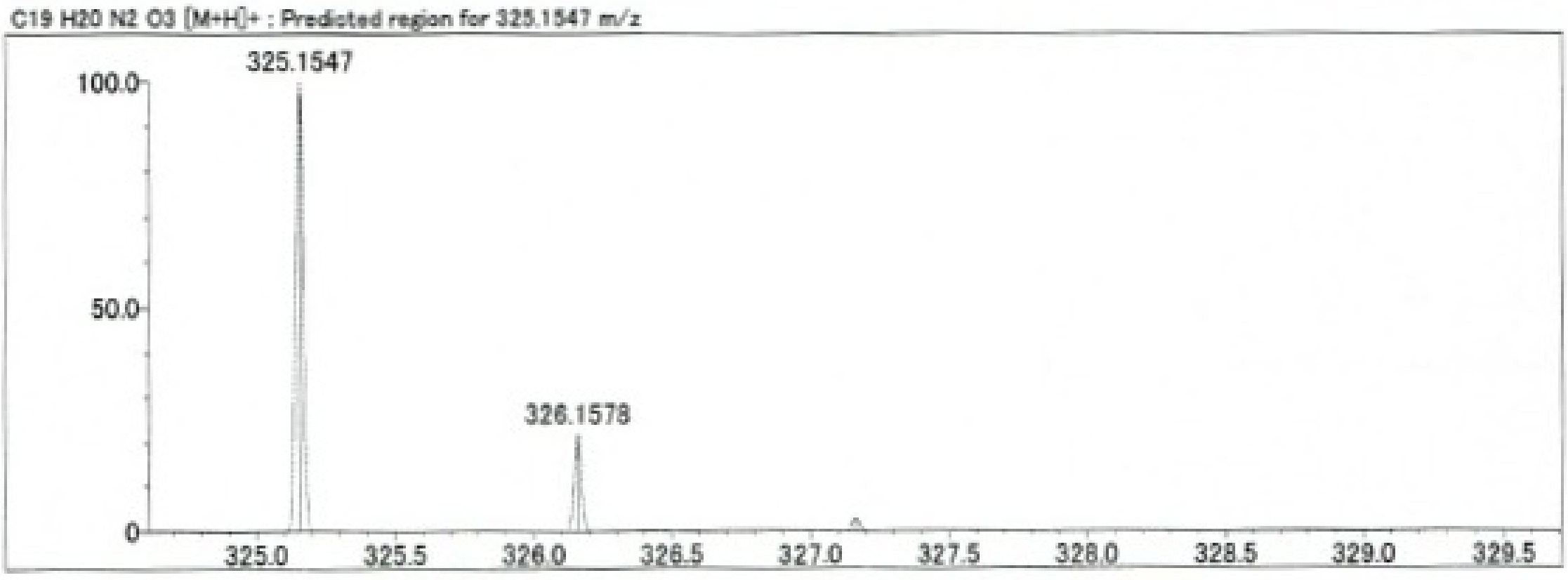

Rank	Score	Formula (M)	Ion	Meas. m/z	Pred. m/z	Df. (mDa)	Df. (ppm)	Iso	DBE
1	82.71	C19 H20 N2 O3	[M+H]+	325.1548	325.1547	0.1	0.31	82.71	11.0

N-(2-Hydroxyethyl)-*N*,3-dimethyl-2-nitrobenzamide (11)

Data File: C:¥LabSolutions¥Data¥TeikyoData¥創薬¥42J¥HTA16-50-42.lcd

Elmt	Val.	Min	Max	Elmt	Val.	Min	Max	Elmt	Val.	Min	Max	Elmt	Val.	Min	Max	Use Adduct
H	1	10	30	N	3	2	3	P	3	0	0	Br	1	0	0	H
2H	1	0	0	O	2	1	5	S	2	0	0	Pd	2	0	0	Na
B	3	0	0	F	1	0	0	Cl	1	0	0	I	3	0	0	
C	4	10	20	Si	4	0	0	Cu	2	0	0					

Error Margin (mDa): 100.0
HC Ratio: unlimited
Max Isotopes: all
MSn Iso RI (%): 75.00

DBE Range: not fixed
Apply N Rule: no
Isotope RI (%): 1.00
MSn Logic Mode: AND

Electron Ions: both
Use MSn Info: no
Isotope Res: 10000
Max Results: 40

イベント#: 1 MS(E+) 保持時間: 1.307 -> 1.307 スキャン番号: 47 -> 47

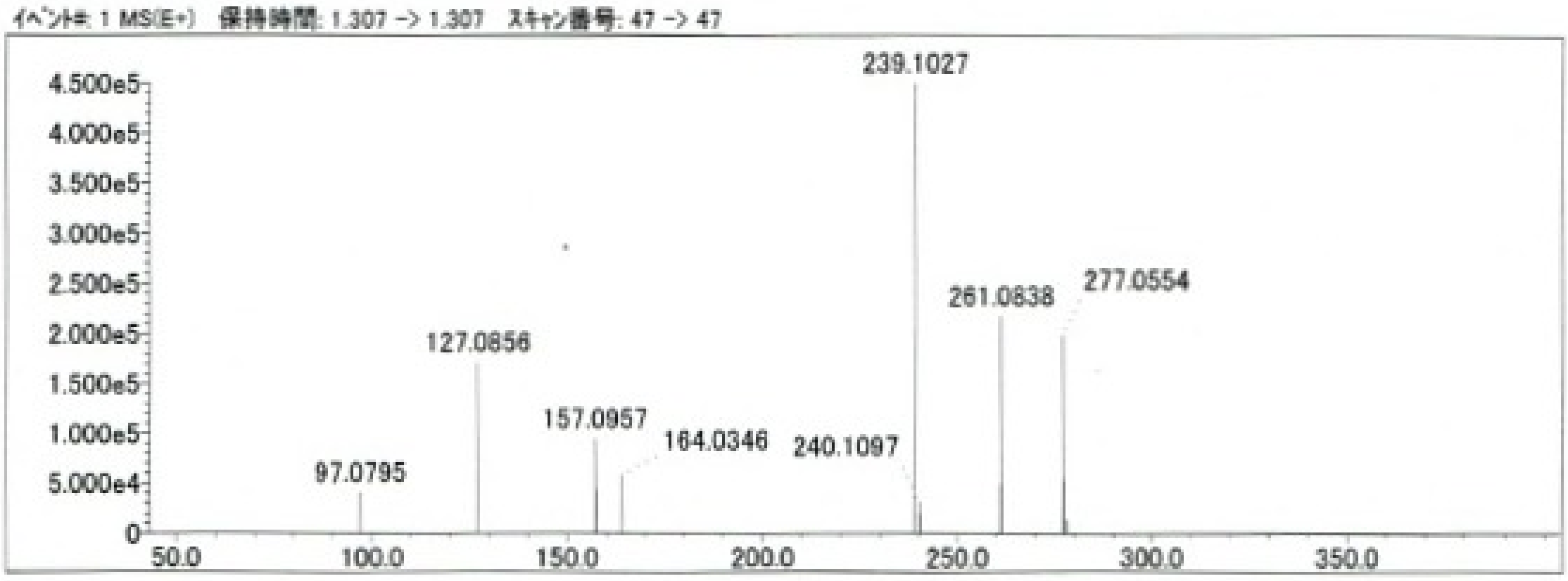

Measured region for 239.1027 m/z

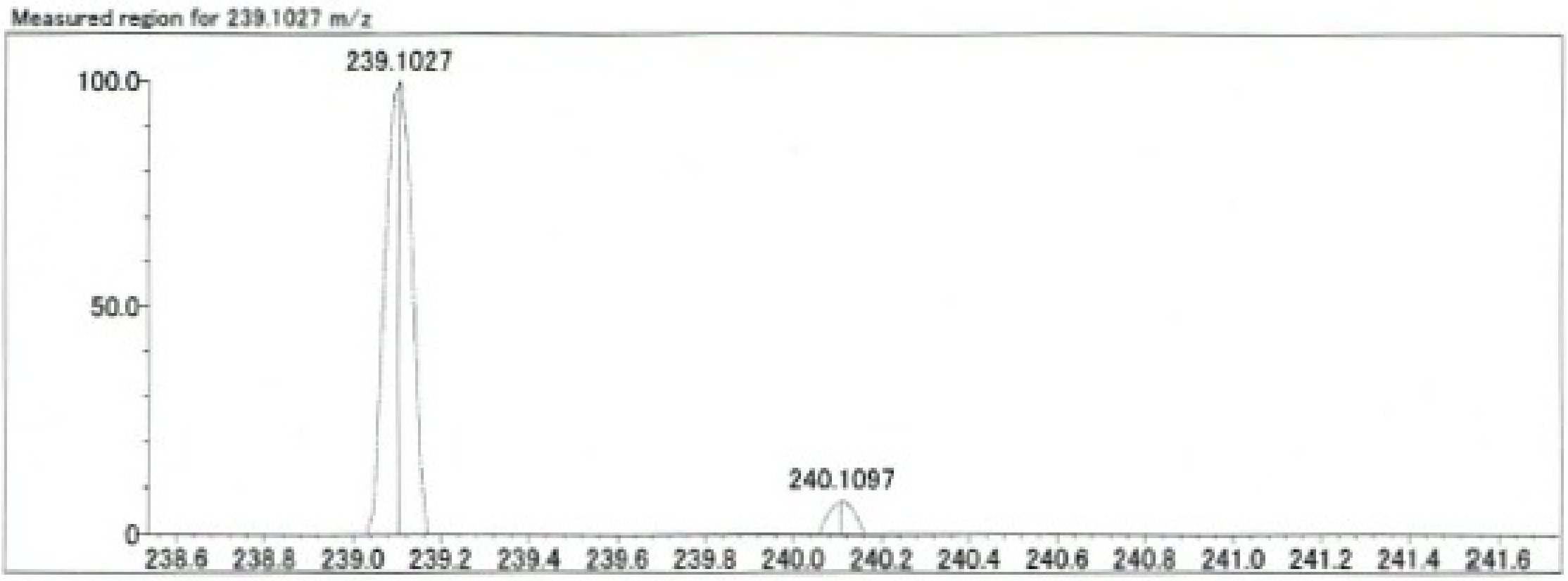

C11 H14 N2 O4 [M+H]+ : Predicted region for 239.1026 m/z

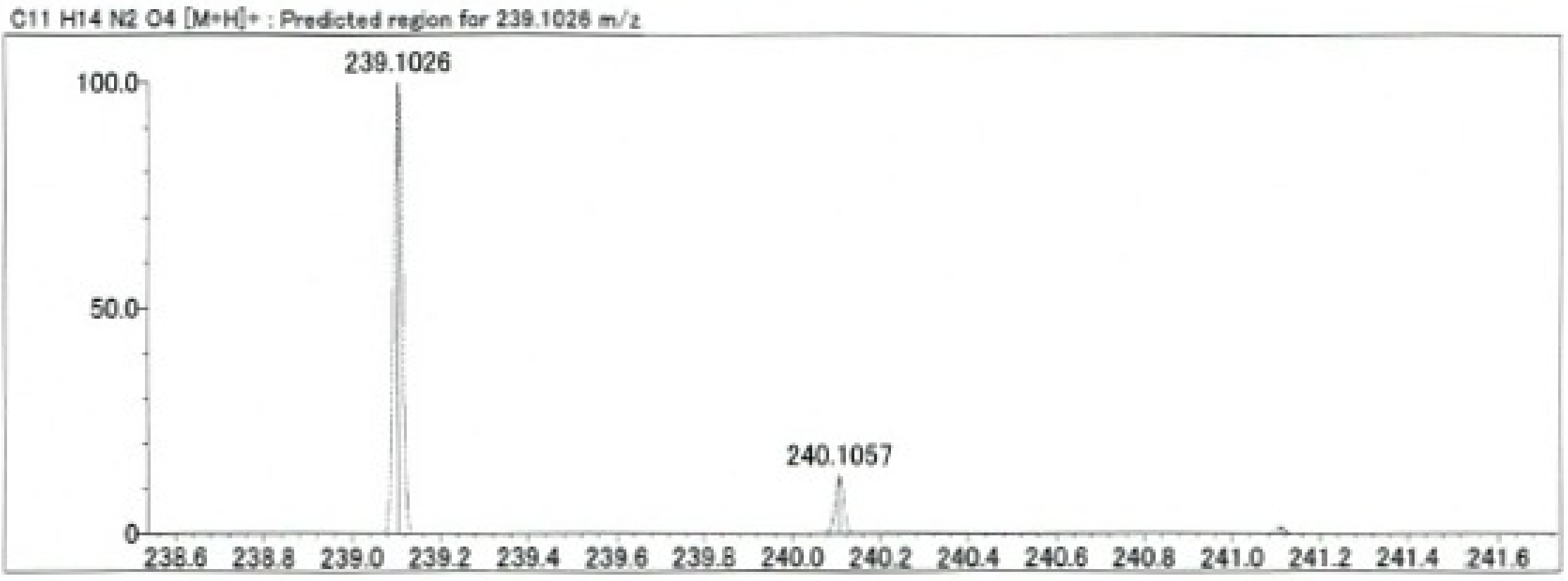

Rank	Score	Formula (M)	Ion	Meas. m/z	Pred. m/z	Df. (mDa)	Df. (ppm)	Iso	DBE
1	59.45	C11 H14 N2 O4	[M+H]+	239.1027	239.1026	0.1	0.42	59.45	6.0

2-Amino-*N*-(2-hydroxyethyl)-*N*,3-dimethylbenzamide (12)

Data File: C:¥LabSolutions¥Data¥TeikyoData¥創薬¥42J¥HTA16-81-28.lcd

Elmt	Val.	Min	Max	Elmt	Val.	Min	Max	Elmt	Val.	Min	Max	Elmt	Val.	Min	Max	Use Adduct
H	1	0	50	N	3	0	10	P	3	0	0	Br	1	0	1	H
2H	1	0	0	O	2	0	15	S	2	0	0	Pd	2	0	0	Na
B	3	0	0	F	1	0	0	Cl	1	0	0	I	3	0	0	
C	4	0	50	Si	4	0	0	Cu	2	0	0	Pt	2	0	0	

Error Margin (ppm): 50
HC Ratio: unlimited
Max Isotopes: all
MSn Iso RI (%): 75.00
DBE Range: not fixed
Apply N Rule: no
Isotope RI (%): 1.00
MSn Logic Mode: AND
Electron Ions: both
Use MSn Info: no
Isotope Res: 10000
Max Results: 40

イベント#: 1 MS(E+) 保持時間: 1.307 -> 1.307 - 1.360 -> 1.370 スキャン番号: 47 -> 47 - 55 -> 57

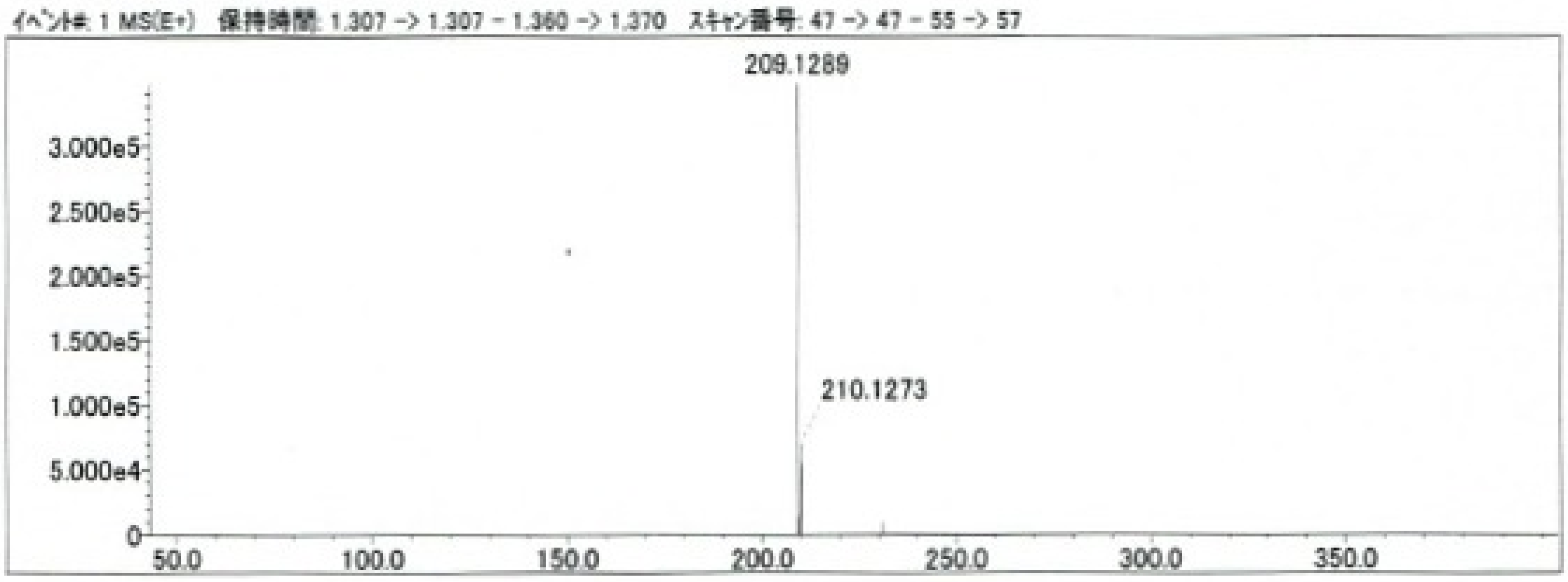

Measured region for 209.1289 m/z

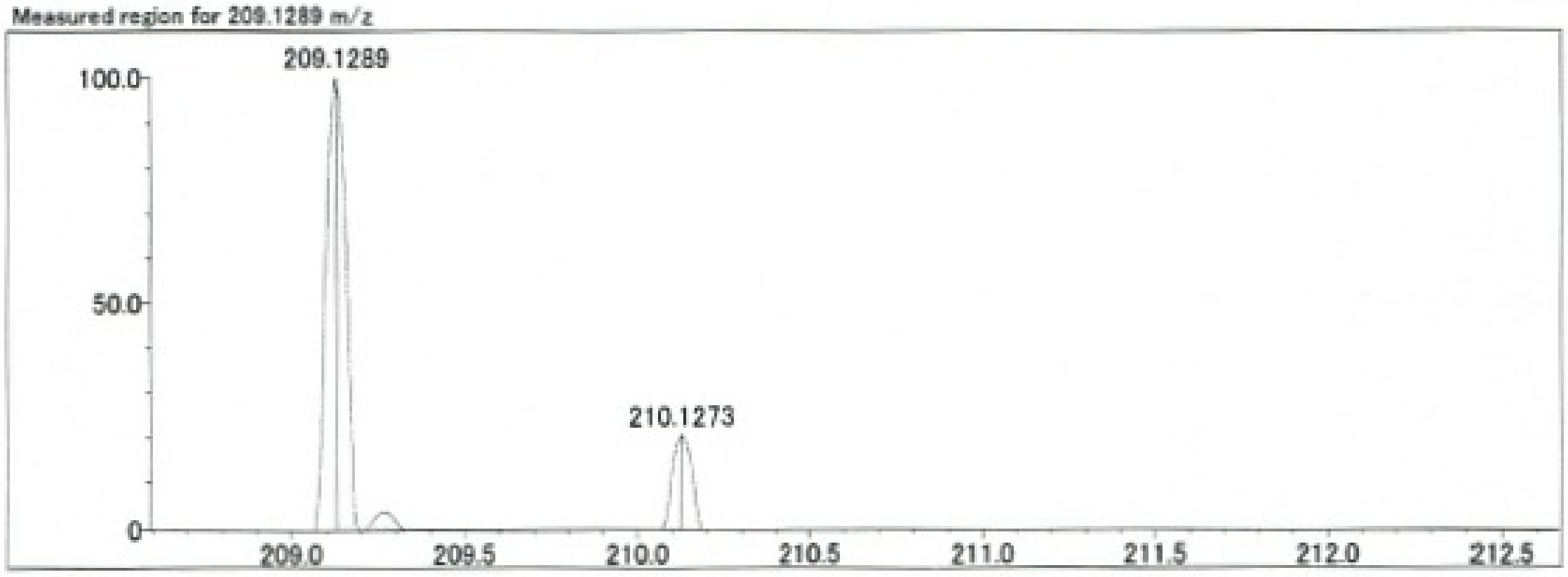

C11 H16 N2 O2 [M+H]+ : Predicted region for 209.1285 m/z

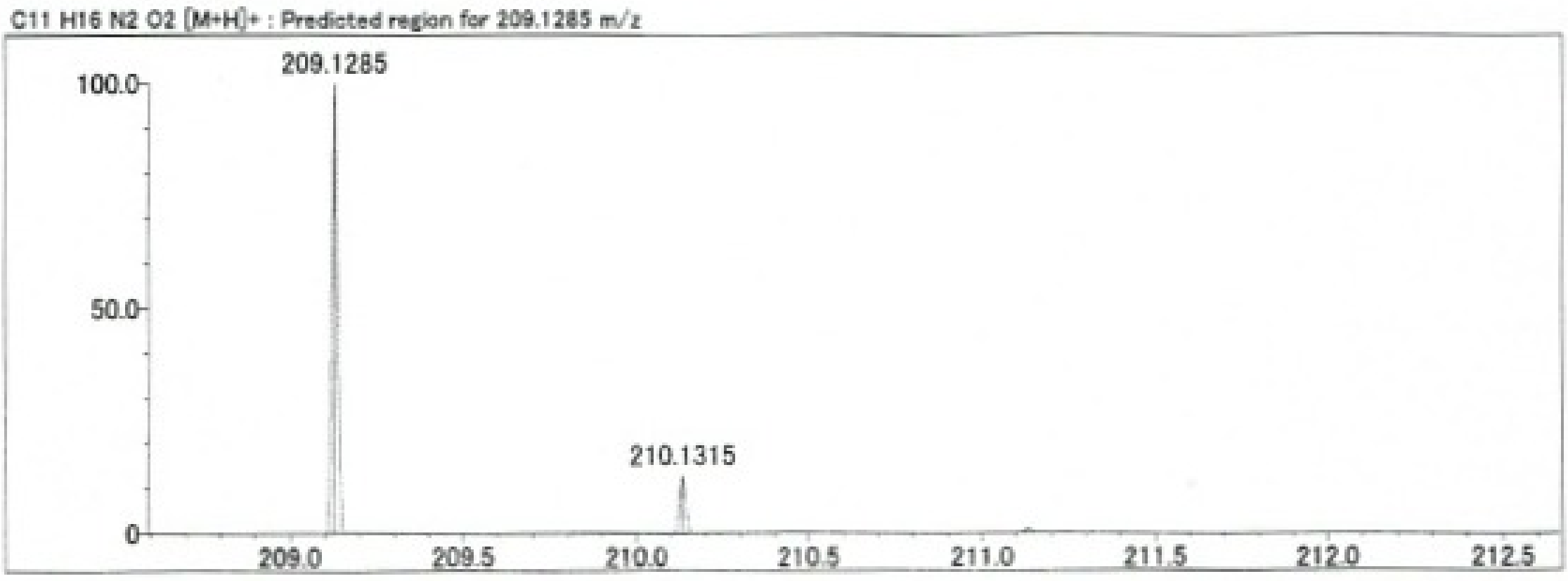

Rank	Score	Formula (M)	Ion	Meas. m/z	Pred. m/z	Df. (mDa)	Df. (ppm)	Iso	DBE
1	51.41	C11 H16 N2 O2	[M+H]+	209.1289	209.1285	0.4	1.91	52.60	5.0

4-(N-(2-(N'-(2-Hydroxyethyl)-N'-methylcarbamoyl)-6-methylphenyl)sulfamoyl)phenyl pivalate (13)

Data File: C:¥LabSolutions¥Data¥TeikyoData¥創薬¥42J¥HTA16-89-33.lcd

Elmt	Val.	Min	Max	Elmt	Val.	Min	Max	Elmt	Val.	Min	Max	Elmt	Val.	Min	Max	Use Adduct
H	1	0	50	N	3	0	10	P	3	0	0	Br	1	0	0	H
2H	1	0	0	O	2	0	15	S	2	0	1	Pd	2	0	0	Na
B	3	0	0	F	1	0	0	Cl	1	0	0	I	3	0	0	K
C	4	0	50	Si	4	0	0	Cu	2	0	0	Pt	2	0	0	

Error Margin (ppm): 50
HC Ratio: unlimited
Max Isotopes: all
MSn Iso RI (%): 75.00

DBE Range: not fixed
Apply N Rule: no
Isotope RI (%): 1.00
MSn Logic Mode: AND

Electron Ions: both
Use MSn Info: no
Isotope Res: 10000
Max Results: 40

ｲﾍﾞﾝﾄ#: 1 MS(E+) 保持時間: 1.280 -> 1.293 ｽｷｬﾝ番号: 43 -> 45

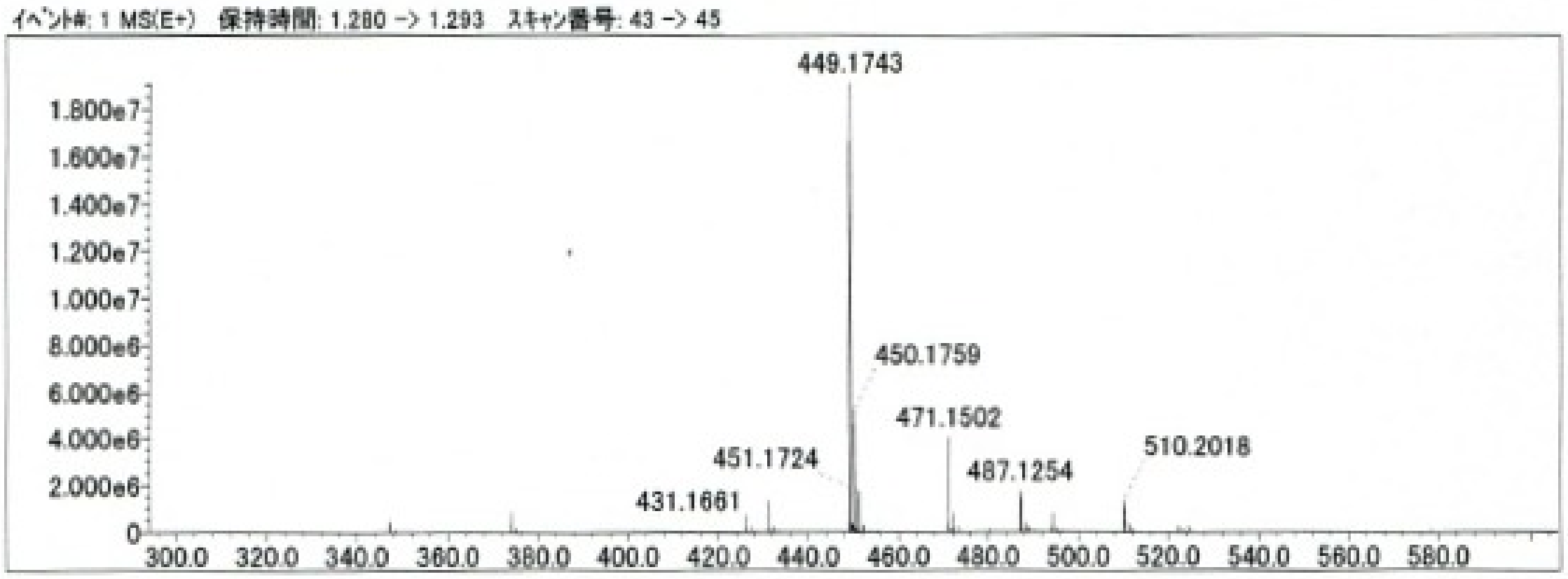

Measured region for 449.1743 m/z

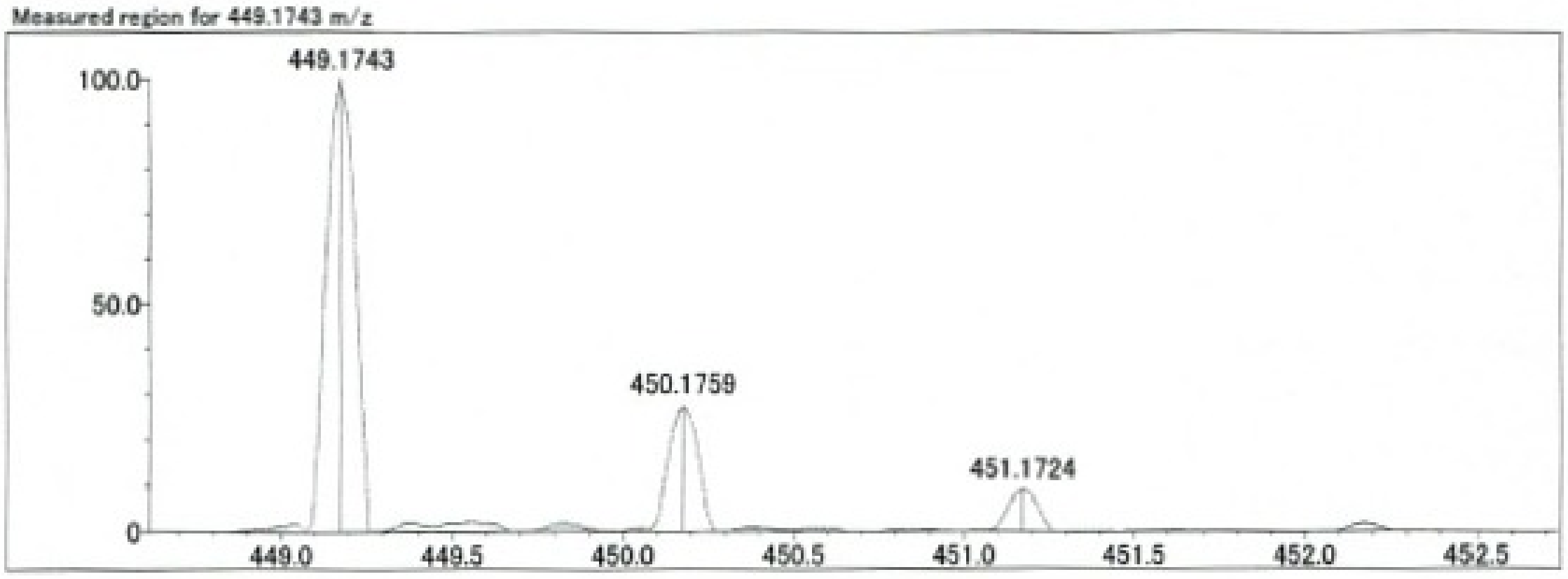

C22 H28 N2 O6 S [M+H]+ : Predicted region for 449.1741 m/z

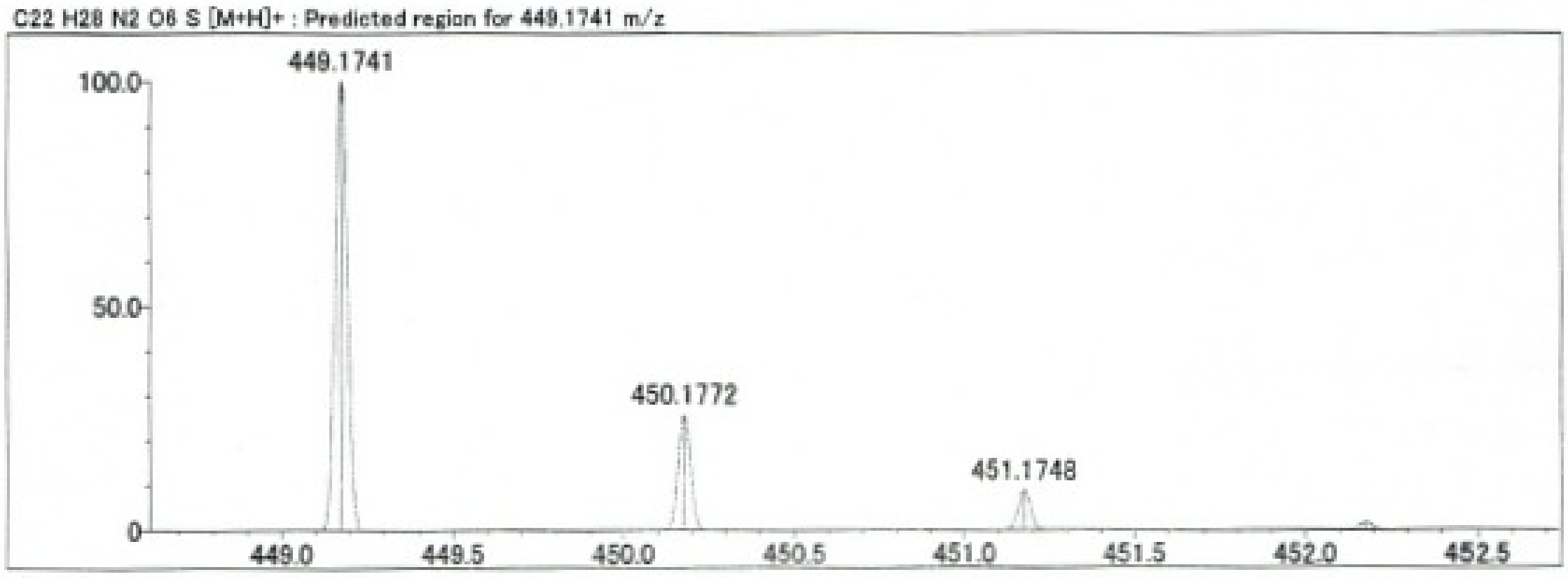

Rank	Score	Formula (M)	Ion	Meas. m/z	Pred. m/z	Df. (mDa)	Df. (ppm)	Iso	DBE
2	95.07	C22 H28 N2 O6 S	[M+H]+	449.1743	449.1741	0.2	0.45	95.07	10.0

4-(*N*-(2-(*N'*-(2-((Methylsulfonyl)oxy)ethyl)-*N'*-methylcarbamoyl)-6-mthylphenyl)sulfamoyl) phenyl pivalate (14)

Data File: C:¥LabSolutions¥Data¥TeikyoData¥創薬¥42J¥HTA17-43-34aAPCI2lod.lod

Elmt	Val.	Min	Max	Elmt	Val.	Min	Max	Elmt	Val.	Min	Max	Elmt	Val.	Min	Max	Use Adduct
H	1	30	40	N	3	2	2	P	3	0	0	Br	1	0	0	H
2H	1	0	0	O	2	8	8	S	2	2	2	Pd	2	0	0	Na
B	3	0	0	F	1	0	0	Cl	1	0	0	I	3	0	0	K
C	4	23	23	Si	4	0	0	Cu	2	0	0	Pt	2	0	0	

Error Margin (ppm): 1000
HC Ratio: unlimited
Max Isotopes: all
MSn Iso RI (%): 75.00

DBE Range: not fixed
Apply N Rule: no
Isotope RI (%): 1.00
MSn Logic Mode: AND

Electron Ions: both
Use MSn Info: no
Isotope Res: 10000
Max Results: 40

ｲﾍﾞﾝﾄ#: 1 MS(C+)　保持時間: 1.280 -> 1.280 - 1.587 -> 1.695　ｽｷｬﾝ番号: 43 -> 43 - 89 -> 105

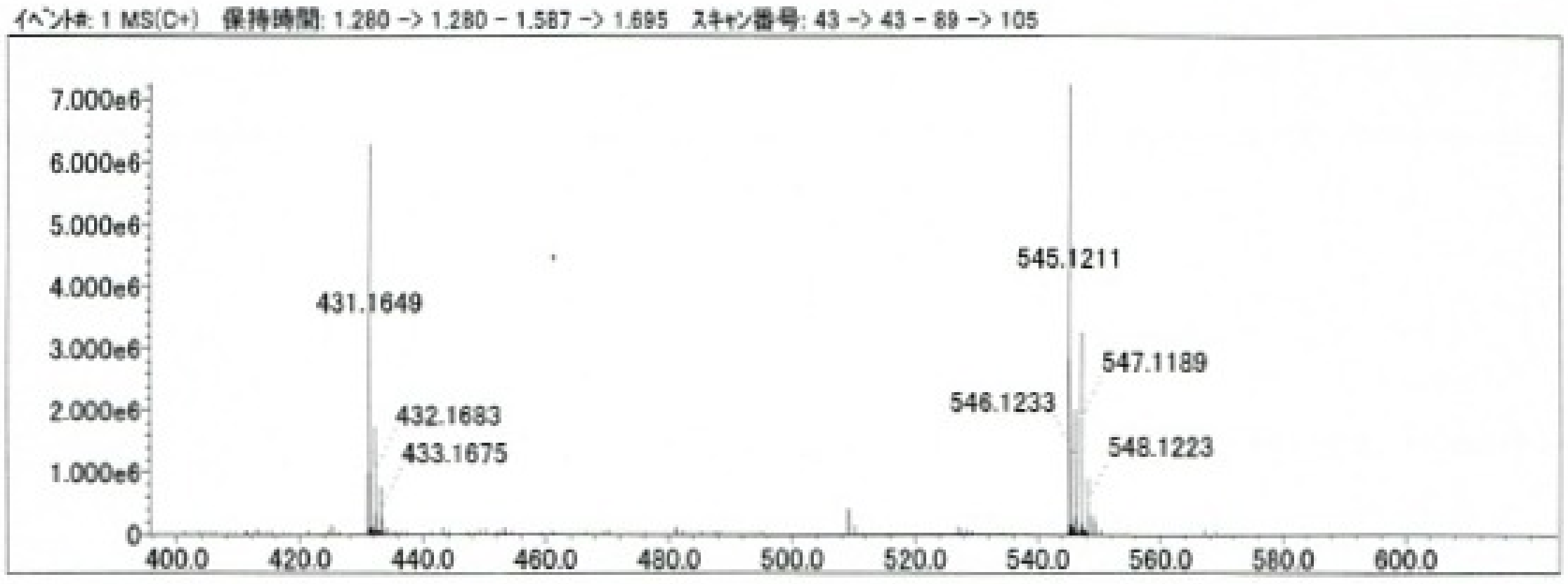

Measured region for 527.1519 m/z

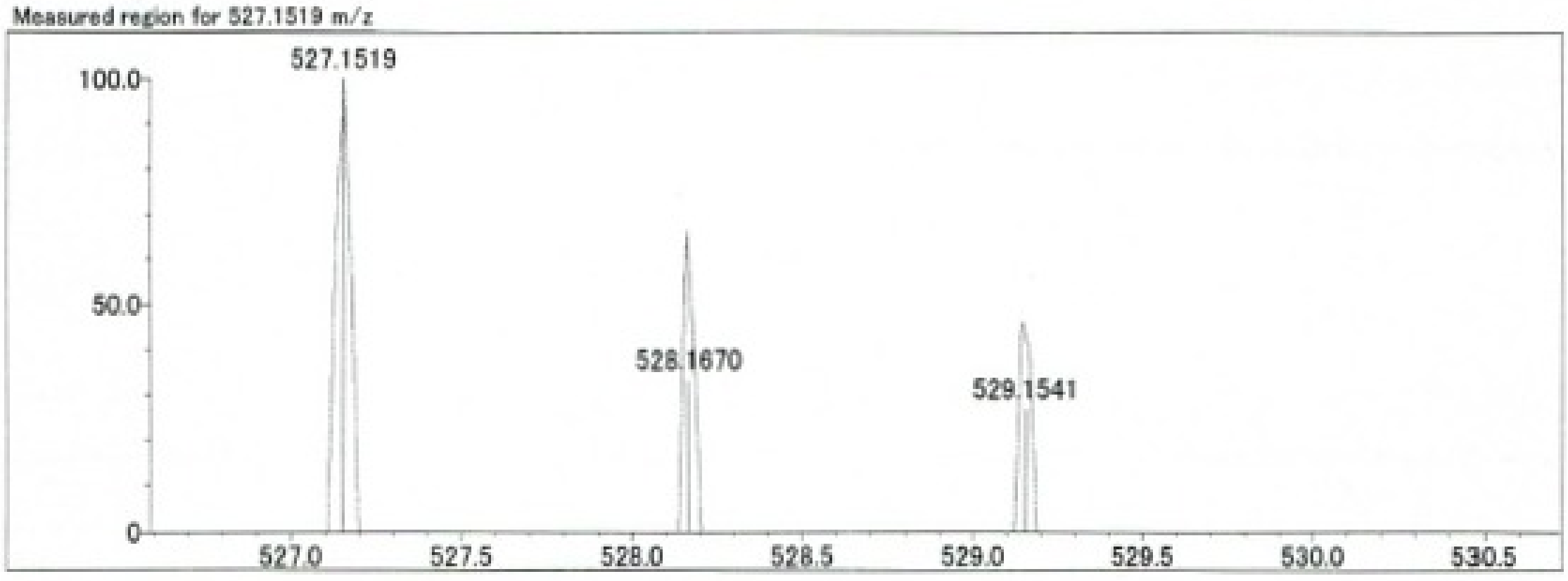

C23 H30 N2 O8 S2 [M+H]+ : Predicted region for 527.1516 m/z

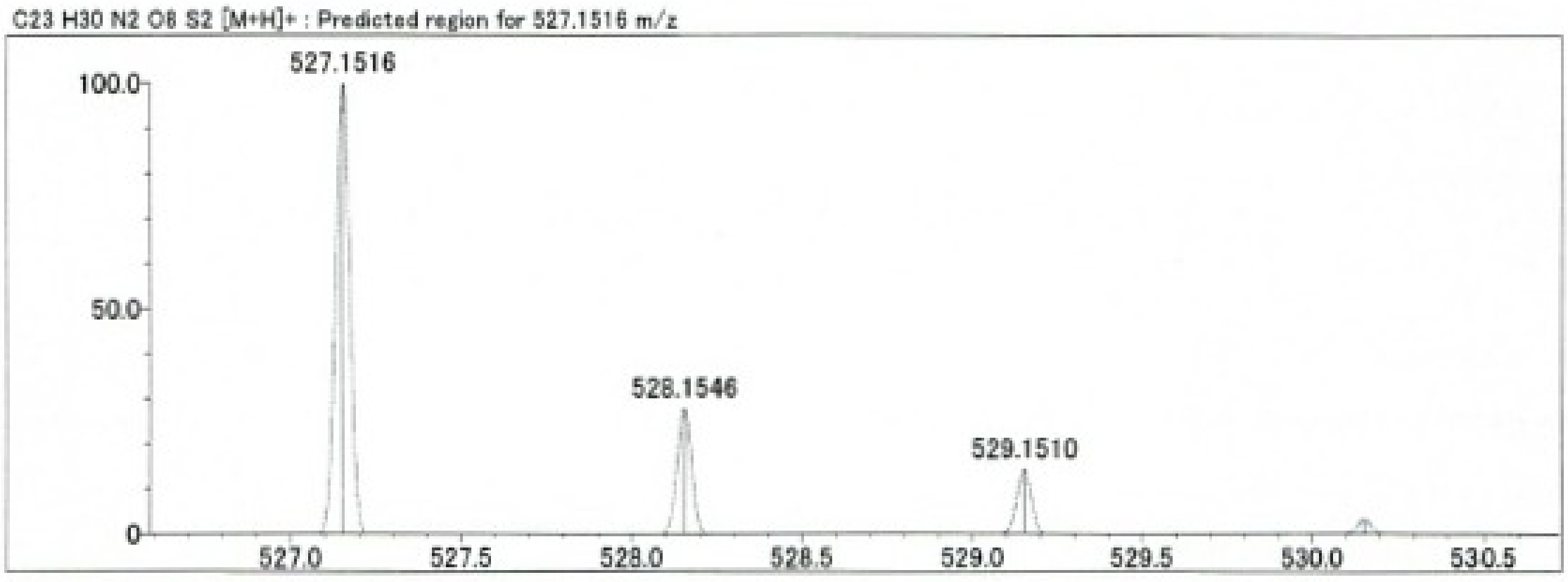

Rank	Score	Formula (M)	Ion	Meas. m/z	Pred. m/z	Df. (mDa)	Df. (ppm)	Iso	DBE
1	28.31	C23 H30 N2 O8 S2	[M+H]+	527.1519	527.1516	0.3	0.57	28.31	10.0
